# The diabetes-obesity-hypertension nexus in Qatar: evidence from the World Health Survey

**DOI:** 10.1186/1478-7954-12-18

**Published:** 2014-08-28

**Authors:** Faleh Mohamed Hussain Ali, Zlatko Nikoloski, Husein Reka, Orsida Gjebrea, Elias Mossialos

**Affiliations:** 1 Qatar Supreme Council of Health, Doha, Qatar; 2 London School of Economics, Houghton Street, WC2A 2AE London, UK

**Keywords:** Diabetes, Determinants of diabetes, Qatar, Middle East, Obesity

## Abstract

**Background:**

As countries develop economically, an “epidemiological transition” occurs whereby a set of chronic diseases increasingly becomes a country’s health challenge. Against this background, this paper examines the most common conditions associated with the prevalence of diabetes in Qatar, with a specific focus on the diabetes-obesity-hypertension nexus.

**Methods:**

We analyzed data from the World Health Organization’s World Health Survey conducted in the State of Qatar in 2006. The survey included demographic, anthropometric, and blood chemistry measurements. Using multivariate logistical regression analysis, we assessed the most common conditions associated with diabetes, using both objective and subjective measures of diabetes. The objective measures relied on random blood sugar tests, and the subjective measure included respondents who affirmatively answered the question on diabetes diagnosis. We repeated our analysis on respondents who had blood glucose levels high enough to be considered diabetic/glucose intolerant but did not answer affirmatively on the question of diabetes diagnosis.

**Results:**

When using the objective measure of diabetes, the following conditions appeared significant: obesity (OR = 1.5, 95% CI = 1.2 – 1.9), higher income (OR = 1.4, 95% CI = 1.0 – 1.9), high cholesterol (OR = 1.4, 95% CI = 1.0 – 1.9), having Qatari origin (OR = 1.3, 95% CI = 1.0 – 1.7), and increasing systolic blood pressure (SBP) 120–139 mmHg (OR = 1.5, 95% CI = 1.2 – 2.0), SBP 140–159 mmHg (OR = 2.2, 95% CI = 1.6 – 3.1), SBP > 160 mmHg (OR = 3.2, 95% CI = 2.0 – 5.3). Similar results were obtained using the subjective measure of diabetes as a dependent variable. When applied to the group of respondents that included pre-diabetics and those who did not know they were diabetic, obesity and hypertension appeared as the only statistically significant explanatory variables.

**Conclusion:**

High prevalence of diabetes, hypertension, and especially obesity is documented among residents of Qatar. Further steps are required to tackle the most common conditions associated with the rising diabetes epidemic in the country, which might also pose significant fiscal challenges in the future.

## Background

As countries move up the income ladder, they undergo health status changes termed the “epidemiological transition”. While communicable diseases are more prevalent in low-income and lower-middle-income countries, chronic diseases take a significant toll in upper-middle-income and high-income countries. Some of the extant research has documented that the burden of chronic diseases is particularly high among countries that have undergone a relatively fast transition from middle- to high-income status [1,2].

One of the countries that has witnessed such rapid development is the State of Qatar. Qatar has recently emerged as the richest country in the world in terms of gross domestic product (GDP) per capita. The World Bank’s World Development Indicators reported that the GDP per capita (in constant PPP (purchasing power parity)) amounted to 78,000 USD in 2011. This rapid economic development has been coupled with significant improvements in human capital, both in terms of education and health. Qatar managed to make significant strides in increasing life expectancy (which increased from 59 years in 1960 to 78 years in 2011). In the same vein, infant mortality (one of the main indicators of economic development and well-being) has dropped by a significant margin – from around 44 in 1968 to 6.4 deaths per 1,000 live births in 2011.

Despite these notable improvements, there are significant challenges that lie ahead, in terms of human development, and especially health. The World Health Organization (WHO) World Health Survey (WHS) has identified three main health challenges in Qatar: (i) premature death and catastrophic injury from road trauma, workplace accidents, and infant and early childhood mortality; (ii) early onset of preventable cancer and diabetes, particularly those where genetic factors may make the local population more vulnerable; and (iii) lifestyle diseases that reduce life expectancy and quality of life, such as obesity and respiratory disease related to smoking and stress-related mental health diseases [3].

However, the diabetes problem is not endemic to Qatar. Five of the top 10 countries for diabetes prevalence in 2010 were in the Gulf region. Prevalence estimates for 2030 (based on anticipated changes in population size and demography) suggest that this will remain the case [4]. These high rates of diabetes in the region have precipitated significant research interest aimed at identifying risk factors that may explain the high diabetes prevalence. A recent literature review documented the available research in the context of diabetes and its risk factors in the Gulf countries [4].

In the context of Qatar, risk factors associated with diabetes were examined in one study [5]. As expected, obesity, hypertension, metabolic syndrome, and heart disease were found to increase the likelihood of having diabetes. In addition, smoking habits and family history of diabetes were major contributors to diabetes. However, while the findings of the study could be a first step, the results should be interpreted with care. Given that the data were collected from primary health centers, it is highly likely that the sample was biased, because it examined a subsection of the population that was unwell and already seeking medical attention. As such, the sample was not a good representation of the entire Qatari population, and the link between diabetes and the conditions most commonly associated with it could be better determined by using a nationally representative sample of individuals. The STEPwise approach to Surveillance (STEPS) survey has been developed by the WHO in order to monitor the extent of chronic diseases and their risk factors in member countries. Whilst informative in nature, the STEPS survey in Qatar only covers Qatari nationals aged between 18 and 64 years, thus rendering it imperfect in terms of representativeness of the entire population [6].

Shedding more light on the nexus between diabetes and its most common conditions is an important research question, particularly given that diabetes is associated with significant complications. Diabetes increases the risk of cardiovascular diseases such as ischemic heart disease, stroke, and peripheral cardiovascular disease [7], and the literature suggests that people diagnosed with diabetes have much lower quality of life compared to people without the condition [8]. The same literature suggests that with a few simple steps (such as weight control and exercise) diabetes mellitus type 2 could be delayed or avoided altogether [8].

Against this background, the main objective of this study was to use the WHO World Health Survey for Qatar (2006) in order to ascertain factors associated with higher prevalence of diabetes. In conducting the analysis, we first used an objective measure of diabetes, i.e., one based on blood samples. Furthermore, we applied our main model to a “subjective” measure of diabetes, i.e., people who have been diagnosed with diabetes. Finally, we conducted a third exercise to explore the link between some of our explanatory variables for people that were pre-diabetic and those who did not know that they were diabetic.

## Methods

### Sampling and data collection

The analysis was based on the WHO World Health Survey conducted for the State of Qatar in 2006 [3]. The WHS series was developed by the WHO as a means to compile comprehensive baseline information on the health of populations in different countries. The survey was implemented by a team of experts from Qatar, drawn from the field of public health, epidemiology, and statistics. Implementation was assisted by a technical team from the WHO [3]. The Qatar WHS is a nationally representative survey. To ensure that the sample households and individuals who were interviewed were representative of all of Qatar, a detailed sampling design was implemented. The sampling design took into account the WHO requirements specified in the WHS sampling guidelines for participating countries. A detailed description of the sampling (as well as other methodological notes on the survey) can be found in the WHO Qatar WHS document [3] (Additional file 1).

The sampling process entailed selection of 6250 households in order to arrive at the desired sample coverage (given a nonresponse rate of 15% to 20%). As there is no adequate population register in Qatar that can be used as a sampling frame, the sampling frame was constructed using the administrative organization of Qatar. Qatar is divided into 10 municipalities, which are divided into one or more zones. These zones are further divided into blocks, which form the smallest administrative unit in the country. There are currently 98 zones and 2392 blocks, although not all of the zones and blocks are currently inhabited. The average number of people living in each block is about 300, although there is large variation in this number. To obtain the sampling frame separately for Qatari and non-Qatari households, the country was divided into primary sampling units (PSUs). The Qatari sampling frame consisted of 394 PSUs, while the non-Qatari frame consisted of 665 PSUs.

The WHS includes standard variables (age, gender, marital and educational status) and self-reported measures of diabetes, smoking habits, and lifestyle (exercise and the amount of fruits and vegetables consumed on a daily basis). In addition, WHS includes data on levels of blood glucose, hypertension, and cholesterol. The total number of the Qatar sample was 4775, with blood samples taken from 3337 of these. All respondents provided information on the basic socio-economic and demographic variables. Table 1 gives a breakdown of the age, nationality, marital status and education levels of respondents, according to whether or not they gave blood. There were no evident differences between the people who gave blood and people that did not give blood. This is especially the case between Qataris and non-Qataris.

**Table 1:** Socio-demographic characteristics of people who gave blood and those that did not

	**Blood (%)**		**No blood (%)**	
	**N = 3337**		**N = 1438**	
**Age**				
0-19	3.52		3.20	
20-29	19.84		23.78	
30-39	34.35		35.95	
40-49	26.34		24.26	
50-59	12.13		8.65	
60 and over	3.82		4.16	
**Gender**				
Male	47.76		49.48	
Female	52.24		50.52	
**Education level**				
Illiterate	0.10		0.09	
Less than secondary	13.18		10.90	
Secondary and above	86.72		89.01	
**Marital status**				
Married	79.98		77.58	
Non married	20.02		22.42	
**Nationality**				
Qatari	46.83		54.01	
Non-Qatari	53.17		45.99	
	**Blood (%)**		**No blood (%)**	
	**Qatari**	**non-Qatari**	**Qatari**	**non-Qatari**
	**N = 1556**	**N = 1781**	**N = 778**	**N = 660**
**Age**				
0-19	4.69	2.55	4.01	2.26
20-29	22.24	17.71	27.3	19.51
30-39	32.20	36.22	34.57	37.63
40-49	25.06	27.45	22.7	26.13
50-59	10.41	13.64	5.79	12.02
60 and over	5.40	2.43	5.64	2.44
**Gender**				
Male	39.72	54.84	46.88	54.80
Female	60.28	45.16	53.12	45.20
**Education level**				
Illiterate	0.14	0.06	0.16	0.00
Less than secondary	19.16	8.46	14.56	7.00
Secondary and above	80.69	91.49	85.28	93.00
**Marital status**				
Married	69.15	89.52	69.97	86.41
Non married	30.85	10.48	30.03	13.59

Data taken from WHO World Health survey [3].

As stated in the WHS report, throughout the implementation of the survey, all ethical procedures were followed. This included at the design, training and implementation stages. All the participants were assured that the information provided would be confidential and would not be used for any reason apart from scientific purposes. It was stressed to the participants that they had the right to refuse participation and to withdraw from participation at any time [3].

### Data analysis

#### Diabetes

We used two variables to define people with diabetes. In the first instance, we used an objective measure of diabetes, with those respondents who had a random blood sugar concentration higher than 140 mg/dl defined as having diabetes [9]. This threshold for diabetes included people considered to have impaired glucose tolerance, but differentiating these would require follow-up testing, which is not possible with the WHS. Hence, in order to improve inference of the most common conditions associated with type 2 diabetes, we included respondents with pre-diabetes in our objective measure of diabetes. As a second measure of diabetes, we used the self-reported WHO question: “Have you ever been diagnosed with diabetes (high blood sugar)?” Finally, we repeated the analysis on a group that represented the difference between objective and subjective measure of diabetes. Effectively, this group encompassed people with a random blood sugar level high enough to be considered diabetic (although they did not report that they had been diagnosed with diabetes) and (given our definition of objective diabetes) people that are considered to have impaired glucose tolerance levels, i.e., pre-diabetics.

#### Cholesterol

Within the WHS, levels of total, low-density lipoprotein, and high-density lipoprotein cholesterol are determined. For the purpose of the present study, the high cholesterol group was defined as those people that had a total cholesterol level of 240 mg/dl or higher, as defined by the WHO.

#### Hypertension

Blood pressure was also measured, and four categorical variables were defined that corresponded to the mean systolic blood pressure (SBP) of the respondents. The four categories were: less than 120 mmHg, 120–139 mmHg, 140–159 mmHg, and 160 mmHg or greater. In addition, another categorical variable of hypertension was defined, which corresponded to an SBP higher than 140 mmHg.

WHS also allows for the construction of measures of obesity. Whilst the ideal measures of obesity would include waist circumference and waist-to-hip ratio, the available data gathered through WHS allowed only for the construction of one, albeit standard, measure of obesity – BMI (body mass index, which is defined as weight in kilograms, divided by the square of height in meters). Following the WHO recommendations, BMI was grouped into four separate categories, and respondents with a BMI between 25 and 30 kg/m^2^ were classified as overweight, while the ones with BMI equal to or greater than 30 kg/m^2^ were classified as obese. For the purpose of our analysis we only used the categorical variables of obese (≥30 kg/m^2^) and non-obese (<30 kg/m^2^) individuals.

It is important to state that in our final analysis we included only individuals aged 20 years and over. There were two reasons for this decision. First, the epidemiological origin of diabetes mellitus type 2 and diabetes mellitus type 1 is different. Given that the latter occurs in the youngest population (aged 0–19 years) and that inclusion of this group might induce bias in our study, we decided to omit them. Second, the number of children with diabetes was extremely low (one aged 0–10 and 14 aged 11–19 years) and, thus, they could be excluded.

In addition to the variables above, we included further anthropometric and demographic variables: age (grouped in 10-year intervals), gender (male and female), and marital status (married or unmarried, with the latter category including separated, divorced, or widowed). We also included three variables that are considered to be significant determinants of diabetes prevalence: smoking, healthy eating habits (such as the consumption of at least five fruits and vegetables per day), and exercise patterns. However, it should be noted that the causal link between diabetes and the last two variables in a one-off cross-section sample could be very difficult to establish, and there is limited possibility to infer whether a higher blood sugar level (or possible diabetes diagnosis) influences healthy eating patterns or if the reverse is true. We also controlled for the level of education attainment, classified into three groups: illiterate, at least primary education, and at least secondary education. Poor people are more at risk for developing diabetes mellitus type 2 and, once they have it, are more likely to suffer complications, indicating a link between living conditions (i.e., social determinants of health) and diabetes mellitus type 2 as well as other ailments [10]. Therefore, income/consumption patterns also could be significant determinants of diabetes and, hence, we included categorical variables for income, defined as poor (the lowest income quintile) and non-poor (everybody else).

In addition, we performed two robustness checks: (i) we included dummies for the income quintiles 1–4 (i.e., we used the highest quintile as a reference group). Whilst our results were intuitive (lowest quintile had a negative coefficient, whilst the dummies for the other quintiles had positive but minuscule coefficients), the variables of interest were insignificant; (ii) we then included the dummies for income quintile 2–5 (i.e., we took the poor as a reference group) – here, our results were again intuitive – the likelihood of having diabetes increased with income (though only the dummies for income quintile 2 and 3 were significant and only at the 5% level of significance).

Finally, Qatar has witnessed a significant migration of foreign nationals in the past decade, so controlling for the Qatari/non-Qatari cohorts was also warranted. Unfortunately, the WHS does not distinguish between white-collar Western non-Qatari migrant workers and blue-collar ones who usually come from countries in South Asia.

### Statistics

We used multiple logistic regression as the main method to analyze which factors were significantly correlated with the prevalence of diabetes. The logistic regression was repeated twice – the first time on the objective measure of diabetes and then on the self-reported measure of diabetes. In addition, we repeated the analysis on the third group of respondents that encompassed respondents that were pre-diabetic or did not know they were diabetic. The independent variables were selected based on literature review and availability in the WHS questionnaire. Because most of the included variables were categorical, we resorted to using dummy variables to test robustness of the data. Results are presented as crude and adjusted odds ratios, with associated 95% confidence intervals. All analyses were conducted in STATA 11.

## Results

Table 2 provides information on diabetes prevalence, hypertension, and BMI among the selected population of respondents, with crude significance levels between various subgroups. The table indicates that the three variables of diabetes, hypertension, and BMI vary significantly by age, education status, and, to some extent, income and nationality. Hypertension affected almost one-fifth of the Qatari population and was also fairly high among non-Qataris. Similar to other countries in the Gulf region, obesity appeared to be a significant problem in Qatar. More than a third of the adult population had a BMI equal to or higher than 30 kg/m^2^, which is a first indication of obesity.

**Table 2:** Prevalence (%) of diabetes, hypertension and obesity by selected variables

	**Diabetes**	***p values***	**Hypertension**	***p values***	**BMI > 30 p values**	***p values***
Total	16.42		18.69		35.37	
**Age**		*p = 0.000*		*p = 0.000*		*p = 0.341*
20-29	8.95		9.77		27.91	
30-39	12.53		10.96		36.52	
40-49	20.00		23.79		40.19	
50-59	28.04		37.24		38.82	
>60	29.92		58.70		39.86	
**Sex**		*p = 0.827*		*p = 0.000*		*p = 0.000*
Male	16.19		24.41		32.45	
Female	16.47		13.43		38.13	
**Marital status**		*p = 0.255*		*p = 0.696*		*p = 0.012*
Married	16.71		18.79		36.42	
Not married	14.89		18.17		31.56	
**Education level**		*p = 0.000*		*p = 0.000*		*p = 0.000*
Less than secondary	19.41		27.39		39.42	
Secondary or above	14.60		15.72		33.78	
**Smoker**		*p = 0.430*		*p = 0.039*		*p = 0.000*
Yes	15.03		22.04		28.14	
No	16.53		18.18		36.54	
**BMI**		*p = 0.000*		*p = 0.000*		
<18.5	6.80		14.05			
18.5-24.99	12.50		11.09			
25-29.99	14.40		19.69			
>30	21.45		24.60			
**Cholesterol**		*p = 0.000*		*p = 0.000*		*p = 0.000*
High	22.96		31.70		43.09	
Normal and Low	15.20		16.98		12.10	
**SBP**		*p = 0.000*				*p = 0.000*
<120	10.47				27.20	
120-139	16.46				36.67	
140-159	24.69				44.42	
>160	36.05				49.34	
**Income**		*p = 0.042*		*p = 0.399*		*p = 0.044*
Poor	14.17		19.80		32.10	
Non poor	16.92		18.43		36.13	
**Nationality**		*p = 0.009*		*p = 0.735*		*p = 0.000*
Qatari	18.12		18.92		42.12	
non Qatari	14.77		18.49		29.09	

BMI = Body Mass Index; SBP = Systolic Blood Pressure, p values reported for Chi squared test for comparing proportions.

Diabetes - random blood sugar higher than 140 mg/dl; Hypertension - systolic blood pressure higher than 140 mmHg.

BMI - individual’s body mass divided by the square of their height, in kg/m2.

Our main findings from the multivariate logistical regression, conducted using our objective definition of diabetes, are shown in Table 3. We found a strong association between diabetes and the independent variables of being obese and suffering from a cardiovascular disease (hypertension); having a higher level of cholesterol was also associated with a higher probability of having diabetes.

**Table 3:** Significant factors associated with objective measure of diabetes (i.e. respondents with random blood sugar levels higher than 140 mg/dl)

	**No diabetes**	**Diabetes**	**Crude OR (95% CI)**	**Adjusted OR**^**† **^**(95% CI)**
	**n(%)**	**n(%)**		
**Body Mass index**				
Obese	890 (78.6)	243 (21.45)	1.7*** (1.5 - 2.2)	1.5*** (1.2 - 1.9)
			(0.109)	(0.112)
**Societal status**				
Poor	521 (85.83)	86 (14.1)	-	-
Non poor	2268 (83.1)	462 (16.9)	1.2** (1.0 - 1.6)	1.4** (1.0 - 1.9)
			(0.127)	(0.156)
**SBP (mmHg)**				
<120	975 (89.5)	114 (10.5)	-	-
120-139	1254 (83.5)	247 (16.5)	1.7 (1.3 - 2.1)	1.5*** (1.2 - 2.0)
			(0.106)	(0.134)
140-159	360 (75.3)	118 (24.7)	2.8*** (2.1 - 3.7)	2.2*** (1.6 - 3.1)
			(0.134)	(0.171)
>160	94 (63.9)	53 (36.1)	4.8*** (3.2 - 7.1)	3.2*** (2.0 - 5.3)
			(0.203)	(0.247)
**Cholesterol**				
High cholesterol	443 (77.0)	132 (22.9)	1.6*** (1.3 - 2.0)	1.4** (1.0 - 1.9)
			(0.094)	(0.133)
Normal and low cholesterol	2309 (84.8)	414 (15.2)	-	-
**Nationality**				
Qataris	1274 (81.9)	282 (18.1)	1.2** (1.0 - 1.5)	1.3** (1.0 - 1.7)
			(0.093)	(0.110)
Non Qataris	1515 (85.0)	266 (14.9)	-	-

Objective measure of diabetes defined as a random blood sugar levels higher than 140 mg/dl. Total number of observations used for the logistic regression is 2981. In addition to the reported variables, the model controls for the following: level of education, gender, age (grouped in 5 ten-year groups), consumption of fruits and vegetables as well as exercise patterns. ^†^Odds ratios adjusted for all variables included in the model. ***significant at 1%, **significant at 5%. Standard errors are reported in the line below the respective coefficients.

Our analysis revealed that blood pressure was also significantly associated with the occurrence of diabetes in Qatar. Adjusted odds ratios increased from 1.5 at SBP 120–139 mmHg to 2.2 at SBP 140–159 mmHg and to 3.2 at SBP 160 mmHg or higher. Like most of the other epidemiological studies in the Gulf area, we found a strong linear relationship between SBP and likelihood of diabetes. As expected, obesity was a significant explanatory variable for the prevalence of diabetes, with BMI ≥30 kg/m^2^ associated with a 1.5-times higher likelihood of having diabetes. This is worrisome given the high rates of overweight and obesity in the country. Furthermore, high levels of cholesterol were also associated with higher prevalence of diabetes, with cholesterol ≥240 mg/dl associated with 1.4-times higher prevalence of diabetes. We also found that nationality played a significant role, with Qataris having 1.3-times more likelihood of having diabetes than non-Qataris.

Finally, we found a significant link between income and likelihood of having diabetes. Compared with being in the lowest quintile, higher income was associated with 1.4-times greater likelihood of having diabetes. After controlling for other factors, age was not found to be significantly associated with the likelihood of having diabetes.

The results of the analysis using the self-reported diagnosis of diabetes as a dependent variable are summarized in Table 4. It appeared that conditions associated with the objective measure of diabetes were also associated with this subjective measure of diabetes. Obesity and cardiovascular disease were the main independent variables associated with diabetes. Similarly, we found significance for other (secondary) conditions of diabetes, such as socio-economic status or nationality. In this analysis, we also found evidence that the prevalence of diabetes increased with age. However, in contrast, we did not find statistically significant evidence that cholesterol was associated with a higher prevalence of diabetes. The magnitude of the coefficients of interest in this analysis was comparable to the magnitude of coefficients of interest in our main model.

**Table 4:** Significant factors associated with subjective measures of diabetes

	**No diabetes**	**Diabetes**	**Crude OR (95% CI)**	**Adjusted OR**^**† **^**(95% CI)**
	**n(%)**	**n(%)**		
**Body Mass index**				
Obese	1132 (86.3)	179 (13.65)	2.0*** (1.6 - 2.6)	1.5*** (1.1- 1.9)
			(0.125)	(0.144)
**Societal status**				
Poor	797 (90.9)	80 (9.12)	-	-
Non poor	3344 (90.9)	336 (9.13)	1.2 (1.0 - 1.6)	1.5** (1.0 - 2.2)
			(0.140)	(0.183)
**SBP (mmHg)**				
<120	1221 (94.9)	65 (5.05)	-	-
120-139	1643 (91.9)	145 (8.11)	1.5** (1.1 - 1.9)	1.2** (1.0 - 1.7)
			(0.125)	(0.112)
140-159	454 (83.3)	91 (16.7)	2.3*** (1.8 - 2.9)	2.0*** (1.3 - 3.0)
			(0.147)	(0.218)
>160	112 (69.6)	49 (30.43)	4.8*** (3.3 - 6.8)	3.6*** (2.1 - 6.2)
			(0.206)	(0.275)
**Nationality**				
Qataris	1960 (88.6)	253 (11.4)	1.7*** (1.4 - 2.1)	1.6*** (1.2 - 2.2)
			(0.111)	(0.139)
non Qataris	2181 (93.0)	163 (6.95)	-	-

Subjective measure of diabetes encompasses answering the question - have you been diagnosed with diabetes. Total number of observations used for the logistic regression is 3031. In addition to the reported variables, the model controls for the following: level of cholesterol, level of education, gender, age (grouped in 5 ten-year groups), consumption of fruits and vegetables as well as exercise patterns. ^†^Odds ratios adjusted for all variables included in the model. ***significant at 1%, **significant at 5%. Standard errors are reported in parentheses below the respective coefficients.

The crude and adjusted odds ratios for the analysis, encompassing the difference between our objective and subjective measures of diabetes are reported in Table 5. The main messages from this exercise were comparable to those from our first two analyses – namely that obesity and cardiovascular disease (hypertension) were the main independent variables associated with impaired glucose intolerance and prevalence of diabetes.

**Table 5:** Significant factors associated with a group of people that encompass pre-diabetics and those that do not know they are diabetic

	**No Diabetes**	**Diabetes**	**Crude OR (95% CI)**	**Adjusted OR**^**† **^**(95% CI)**
	**n(%)**	**n(%)**		
**Body Mass index**				
Obese	991 (87.9)	137 (12.15)	1.3*** (1.0 - 1.7)	1.2** (1.0 - 1.6)
			(0.119)	(0.127)
**SBP (mmHg)**				
<120	1000 (92.2)	85 (7.83)	-	-
120-139	1323 (88.4)	173 (11.56)	1.2 (1.0 - 1.5)	1.4** (1.0 - 1.9)
			(0.114)	(0.146)
140-159	410 (86.3)	65 (13.68)	1.4** (1.1 - 1.9)	1.8*** (1.2 - 2.7)
			(0.147)	(0.191)
>160	126 (85.7)	21 (14.29)	1.5** (1.0 - 2.2)	1.9** (1.1 - 3.4)
			(0.242)	(0.251)

This group of people is the difference between those that have diabetes with the objective measure and those that answer affirmatively to the question whether or not you have diabetes. Total number of observations used for the logistic regression is 2980. In addition to the reported variables, the model controls for the following: income levels, nationality, level of cholesterol, level of education, gender, age (grouped in 5 ten-year groups), consumption of fruits and vegetables as well as exercise patterns. ^†^Odds ratios adjusted for all variables included in the model. ***significant at 1%, **significant at 5%. Standard errors are reported in parentheses below the respective coefficients.

As a robustness check, we repeated the analysis on two subsamples, one consisting entirely of Qatari respondents and the other one of non-Qatari respondents. The results obtained from this analysis closely correspond to the findings obtained when analyzing the entire sample.

## Discussion

Four findings stem from this paper analysing WHS data from Qatar. Firstly, there was a significant relationship between blood pressure and likelihood of having diabetes, which is consistent with previously published research [11-14]. We found a positive and linear relationship between high blood pressure and diabetes prevalence. Secondly, there was a significant relationship between obesity and prevalence of diabetes, also confirmed by previous studies [5]. Our results indicated that obesity is a significant problem in Qatar; almost a third of the overall population in the country would be considered obese on the basis of BMI values. Thirdly, the results suggest that high cholesterol is a problem in Qatar and was also associated with a higher prevalence of diabetes, consistent with other studies [5]. Finally, Qataris had a higher likelihood of having diabetes compared to non-Qataris. Such a relationship has been documented in the context of the United Arab Emirates, with nationals showing a higher likelihood of diabetes compared with non-nationals [15].

Obesity appears to be a particular problem for the region of Doha, where almost 40% of the population is currently considered obese. According to other estimates, about 45% of adult urban Qataris are obese (with over 70% being overweight). The obesity problem in Qatar is a recent phenomenon, which is closely linked with the economic development that the country has sustained in the last few decades [16]. Rapid socio-economic development had led to increased ownership of cars, a rapid increase in disposable income among Qatari families (especially among urban dwellers), as well as significant changes in dietary and lifestyle patterns. Finally, the development has been associated with an increase in the consumption of high fat caloric-dense food, increased consumption of refined sugar and salt, increased rates of smoking, and decreased exercise [17,18]. All of this has resulted in an increase of the overall obesity rate in the country. Obesity is not only a problem in itself but also has significant health implications as it increases the overall health costs of Qatari families [19]. A simple t-test on the health costs of families with obese and non-obese members revealed that the costs of the former were much higher.

It is also worth noting that in this study we controlled for additional factors that might drive the likelihood of having diabetes: education level, lifestyle factors (diet and exercise), and gender. However, most of the additional variables were not significant, possibly suggesting that some of the additional factors, such as diet and exercise, might interact with others, such as the education variables. In some of the comparable published studies (in Oman, for example), the authors found a strong link between education and diabetes, especially in urban areas [20], although these studies did not control for lifestyle factors. However, our results were comparable to the findings of similar studies conducted on the rest of the countries in the region [21-25].

Our results (at least on the descriptive side) are similar to the results gathered through the Qatari STEPwise Survey [6]. According to the survey, about 12.7% of Qataris had been diagnosed as diabetic within the previous 12 months. Moreover, the survey found that the diabetes prevalence in the country was 16.7%. This is higher than the prevalence of diabetes reported in the rest of the countries in the Eastern Mediterranean and also much higher than the diabetes prevalence rate in other high income countries. Whilst the results from the WHS and STEPwise survey were similar, two important caveats prevent direct comparison between results, which are that the STEPwise survey was conducted on Qatari nationals only and was also only conducted on people aged 18 to 64 years.

The present paper adds to the existing literature in the area of diabetes research in a few crucially important ways. Firstly, using the WHO World Health Survey, we conducted an analytical exercise of the nexus between diabetes and its main determinants. In that respect, we have added another country study to a fledgling literature on diabetes in the Gulf region (extant studies have focused on Saudi Arabia, Oman, United Arab Emirates, and Kuwait). Secondly, similar to the other literature we found a strong association between certain chronic conditions and diabetes, specifically obesity and hypertension. Thirdly, the chronic conditions of obesity and hypertension were found to be important regardless of whether an objective (blood chemistry) or a subjective (previous diagnosis) measure of diabetes was used.

Finally, our results carry significant weight with regard to Qatar health policy. A few possible ways to address the diabetes epidemic in the country include campaigns (both on national and micro/general practitioner/clinic level) that would promote regular testing of pre-diabetic cases, paying particular attention to daily calorie intake and the type of food that is consumed on a daily basis, as well as regular exercise.

## Limitations

This study was limited by the cross-sectional nature of the data, which did not provide any indication of the direction of effect or causality. This limitation also prevented any analysis of temporal changes in prevalence of diabetes and factors associated with diabetes. In that respect, while we included controls for diet and lifestyle, it was not possible to identify whether the lifestyle changes precipitated changes in diabetes levels or vice versa. Longitudinal studies would serve as a complement to the present study, especially in order to determine causality and the directional effects of the explanatory variables.

## Conclusion

Building on the WHO’s World Health Survey, our paper sheds more light on the common conditions associated with diabetes in Qatar. We conducted a multivariate logistical regression analysis on both the entire sample and on two stratified samples based on residence and we found the following: (i) obesity was associated with a higher propensity of having diabetes; (ii) high systolic blood pressure was also associated with higher prevalence of diabetes. Specific analyses including dummy variables demonstrated the robustness of the results.

Our findings are important with regard to health policy. As indicated by Guy and Nunn [26], because some chronic diseases take their toll in middle- and high-income countries, there is a danger that improvements, such as higher life expectancy and lower mortality (achieved at the height of the economic boom) could be reversed. This is becoming more worrying, given that some of the conditions that we have discussed (such as high cholesterol or obesity) are already high in Qatar and continue to have an upward trend.

While our study is the first step, there is more to be done in order to devise policy decisions aimed at tackling the existing health challenges in Qatar. More importantly, the right policy decisions should be based on a careful and sound analysis. Hence, constructing a longitudinal study where individuals would be surveyed in the medium term could: (i) create the basis for a thorough understanding of risk factors associated with diabetes; and (ii) enable the right policy choices to be made.

## Consent

Written informed consent was obtained from the patient for the publication of this report and any accompanying images.

## Competing interests

The authors declare that they have no competing interests.

## Authors’ contribution

FHMA and EM provided the overall guidance/framework for the paper. The analysis and literature review was jointly done by ZN, HR and OG. All authors participated in drafting of the final manuscript. All authors read and approved the final manuscript.

## Supplementary Material

Additional file 1 World Health Survey Qatar. Click here for file
