# Supplementary material for: The diabetes-obesity-hypertension nexus in Qatar: evidence from the World Health Survey
Source: Popul Health Metr. 2014 Aug 28;12:18. doi: 10.1186/1478-7954-12-18 (PMC4148083; doi:10.1186/1478-7954-12-18)
Supplement: Additional file 1 — World Health Survey Qatar. [file 1478-7954-12-18-S1.pdf]

World Health Survey
Qatar

Contents

1. Introduction 1
1.1 Health system in Qatar 2
1.2 Health system performance in Qatar 5
1.3 Sociodemographic profile 7
1.4 Health-related surveys in Qatar 8

2. Methodology 9
2.1 Introduction 9
2.2 Sample design 9
2.3 Questionnaire design 13
2.4 Training of the data collectors 15
2.5 Quality assurance 16
2.6 Ethical considerations 16
2.7 Data analysis 17
2.8 Limitations of the survey 17

3. Profile of the household population and individual respondents 18
3.1 Introduction 18
3.2 Household population profile 18
3.3 Sociodemographic profile of individual questionnaire respondents 23
3.4 Work history and benefits 27

4. Risk factors for poor health 35
4.1 Introduction 35
4.2 Use of tobacco, nutrition, physical activities and obesity 35
4.3 Blood pressure 46
4.4 Blood chemistry 49
4.5 Access to drinking-water and sanitation 53
4.6 Fuel for cooking 54

5. Morbidity prevalence 57
5.1 Introduction 57
5.2 Noncommunicable diseases 57
5.3 Coverage for vision care 69
5.4 Coverage for oral health 69
5.5 Injuries and treatment of injuries 70
5.6 Female health care and screening 72
5.7 Care during pregnancy and childbirth 74
5.8 Child health care (preventative and curative) 77
5.9 Reproductive and sexual health care 85

6. Health state valuation 87
6.1 Introduction 87
6.2 General health rating 89
6.3 Mobility 91
6.4 Self-care 93
6.5 Pain and discomfort 95
6.6 Cognition 97
6.7 Interpersonal activities 99
6.8 Vision 101
6.9 Hearing 103
6.10 Sleep and energy 105
6.11 Affect 106
6.12 Breathing 110
6.13 Functioning assessment 112

7. Health system responsiveness 116
7.1 Introduction 116

7.2 Self-assessed need for health care 116
7.3 Need for outpatient health care 117
7.4 Health systems responsiveness 119
7.5 Health provider information 131
7.6 Overall satisfaction with the health care system 137

8. Health expenditure, family support networks and transfers 143
8.1 Introduction 143
8.2 Health expenditure 143
8.3 Household and family support networks and transfers 150

9. Social capital and subjective well-being 158
9.1 Introduction 158
9.2 Social capital 158
9.3 Subjective well-being and quality of life 166

10. Major findings and policy recommendations 171
10.1 Introduction 171
10.2 Public health in Qatar 172
10.3 The Qatari health system 176

11. References 179

1. Introduction

The World Health Organization (WHO) has developed a World Health Survey (WHS) to compile comprehensive information on the health of populations, on the outcomes associated with the investment in health systems and baseline evidence on the way health systems are currently functioning; and to extend the ability to monitor inputs, functions and outcomes.*

Objectives of the WHS

- Develop a means of providing valid, reliable and comparable health information
- Build the evidence base to monitor health systems achievement of desired goals
- Provide policy-makers with the evidence they need to draft policies and strategies

The WHO World Health Survey was developed in Qatar and other Gulf Cooperation Council (GCC) countries through consultation with policy-makers and in collaboration with the people involved in routine health information systems. Training and other preparations for the WHS are complementary to health information systems team efforts to ensure data input in a cost-effective way and that important gaps in health information are covered. The WHS also assists with the establishment of a baseline for the health of a population prior to scale-up health interventions and activities.

The Qatari National Health Authority (NHA) successfully conducted the first World Health Survey in cooperation with WHO's Regional Office for the Eastern Mediterranean and the planning council of the Qatar Statistical Authority in 2006. The Qatar WHS country tables were first available in April 2008. These tables provide basic tabulations from each of the different modules, which include household population and individual respondents, health risk factors, morbidity prevalence, health status valuation, health system responsiveness, health expenditure and family support networks, including standard measures of tobacco use, nutrition, physical activities and obesity, current employment details and work history.

The Qatar 2006 World Health Survey was analysed by WHO headquarters experts in Geneva using standard methodologies with STATA statistical software. In May 2008, the draft technical report was jointly reviewed by the WHO headquarters and NHA technical teams.

Qatar is preparing to join other GCC countries for the WHS censuses in response to UN resolutions that call for all nations to conduct a census as close to 2010 as possible so that the data can be comparable with those of other nations. The NHA team has already begun taking steps to plan for World Health Survey 2010. In December 2008, NHA plans to host a UN statistical workshop on vital statistics data quality in Doha. It is expected this workshop will continue the ongoing coordination and cooperation among the GCC countries for the 2010 round of population and housing censuses. Joint meetings of the GCC countries will focus on sharing knowledge and coordinating the implementation of new technologies.

* http://www.who.int/healthinfo/survey/en/

1.1 Health system in Qatar

1.1.1 Qatar's health and wellness 2010

The health system in Qatar has been able to expand health services and health care delivery systems to ensure the provision of the highest quality of health care. Qatar hopes that the nation's health care system will match the best in the world. The current health system focuses on improving the health of people and maintaining their well-being by improved access to high quality health programmes and preventive services. The health system in Qatar seeks to create an outstanding and well managed health care system through capacity-building among highly qualified health professionals to gain skills with the most advanced medical technology. The health system in Qatar is looking forward to overcoming three major challenges:

- premature death and catastrophic injury from road trauma, workplace accidents, and infant and early childhood mortality
- early onset of preventable cancer and diabetes, particularly those where genetic factors may make the local population more vulnerable
- lifestyle diseases that reduce life expectancy and quality of life, such as obesity and respiratory disease related to smoking and stress related mental illness.

The establishment of the National Health Authority in Qatar was foundational for the creation of a health system framework to monitor all the effectiveness of health care service provision, and maintaining the public wellbeing. Measurable improvements in the health of the population are possible and necessary if we are to achieve a world class health system.

The National Health Authority was established by an Emiri Decision in 2005, replacing the former Ministry of Public Health. NHA was made responsible for guiding health system reform in Qatar and establishing one of the worlds most admired and renowned health systems in the world. The NHA's roles are to:

- create a clear vision for the nation's health direction
- set goals and objectives for the country
- regulate the medical landscape
- protect the public's health
- set the health research agenda
- monitor and evaluate progress toward achieving those objectives.

Building a healthy place to live and to work is considered a major priority for the NHA. To protect the public's health and safety, the NHA has a special role to play in ensuring that the public health of the country receives adequate attention. It oversees public health programmes related to the control of infectious diseases, and coordinates with other agencies on environmental, occupational and public safety promotion. Oversight of food and drugs will also be enhanced. Another key NHA function is to oversee the quality and effectiveness of service delivered by primary care providers, by providers in hospitals and in the public and private sectors to ensure that standards are met and performance targets achieved. It is the NHA's responsibility to ensure that the nation is prepared for any public health emergency. The basic requirements of a world-class health system are to be well prepared for any mass emergency and to learn from the experiences of others in preparing for and dealing with any disasters, such as outbreaks

of avian flu. The NHA does not provide clinical services. Instead our goal is to vest responsibility of care in the hands of public institutions such as Hamad Medical Corporation and the private sector while regulating, monitoring, and evaluating this care against agreed-upon outcomes. The NHA shoulders the responsibility of providing extensive staff training and development opportunities across all sectors to enhance workforce competence and confidence.

In short, it is the NHA's commitment to establishing an environment that promotes quality and wellness through wise policies in such areas as health insurance, information technology, licensure and credentialing, and continuing medical education.

In 2007, the board of the NHA published *Caring for the future*, its three year strategic direction. This signalled a far reaching programme to promote public health, encourage healthy lifestyles, provide community-based primary care and ensure that, when needed, some of the world's most advanced and highest quality care is available in tertiary medical facilities, which are also expected to be research leaders on the frontiers of science.

Caring for the future describes five strategic goals.

1. Building a healthy place to live and work:

- bolster programmes in public, environmental and occupational health
- enhance oversight of food and drugs
- emergency preparedness including ensuring the GCC is well prepared
- ensuring robust professional licensing and regulation of drugs and other treatments.

2. Ensuring a healthy population: the wellness model:

- create an independent institution to oversee primary care
- adopt a wellness approach and build new wellness centres
- implement information links between primary care and the tertiary sector, especially Hamad Medical Corporation (HMC).

3. Establish world-quality care in hospitals:

- foster innovation and quality improvement through decentralization and integrated public hospitals, creating competition, obtaining international accreditation and partnering with internationally recognized centres of excellence
- commission a master plan of hospital facilities and services to ensure integration
- ensure public centres of excellence: women's and children's hospitals, the Heart Centre for Cardiac Care, Al-Amal Centre for Cancer Care, a diabetes and obesity centre, and a neurosciences centre and mental health programme including psychiatric facility.

4. Encourage development of the private sector:

- develop policies to screen all future major hospital construction requests.

5. A better life for people with long-term conditions (LTC) and disabilities:

- produce a strategy for long-term conditions and disabilities to rapidly develop facilities and community-based services to enable independence for people with LTC and disabilities.

The National Health Authority is charged with providing preventive and treatment based public health services both in Qatar and supervising medical treatment of Qatari nationals abroad. Besides, it regulates the marketing and manufacturing of drugs in accordance with international quality standards, within the frame work of the public policy of the state and in accordance with a strategy aiming to realize the national health objectives.

The National Health Authority also supervises Hamad Medical Corporation, Hamad Specialist and Educational Hospital, private medical facilities, laboratories, pharmacies, councils of auxiliary medical professions, hospitals, primary health care centres and other public medical treatment utilities. The National Health Authority undertakes the organization of the medical private sector and it acts in coordination with the private health insurance system to improve the quality of health services and disseminate health education and awareness.

1.1.2 Preventive health care services

The vision of the NHA is to promote and protect the health of the population through partnership, innovation and action in health promotion. The NHA's role is to implement preventive health initiatives that are complementary to ongoing health care system performance improvements by improving access to public health services, expanding the capacity of occupational health clinics, promoting practical approaches to the collection and use of routine health information, and professional development of national health providers. Preventive health care service aims are to promote health education, provide health protection by preventing infectious disease and contribute to emergency preparedness. Public health service delivery is an information-intensive field that encompasses the disciplines of public policy, planning, clinical care, and computer science. The NHA's Public Health Department has established community outreach for vaccination and other preventive services, such as monitoring food quality and safety, providing health education, and insuring environmental health and safety. A health promotion division has been established that includes non-communicable disease and health education teams comprising four units: tobacco control unit, healthy lifestyle and nutrition, chronic diseases and accident prevention.

1.1.3 Primary health care

The primary health services department manages 23 primary health care centres, which are conveniently distributed across the country. It provides various programmes including health awareness; mother and child care; immunization against childhood diseases; diagnostic and treatment services of common and chronic diseases; medicines; nutritious food; clean water and ambulance and emergency services.
The state continues to set up health centres on the highways such as al-Shamal, Dukhan, Abu Samra and Mesaieed highways, in collaboration with the ambulance and emergency health services centres, thus effectively expanding health care activity outside Doha. In the same context, a number of developments have been made including the inauguration of a child emergency unit in al-Wakrah health centre and al-Matar health centre; enlarging the parking lot of Madinat Khalifa health centre; completing the emergency unit of al-Shamal health centre and rehabilitating some other health centres. Two health centres are going to be opened for the first time in Doha central market and the al-Kara'ana area on the road to Abu Samra.

Health commission services

The health commission department carries out medical checks on everybody entering the country for work or visit, and issues certificates of health fitness for such categories as those who are about to get married and who are applying for jobs, universities or public housing.

Hamad Medical Corporation (HMC)

Established in 1982, Hamad Medical Corporation is one of the region's most distinguished specialized medical establishments comprising five hospitals. Presently, the corporation manages the following hospitals: Hamad General Hospital, Rumaila Hospital, Women's Hospital, Al-Khor Hospital and Al-Amal Hospital, which is a specialty cancer treatment facility. HMC has won the Joint Commission International[*] certification for quality health facilities.

The private health sector

The private health sector comprises 166 private clinics and medical complexes, 131 dental clinics, 4 general hospitals and 1153 medical physicians. It plays a vital role in the provision of health care services in Qatar.

1.2 Health system performance in Qatar

Qatar has an opportunity to create a health care system that will provide the most effective and advanced health care to its people. The heart of Qatar's strategic vision for the future is helping people achieve their full potential, thereby benefiting the entire population, including individuals, their families, the community and the nation.

The NHA is embarking on an ambitious programme to enhance the wellness of the people of Qatar so that a vibrant, healthy and productive society can be established for today and for the future. The essence of that programme is set out in *Caring for the future*[†], the strategic document, published by the NHA in 2007, setting out the vision for health services to 2010.

An assessment of the health system performance in Qatar in terms of health outcome indicators shows improvement over the past 30 years. The crude death rate has declined from 2.5 per year in 1981 to 1.9 per year per 1000 inhabitants in 2005, based on the NHA vital statistics registration records[‡]. Although maternal neonatal health still needs improvement in Qatar, vital statistics records indicate that infant mortality has been halved from 17.0 per 1000 live births per year in 1981 to 8.2 per year in 2006. NHA is actively working with Hamad Medical Corporation to strengthen accurate and consistent reporting of miscarriages and neonatal deaths. Life expectancy remained steady over the past two decades and is estimated to be approximately 75 years for men and women.

Primary health services offer effective immunization and antenatal care programmes. The coverage of Qatari children immunized for measles reached 100% in 2005 comparing with 69% in 1983. The percent of pregnant women attended by skilled healthcare providers was reported to be 100% in 2006. In 1998, there were an estimated 12.6 physicians per 10 000 population and 28.9 trained, licensed nurses per 10 000 population. In 2006, the Qatar World Health Survey reported 27.6 physicians and a very

---

[*] http://www.jointcommissioninternational.com/
[†] http://www.nha.org.qa/jsp/content.jsp?menuId=2
[‡] The crude death rate is the number of deaths in a year divided by the population and multiplied by one thousand

large increase to 73.8 nurses per 10 000 population. The quality of health care is a priority in the Qatar health system.

Health infrastructure and health status profile

Table 1.1 presents the health manpower indicators[*] per 10 000 population in Qatar, according to the National Health Authority 2006 annual health report. As mentioned previously, the report indicates that there were 27.9 physicians and 73.8 nurses per 10 000 population.

Table 1.1 Qatar health human resources indicators per 10 000 population, 2006

Type of health worker	Number per 1000 population
Physician	27.6
Dentist	5.2
Nurse	73.8
Pharmacist	12.6
Other health care provider	27.1

Table 1.2 presents data from various sources on health status and health infrastructure for Qatar. In 1998 mothers received at least five antenatal checkups in 52.6% of births. Tetanus toxoid coverage is 100% for Qatari children. Also the table shows that 100% of births were delivered in medical institutions and assisted by health professionals. The table shows that the under 5 mortality rate in 2006 was 10.5 deaths per 1000 live births. Diabetes prevalence is estimated to be as high as 15% of the population, with more than 40% of adults reporting cigarette smoking in the past month, based on Qatar's 2007 WHO Global Youth Tobacco Survey [†].

[*] NHA–Qatar Statistical Authority http://www.planning.gov.qa/arabic/ppc/eng/studies.htm
[†] *WHO/CDC Global Youth Tobacco Survey (GYTS)* http://www.who.int/tobacco/surveillance/gyts/en/index.htm

Table 1.2 Qatar health status and health infrastructure indicators, 2006

Indicator	
Percentage of births for which the mother received at least five antenatal checkups (1998)	52.6%
Percentage of children who received 2 doses of tetanus toxoid vaccine (2006)	100%
Percentage of births delivered in medical institutions (2006)	100%
Percentage of deliveries assisted by health professionals (2006)	100%
Percentage of children fully immunized (2006)	97.2%
Crude birth rate per 1000 population (2006)	16.9
Under 5 mortality rate per 1000 live births (2005)	10.5
Infant mortality rate per 1000 live births (2005)	8.2
Life expectancy at birth (2005)	75.5 years

1.3 Sociodemographic profile

Table 1.3 presents the sociodemographic profile of Qatar (Annex 1). The data were collected from the Qatar annual health report of 2006 and the 2004 census*. The population of Qatar was estimated to be 838 065 in 2006. In 2007, the population estimate was 1 036 000, based on the 2006 WHS. However, the Ministry of Interior records tracking migration indicate that the Qatar population was estimated to be 1.5 million in April 2008 due to the influx of non-Qatari labourers.

The annual growth rate of population in Qatar was 3.5% during 1990–2006. The sex ratio for the country is 200.4 females per 1000 males. According to the 2004 census, Qatar has a literacy rate of 89%. The crude birth rate and the crude death rate for Qatar is about 16.86 and 2.09 for 2006 with total fertility rate of 2.67. (Table 1.3).

Table 1.3 Selected sociodemographic indicators for Qatar

Population (estimated 2006)	838 065
Annual population growth rate (%) 1990–2006	3.5
Sex ratio (females per 1000 males)	200.4
Literacy rate (%; 2004 census) total	89.0
male	89.1
female	88.6
Crude birth rate per 1000 population (2006)	16.9
Crude death rate per 1000 population (2006)	2.1
Total fertility rate per 1000 women aged 15-49 (2006)	2.7
Percentage of the population aged under 15	22.5
Percentage of the population aged over 65	1.2

* Ministry of Public Health annual report, 2006; Qatar census, 2004

1.4 Health-related surveys in Qatar

In Qatar, census and vital statistics (births and deaths) registration and hospital registration systems provide reliable data on socioeconomic and demographic aspects of the population. However very little information is available on the health of the population, particularly on morbidity indicators. In view of the then lack of routine morbidity information, the Ministry of Public Health conducted the Gulf Family Health Survey Program (GFHS) Qatar Family Health Survey (QFHS-98) in 1998. It is a nationally representative household sample survey. The sample of the QFHS-98 was a probability design based on the 1997 population census as the sample frame. The target population consisted of Qatari households and successfully covered completed 4207 households.

The objectives of Qatar Family Health Survey were to provide information on the general health of the adult population, reproductive health and child health. It constructed groups of indicators related to general health of the adult population, aged 15 years and over. The selected indicators covered general aspects of physical health, acute illnesses, care-seeking behaviour and smoking. Findings from this survey are shown in the following table (Table 1.4).

Table 1.4. Percentage of adults in Qatar with selected illnesses, 1998

Condition	Percentage
High blood pressure	6.5
Heart disease	2.2
Myocardial infarction	0.6
Diabetes	7.9
Joint disease	6.3
Gastric ulcer	2.8
Kidney disease	2.2
Persistent headache	4.6

Source: Qatar Family Health Survey

2. Methodology

2.1 Introduction

The purpose of this chapter is to explain important features of the World Health Survey (WHS), including the sample design, the questionnaire and major aspects of the analysis of the survey. This includes the training procedure for interviewers and the data entry process for the completed questionnaires.

The World Health Survey series was developed by the World Health Organization (WHO) as a means to compile comprehensive baseline information on the health of populations in different countries. Due to the standardized questionnaire, this information is also comparable between countries, and currently the WHS has been implemented in more than 70 countries. The survey was designed on a modular basis, with the intention of providing low-cost information that supplements data from national health information systems in order to build up an evidence base for policy-makers. The motivation for conducting the WHS in Qatar is to obtain good quality data and evidence that will form the basis of health reform in the country. As noted in Chapter 1, Qatar's health system has undergone large reforms since 2006. The results from the WHS conducted in 2006 are therefore useful in two respects: as an indicator of the health of the Qatari population and as a baseline against which the changes to the health system can be assessed.

The survey was implemented by a team of experts from Qatar, drawn from the fields of public health, epidemiology and statistics. The implementation was assisted by a technical team from WHO. There were two main stakeholders in the Qatar WHS programme. These were the National Health Authority of Qatar (see Chapter 1 for a description of the functions of the NHA) and the Planning Council of Qatar. The Planning Council is the main Qatari state organization concerned with preparing economic and social policies and plans. One of its main goals is to facilitate cooperation with other GCC countries to encourage economic and social integration. Within the health context, the Planning Council aims to:

- provide valid, reliable and comparable information to supplement the information provided by routine health information systems
- build the evidence base necessary for policy-makers to monitor if health systems are achieving the desired goals, and to assess if additional investment in health is achieving the desired outcomes
- provide policy-makers with the evidence they need to adjust their policies, strategies and programmes as necessary.

The NHA and the Planning Council of Qatar worked in close collaboration during the implementation of the WHS to ensure that the results obtained from the survey are of the highest quality and are of most use to policy-makers.

2.2 Sample design

The Qatar WHS is a nationally representative survey. To ensure that the sample of households and individuals who are interviewed are representative of all Qatar a detailed sample design was implemented. The sampling design took into account the WHO requirements specified in the World Health Survey sampling guidelines for participating countries.

2.2.1 Background information to the sample design

The population of Qatar can be divided into different groups. There are both Qatari and non-Qatari residents and these residents either live in a household or live collectively—Qatar has a high proportion of non-Qatari residents (about 75%), many of them living in camps or in special housing quarters. The latest population census was conducted in 2004. This indicated that there were 744 029 persons in Qatar. This consisted of 27 403 Qatari households and 53 733 non-Qatari households. Furthermore, there were 28 022 mostly non-Qatari collective households. Of these collective households, 8214 contained more than 7 persons.

To facilitate the sample design the population of Qatar was divided into the following groups and subgroups:

1. Non-institutional population
   - 1.1 Qatari households:
     - with no non-Qatari members
     - with one or more non-Qatari members
   - 1.2 Non-Qatari population:
     - living in regular households.
2. Institutional population:
   - 2.1 Qatari and non-Qatari population living in collective households (e.g., army barracks, hospitals, dormitories, prisons)
   - 2.2 Non-Qatari population
     - living in collective households with 7 or fewer members
     - living in collective households with more than 7 members.

The World Health Survey in Qatar included both the institutional and non-institutional population. However, this report only includes the results of the non-institutional population and excludes those living in a collective household. A separate report will be produced discussing the health of the institutional population. Therefore the scope of this report is only the Qatari households and the non-Qatari population living in regular households. The sample design reported below refers only to the sample design for these households.

2.2.2 Sample design

The WHS sampling guidelines recommend a target sample size of 5000 individuals. As will be explained later, one person is selected from each of the sampled households to respond to the questionnaire. As a result, the target sample size for households is also 5000.

The non-response rate for previous surveys conducted in Qatar has been relatively small, about 5%–10%, which is similar to the rate seen in surveys conducted in countries with a similar level of development to Qatar. The WHS, however, involves the completion of a long and complex questionnaire, coupled with a number of physical measurements with specialized instruments. Therefore a substantially higher rate of non-response in the Qatari WHS was expected. Thus, assuming a non-response rate of 20%, the required sample size to reach the target 5000 households was calculated to be 6250 households.

The target sample size of 6250 was divided equally between Qatari and non-Qatari households. As there are fewer Qatari households the result of this equal division means that a higher proportion of Qatari households are covered in the survey. The reason for this was to achieve greater precision for the estimates produced from the survey for the Qatari population. A further reason is due to the wider age and socioeconomic distribution of the Qatari population than the non-Qatari population. To ensure that all parts of the Qatari population are represented in the survey a larger sample proportion is required. Table 2.1 summarizes the sample size calculations for the Qatar World Health Survey.

2.2.3 Sampling frame

There is no adequate population register in Qatar that can be used as the sampling frame. Therefore the frame was constructed using the administrative organization of Qatar.

Qatar is divided into ten municipalities, which are divided into one or more zones. These zones are further divided into blocks, which form the smallest administrative unit in the country. There are currently 98 zones and 2392 blocks, although not all of the zones and blocks are currently inhabited. The average number of persons living in each block is about 300, although there is large variation in this number. Furthermore, Qatari and non-Qatari households are mainly in separate geographical locations, necessitating two different listings of the blocks.

To obtain the sampling frame, separately for Qatari and non-Qatari households, the country was divided into primary sampling units (PSUs). This was done twice (for the different types of household), and therefore some of the PSUs overlap. Contiguous administrative blocks were combined so that each PSU contained at least 56 households of the required type. PSUs were designed to not cut across zones, unless this was impossible, and no PSUs crossed municipality boundaries. The PSUs were constructed using information from the 2004 population census. From each PSU 21 households were selected into the final sample. The Qatari sampling frame consisted of 394 PSUs, while the non-Qatari frame consists of 665 PSUs.

The number of PSUs which were required to be sampled to achieve the target sample size of 3150 (for both Qatari and non-Qatari households), given a sample size within each PSU of 21, was 149. This was rounded to 150. These were selected using systematic random sampling, with probability proportional to the size of the PSUs, after sorting the sampling frames by municipality and zone. Four PSUs were selected with probability 1 (two for the Qatari frame and two for the non-Qatari frame) to ensure that each of the 10 municipalities in Qatar was represented in the final survey.

Table 2.1 Sample size calculations for the Qatari World Health Survey

	Sample size	Assumed non-response rate	Target sample size		Sample fraction
	**Households**		**Households**	**Persons**	**Households**
Qatari	3125	20%	2500	2500	9.1%
Non-Qatari	3125	20%	2500	2500	4.6%
Total	6250	20%	2500	2500	6.2%

After selecting the sample PSUs from each of the two frames, the households within these PSUs were enumerated. This was to ensure that all households in that PSU were included in the final sampling frame, especially those that had been built since the 2004 population census. After this enumeration procedure has been completed the final sample was obtained. Various random sampling methods were implemented to obtain the final sample of 21 households from each of the PSUs.

2.2.4 Final sample information and non-response

After conducting the sampling process described above, the actual sample size was 6098 households. This was broken down into 3065 Qatari households and 3033 non-Qatari households.

It was seen that out of these selected households, 238 (7.8%) of Qatari and 500 (16.5%) of non-Qatari households were impossible to interview. The main reasons why this was the case was that the households were either empty blocks, there was a major language barrier which proved insurmountable or there was difficulty in accessing the respondents. Furthermore, 362 (11.7%) of the Qatari and 172 (5.7%) of the non-Qatari households refused to participate in the survey. Therefore the overall non-response rate (excluding household which were impossible to interview) was 522 (8.7%). There were also a number of households that did not complete the interview once it had started. For Qatari households the number was 44 (1.4%), while for non-Qatari households there were 9 (0.3%) that did not complete the questionnaires.

Overall, the response rate achieved was 79.5% for Qatari households and 77.6% for non-Qatari households, with an overall response rate of 78.3%. This means that 2423 Qatari households and 2352 non-Qatari were interviewed, with an overall sample size of 4775 households (Table 2.2).

Table 2.2 Respondent households by their nationality and municipality

	Nationality					
	Qatari		Non Qatari		Total	
**Municipality**	***N***	**%**	***N***	**%**	***N***	**%**
Doha	*889*	35.9	*1519*	64.6	*2388*	50.1
Al Rayyan	*1021*	42.1	*504*	21.4	*1523*	31.9
Al Wakra	*141*	5.8	*115*	4.9	*256*	5.4
Umm Salal	*221*	9.1	*32*	1.4	*253*	5.3
Al Khor	*78*	3.2	*49*	2.1	*127*	2.7
Al Shamal	*37*	1.5	*19*	0.8	*56*	1.2
Al Ghuwariya	*14*	0.6	*16*	0.7	*30*	0.6
Al Jumaliya	*20*	0.8	*42*	1.8	*62*	1.3
Jaryan Al Batna	*20*	0.8	*17*	0.7	*37*	0.8
Mesaieed	*2*	0.1	*39*	1.7	*41*	0.9
Total	*2423*	100	*2352*	100	*4775*	100

2.2.5 Sample weights

The differential sizes of each of the PSUs used in the selection of the final sample of households means that each of the households in Qatar did not have an equal probability of being selected into the sample. Analysing the survey without taking into account this fact will produce results that are not representative of the population. Therefore during the analysis the data were weighted to account for these differential selection probabilities. These weights are calculated from the sizes of the different PSUs and to account for households that did not respond to the survey. The sampling weights were applied at each stage of the analysis. After weighting the counts of respondents in each category may not be a round number. If this was the case the count was rounded to the nearest whole number. Therefore in some tables the total number of respondents may differ from the sum of the respondents in each of the categories.

2.3 Questionnaire design

The Qatar World Health Survey uses a common survey instrument, developed by WHO, with separate modules for various components. The modules cover key aspects of health system outcomes, inputs to that system and aspects of the way systems function. The different modules cover:

- the health states of the population: measuring health in multiple domains
- risk factors and their association with health states: measuring various risk factors such as tobacco, nutrition and physical activity levels
- the responsiveness of health systems: whether a health system meets the legitimate expectations of the population
- coverage, access and use of key health services such as immunization, treatment of childhood illness, safe motherhood interventions, essential treatments, mental health interventions, etc.
- health care expenditures: how much households contribute to the health system.

The questionnaire is divided into two sections, with a household and an individual questionnaire. Both of these are face-to-face questionnaires, and the standard instruments provided by the WHO have been modified slightly to suit the Qatari context.

2.3.1 Household questionnaire

The aim of the household questionnaire is to collect information that is common to all those who live in the same household. To answer the household questionnaire one person, a key informant, was interviewed from each of the sampled households.

This questionnaire first collected information regarding all the residents in the selected household, including their ages, education, marital status and whether any person had a long-standing illness or disability. Further questions on the household questionnaire related to information about the facilities in the household, expenditure on health and other items, monetary and in-kind transfers in and out of the household, assets owned and the income of the household. A further section obtained information about the birth history of each of the women aged 15 to 49 in the household, while the final section collected information about deaths in the household.

2.3.2 Individual questionnaire

One person aged over 18 from each of the sampled households was selected to answer the individual questionnaire. This individual did not have to be the same person as answered the household questionnaire. The household questionnaire recorded each of the members of the household. Kish grid tables were used to randomly select one person from the list of eligible men and women to answer the individual questionnaire.

The individual questionnaire consists of a number of different modules. The modules selected for use in Qatar are as follows.

1. Sociodemographic characteristics: This section includes questions on the mother tongue of the respondent, the date of birth, education, religion and marital status.
2. Work history and benefits: Information regarding the working history of the individual was collected, including benefits received from work, reasons for not working and occupation.
3. Health state description: self-ratings of health on a number of different domains, such as mobility, self-care and pain were included here. Also in this section was the WHODAS 12-item functioning assessment and the vignettes for health. These will be explained in further sections below.
4. Anthropometric and blood tests: the respondents were asked to have their height, weight, blood pressure and various blood chemicals measured.
5. Risk factors and preventative health behaviour: this module contained questions related to risk factors such as consumption of tobacco, nutrition and physical activity including both vigorous and moderate activity.
6. Chronic conditions and health services coverage: this section included questions on arthritis, angina, asthma, depression, depression and diabetes and asks sufferers whether they are taking drugs for these conditions. Also asked were questions on injuries, oral health and vision. Three further subsections were asked in this module, including questions on cervical and breast cancer screening, child vaccinations and illness, and reproductive and sexual health.
7. Health care use: use of the health system was investigated in this module, including an assessment of the responsiveness of the system. The module covered areas such as the importance of health care, seeing health care providers, outpatient care and care at home and inpatient hospital care.
8. Social capital: general opinions on life, interaction with the community and social trust were investigated in this section. A small number of questions were also asked about crime.
9. Subjective well-being and quality of life: The final section asked about the quality of life of the respondent, and included the WHOQol-8 instrument for assessing this.

All the modules used in both household and individual questionnaires were checked by both local and international experts in collaboration with WHO. The instrument was translated into Arabic and adapted to suit the culture in Qatar. The questionnaires were then tested for cultural applicability and sensitivity through word and pilot testing of the questionnaires.

2.3.3 Vignettes

An important aspect of the World Health Survey series is the inclusion of vignettes. These were included in both the health care use and the health care description modules. They were developed to enable within- and between-population comparisons to be conducted. The rationale behind the vignettes is that different subgroups of the population may have different ideas about what constitutes “good” health. For example, a younger person may feel that the inability to run a short distance means that their mobility is not good, but slightly impaired. However, an older adult may not consider that this is important, and even though they cannot run a short distance that their health is still good. The vignettes within the questionnaire are designed to ensure that an individuals’ responses on their health state or on health system responsiveness can be made comparable across both subgroups within countries and across counties.

Vignettes are hypothetical stories about peoples’ health condition and their experience with health care systems. In vignettes, the respondents are asked to rate the condition and experience of the person in the story as if it was respondents’ own experience. This rating is used to calibrate respondents’ self-reports on their health state. A similar procedure is used to rate an individuals experience of the health system.

The responses to the vignettes are on a scale ranging from “very good” to “very bad”. It is assumed that the differences between each of the ratings is similar, and hence the difference between “very good” and “good” is the same as the difference between “good” and “moderate”. To obtain a figure for health or responsiveness which is comparable within the population a statistical method, called the CHOPIT model, is applied to the responses. This analyses the distribution of the vignette responses and compares it to the reports for the respondent’s health state.

The results of the health state vignettes will be discussed further in the relevant chapters. However, it was seen that the responses to the vignettes were of low data quality, with many respondents rating each of the vignette experiences as “very good”, even when it was clear that the health state of the vignette is very poor. It was decided to not analyse the vignettes for this report, and further analysis is required to assess if the data obtained from the vignettes can be used at all. The vignettes for the health system responsiveness were used in the analysis, and will be discussed in Chapter 7.

2.4 Training of the data collectors

A series of training events was conducted to ensure that the collection of data was conducted to the highest possible standards.

First, a training course lasting a week was conducted to train the trainers in preparation for conducting the training of the local interviewers. This was a workshop organized by WHO experts, and was carried out in Qatar from 25 to 30 March 2006. This discussed the research methodology and data collection tools used in the survey. Attendees included the Qatar WHS team and also WHS teams from other gulf states and provided a forum for the exchange of experiences and ideas between countries of similar background.

Two days after the completion of the train-the-trainers workshop, the Qatar WHS team commenced training of local interviewers and supervisors. Trainees had a total of 56 hours extensive training, comprising 8 hours training per day over 7 days, from 1 to 8 April 2006. These daily sessions involved 6 hours of training at the workshop, and an additional 2 hours home assignment each evening. After the opening session, interviewers were divided into four groups, three of which consisted of Arabic-speaking

interviewers and one of English-speaking interviewers. During practical sessions, these groups were further divided into smaller working groups to facilitate interaction. Due to cultural considerations, more female interviewers were recruited than males. It was decided that male interviewers would be used in settings where prior permission had been obtained from families of respondents. A decision was made to allow male supervisors to also work as interviewers in settings where male interviewers were needed. In addition to the basic training given to all interviewers, supervisors were given specialist training sessions. It was decided that based on performance in the post-training test, more male interviewers would be recruited for supervision and call-back interviews in order to have adequate numbers of supervisors of both sexes.

The training programme covered a number of aspects, including administrative issues, fieldwork planning, a review of all materials, contact procedures, consent and confidentiality and also how to conduct an interview. Further session were held on the interview procedures in the field, supervision in the field and reporting procedures, and the general structure of survey team and role of all members of the team.

Role-play was conducted during training sessions and as homework assignments. As attested to by interviewers, this contributed greatly to improving their command of the questionnaires. To add value to the training, the first two questionnaires administered in the field by each interviewer were regarded as part of the training. These questionnaires were reviewed and feed back was given. Quality assurance was a cross-cutting issue in all the training sessions, and quality was stressed by all the trainers. Supervisors and interviewers were evaluated continuously before, during, and after the training programme. This evaluation process continued during field work.

Of the 195 field workers trained, 187 had at least a university degree, with the remaining eight having completed secondary school. Most of these field workers were relatively young and were working either for the National Health Authority or the Planning Council. The majority also had previous research experience from the Qatar population census and other surveys. The ratio of male to female interviewers was about one to two, in order to accommodate cultural norms.

2.5 Quality assurance

Throughout the survey implementation quality assurance procedures were conducted to ensure that accurate and reliable data were obtained. This assurance was undertaken by an external international expert in the field. Using the WHO quality assurance standards and guidelines (procedures for quality assurance implementation by country survey teams and quality assurance advisors) that were provided by WHO upon my request, the following activities were carried out.

- Desk review and content analysis: the evaluation reviewed all implemented, ongoing activities and outputs achieved by the survey team according to the work plans, progress reports, survey products and survey documentation.
- In-depth interviews: key contacts, including the survey staff, and other partners associated with the survey were interviewed to assess the progress of the survey.
- Participant observations: through field visits to selected survey sites and the performance of the interviewers in collecting the data were assessed.

2.6 Ethical considerations

Throughout the implementation of the survey, all ethical procedures were followed. This included at the design, training and implementation stages. All the participants

were assured that the information provided would be confidential and would not be used for any reason apart from scientific purposes. It was stressed to the participants that they had the right to refuse participation and to withdraw from participation at any time.

2.7 Data analysis

Once the questionnaire had been completed the responses were entered into a spreadsheet for analysis. Before the analysis occurred the data were cleaned, and impossible or implausible values were studied and removed from the analysis if appropriate. After this process was finished for all questions the responses were then analysed.

The analysis conducted for this report was simple tabulations of the data against important subcategories. These categories were, in general, sex, nationality (Qatari or non-Qatari), region, age and wealth. Three different regions were used in the analysis: the two largest municipalities of Doha and Al Rayyan and the rest of Qatar. Age was also grouped into categories, which differed depending on the analysis conducted. The wealth variable is explained further below. At all stages sampling weights were used to ensure the results obtained were representative of the population of Qatar.

2.7.1 Wealth quintiles

In many countries it is known that wealth is highly related to health. The measurement of wealth is not simple, and a number of different methods have been used in different reports to capture the wealth of a household. For the Qatar WHS it was hoped to use income of the main earner in the household as a proxy for wealth. However it was seen that many households did not answer this question, and it could therefore not be used.

A different method is to use the assets that the household owns as an indicator of wealth. The households reported ownership of 20 different items, including televisions, microwaves, computers, cars, mobile phones and yachts. The number of each of these 20 items was also recorded. The majority of households reported this information.

A statistical technique called principal components analysis was applied to this asset data, taking into account the number of items per household member. The first factor that is produced using this method is taken as representing wealth. This factor is divided into five different groups using the sampling weights and the number of household members, and these are termed as wealth quintiles. Therefore the wealth quintile variable has the values of 1 to 5, with 1 being the poorest households and 5 the richest. The analysis comparing the bottom quintile to the top quintile within each data set will be reflecting those in relative poverty.

2.8 Limitations of the survey

The main limitation of the Qatar WHS was that the information collected was a self-report from the individuals interviewed. A number of responses, including such items as morbidity and health state valuation are impossible to verify. The vignettes are intended to solve some of these problems, although it has already been seen that the responses to the vignettes were of poor quality and therefore could be used to adjust differential responses between groups.

During the analysis it was noted that the data on mortality did not mirror the mortality information contained in the Qatari vital statistics. Information was is collected on both births and deaths in Qatar with a high degree of accuracy. The household questionnaire in the WHS collected information about deaths in the household and also about deaths

of siblings of household members. The results of these sections were deemed to be less accurate than the vital statistics information, and hence the results are not reported in this report.

3. Profile of the household population and individual respondents

3.1 Introduction

The Qatari World Health Survey collected information on the sociodemographic profile of all those who lived in the selected households. Individual respondents were randomly selected from all adults aged over 18 currently listed as living in the households, with one individual selected from each household. Further information was obtained about sociodemographic details and working history from these individuals. This chapter presents the results for the sociodemographic profile of both the members of the households and the selected individuals, coupled with a detailed study of the work history of the individual respondents.

3.2 Household population profile

This section provides a summary of the sociodemographic information provided in the household questionnaire, concentrating on the age profile, sex, marital status and educational achievement. The household questionnaire was administered to a key informant within the household who answered on behalf of all members of that household. Overall, 4779 households were interviewed, covering a population of 29 339 inhabitants. It was seen that 48.5% of the household population was male, and 39.5% were Qatari nationals.

Analysis of certain questions was only conducted for certain age groups. The age and sex analysis covers the population of all ages, while marital status is summarized for those aged 15 and above. Educational status statistics cover the population aged 10 and above.

3.2.1 Age and sex distribution of the household population

A summary of the age and sex distribution of the household population is shown in Table 3.1. This is presented for the total population and also broken down by nationality, either Qatari or non-Qatari, and by sex.

Table 3.1. Percentage distribution of the household population by selected characteristics

		Sex		Nationality			
		Male	Female	Qatari	Non-Qatari	Overall	*N*
**Sex**							
	Male	-	-	49.9	47.5	48.5	*14219*
	Female	-	-	50.1	52.5	51.5	*15119*
**Nationality status**							
	Qatari	40.7	38.4	-	-	39.5	*11583*
	Non-Qatari	59.3	61.6	-	-	60.5	*17756*
**Age**							
	0 to 14	35.4	31.9	39.6	29.6	33.6	*9847*
	15 to 29	24.6	28.0	28.9	24.8	26.4	*7744*
	30 to 59	36.3	37.9	27.1	43.7	37.1	*10890*
	60 or over	3.7	2.2	4.4	1.9	2.9	*858*
**Region**							
	Doha	46.2	45.8	30.4	56.2	46.0	*13498*
	Al Rayyan	37.4	38.3	49.1	30.5	37.9	*11104*
	Other regions	16.4	15.9	20.5	13.3	16.1	*4736*
**Marital Status**							
	Never Married	34.0	29.6	40.6	26.7	31.7	*6177*
	Currently Married	64.9	64.6	53.5	71.0	64.7	*12619*
	Separated	0.1	0.2	0.0	0.2	0.1	*28*
	Divorced	0.5	2.1	2.3	0.8	1.4	*267*
	Widowed	0.5	3.5	3.6	1.2	2.1	*401*
**Education**							
	Less than primary school	10.4	10.8	12.7	9.3	10.6	*2393*
	Primary school completed	16.2	13.7	18.1	13.0	14.9	*3364*
	Secondary school completed	17.0	14.1	20.1	12.7	15.5	*3498*
	High school completed	15.4	16.4	18.5	14.4	15.9	*3595*
	College/pre-university completed	10.4	8.3	3.6	12.7	9.3	*2096*
	University completed	18.6	16.9	17.0	18.2	17.7	*4006*
	Post graduate degree completed	2.7	1.1	1.3	2.2	1.8	*418*
	Informal education; can read and write	6.6	11.1	1.9	13.1	8.9	*2011*
	Illiterate	2.8	7.6	6.7	4.4	5.3	*1197*
**Household size**							
	1 to 5	31.2	28.2	6.5	44.7	29.7	*8699*
	6 to 10	40.0	42.1	46.0	37.9	41.1	*12048*
	11 to 15	18.5	19.3	29.7	11.9	18.9	*5554*
	Over 15	10.2	10.5	17.8	5.5	10.4	*3038*
**Number of respondents**		*14219*	*15119*	*11583*	*17756*		*29339*

The results show that just over a third of the residents in the surveyed households were under 15 years of age, while only 2.9% were over 60 years old. There were higher percentages of Qatari nationals in these two age groups compared with non-Qatari nationals, while there were a higher percentage of non-Qataris in the 30 to 59 age group. The population pyramid shown in Figure 3.1 shows the age and sex distribution of the household population, broken down by nationality status. It is clear that there is an excess of non-Qatari females and, to a lesser extent, males in the 25–44 age groups. The pyramid indicates that fertility of Qataris has fallen in the last 10 to 15 years, although the fertility rate of non-Qataris is still relatively high.

Figure 3.1. Population pyramid for household residents, by nationality

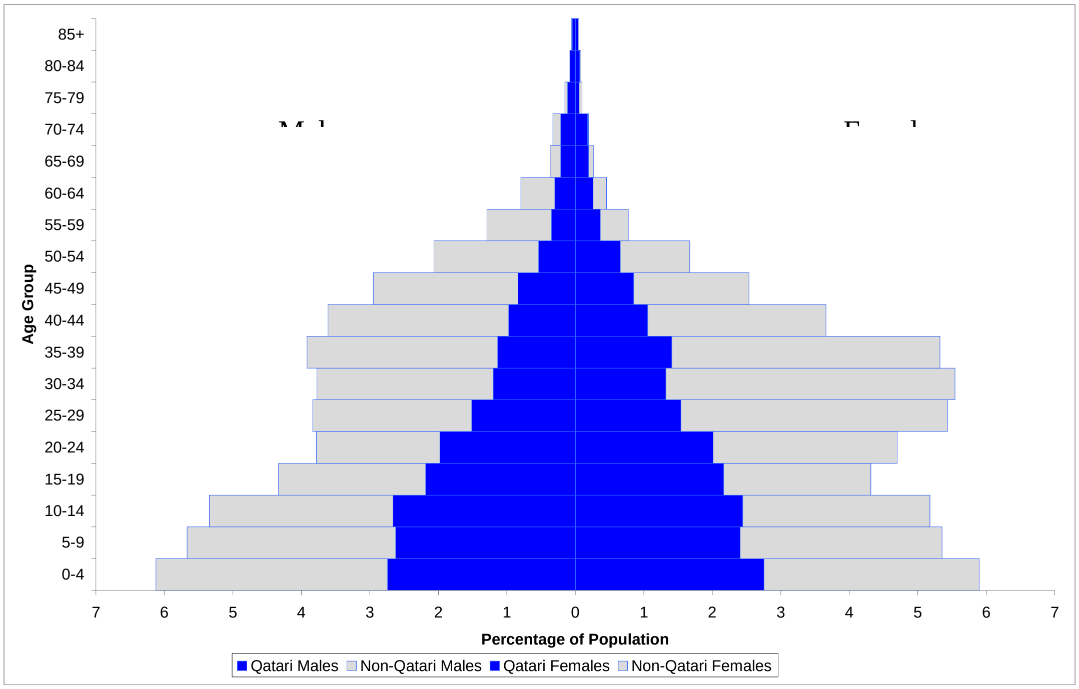

3.2.2 Marital status

The marital status of the household residents aged over 15 was collected, and the results are summarized in Table 3.1. Almost two-thirds of the population (64.7%) were then married, while 31.7% had never been married. There are very few people who were separated from their partners (0.1%), divorced (1.4%) or widowed (2.1%).

A higher proportion of males compared with females had never married, while females were more likely to be widowed or separated than males. Figure 3.2 shows the population pyramid for all household residents, broken down by marital status. Ever-married residents were those who were then married, divorced, separated or widowed. The pyramid indicates that females married at an earlier age than males, with a higher proportion married at each age category until the 40–44 age group.

Figure 3.2. Population pyramid for all household residents, by marital status

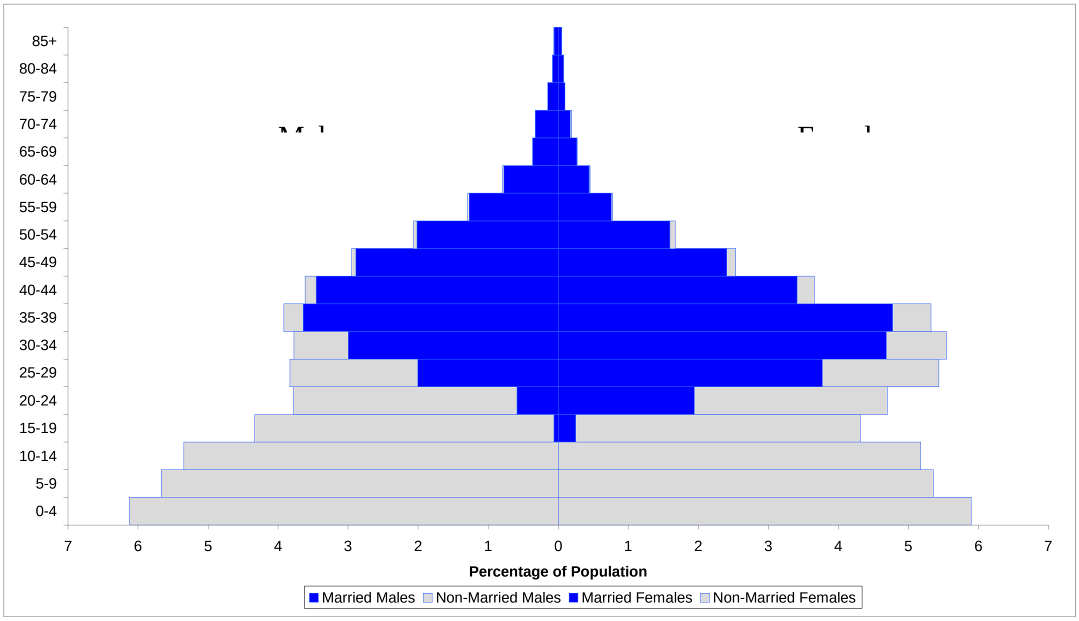

Differences in marital status were also observed by nationality. Non-Qataris were more likely to be currently married, with 71% in this category, compared with only 53.5% of Qataris. The percentage of never-married household members is higher for Qataris though, with 40.6% in this group, compared with 26.7% of non-Qatari nationals. A higher percentage of Qataris were also widowed or separated.

Figures 3.3 and 3.4 show the population pyramid by marital status for Qataris and non-Qataris separately. Figure 3.3, for Qataris, highlights the earlier marriage of females than males, and also the reduced fertility for the younger ages (indicated by the similar sized bars in the age groups 0 to 14). The non-Qatari pyramid shows the great excess of females in the 20 to 44 age groups, compared to the males. These pyramids also show the overall household population profile of the Qataris and non-Qataris, which can be seen by studying the overall shape of the pyramid. This confirms the discussion from section 3.1.1 regarding the high fertility of the Qatari population, with some evidence of a fall in fertility in the last decade. The non-Qatari pyramid shows clearly the large excess numbers of both males and females in the 25 to 44 age groups.

Figure 3.3. Population pyramid for Qatari household residents, by marital status

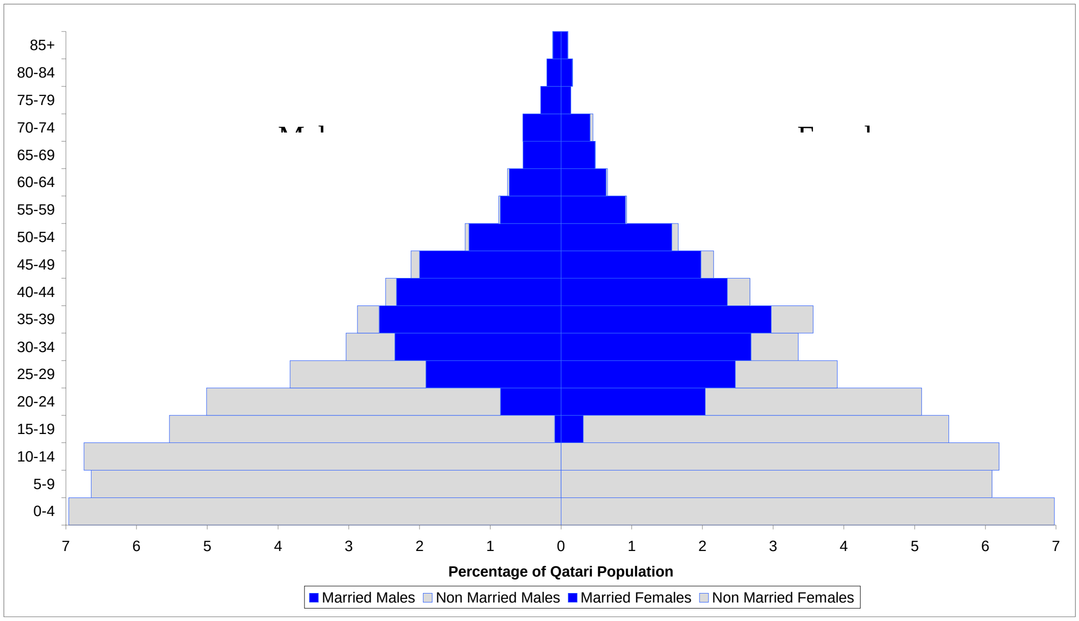

Figure 3.4. Population pyramid for non-Qatari household residents, by marital status

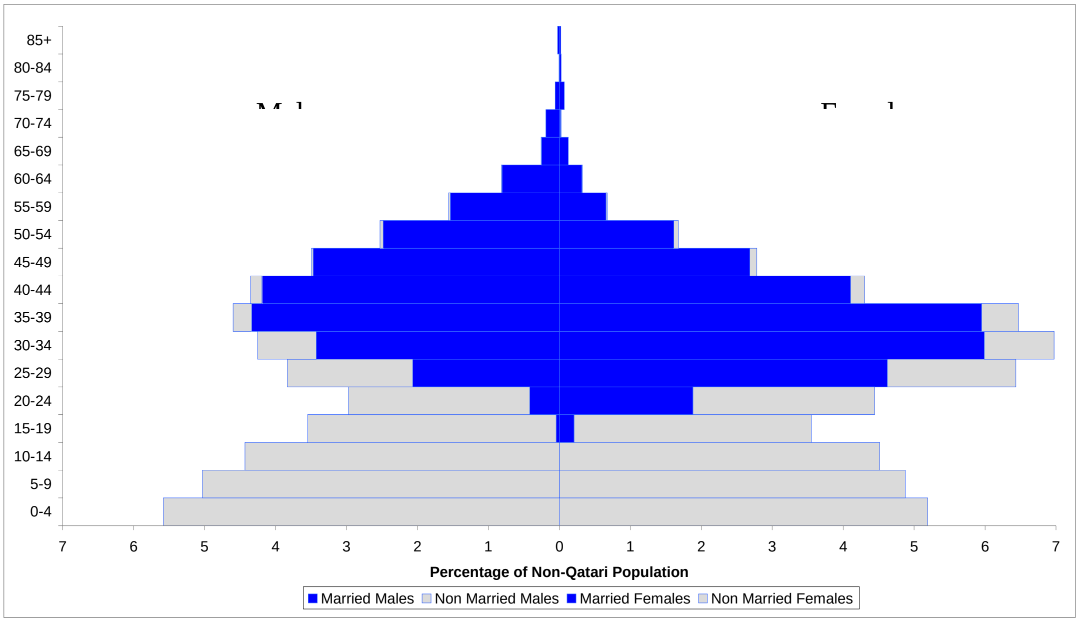

3.2.3 Educational status

The distribution of educational level across the household population in Qatar for those aged 10 and above is also shown in Table 3.1. There are nine categories for educational level. Seven relate to formal schooling, with the level ranging from less than primary school to postgraduate degree completed. There are also two categories for those with informal schooling. These categories report the literacy of the household member: either the member is able to read and write or they are illiterate.

It is important to note that the results for education, displayed in Table 3.1, are not adjusted for age. The difference in the age profiles of Qatari and non-Qatari household members indicate that there are a higher percentage of Qataris who will not have completed their education at the time of the survey (i.e. they are aged over 10 but are still attending school). Therefore lower levels of education shown in the table for Qataris may reflect the difference in the age profiles rather than an actual difference in educational level.

Overall, 44.9% of the household members completed high school (this includes those who went on to university). The percentage of those without any formal schooling is 14.2%, with almost two-thirds of these individuals being able to read and write. Females are more likely to have had informal schooling, while a greater percentage of males have achieved a higher level of education, albeit by only a small amount. Non-Qatari nationals have higher educational achievements than Qataris, with a larger percentage having completed college, university and post-graduate degrees, while more Qataris have low levels of formal education. Informal education was mainly stated as being used by non-Qataris, with 17.5%, compared to Qataris, where only 8.6% had this type of education. However, the majority of non-Qataris with informal education are literate, while the majority of Qataris with the same kind of education are illiterate.

3.2.4 Household size

Household size varies widely in the survey. Household members are most likely to live in a household with between six and ten people as co-residents. About 29.7% of individuals live in a household with five or fewer residents, while about 10% live in households with more than 15 residents.

Smaller households are more a feature of non-Qataris, with 44.7% of non-Qatari individuals living in a household with fewer than five inhabitants, compared with 6.5% of Qataris. Large household sizes are mainly Qataris, with 29.7% of individuals living in a household of size 11 to 15, and 17.8% living in a household with more than 15 inhabitants.

3.3 Sociodemographic profile of individual questionnaire respondents

This section provides the characteristics of the respondents who answered the individual questionnaire. In every household one person aged 18 or over was selected at random to participate in a more detailed individual questionnaire. The characteristics of those who responded in this section are presented here. Information was collected from 4779 people. Table 3.2 shows the characteristics of the individuals broken down by sex, age, nationality, region and education, along with information about their mother tongue. These responses will be discussed in the sections below.

3.3.1 Age and sex distribution and other sociodemographic indicators

Of the respondents to the individual questionnaire, 50.6% were male. The highest proportion of responses was obtained for the 30–39 years age group, which had 35.3%

of the respondents. Fewer than 3% of the respondents were in the 60–69 and over 70 age groups. Comparing this to the distribution of household residents ages, seen in the population pyramids, there are lower percentages of the young (under 30) and old (over 60) who were sampled to answer the individual questionnaire. In the full household population, 29.2% of household members were 20–29 years old, while only 23% of the individuals were in the wider age range of 18–29 in the individual questionnaire. These deficits were balanced by a larger proportion of individuals in the 30–59 age groups compared to the household age distribution.

Table 3.2 Percentage distribution of selected background characteristics of respondents to the individual questionnaire

	Sex			
	**Male**	**Female**	**Overall**	***N***
**Age**				
18 to 29	18.9	27.3	23.0	*1101*
30 to 39	29.8	41.0	35.3	*1687*
40 to 49	31.2	21.6	26.5	*1265*
50 to 59	16.1	7.4	11.8	*564*
60 to 69	3.1	2.2	2.7	*127*
70 or over	1.0	0.5	0.7	*35*
**Nationality status**				
Qatari	27.6	40.1	33.8	*1615*
Non-Qatari	72.4	59.9	66.2	*3164*
**Region**				
Doha	56.6	52.1	54.4	*2600*
Al Rayyan	28.6	33.1	30.8	*1471*
Other regions	14.8	14.8	14.8	*707*
**Marital status**				
Never	15.6	11.8	13.7	*655*
Currently	82.9	81.8	82.4	*3936*
Divorced	0.4	0.4	0.4	*20*
Widowed	0.7	2.7	1.7	*79*
Separated	0.4	3.3	1.9	*89*
**Education**				
Less than primary school	3.1	3.3	3.2	*153*
Primary school completed	7.1	5.6	6.4	*305*
Secondary school completed	11.0	10.4	10.7	*510*
High school completed	16.3	21.1	18.7	*891*
College/pre-university completed	19.3	17.9	18.6	*889*
University completed	35.3	31.9	33.6	*1605*
Post graduate degree completed	4.9	2.6	3.7	*179*
Missing	3.2	7.3	5.2	*248*
**Mother tongue**				
Arabic	63.3	72.8	68.0	*3250*
English	5.9	5.1	5.5	*264*
Urdu	8.3	5.8	7.0	*335*
Malayalam	7.7	5.7	6.7	*322*
Farsi	1.2	0.8	1.0	*47*
Bengali	1.5	0.6	1.1	*51*
Other	12.2	9.1	10.7	*510*
**Number of respondents**	**2418**	**2361**	**4779**	

About two-thirds of the respondents stated that they were non-Qatari. There was not an even distribution between males and females regarding nationality status. Non-Qataris made up 72.4% of males, but only 59.9% of females. The age distribution by nationality status and sex differed as well, as can be seen in Figure 3.5. For males, there was an excess of non-Qataris compared with Qataris in the 40–59 age groups. For females, there was a larger proportion of non-Qataris in the 25–39 age groups than Qataris. In the older (above 60 years) and younger (below 25 years) there was a higher percentage of Qataris.

Figure 3.5. Age distribution of individual respondents, by nationality and sex

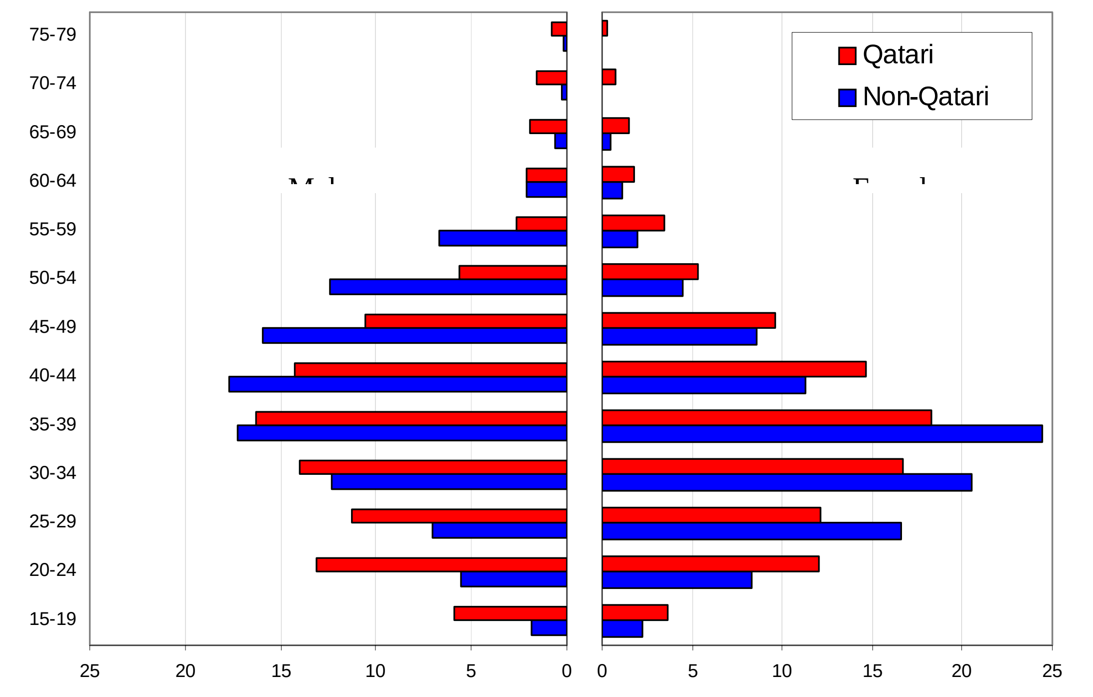

The majority of the respondents were from the capital (54.4%), Doha, while 30.8% lived in Al Rayyan. Only 14.8% of the respondents lived in other regions.

3.3.2 Marital status

Over 82% of the respondents aged 18 or over were then married, with 13.7% stating that they had never been married. A higher percentage of males than females had never been married, with 15.6% of males in the never-married category, compared to 11.8% of females. Conversely, a higher percentage of females than males were widowed or separated from their partner: 3.3% of females were separated, compared to only 0.4% of males.

If marital status is broken down by nationality status there are some striking differences. A graph of this is shown in Figure 3.6. Over 20% of Qataris had never married, compared to only 10% of non-Qataris, while the percentage of married respondents was far higher for non-Qataris. Individuals who were widowed or separated were almost all Qatari, with only a very small percentage non-Qatari.

Figure 3.6. Marital status of individual respondents in qatar, by nationality

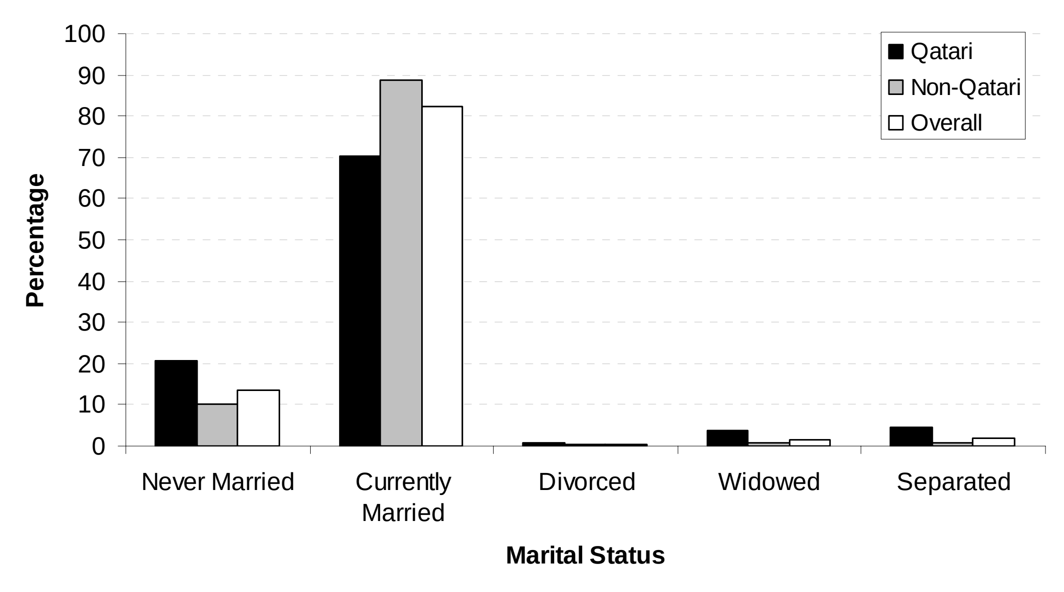

3.3.3 Educational status

Information was collected about the educational status of individual respondents aged 18 and above. The different categories of education given in Table 3.2 show the percentage of individuals who had obtained a certain level of education; 3.2% of the individuals did not finish primary school, while a further 6.4% completed only primary school but went no further. At the other end of the educational spectrum, 37.3% had completed university or higher. Males had a slightly higher educational level than females, with almost 60% having completed college or /pre-university, university or a postgraduate degree or higher, while only 52.4% of females were in the same situation.

3.3.4 Mother tongue

The breakdown of the mother tongue of the respondent is also shown. The majority of individuals spoke Arabic, with 68% stating that this was their main language. Other languages, namely English, Urdu and Malayalam, were spoken by between 5% and 7% of respondents, while only small percentages spoke Farsi or Bengali. There were over 10% of people who spoke other languages. Almost all Qataris spoke Arabic (99%) as their mother tongue. The languages spoken by non-Qataris are presented in Figure 3.7. The majority of non-nationals still spoke Arabic as their mother tongue, but over 10% spoke Urdu and Malayalam and 8.1% spoke English. Over 16% spoke a language not listed in the survey questionnaire.

Figure 3.7. Mother tongue of non-Qatari nationals

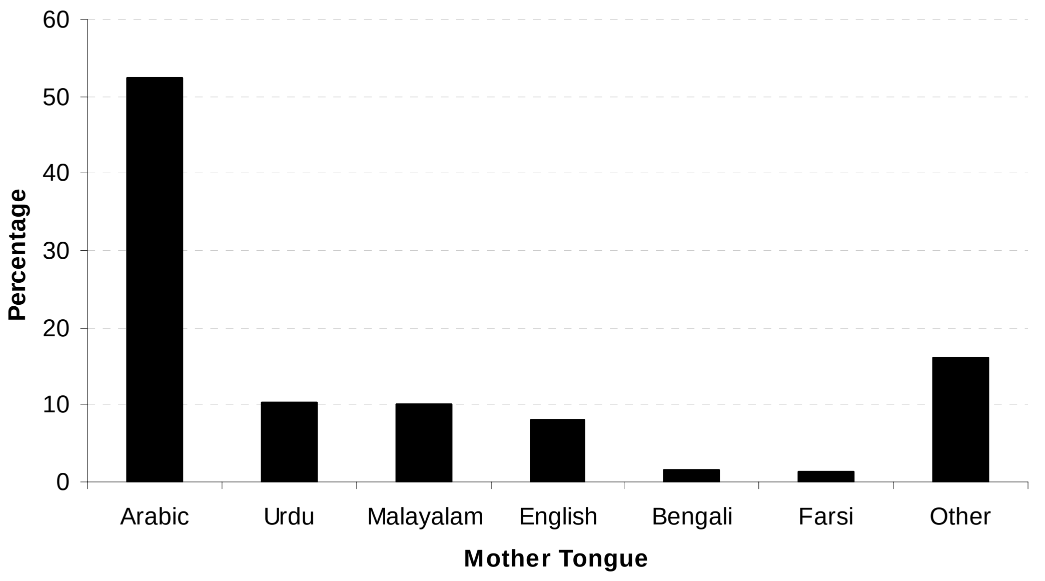

3.4 Work history and benefits

Each of the respondents was asked about their lifetime work history. Further questions were asked regarding the benefits that they took from the work that they did. The work history questions included why a particular person had never worked or was not then working, followed by more information about the type of work they did in their main and other jobs.

3.4.1 Work history

A summary of the work history of the respondents is shown in Table 3.3. This table first shows the percentage of people who had ever had a job, followed by a breakdown of the reasons why an individual had never worked.

Table 3.3 Percentage of respondents who have ever worked, and reasons given for never working

		% ever worked for money	*N*	Reason why never worked: Homemaker	Can't find job	Studies/training	Health problems	Other	*N*
**Sex**									
	Male	93.3	*2418*	6.2	16.4	55.8	0.5	21.1	*160*
	Female	48.8	*2361*	72.1	6.5	10.9	0.6	9.9	*1206*
**Nationality status**									
	Qatari	64.6	*1615*	54.2	8.0	23.9	0.9	13.1	*572*
	Non-Qatari	74.8	*3164*	71.7	7.4	10.7	0.4	9.9	*794*
**Region**									
	Doha	76.4	*2600*	67.4	6.6	12.5	0.7	12.8	*612*
	Al Rayyan	64.3	*1471*	63.0	8.9	18.1	0.3	9.8	*525*
	Other regions	67.6	*707*	59.5	7.6	21.7	1.0	10.3	*229*
**Age group**									
	18 to 29	51.4	*1101*	38.3	11.8	39.3	0.4	10.2	*535*
	30 to 44	76.0	*2390*	81.8	6.2	1.9	0.5	9.6	*571*
	45 to 59	81.5	*1126*	81.4	2.8	0.0	0.8	15.0	*207*
	60 to 69	66.5	*127*	73.3	1.0	0.0	1.5	24.1	*42*
	70 or over	69.8	*35*	71.8	0.0	0.0	6.3	21.9	*11*
**Wealth quintile**									
	Poorest	71.0	*1429*	62.2	7.7	17.4	0.4	12.3	*355*
	< average	69.4	*1197*	70.5	5.1	13.8	1.1	9.5	*322*
	Average	72.2	*932*	63.7	6.4	16.9	1.1	12.0	*284*
	> average	72.9	*708*	61.3	9.9	18.2	0.0	10.6	*227*
	Richest	71.8	*513*	63.2	10.9	14.2	0.0	11.7	*190*
**Total**		**71.3**	***4779***	**64.4**	**7.6**	**16.2**	**0.6**	**11.2**	***1366***

Of all the respondents to the questionnaire, 71.3% had worked in a job for which they received payment in either money or goods at some point in their lives. Almost all males had worked, with over 90% stating that they had been paid for work, compared with less than half of females. Non-Qatari nationals were more likely to have ever worked for money than Qataris, while those in Doha were more likely to have ever worked than in the other regions of Qatar. A large proportion of those in the 30–44 and in the 45–59 age groups had worked at some point (76% and 81.5%, respectively). However, in the youngest and oldest age groups fewer people reported ever working. Only 51.4% of 18–29 year olds had ever worked, while of two age groups over 60 years old, less than 70% reporting working.

The reasons why an individual had never worked displayed some expected trends. The main reason given by women for never working was that they were homemakers (housewives). Almost three-quarters of the women who had not worked fell into this category. Interestingly, 6.2% of males also stated that this was the reason for never having worked. There was a greater percentage of homemakers in the non-Qatari group of respondents, and also in Doha. The next most common reason why someone had never worked was due to being involved in studies or training. This was more likely to be the reason given by the youngest group, 18–29 year olds, where almost 40% who had never had a job as they had been studying.

A number of people stated that they had never worked because they had looked but could not find a job. Overall this was the reason given by 7.6% of respondents. For males who had never worked, 16.4% stated that not finding a job was the reason, while this reason affected only 6.5% of females. This was given as a reason by a higher proportion of younger than older respondents. Health problems were only given as a reason for not working by 0.6% of individuals.

The above information was related to those who had never worked in their life. Also asked was whether the person was currently working for pay, and if not, the reason why they were not (see Table 3.4). Overall, 12.3% of respondents who stated that they had worked at some point in their lives were currently not working. The main reason for this was due to being a homemaker, with 52.5% stating this as the reason for not working at the time of the survey. Almost 70% of the females who were currently not working gave this response, predominantly non-nationals.

Table 3.4 Percentage distribution for reasons why not working, for respondents who have worked at some stage in their lives

		Homemaker	Can't find job	Voluntary work	Studies/training	Retired	Ill health	Other	*N*
**Sex**									
	Male	2.1	15.4	7.9	3.2	54.1	6.8	10.5	*106*
	Female	69.9	6.8	4.7	2.7	8.9	2.6	4.4	*313*
**Nationality status**									
	Qatari	30.0	8.1	1.5	2.2	42.1	8.9	7.2	*168*
	Non-Qatari	67.9	9.5	8.2	3.3	5.8	0.2	5.1	*251*
**Region**									
	Doha	60.2	8.3	6.6	2.2	16.4	1.9	4.4	*232*
	Al Rayyan	44.6	8.0	1.6	3.7	24.3	7.3	10.6	*141*
	Other regions	40.4	15.0	12.0	3.4	27.9	1.3	0.0	*47*
**Age group**									
	18 to 29	56.8	12.6	6.3	14.7	0.0	0.0	9.7	*55*
	30 to 44	68.1	11.9	5.1	1.1	4.9	2.9	6.1	*207*
	45 to 59	42.4	5.4	5.5	1.4	32.1	7.4	5.8	*111*
	60 to 69	5.8	0.0	7.8	0.0	80.4	3.9	2.1	*31*
	70 or over	0.0	0.0	4.0	0.0	96.0	0.0	0.0	*15*
**Wealth quintile**									
	Poorest	52.6	9.4	9.5	3.5	13.1	5.4	6.5	*123*
	< average	55.3	7.1	5.1	1.4	22.4	4.4	4.3	*87*
	Average	48.8	9.7	1.6	3.6	29.4	4.9	2.0	*71*
	> average	62.0	3.0	4.7	2.7	18.4	1.7	7.5	82
	Richest	40.5	18.6	4.0	2.8	24.1	0.0	10.1	57
**Total**		52.7	8.9	5.5	2.9	20.3	3.7	6.0	***419***

The percentage of individuals who stated that they were retired was 20.3%. Of Qataris 42.8% responded that this was the reason for not working at the moment, compared with only 5.8% of non-Qataris. This partly reflects the age structure by nationality status, with a greater proportion of Qatari nationals aged over 60. Ill health was given as the main factor for not working by 3.7% of individuals. A higher percentage of males than females stated this as the reason, with 6.8% compared with 2.6% of females saying that this was the case. There was also a large difference between Qataris and non-Qataris, with 8.9% of national's stating that ill health was stopping them working, compared with only 0.2% of non-nationals. The main region that ill health affected work was in Al Rayyan, where 7.3% of those who lived here stated this reason, compared with 1.9% in Doha and 1.3% in the other regions of Qatar.

3.4.2 Current employment details

If the respondents were then working, further details were obtained regarding their employment. These details included the type of employer and the occupation of the main job. This information is presented in Table 3.5 for the type of employer and in Table 3.6 for the main occupation.

The majority of respondents worked for the government, while 36.6% worked in the private sector. Under 4% were either self-employed or were working for another employer. For females who were then working, a higher percentage worked in the government sector than males, while a greater proportion of males had a private employer or were self-employed. Qatari nationals were mainly employed by the government, with only 7.6% working privately. Conversely, almost half of non-Qataris worked in the private sector, with 42.3% working for the government. Private employers were more common in Doha than in Al Rayyan, with individuals from other regions of Qatar being most likely to be employed by the government.

For the main occupation, this is broken down in Table 3.6 by nationality, sex and region. A greater proportion of non-Qataris reported that they were professionals, while Qataris were more likely to be clerks or service or sales workers. With regard to sex, 39.7% of females were professionals and 32.5% were clerks, compared to 27% and 17.7% respectively for males. Males were more likely to be legislators, senior officials or managers, technicians or service or sales workers. Finally, out of those who were working in Doha the largest percentage of individuals were professionals. In Al Rayyan the percentage of professionals was also the highest, but there was also a high percentage of clerks and service and sales workers. Finally, in the other regions of Qatar 23.3% of workers were professionals and 21.2% were clerks. There was also a much higher proportion of people who were plant or machine operators or assemblers in these outlying regions than in Doha or Al Rayyan.

3.4.3 Benefits received from work

The receipt of benefits from work can form an important part of the employment package and aid in the financial stability of a household. Types of benefits that can be offered are a pension, medical services and cash bonuses. Some jobs also give food or provisions in lieu or salary. Table 3.7 shows the percentage of then working individuals who were receiving benefits and the type of benefits that were received.

Table 3.5 Percentage distribution of employer type for individuals currently working

		Government	Private	Self-employed	Other	Missing	*N*
**Sex**							
	Male	53.3	37.9	4.8	3.7	0.3	*2147*
	Female	60.6	33.2	1.9	3.5	0.8	*834*
**Nationality status**							
	Qatari	87.0	7.6	2.6	2.7	0.1	*873*
	Non-Qatari	42.3	48.6	4.5	4.1	0.5	*2109*
**Region**							
	Doha	49.6	42.8	3.6	3.5	0.5	*1747*
	Al Rayyan	59.0	31.8	6.2	2.7	0.3	*804*
	Other regions	72.1	20.5	1.4	6.0	0.0	*431*
**Age group**							
	18 to 29	53.3	38.6	2.7	5.0	0.4	*507*
	30 to 44	58.2	35.6	3.2	2.8	0.3	*1606*
	45 to 59	52.5	37.0	5.2	4.7	0.6	*805*
	60 to 69	40.3	43.5	13.1	3.1	0.0	*53*
	70 or over	14.2	36.9	48.9	0.0	0.0	*10*
**Wealth quintile**							
	Poorest	57.0	36.0	3.2	3.6	0.3	*747*
	< average	60.8	31.3	3.8	3.7	0.4	*621*
	Average	52.6	39.9	4.4	3.2	0.0	*661*
	> average	51.2	37.7	5.0	5.2	1.0	*528*
	Richest	54.1	39.1	3.8	2.5	0.5	*425*
**Total**		**55.4**	**36.6**	**4.0**	**3.7**	**0.4**	***2981***

Table 3.6 Main occupation of respondents who are currently working

	Nationality status		Sex		Region		
	Qatari	Non-Qatari	Male	Female	Doha	Al Rayyan	Other regions
Legislator, senior official or manager	15.1	15.0	17.3	9.3	16.1	14.1	12.3
Professional (engineer, doctor, teacher, clergy)	23.3	33.6	27.0	39.7	34.4	26.1	23.3
Technician or associate professional	8.9	11.4	12.3	6.3	10.5	8.7	15.1
Clerk (secretary, cashier)	30.8	18.2	17.7	32.5	20.9	24.3	21.2
Service or sales worker	18.2	12.0	15.9	8.6	11.8	18.5	13.1
Agricultural or fishery worker	0.2	1.2	1.2	0.1	0.2	0.5	4.1
Craft or trades worker	0.4	1.6	1.6	0.5	1.2	1.6	0.9
Plant/machine operator or assembler	1.3	3.8	4.1	0.3	1.9	2.7	8.4
Elementary worker	0.7	1.6	1.3	1.3	0.9	2.4	1.0
Missing	1.1	1.8	1.7	1.4	2.1	1.1	0.6
Number of respondents	***873***	***2109***	***2147***	***835***	***1747***	***804***	***431***

Table 3.7 Percentage of workers receiving benefits from work, and distribution of the type of benefit received

					Type of benefit in main job							
		% receiving benefits	Missing	*N*	Pension	Medical	Food/ provisions	Cash bonuses	No benefits	Other	Don't know	*N*
**Sex**												
	Male	47.6	0.2	*2147*	16.6	38.9	5.2	38.7	25.1	15.7	0.3	*1024*
	Female	26.8	0.4	*834*	20.9	28.5	3.7	42.3	10.0	10.8	1.5	*227*
**Nationality status**												
	Qatari	32.6	0.3	*873*	31.3	26.7	3.5	39.3	15.3	7.4	1.3	*286*
	Non-Qatari	45.6	0.3	*2109*	13.2	40.1	5.4	39.3	24.5	17.1	0.3	*964*
**Region**												
	Doha	44.9	0.2	*1747*	18.3	36.7	2.9	40.7	21.9	15.0	0.4	*789*
	Al Rayyan	31.8	0.6	*804*	14.4	32.4	7.1	38.2	20.7	14.6	0.9	*256*
	Other regions	47.8	0.2	*431*	17.5	44.1	10.1	35.5	26.2	14.5	0.8	*207*
**Age group**												
	18 to 29	35.5	0.0	*507*	17.0	40.0	6.1	34.5	15.5	14.3	1.0	*181*
	30 to 44	40.7	0.2	*1606*	16.3	34.0	3.2	41.7	23.4	14.0	0.6	*659*
	45 to 59	48.5	0.6	*805*	19.6	40.6	6.5	37.9	23.9	16.5	0.2	*391*
	60 to 69	35.5	0.0	*53*	10.7	43.0	20.8	28.8	22.0	18.1	0.0	*19*
	70 or over	13.7	0.0	*10*	0.0	0.0	0.0	0.0	0.0	0.0	0.0	*1*
**Wealth quintile**												
	Poorest	42.3	0.7	*922*	15.6	40.1	7.6	34.4	26.6	14.9	0.0	*317*
	< average	40.2	0.0	*797*	17.9	30.2	2.1	38.7	21.4	17.0	1.5	*250*
	Average	42.4	0.0	*573*	19.1	41.0	3.7	45.0	21.9	11.2	0.0	*280*
	> average	44.0	0.6	*412*	18.5	35.2	4.9	36.1	24.9	19.8	1.2	*234*
	Richest	39.3	0.0	*277*	15.4	37.4	6.4	44.5	13.3	10.7	0.0	*169*
**Total**		**41.8**	**0.3**	***2981***	**17.4**	**37.0**	**5.0**	**39.3**	**22.4**	**14.8**	**0.5**	***1251***

Only 41.8% of the respondents to the survey stated that they obtained benefits in their main job. This percentage was higher for males than females, with 47.6% of males receiving benefits as opposed to 26.8% of females. Non-Qataris were more likely to receive benefits than Qatari nationals, and the region with the lowest receipt of benefits was Al Rayyan, with under one-third of workers receiving benefits. Age was also related to benefit receipt, with the highest proportion of people with benefits in the 45 to 59 age group. In this age group 58.8% received some benefits through work, while in the 18–29 age group this figure was 35.5%.

The type of benefit received by those who stated that they did receive benefits from their work was mainly either medical services or cash bonuses: 37% of individuals said that they received medical services, while 39.3% were given cash bonuses. Medical services were more often given to males than females and non-Qataris than Qataris. There were also a higher percentage of people in the areas outside of Doha and Al Rayyan who received this benefit.

Pensions were given to 17.4% of respondents. Qataris were the main beneficiaries of a pension scheme, with 45.6% receiving this benefit compared with 13.2% of non-nationals. Food or provisions were most common in the other regions of Qatar, where 10.1% of the respondents obtained this benefit. There was also a large proportion of individuals stating that they obtained benefits other than those listed in the questionnaire.

4. Risk factors for poor health

4.1 Introduction

Health does not consist of only one dimension; there are a number of different facets which contribute to an individual's being in good health. One of these facets is the exposure of an individual to factors that influence the ability to achieve good health, such as smoking, nutrition and physical activity. There are a number of kinds of behaviour that promote good health and are known to decrease the likelihood of illness.

This chapter studies some of the most common risk behaviour, including smoking, dietary habits (mainly fruit and vegetable consumption) and physical exercise. Also included is an assessment of the proportion of adults and children who are overweight or obese, coupled with an analysis of blood pressure and chemistry for adults, and household environmental risk factors, namely the source of drinking water, sanitation and the type of cooking fuel used in the household.

The questions and measurements for the majority of this chapter were asked to a randomly selected member of each household aged over 18. Childhood obesity was assessed for one child in each household aged under 5 years, while the household characteristics were obtained from the main respondent in each household.

4.2 Use of tobacco, nutrition, physical activities and obesity

4.2.1 Use of tobacco

The risks associated with tobacco use are well known. Cigarette smoking is the most prevalent form of tobacco consumption, accounting for 85% of all tobacco consumed worldwide (FAO, 2003). Consumption of tobacco is increasing worldwide, although this hides disparities between more and less developed countries. More developed countries have seen a fall in the amount of tobacco consumed. This reduction is more than cancelled by the increase in consumption in less developed countries (FAO, 2003).

All forms of tobacco consumption are detrimental to health, whether the tobacco is smoked or chewed. It is estimated that worldwide tobacco is responsible for more than one in 10 adult deaths, with the main illnesses associated with tobacco use being lung cancer (as well as other cancers), vascular disease (including heart disease and strokes), chronic bronchitis and emphysema (World Bank, 1999).In 1999 a survey conducted by the Hamad Medical Centre indicated that the smoking prevalence for males in Qatar was 37%, while for females the prevalence was 0.5% (www.emro.who.int/TFI/countryprofile.htm). This can be compared to the percentage of males and females stating that they smoked in the current survey.

The percentage of adults reporting regular or irregular smoking is shown in Table 4.1. Overall, only 11.1% of respondents stated that they smoked every day, whilst 2.6% say that they smoked, but not every day. This compares favourably with estimates for smoking prevalence for the Middle Eastern region as a whole. Estimates from 1995 indicate that overall prevalence is for the region is 38% (Jha et al., 1995).

Table 4.1 Prevalence of smoking and average daily tobacco consumption

	Current daily smoker	Smoker, not daily	Non-smoker	*N*	Average daily tobacco consumption*	*N*
**Gender**						
Male	19.9	4.2	75.9	*2386*	16.5	*440*
Female	2.2	0.9	96.9	*2335*	16.1	*51*
**Nationality status**						
Qatari	10.9	2.2	86.9	*1597*	18.3	*162*
Non-Qatari	11.3	2.8	86.0	*3124*	15.6	*329*
**Region**						
Doha	12.3	2.7	84.9	*2566*	16.0	*300*
Al Rayyan	10.3	2.3	87.4	*1453*	18.3	*137*
Other regions	8.5	2.5	89.0	*702*	14.3	*54*
**Age group**						
18 to 29	10.2	2.2	87.6	*1086*	15.0	*104*
30 to 44	11.1	2.9	85.9	*2369*	16.0	*246*
45 to 59	12.5	2.5	85.0	*1109*	17.7	*130*
60 to 69	9.1	0.0	90.9	*122*	23.3	*10*
70 or over	3.9	1.8	94.3	*35*	50.0	*1*
**Wealth quintile**						
Poorest	9.9	2.2	87.9	*1210*	16.4	*113*
< average	12.5	3.0	84.5	*998*	17.1	*116*
Average	10.8	2.7	86.6	*1012*	17.6	*103*
> average	11.7	2.1	86.1	*833*	15.7	*90*
Richest	11.2	2.9	85.9	*669*	14.8	*69*
**Total**	**11.1**	**2.6**	**86.3**	***4721***	**16.5**	***491***

* Average calculated only for those who stated they smoked every day

* Tobacco consumption consists of manufactured cigarettes, hand-rolled cigarettes, pipes, cigars, *shisha*, *tomback*, smokeless tobacco and other forms. The average is the number of units smoked, mainly consisting of manufactured cigarettes

Table 4.1 also shows the percentage of smokers broken down by background characteristics. It is clear that women smoke much less than men, with almost 20% of men smoking every day, as compared to only 2% of women. Regular smoking also decreases with age, especially for men over the age of 60. Figure 4.1 shows daily cigarette use by age and sex. Female use is fairly constant between the ages of 20 and 60, while male use peaks in the age group of 20–29.

Figure 4.1. Percentage of respondents reporting daily tobacco use, by sex and age

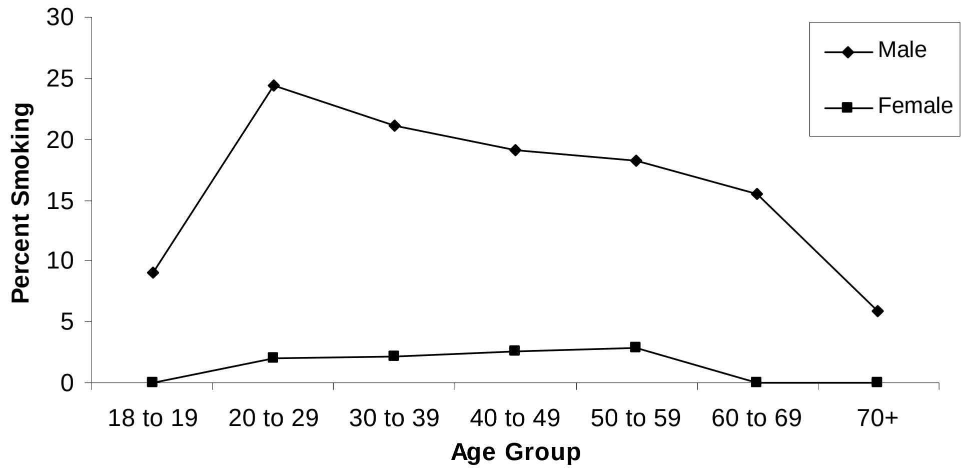

The comparison of the percentage stating that they smoke between 1999 and the current survey indicates a dramatic fall for males, and a large increase for females. This decrease from a prevalence of 37% in 1999 to 19.9% in 2008 may mirror the trends that are happening in the country. However, it is thought that the estimate from the World Health Survey is an underestimate of the true prevalence of smoking amongst males in the population. The percentage of females who smoke has increased from 0.5% in 1999 to 2.2% in 2006. It is still thought that this figure may underestimate the true prevalence of smoking among women. This is likely to be due to smoking being seen as socially undesirable so respondents to the survey were reticent about admitting this habit to the interviewer.

If the figures for 2006 are studied again, it is seen that there was no real difference in the prevalence of smoking between Qataris and non-Qataris. Doha residents reported the highest smoking prevalence compared to Al Rayyan and other regions. There were no large differences between the percentages of daily smokers by wealth quintile, although it is noticeable that the poorest fifth of the respondents do smoke less than the rest of the respondents.

The average number of tobacco products used a day for daily smokers is also displayed in Table 4.1. This figure includes all types of product, such as manufactured and hand-rolled cigarettes, pipes, cigars or *shisha*. The average number of tobacco products smoked, for all smokers who reported daily use, was 16.5, with little difference between men and women. There is some evidence that the average amount smoked decreases as wealth rises, but the differences between groups are small. Of those who smoked, Qataris smoked more than non-Qataris, while in the regions outside of Doha and Al Rayyan the mean number of cigarettes smoked per day is the lowest. Therefore these

regions have the lowest prevalence and the lowest mean number smoked. Number of cigarettes smoked increases as age rises, although this must be interpreted with caution due to few people reporting daily cigarette use in the two oldest age groups.

A breakdown of the specific tobacco products smoked is shown in Table 4.1a. This lists the number of people who smoked regularly each of the different tobacco products and the mean amount that they smoked of these products. Again, this table only contains those who stated that they smoked every day. The majority of daily smokers smoked manufactured cigarettes, and the trends seen for the mean amount smoked of all tobacco products are reflected for manufactured cigarettes. Few people smoked other tobacco products regularly, with the most common being *shisha*. This is mainly smoked by males. No real trends can be seen in these different types of tobacco use due to the small numbers using the different products.

4.2.2 Nutrition (dietary habits)

Knowledge of the dietary habits of a population is vital for targeted planning and implementation of nutritional health policies and programmes. The adequate consumption of fruit and vegetables is one way to reduce the risk of cardiovascular disease and certain cancers (van't Veer et al., 1999), and also is important in providing a diversified diet (WHO, 2003). WHO recommends that an adequate intake of fruit and vegetables is five or more servings in a typical day, with an intake of less than this amount being classified as insufficient. Five servings should equate to about 400 g of fruit and vegetables (WHO, 2003). Table 4.2 shows the percentage of respondents who reported sufficient and insufficient fruit and vegetable intake[*].

Less than one in five (18.8%) respondents reported that they ate sufficient fruit and vegetables on a typical day, with the vast majority stating that they do not eat five servings per day. The mean number of servings eaten on a typical day is 3.3, which is well below the recommended amount. Indeed, almost 40% of respondents stated that they ate two servings or less per day.

There was little variation in sufficient consumption by sex, region or wealth. Non-Qataris are more likely to eat five servings a day than Qataris, while consumption of an adequate amount increases as age rises. One-third of those in the oldest age group of 70 and above consumed the recommended amount or more, while only 16.7% of those aged 18–29 had a sufficient intake.

[*] The measurement of "sufficient" and "insufficient" assumes that the reported servings of fruit and vegetables are of adequate size. The recommended serving size is 80 g. The interviews used guidelines provided by WHO to help the respondents assess if the amount consumed is of sufficient size.

Table 4.1a Average daily consumption of different forms of tobacco

		Manufactured cigarettes*	*Valid count*	Hand-rolled cigarettes *	*Valid count*	Pipefuls of tobacco*	*Valid count*	Cigars, cheroots, cigarillos *	*Valid count*	*Shisha*, tomback *	*Valid count*	Smokeless tobacco*	*Valid count*	Other tobacco*	*Valid count*
**Sex**															
	Male	16.8	*412*	9.5	*16*	3.0	*11*	4.9	*12*	2.5	*32*	5.4	*4*	1.9	*8*
	Female	16.4	*50*	–	*0*	1.0	*1*	–	*0*	1.0	*5*	–	*0*	2.0	*3*
**Nationality status**															
	Qatari	18.6	*154*	10.6	*2*	3.4	*7*	1.2	*3*	2.4	*17*	1.3	*2*	2.8	*4*
	Non-Qatari	15.9	*308*	9.3	*13*	2.0	*5*	6.2	*9*	2.2	*19*	10.1	*2*	1.4	*7*
**Region**															
	Doha	16.4	*281*	9.1	*7*	2.8	*7*	5.3	*11*	2.3	*23*	6.6	*3*	1.6	*9*
	Al Rayyan	18.6	*129*	15.0	*5*	2.0	*4*	1.0	*1*	2.3	*12*	–	*0*	–	*0*
	Other regions	14.4	*53*	1.8	*3*	4.5	*1*	2.0	*1*	2.0	*2*	2.0	*1*	1.6	*2*
**Age group**															
	18 to 29	15.6	*98*	2.2	*2*	1.8	*5*	1.0	*1*	1.3	*11*	1.3	*1*	2.2	*2*
	30 to 44	16.2	*230*	9.7	*8*	5.3	*4*	8.6	*6*	2.4	*20*	8.9	*2*	1.9	*8*
	45 to 59	17.8	*124*	10.8	*5*	1.6	*3*	1.8	*4*	3.7	*6*	1.0	*1*	1.5	*1*
	60 to 69	25.4	*9*	15.0	*1*	–	*0*	1.0	*1*	–	*0*	–	*0*	–	*0*
	70 or over	50.0	*1*	–	*0*	–	*0*	–	*0*	–	*0*	–	*0*	–	*0*
**Wealth quintile**															
	Poorest	17.1	*106*	11.0	*1*	1.7	*4*	1.7	*3*	2.4	*12*	2.0	*1*	1.6	*6*
	< average	17.0	*112*	13.5	*4*	2.0	*1*	1.7	*3*	1.9	*4*	–	*0*	2.8	*3*
	Average	17.7	*98*	6.8	*5*	1.7	*2*	5.5	*5*	1.5	*5*	8.5	*2*	1.8	*2*
	> average	15.9	*82*	8.9	*6*	4.7	*5*	1.0	*1*	3.2	*10*	1.3	*1*	–	*0*
	Richest	15.7	*64*	–	*0*	1.0	*1*	–	*0*	1.6	*6*	–	*0*	–	*0*
**Total**		**16.8**	***462***	**9.5**	***16***	**2.8**	***12***	**4.9**	***12***	**2.3**	***36***	**5.4**	***4***	**1.9**	***11***

4.2.3 Physical activities

Exercise and physical activity have a significant protective effect against ischaemic heart disease, strokes, type-2 diabetes and certain cancers. Evidence also suggests that exercise has positive effects on hypertension, osteoporosis, osteoarthritis and mental and psychological health. Globally, lack of physical activity is estimated to cause 1.9 million deaths a year (www.who.int/dietphysicalactivity/pa/en/index.html). WHO recommends at least 30 minutes of regular, moderate intensity physical effort for at least five days a week, totalling 150 minutes (www.who.int/dietphysicalactivity/factsheet_recommendations/en/index.html).

The World Health Survey asks questions about physical activity at both work and at home, including vigorous and moderate intensity exercise[*]. Exercise includes lifting, digging, cleaning, cooking and washing that cause small or large increases in breathing or heart rate. Furthermore, cycling to and from places for at least 10 minutes is included. The number of days that this exercise is conducted is noted, coupled with the length of time that these activities take place for. From this information the amount of exercise per week is calculated and categorized into sufficient or insufficient, with those reporting more than 150 minutes of exercise a week being classified as sufficient. The results for this are shown in Table 4.2 for all respondents and by subgroup.

The results indicate that 44% of respondents took sufficient exercise over the course of a typical week. The percentage of males who attained the target was higher than females, with 51% of men compared to only 38% of women. As expected, age was also highly related to exercise, with those over 60 indicating a much lower level of exercise than younger age groups. The difference between males and females in reporting insufficient weekly exercise remained fairly constant in all age groups, as can be seen in Figure 4.2.

There is again little difference in the percentages of respondents with sufficient weekly activity by nationality status and wealth, while there are small differences by region, with Al Rayyan indicating a larger percentage of adults not conducting the requisite levels of exercise compared to Doha and other regions of Qatarthe other two regions.

Table 4.2 also shows the percentage of respondents who did over 150 minutes of vigorous activity in a typical week, omitting the moderate activity. The percentage of respondents who reported that they did more than 150 minutes of vigorous activity was only 13.4%. This is about 30% less than the percentage who are classified as doing the sufficient amount of activity if both moderate and vigorous activities are taking into account.

---

[*] Vigorous intensity physical activity is defined as an activity that requires a large amount of effort and causes rapid breathing and a substantial increase in heart rate. Examples are running, fast cycling and heavy shovelling or digging ditches. Moderate-intensity physical activity is defined as a moderate amount of effort that noticeably accelerates the heart rate. Examples are brisk walking, dancing, gardening and carrying/moving moderate loads (under 20 kg).

Table 4.2 Prevalence of insufficient intake of fruit and vegetables and insufficient physical activity

	Fruit and vegetable intake			Any physical activity		Vigorous activity		
	Insufficient*	Sufficient	*N*	Insufficient**	Sufficient	Insufficient***	Sufficient	*N*
**Sex**								
Male	81.0	19.0	*2256*	49.3	50.7	81.4	18.6	*2386*
Female	81.3	18.7	*2226*	62.3	37.7	91.9	8.1	*2335*
**Nationality status**								
Qatari	84.5	15.5	*1484*	54.4	45.6	84.5	15.5	*1597*
Non-Qatari	79.5	20.5	*2998*	56.4	43.6	87.6	12.4	*3124*
**Region**								
Doha	80.7	19.3	*2463*	54.7	45.3	86.8	13.2	*2566*
Al Rayyan	82.3	17.7	*1381*	59.3	40.7	87.4	12.6	*1453*
Other regions	80.4	19.6	*638*	52.1	47.9	84.1	15.9	*702*
**Age group**								
18 to 29	83.3	16.7	*1014*	54.0	46.0	83.4	16.6	*1086*
30 to 44	81.4	18.6	*2243*	55.2	44.8	86.7	13.3	*2369*
45 to 59	79.5	20.5	*1078*	55.3	44.7	88.4	11.6	*1109*
60 to 69	77.8	22.2	*113*	75.4	24.6	94.5	5.5	*122*
70 or over	63.8	36.2	*34*	85.0	15.0	92.8	7.2	*35*
**Wealth quintile**								
Poorest	81.4	18.6	*1139*	56.6	43.4	87.1	12.9	*1210*
< average	81.0	19.0	*951*	57.0	43.0	87.7	12.3	*998*
Average	80.9	19.1	*964*	52.7	47.3	86.1	13.9	*1012*
> average	79.8	20.2	*797*	56.1	43.9	85.9	14.1	*833*
Richest	83.1	16.9	*630*	56.3	43.7	85.6	14.5	*669*
**Total**	**81.2**	**18.8**	***4482***	**55.7**	**44.3**	**86.6**	**13.4**	***4721***

* Insufficient classified as less than 5 portions of fruit and vegetables a day
** Insufficient classified as less than 150 minutes physical activity (moderate or vigorous) in a typical week
*** Insufficient classified as less than 150 minutes vigorous activity in a typical week at work or for exercise

Figure 4.2 Percentage of respondents reporting insufficient weekly exercise, by sex and age

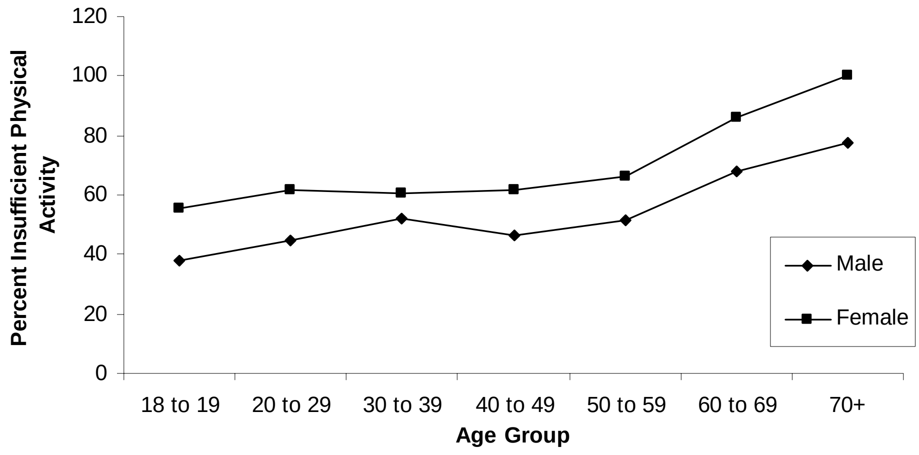

More males than females did over 150 minutes of vigorous activity, with 18.6% of males compared with 8.1% of females. The percentage achieving this amount of exercise fell as age increases, and Qataris were more likely to attain the target than non-Qatari respondents.

4.2.4 Overweight and obesity among adults

The health consequences of being overweight or obese are serious. Cardiovascular disease, diabetes, musculoskeletal diseases and some cancers have all been linked to being overweight (www.who.int/topics/obesity/en/). To measure the degree of overwight, the body mass index (BMI) was used. This is a simple index which measures weight-for-height and is used to classify obesity and overweight in an individual. It is defined as the weight in kilograms divided by the square of the height in metres. A BMI score of higher than 25 is considered overweight, while a score of higher than 30 is termed obese. A score of over 40 is termed morbidly obese. Conversely, a BMI of less than 20 is defined as underweight. Therefore a BMI of between 20 and 25 is considered as the normal range. Even though these categories are extremely useful, there is evidence that risk of chronic disease starts to increase above a BMI of 21.

Table 4.3 displays the percentage of adults in different categories of BMI. Of respondents overall, 39% were classified as being overweight, with 28.8% classified as obese and 3.3% morbidly obese. Therefore almost two-thirds of the respondents were classified as being over the optimum range of BMI. Underweight is classified here as having a BMI of less than 20, and 4.2% of the respondents were in this category. About 10% of respondents did not have a recorded height or weight, either through refusal or because they were not able to have it recorded.

Differences between males and females were seen, with more males being classified as overweight, but more females in the obese and morbidly obese categories. Overall, 35.1% of females were obese, compared to 29.2% of males. There was also a large difference between nationals and non-nationals. About 40% of Qataris were classified as obese, while 28.3% of non-Qataris were in the same categories. About a quarter of

non-national adults were in the normal weight band of BMI, compared to only 20.2% of nationals. The proportion of adults in the normal BMI group fell as age increased, except for the oldest age category. In the 45–59 age group there are only 17% of the adults classified as having normal BMI, with over 45% in the overweight group.

Table 4.3 Prevalence of obesity amongst adults (measured using body-mass index)

	BMI *					
	**Underweight**	**Normal**	**Overweight**	**Obese**	**Morbidly obese**	***N***
**Sex**						
Male	3.8	23.2	43.8	27.1	2.1	*2018*
Female	4.7	26.0	34.2	30.5	4.6	*2016*
**Nationality status**						
Qatari	4.7	20.2	35.2	34.2	5.8	*1334*
Non-Qatari	4.0	26.8	40.9	26.2	2.1	*2700*
**Region**						
Doha	3.3	26.9	40.4	26.7	2.8	*2208*
Al Rayyan	5.5	21.2	38.2	31.1	3.9	*1249*
Other regions	5.1	23.4	35.4	32.0	4.2	*577*
**Age group**						
18 to 29	9.4	34.9	31.2	22.1	2.4	*941*
30 to 44	2.6	23.7	39.4	30.5	3.9	*2003*
45 to 59	2.5	17.0	45.9	31.7	3.0	*959*
60 to 69	5.7	18.4	40.6	31.8	3.5	*99*
70 or over	6.7	30.7	31.4	24.3	6.9	*31*
**Wealth quintile**						
Poorest	5.3	22.5	39.7	29.4	3.3	*1039*
< average	3.4	25.5	41.7	27.5	1.9	*848*
Average	3.9	24.9	38.0	28.7	4.5	*844*
> average	4.2	26.4	37.1	28.8	3.4	*730*
Richest	4.3	24.4	37.7	29.9	3.8	*573*
**Total**	**4.2**	**24.6**	**39.0**	**28.8**	**3.3**	***4034***

* The categories of BMI represent the following: Underweight: BMI of Under 20
Normal weight: BMI between 20 and 25, Overweight: BMI between 25 and 30, Obese: BMI between 30 and 40
Morbidly obese: BMI above 40

4.2.5 Overweight and obesity among children

Childhood obesity is associated with a higher chance of premature death and disability in adulthood (WHO, 2008a), as infants who are obese are more likely to remain obese into later life. Diseases such as cardiovascular disease and diabetes can develop at an earlier age. Many of the problems with overweight and obesity do not become apparent until later life.

For the purposes of this survey, one child aged under 5 was selected to have its weight and height measured. To select this child a roster of all the infants under the age of 5 in the household was drawn up. One child was selected from this roster using a Kish table. The recording of these figures was not without error, with a number of households with a child under 5 years old (as stated on the household roster) not recording any information about the child. Furthermore, a number of heights or weights were recorded that were outside of the possible range for a child of that age. Infants with unreliable heights and weights in this way were not included in this analysis. However, it is unclear whether the measurements for many of the children were reliable, and the BMI calculations may be influenced by this measurement error. The results for childhood BMI, shown in Table 4.4, should be interpreted with this consideration in mind. BMI for each child was calculated using the WHO Anthro software (WHO, 2008b), which uses the WHO standard BMI-for-age tables to assess overweight and obesity. This software takes into account the height, weight and age of the child and assesses the calculated BMI against a reference population of children of the same age. The results are BMI *z*-scores, which are interpreted slightly differently to normal BMI scores. A negative *z*-score indicates that the child has a below average BMI for its age, while a positive score indicates that the child has a higher BMI than a reference child of the same age. The further away from zero the score is, the more extreme the BMI. Scores of below -2 *z*-scores indicate an extremely low BMI for a child, compared to those the same age, while a score of above +2 shows a very high BMI.

The prevalence of children with an extremely large BMI for their age is very high. Only 16% of the children measured in the survey had a BMI close to zero; we considered the normal range to be between –1 and +1 *z*-scores. Of the children, 42.6% had a score between +1 and +2, while 28.7% were assessed as having an extremely large BMI for their age. Furthermore, almost 13% of the children had a BMI which was much less than those in a reference population.

Males were considerably more likely to be in the extreme high BMI category than females, although the reverse was true for those in the category of +1 to +2 BMI *z*-scores. Therefore the percentage of males and females who had a BMI of over +1 *z*-score, indicating a higher BMI than those of the same age in a reference population, is similar. The regions outside of Doha and Al Rayyan had a smaller percentage of high BMI for age than in these regions. Children in households in the poorest wealth quintile had the highest percentage of children who have a very low BMI for their age. Finally, the percentage of children who had a BMI over +1 *z*-score above the reference population markedly increased with age. The percentage of children aged 1 in the categories of +1 to +2 *z*-scores and over +2 *z*-scores was 22.4%. In comparison, in the 4 year olds group the same percentage was 36.2%. There were only a very few children aged under 1 with the requisite measurements to calculate their BMI.

Table 4.4 Distribution of BMI-for-age scores for children aged under 5 years

	BMI *z*-score*						
	**<–2**	**–2 to –1**	**–1 to +1**	**+1 to +2**	**>+2**	**N**	**Missing****
**Sex**							
Male	12.5	16.7	42.6	9.7	18.4	*393*	14.8
Female	12.9	15.5	42.5	15.5	13.6	*377*	15.5
**Nationality status**							
Qatari	11.9	15.8	44.4	10.8	17.1	*300*	14.5
Non-Qatari	13.2	16.3	41.4	13.7	15.4	*470*	15.5
**Region**							
Doha	12.5	16.2	42.2	10.7	18.4	*369*	15.8
Al Rayyan	12.0	15.7	43.1	16.0	13.2	*290*	13.2
Other regions	15.3	17.0	42.1	9.9	15.7	*111*	17.6
**Age of child**							
Under 1	0.0	34.5	0.0	22.1	43.4	*3*	86.4
1 year old	18.0	19.6	39.9	5.3	17.1	*39*	15.7
2 years old	14.0	17.5	42.0	11.6	14.9	*395*	10.6
3 years old	10.3	14.1	46.8	15.3	13.6	*216*	13.9
4 years old	11.4	13.8	38.6	12.9	23.3	*117*	13.2
**Wealth quintile**							
Poorest	15.4	14.1	41.3	11.0	18.3	*210*	17.1
< average	12.9	16.7	37.6	16.2	16.6	*200*	14.3
Average	9.2	20.3	45.7	11.4	13.3	*132*	18.9
> average	12.8	14.9	49.7	10.4	12.2	*130*	10.9
Richest	11.0	15.4	41.6	13.0	19.0	*97*	12.2
**Total**	**12.7**	**16.1**	**42.6**	**12.6**	**16.1**	***770***	**15.1**

* BMI for children is measured in *z*-scores, categorized into five groups .A negative z-score indicates a lower BMI than the norm, while a positive score indicates a higher BMI than the norm. A score of under -2 indicates a very low BMI for the child’s age. A score of over +2 indicates a very high BMI for the child’s age
** Proportion of households with a child under 5 who did not report a height or weight for a child

4.3 Blood pressure

Adult respondents were asked to give consent for their blood pressure measurements to be taken and for a small sample of blood to be obtained from a finger prick to be analysed for blood chemistry (to be discussed in the next section). A non-optimal blood pressure is associated with higher incidence of cardiovascular disease, renal disease and hypertensive disease.

Blood pressure, both systolic and diastolic, was measured three times for every respondent. Blood pressure was obtained by taking the average of these three measures for an individual. Table 4.5a shows the mean blood pressure for all respondents and for certain subgroups of the population. Blood pressure was also classified into three categories, of low, normal or high, following standard guidelines (Longmore et al., 2007). Table 4.5b shows the percentage of individuals in each category. These tables were also cross-classified with a question from another part of the questionnaire, regarding whether the individual had ever had a diagnosis for high blood pressure and also if the respondent was currently on medication for high blood pressure.

Males, as expected, had a higher blood pressure (both systolic and diastolic) than females. Also, blood pressure was closely related to age, with large increases in pressure as age increased. There were no large differences in mean blood pressure across nationality, region or wealth. The classification of blood pressure into groups portrayed a similar story. Overall, 10.7% of respondents had high systolic blood pressure, while 9.4% had high diastolic blood pressure. However, there was a large percentage of respondents who were classified as having low blood pressure. Overall, about 25% of respondents fell into this category. A large proportion of these were female, with about 34% of women having low blood pressure, compared to about 17% of men. Men are about twice as likely to have high blood pressure.

The prevalence of high blood pressure increased as age rose, with about 4% for the 18–29 year olds, compared to 44.3% for systolic blood pressure for the 60–69 year olds and 25.5% for the same age group for diastolic blood pressure. Regarding wealth, the poorest group had the highest percentage of high blood pressure and the lowest percentage of low blood pressure, irrespective of the type of pressure measured. Over a quarter of those in the highest wealth groups had a low systolic pressure. Blood pressure by education is also displayed in Table 4.5b. It is clear from this table that the lower the educational level, the higher the percentage of people with high blood pressure. Almost 25% of those with less than a primary-school education had high systolic pressure, and 18% had high diastolic pressure. This can be compared with those with a university education, where 7% had systolic high blood pressure and nearly 8% had high diastolic pressure.

Table 4.5a Mean blood pressure and mean levels for certain blood chemicals

	Blood pressure (average)			Blood chemistry (average)					
	Systolic	Diastolic	*N*	Cholesterol	TG	HDL	LDL	RBS	*N*
**Sex**									
Male	128.0	82.7	*2010*	208.6	166.0	50.4	125.2	121.5	*1772*
Female	120.7	78.0	*2001*	215.4	143.9	60.5	128.9	120.7	*1758*
**Nationality status**									
Qatari	124.1	79.6	*1294*	210.7	145.3	55.2	127.9	125.8	*1147*
Non-Qatari	124.5	80.7	*2717*	212.6	159.6	55.7	126.6	118.8	*2382*
**Region**									
Doha	123.7	80.4	*2198*	212.6	156.6	56.3	126.9	119.8	*1913*
Al Rayyan	125.1	80.0	*1247*	212.4	151.5	55.2	128.8	125.4	*1134*
Other regions	125.1	80.7	*566*	208.8	155.7	52.9	123.7	117.0	*483*
**Age group**									
18 to 29	118.6	76.9	*920*	200.6	136.9	54.4	119.4	110.4	*776*
30 to 44	122.6	79.8	*2002*	213.6	156.3	56.2	127.5	116.6	*1758*
45 to 59	131.2	84.0	*962*	216.7	166.6	54.8	130.7	135.5	*880*
60 to 69	141.8	86.4	*97*	221.7	149.4	58.5	143.0	141.4	*91*
70 or over	142.7	85.1	*29*	211.7	149.8	51.5	120.0	130.6	*26*
**Wealth quintile**									
Poorest	125.7	81.4	*1037*	211.3	155.4	54.9	126.6	119.3	*906*
< average	122.9	79.3	*855*	209.6	156.2	56.3	124.7	121.3	*760*
Average	124.5	80.1	*836*	213.7	152.6	53.4	130.1	123.3	*720*
> average	124.1	80.6	*712*	210.1	147.3	54.9	127.5	122.2	*627*
Richest	124.2	79.9	*571*	217.0	165.4	59.0	126.6	119.2	*518*
**Diagnosed with high blood pressure**									
No	122.9	79.5	*3623*	211.1	152.7	55.7	126.8	118.5	*3172*
Yes	137.6	87.7	*388*	219.5	173.6	53.5	129.3	142.6	*357*
**On medication for high blood pressure**									
No	123.3	79.7	*3749*	211.7	153.3	55.6	127.1	118.9	*3287*
Yes	140.2	88.9	*262*	215.3	175.4	53.8	126.3	147.8	*242*
**Total**	**124.4**	**80.3**	***4011***	**212.0**	**154.9**	**55.5**	**127.1**	**121.1**	***3530***

Table 4.5b Percentage distribution of high, low and normal blood pressure

	Systolic blood pressure*				Diastolic blood pressure**				Hypertension***	
	Low	Normal	High	*N*	Low	Normal	High	*N*	%	*N*
**Sex**										
Male	16.7	69.4	13.9	*2010*	15.8	71.7	12.5	*2008*	18.8	*2008*
Female	33.9	58.6	7.5	*2001*	32.0	61.7	6.2	*1999*	9.9	*1999*
**Nationality status**										
Qatari	25.3	64.6	10.2	*1294*	25.5	67.7	6.8	*1294*	12.6	*1294*
Non-Qatari	25.3	63.7	10.9	*2717*	23.2	66.3	10.6	*2713*	15.2	*2713*
**Region**										
Doha	25.3	65.1	9.6	*2198*	23.0	68.1	8.9	*2196*	13.7	*2196*
Al Rayyan	27.2	61.0	11.9	*1247*	27.8	62.9	9.4	*1246*	15.1	*1246*
Other regions	21.1	66.6	12.4	*566*	19.0	69.9	11.1	*566*	15.3	*566*
**Age group**										
18 to 29	36.6	60.0	3.4	*920*	34.8	61.3	3.9	*919*	5.7	*919*
30 to 44	26.9	66.3	6.8	*2002*	24.6	68.3	7.2	*2000*	10.6	*2000*
45 to 59	13.6	65.1	21.3	*962*	14.0	68.8	17.2	*962*	26.2	*962*
60 to 69	6.7	49.0	44.3	*97*	10.5	64.0	25.5	*97*	46.5	*97*
70 or over	6.1	47.3	46.6	*29*	8.4	76.6	15.0	*29*	46.6	*29*
**Education**										
Less than primary	23.5	51.8	24.6	*130*	17.9	64.2	18.0	*130*	28.9	*130*
Primary	16.8	68.8	14.3	*260*	15.4	69.0	15.7	*260*	20.6	*260*
Secondary	23.7	62.9	13.5	*435*	22.4	68.2	9.4	*435*	16.2	*435*
High school	29.5	61.8	8.8	*766*	28.3	63.8	7.9	*765*	11.3	*765*
College	25.8	64.2	10.0	*744*	23.1	68.7	8.3	*743*	13.7	*743*
University	26.3	66.7	7.0	*1317*	25.9	66.2	7.9	*1316*	11.2	*1316*
Postgraduate	25.5	65.5	9.1	*151*	16.5	77.3	6.1	*151*	12.7	*151*
Missing	17.0	57.4	25.7	*207*	21.1	62.1	16.8	*207*	28.6	*207*
**Wealth quintile**										
Poorest	21.0	66.4	12.6	*1037*	21.5	67.0	11.5	*1037*	16.9	*1037*
< average	26.9	64.8	8.4	*855*	26.3	67.1	6.6	*855*	11.0	*855*
Average	25.6	63.8	10.7	*836*	24.4	65.4	10.2	*834*	14.8	*834*
> average	28.1	62.0	9.9	*712*	23.6	66.9	9.6	*712*	14.6	*712*
Richest	27.0	61.3	11.7	*571*	24.5	67.6	7.9	*570*	13.8	*570*
**Diagnosed with high blood pressure**										
No	27.1	64.6	8.3	*3623*	25.2	67.9	6.9	*3619*	11.3	*3619*
Yes	8.8	57.9	33.3	*388*	12.3	55.8	31.9	*388*	42.6	*388*
**On medication for high blood pressure**										
No	26.7	64.6	8.7	*3749*	24.9	67.5	7.6	*3745*	12.0	*3745*
Yes	5.4	55.2	39.5	*262*	9.4	55.5	35.1	*262*	47.7	*262*
**Total**	**25.3**	**64.0**	**10.7**	***4011***	**23.9**	**66.7**	**9.4**	***4007***	**14.4**	***4007***

* Systolic BP: low = 0/120; normal = 110 to 140; high = above 140
** Diastolic BP: low = 0 to 70; normal = 70 to 90; high = above 90
*** Hypertension: either systolic BP > 140 or diastolic BP > 90 or both

The average blood pressure varied widely by diagnosis of hypertension. Those who had been diagnosed with high blood pressure had a systolic blood pressure almost 15 mmHg higher and a diastolic blood pressure 8 mmHg higher than those without a diagnosis. The differences between those then taking medication and those who were not were even more extreme. One-third of those who had ever been diagnosed with high blood pressure had a raised level of systolic blood pressure, with slightly fewer of respondents in this group having high diastolic blood pressure.

Nearly 15% of those who had blood pressure measurements taken were classified as being hypertensive. Nearly double the percentage of males than females were diagnosed with this condition, with 18.8% of males and 9.9% of females. The percentages rose dramatically with age, from 5.7% of the youngest age group to nearly half of those aged 60–69 or over 70. Hypertension fell as educational level increased, with over 28% of those with less than primary-school education or who did not report education with the condition, compared to between 11% and 14% of those with at least a high-school education. Just under half of those currently on medication for high blood pressure were included in the hypertensive category based on the results of this survey.

4.4 Blood chemistry

As noted previously, blood was taken from assenting respondents and analysed for certain lipids and sugars. The blood test calculated levels of cholesterol, triglycerides, high- and low-density lipoprotein cholesterol (HDL and LDL respectively) and random blood sugar (RBS). The average levels of each of these measures are displayed in Table 4.5a, while the classifications of the blood chemistry into low, normal and high categories are displayed in different tables.

4.4.1 Cholesterol

Cholesterol is a critical fat which is an important component of cells and plasma. A high level of cholesterol is associated with a raised risk of cardiovascular disease, including heart attacks, strokes and peripheral vascular disease. The results from the Qatar World Health Survey indicate that women had a higher average level of cholesterol than men (Table 4.5a). There were differences in average cholesterol depending on age, with older adults having higher levels than younger respondents. Also, the mean level was associated with wealth, with the richest group having a higher cholesterol level than poorer groups.

Table 4.5c shows the percentage of adults in the three categories of low, normal and high cholesterol. Over a quarter of respondents had a high cholesterol level, with fewer than 10% being classified as having a low level. There was a higher percentage of females in the high cholesterol category, and a lower percentage in the low category. There was also a higher percentage of non-Qataris in the high cholesterol group, and those living in Doha and Al Rayyan had a higher prevalence than those in other regions of Qatar. Age again was strongly associated with high cholesterol levels, with nearly one-third of respondents aged 60–69 being classified into the high cholesterol level. There was a variation in the percentages by educational level, although there were no clear trends associated with this. Wealth also indicated variation, although the richest group did not have the highest prevalence. The highest prevalence was actually in the average wealth group. The poorest had the lowest prevalence of high cholesterol.

4.4.2 Triglycerides

About 90% of dietary intake and 95% of the fat which is stored in the body’s tissues are stored as triglycerides. High levels of these lipids are associated with atherosclerosis,

and as a result, heart disease and stroke. A level of up to 200 mg/dl is considered normal, while over this boundary is considered as high. Triglycerides are affected by eating, and the measurement for this chemical should be conducted after 8 to 12 hours of fasting. The requisite length of time was not left between eating and measurement for the Qatar World Health Survey, and because of this it may be that a high level of triglycerides in an individual is due to the respondent having eaten recently.

The mean triglyceride level was just under 155 mg/dl, which is in the normal range for the chemical (Table 4.5a). There are differences between groups though, with males having a much higher average level than women, by over 22 mg/dl. Differences were also seen by nationality, with non-Qataris having a higher level than Qataris, and age group, with the youngest having the lowest levels and the middle aged (30–59) displaying the highest levels. There was also a large difference depending on whether the individual had been diagnosed with hypertension or was currently on medication for high blood pressure. Those on medication had an average triglyceride level of 175 mm/dl, compared with 153 mm/dl for those not on medication.

Table 4.5c Percentage distribution of cholesterol and triglyceride levels

	Cholesterol*				Triglycerides**		
	**Low**	**Normal**	**High**	***N***	**Normal**	**High**	***N***
**Sex**							
Male	11.1	65.1	23.8	*1782*	70.5	29.5	*1772*
Female	7.3	65.5	27.2	*1757*	80.2	19.8	*1758*
**Nationality status**							
Qatari	8.3	67.9	23.8	*1146*	78.6	21.4	*1147*
Non-Qatari	9.6	64.1	26.3	*2392*	73.7	26.3	*2382*
**Region**							
Doha	8.8	64.4	26.9	*1924*	73.5	26.5	*1913*
Al Rayyan	9.6	64.5	25.9	*1134*	77.2	22.8	*1134*
Other regions	10.2	70.8	19.0	*481*	78.0	22.1	*483*
**Age group**							
18 to 29	13.9	68.3	17.8	*776*	84.0	16.0	*776*
30 to 44	7.5	66.2	26.4	*1765*	73.6	26.4	*1758*
45 to 59	9.1	61.1	29.8	*881*	70.7	29.3	*880*
60 to 69	6.7	61.2	32.1	*91*	76.7	23.3	*91*
70 or over	2.7	71.6	25.7	*26*	80.3	19.7	*26*
**Education**							
Less than primary	8.9	64.9	26.2	*117*	75.5	24.6	*118*
Primary	11.0	62.0	27.0	*235*	75.7	24.3	*235*
Secondary	13.3	61.2	25.5	*385*	77.3	22.8	*383*
High school	11.3	64.2	24.5	*687*	77.3	22.8	*682*
College	6.7	66.3	27.1	*648*	73.2	26.8	*649*
University	8.2	67.6	24.3	*1144*	75.6	24.4	*1139*
Postgraduate	8.3	63.2	28.5	*133*	70.8	29.2	*133*
Missing	6.7	66.5	26.8	*189*	80.8	19.2	*190*
**Wealth quintile**							
Poorest	9.3	68.1	22.7	*908*	76.2	23.9	*906*
< average	9.8	64.6	25.6	*761*	76.5	23.5	*760*
Average	9.7	62.2	28.1	*721*	74.5	25.5	*720*
> average	8.9	65.0	26.1	*631*	76.2	23.8	*627*
Richest	8.0	66.1	26.0	*519*	72.2	27.9	*518*
**Total**	**9.2**	**65.3**	**25.5**	***3539***	**75.3**	**24.7**	***3530***

* Cholesterol: Low = Below 120; Normal = 120 to 240; High = Above 240

** Triglycerides: Normal = 0 to 200; High = Above 200

The classification of individuals into having a normal or high triglyceride level is shown in Table 4.5c. Overall, about 25% of the respondents had a raised level of triglyceride. The trends shown in this categorization mirror the trends seen in the average levels, with males, non-Qataris and the middle aged having the highest percentages with a high triglyceride level. It is also observed that there were a higher proportion of those living in Doha in the high risk category than in the other regions. There was little variation by education or wealth, although there was some evidence of raised levels in the very well educated and in the richest group of adults.

4.4.3 High- and low-density lipoprotein cholesterol

HDL and LDL play an important role in the transportation of cholesterol and triglyceride from the liver to the peripheral tissues. A high level of HDL is seen as evidence of a healthy metabolic system, while a low level of LDL is considered healthy. Low levels of HDL and high levels of LDL are associated with atherosclerosis in a similar way to triglyceride and cholesterol. Mean levels of these lipoproteins are shown in Table 4.5a, while the categorization of the levels is displayed in Table 4.5d. As the measurements were not taken after the individual had fasted the HDL levels will be more reliable than the LDL levels, which are affected more by the respondent having eaten recently.

The mean level of HDL was 55.5 mg/dl, while the mean level of LDL was 127.1mg/dl. This indicates that the mean level of LDL is very close to the edge of the normal adult range, which is 62–130 mg/dl. Females had higher mean levels of HDL, although there was only a small increase in the level of LDL compared to males. Variation in HDL levels across other subgroups was minimal, although there were some differences between age groups. LDL was also fairly consistent across population subgroups, with the exception of age. Levels increased from an average of 119 mg/dl for the 18–29 year olds to a high of 143 mg/dl for 60–69 year olds, above the threshold for a normal LDL level.

Table 4.5d Percentage distribution of high and low density lipoprotein cholesterol levels

	High density*				Low density**				Random blood sugar***			
	Low	Normal	High	*N*	Low	Normal	High	*N*	Normal	Pre-diabetes	Diabetes	*N*
**Sex**												
Male	22.2	76.7	1.1	*1768*	7.8	49.6	42.6	*1355*	84.2	9.0	6.8	*1743*
Female	9.9	88.8	1.3	*1746*	6.6	47.5	46.0	*1373*	84.1	10.6	5.4	*1723*
**Nationality status**												
Qatari	15.4	83.5	1.1	*1144*	7.4	47.5	45.0	*885*	82.2	9.9	7.9	*1129*
Non-Qatari	16.4	82.3	1.3	*2369*	7.1	49.0	43.9	*1843*	85.1	9.8	5.2	*2337*
**Region**												
Doha	16.6	82.1	1.3	*1903*	7.5	49.2	43.3	*1550*	84.5	9.8	5.6	*1874*
Al Rayyan	16.0	83.0	1.1	*1129*	6.3	46.3	47.4	*804*	81.8	11.2	6.9	*1118*
Other regions	14.3	84.7	1.0	*482*	7.8	50.5	41.8	*373*	88.1	6.3	5.7	*473*
**Age group**												
18 to 29	17.0	82.1	0.8	*773*	9.8	52.9	37.3	*537*	89.1	7.6	3.2	*764*
30 to 44	14.8	83.8	1.4	*1749*	6.4	49.1	44.5	*1386*	86.2	9.6	4.3	*1723*
45 to 59	16.6	82.4	1.0	*875*	7.0	45.1	47.9	*712*	77.3	11.7	11.1	*863*
60 to 69	25.9	70.3	3.8	*91*	4.7	39.4	55.9	*71*	71.4	14.7	14.0	*91*
70 or over	21.8	78.2	0.0	*26*	8.7	47.6	43.7	*21*	73.9	10.0	16.1	*26*
**Education**												
Less than primary	10.7	89.3	0.0	*118*	4.0	40.5	55.5	*83*	81.7	4.8	13.5	*115*
Primary	17.8	81.8	0.4	*233*	6.3	48.5	45.2	*175*	80.4	12.3	7.3	*226*
Secondary	18.0	80.4	1.6	*380*	6.8	54.1	39.1	*279*	84.7	10.3	5.1	*374*
High school	15.8	82.2	1.2	*682*	7.9	52.4	39.7	*510*	83.2	10.2	6.7	*672*
College	11.7	87.1	1.2	*645*	6.2	46.2	47.6	*524*	85.2	10.4	4.4	*633*
University	18.3	80.4	1.3	*1134*	8.2	47.6	44.3	*905*	86.8	8.6	4.6	*1124*
Postgraduate	15.2	83.6	1.2	*132*	5.9	57.2	36.8	*103*	85.8	12.2	1.9	*133*
Missing	15.8	82.4	1.8	*190*	6.8	37.1	56.1	*147*	71.6	11.4	17.0	*188*
**Wealth quintile**												
Poorest	16.4	82.4	1.2	*900*	7.0	50.6	42.4	*673*	85.6	8.7	5.8	*890*
< average	14.6	84.3	1.1	*757*	7.8	50.8	41.4	*595*	84.1	10.2	5.6	*747*
Average	16.8	82.1	1.2	*717*	6.7	45.6	47.7	*556*	81.9	11.2	6.8	*704*
> average	16.3	82.7	1.0	*623*	6.3	48.2	45.5	*518*	83.3	9.4	7.2	*611*
Richest	16.3	81.8	1.9	*517*	8.5	46.2	45.3	*385*	85.6	9.6	4.8	*515*
**Total**	**16.1**	**82.7**	**1.2**	***3513***	**7.2**	**48.5**	**44.3**	***2727***	**84.1**	**9.8**	**6.1**	***3466***

Classifying HDL and LDL into categories indicates that the vast majority of respondents had a normal HDL level (82.7%), while only 48.5% of adults had a normal LDL level. There were 22.2% of males with a low HDL level, compared to 9.9% of women, with a lower level indicating greater risk of disease. It is also clear that older adults (aged over 60) were more likely to have a lower level of HDL. The relationship between education and low HDL levels was not clear, with individuals with no education having the lowest percentage in the low HDL category. Respondents in the university education group had the highest percentage, but those in the primary- and secondary-school groups also had a high percentage of people with a suboptimal level of HDL.

The percentage of people with high LDL levels was similar between nationalities and regions. There is some indication more females were in the risky group with a level of over 130 mg/dl with 46% compared to 42.6% for males. Age was again related to the levels of the chemical, with the percentage of people with a high level of LDL increasing as age rose, except for the oldest age group. This oldest group may not conform to the expected trend due to a small sample size. There was a large difference between educational levels, although no clear trends could be discerned. Those with no formal education and those who only attended college (and no higher) had the highest percentage with a high level of LDL, while those with a postgraduate degree had the lowest. It is also important to note that there was a large proportion of missing data on this test, with almost a quarter of the respondents not having a recorded LDL.

4.4.4 Random blood sugar

Blood sugar tests measure the body's capacity to process glucose and are therefore a good test of whether an individual has diabetes or prediabetes. For the random blood sugar test the blood is tested for glucose at any time of the day, and a level over 140 mg/dl is considered as an indicator for prediabetes, while over 200 mg/dl is an indicator for diabetes.

Table 4.5a shows that the mean blood sugar level was 121 mg/dl. This increased with age, with those aged 60–69 having a mean level in the prediabetic range. There was a slight difference between the sexes, with females having a higher mean level, and also a small difference between regions. The regions outside of Doha and Al Rayyan had the lowest mean blood sugar levels, while Al Rayyan had the highest.

Overall, 9.8% of respondents were classified as having prediabetes and 6.1% with diabetes (Table 4.5d). Both prediabetes and diabetes prevalence increased with age, with 28.7% of adults in the 60–69 age group being placed in one of these two categories. There was also great variability by education. Those with less than a primary school education and those who did not report their educational level had the highest level of diabetes, at 13.5% and 17.0% respectively. However, those with less than a primary-school education had the lowest levels of prediabetes, with only 4.8%. If diabetes and prediabetes are taken together the percentage falls slightly as education increases.

4.5 Access to drinking-water and sanitation

4.5.1 Access to drinking-water

Good access to a water supply is a fundamental need and a human right. It is expected in a country of Qatar's level of development that all residents will have access to water, although this section will identify the exact source that household obtained their

drinking-water from. Table 4.6 shows the sources for water for the households included in the survey.

The vast majority of the respondents drank the water that is piped into their houses. 2.4% used bottled water, while another 2.4% bought their drinking water from a tanker/truck vendor. Households in the other regions of Qatar were the most likely to obtain drinking water from bottled sources or from a tanker/truck. Nationals were also more likely to use sources other than piped water for their drinking-water. It is also evident that the poorest group of respondents used tanker/truck vendors more than richer groups.

4.5.2 Access to sanitation

Sanitation facilities play a crucial role in health, personal hygiene and the spread of communicable diseases across a population. The sanitation facilities in Qatar are expected to be of a high standard, with all people having access to an improved source of sanitation. This is highlighted in Table 4.6, which displays the type of toilet used in different households.

The vast majority of the households had a toilet which flushed to a piped sewerage system. Overall, 93.6% of households had this facility. A septic tank was the main alternative, with 5.9% of households using this method. Only 0.4% of households had a pour flush latrine. The pour flush latrine was most common in regions outside of Doha and Al Rayyan and also in the poorest group of households. However, even in this poorest group, only 1% of households used a pour flush toilet. The regions outside of the main two areas also showed the highest use of a flush to a septic tank. It must be noted that it is thought that the proportion of households that have a flush-to–septic tank is underestimated in this survey. This may be due to the respondents not knowing accurately exactly the sewerage arrangements for their household.

4.6 Fuel for cooking

In a similar fashion to the previous section on drinking-water and sanitation, the use of solid fuel for cooking is expected to be very low in Qatar. The health risks when using cleaner fuel, such as electricity, gas or kerosene, are greatly reduced compared to solid fuel. Table 4.7 shows the percentage of households using different types of cooking fuel. It is seen that only three types of fuel were used, none of which is termed a solid fuel. Gas was the most used, with 93% of households using this as their main cooking fuel. Kerosene and paraffin were hardly used at all, with only 0.1% of the households using this type of fuel. Electricity was the method of choice for about 7% of households, although this was higher for non-Qatari households and those in richer groups. Indeed, electricity use increased as wealth increases.

Table 4.6 Percentage distribution of household access to drinking water and sanitation

	Source of drinking water				Type of toilet				
	Piped water	Bottled water	Tanker-truck vendor	*N*	Flush to piped sewerage	Flush to septic tank	Pour flush latrine	Other	*N*
**Sex**									
Male	94.8	2.5	2.7	*2364*	94.0	5.5	0.4	0.1	*2418*
Female	95.6	2.3	2.2	*2321*	93.2	6.3	0.4	0.1	*2361*
**Nationality status**									
Qatari	92.9	3.1	3.9	*1599*	93.7	6.0	0.3	0.0	*1615*
Non-Qatari	96.4	2.0	1.6	*3086*	93.5	5.9	0.4	0.1	*3164*
**Region**									
Doha	96.4	2.2	1.4	*2580*	94.6	5.0	0.2	0.2	*2600*
Al Rayyan	96.9	0.9	2.2	*1436*	94.3	5.7	0.0	0.0	*1471*
Other regions	86.8	6.5	6.6	*669*	88.4	9.6	1.9	0.0	*707*
**Age group**									
18 to 29	95.2	2.5	2.3	*1077*	93.1	6.3	0.6	0.0	*1101*
30 to 44	94.6	2.9	2.5	*2345*	92.9	6.7	0.3	0.1	*2390*
45 to 59	96.5	1.2	2.3	*1101*	95.0	4.4	0.4	0.2	*1126*
60 to 69	95.1	3.0	1.9	*126*	97.7	1.8	0.4	0.0	*127*
70 or over	94.6	1.6	3.8	*35*	96.4	3.6	0.0	0.0	*35*
**Wealth quintile**									
Poorest	93.4	2.2	4.4	*1186*	90.7	8.0	1.0	0.3	*1227*
< average	94.3	2.7	3.1	*999*	94.0	5.8	0.1	0.1	*1020*
Average	96.3	2.5	1.2	*1003*	93.7	6.2	0.1	0.0	*1022*
> average	96.4	2.3	1.4	*828*	95.0	4.7	0.3	0.0	*838*
Richest	96.7	2.3	1.0	*670*	96.5	3.2	0.3	0.0	*672*
**Total**	**95.2**	**2.4**	**2.4**	***4685***	**93.6**	**5.9**	**0.4**	**0.1**	***4779***

Table 4.7 Percentage distribution of households by type of cooking fuel used

	Gas	Electricity	Kerosene/ paraffin	*N*
**Sex**				
Male	92.8	7.1	0.1	*2418*
Female	93.2	6.7	0.0	*2361*
**Nationality status**				
Qatari	96.0	4.0	0.0	*1615*
Non-Qatari	91.6	8.4	0.1	*3164*
**Region**				
Doha	93.7	6.2	0.1	*2600*
Al Rayyan	91.9	8.1	0.0	*1471*
Other regions	92.8	7.0	0.2	*707*
**Age group**				
18 to 29	92.3	7.8	0.0	*1101*
30 to 44	93.8	6.1	0.1	*2390*
45 to 59	92.3	7.7	0.0	*1126*
60 to 69	90.8	9.2	0.0	*127*
70 or over	98.3	1.7	0.0	*35*
**Wealth quintile**				
Poorest	96.4	3.6	0.1	*1227*
< average	96.4	3.5	0.0	*1020*
Average	91.0	8.9	0.1	*1022*
> average	89.9	10.1	0.0	*838*
Richest	88.9	11.1	0.0	*672*
**Total**	**93.0**	**6.9**	**0.1**	***4779***

5. Morbidity prevalence

5.1 Introduction

The delivery of health care to individuals who require it is vital for the health system to contribute to social objectives, which include population health improvement and the reduction of health inequalities. To enable the assessment of whether the delivery of this health care is reaching those in need the morbidity profile of the population is needed, alongside whether those in need are actually receiving care to alleviate their illness. Information on the effective coverage of critical health interventions is becoming a cornerstone in the assessment of health services provision. This chapter therefore summarizes the number of respondents in need of specific health interventions and how many of these respondents are actually receiving the care that they need.

The term health coverage is defined as a probability of receiving a health intervention conditional on the presence of a health care need. This definition is predicated on three premises:

1. that the presence of the health care need is a pre-condition for receiving the associated intervention
2. that coverage is defined at the individual level
3. that coverage refers to the anticipation of a certain outcome of the interaction between the individual and the health system when the health care need emerges.

The Qatar World Health Survey collected information about the prevalence and coverage of certain chronic conditions in adults aged 18 or over. In each household, a randomly selected respondent was asked about different chronic illnesses. First they were asked whether they had been diagnosed with a specific illness. If so, further questions were asked regarding their treatment and whether they were on current medication for the illness. A verbal diagnosis of the likelihood that an undiagnosed illness was present was also conducted. This consisted of a number of questions which were related to the illness in question, where an affirmative response to the question, coupled with other relevant symptoms, may indicate the presence of the illness. The coverage of individuals who are thought to have the illness can then be calculated. Coverage does not only relate to chronic conditions, but also to oral health, vision and hearing. Female and maternal and child health needs are also considered in this chapter.

The Qatar World Health Survey did not ask any questions related to communicable diseases, such as tuberculosis and HIV/AIDS. The rates of tuberculosis and HIV/AIDS in the country are very low, with a HIV prevalence rate of only 0.2% (UNAIDS, 2008). New migrants to the country are screened for certain communicable diseases, a policy which is credited with minimizing the numbers of new infections.

5.2 Noncommunicable diseases

Data were gathered in the survey regarding the prevalence and coverage of a range of noncommunicable diseases, including asthma, arthritis, angina, diabetes, cancer and depression. The need referred to the percentage of respondents who had been formally diagnosed with the condition, while coverage referred to the proportion of respondents who obtained treatment for this condition. During analysis, almost all those who had been diagnosed with a condition had reported that they obtained treatment, indicating that the coverage for all conditions that had been formally diagnosed was at or very close to 100%. These results are therefore not presented. Instead, the percentage of

those who had been formally diagnosed with a condition who had been taking medication in the two weeks before the survey is presented.

For certain diseases the verbal diagnosis was evaluated to give the proportion of respondents who are thought to be suffering from the illnesses. All respondents were asked about symptoms, including those with a formal diagnosis. These verbal diagnoses may give a more accurate picture of the prevalence of a condition in Qatar at the time of the survey, as the reports are based on reports of symptoms rather than formal diagnoses which may have occurred many years before the survey was conducted. The percentage of these verbally diagnosed individuals who were then taking medication is also presented, which will give some idea of the unmet need for medication for different illnesses. It must be noted that if individuals stated that they had never been diagnosed with a condition then they are not asked if they are taking any medication (as it is assumed that because they have not been formally diagnosed then they will not be taking medication).

The results in all tables for this chapter exclude missing data, except where noted.

5.2.1 Arthritis

Table 5.1 displays the percentage of respondents stating that they had been diagnosed with arthritis, and the percentage of those who had been formally diagnosed who are currently taking medication. Across all respondents, the prevalence for arthritis was 7.8%, with under half of these taking medication or receiving treatment for the condition in the two weeks prior to the survey. Females were much more likely than males to report arthritis, with 10.8% reporting the condition compared with 4.8% respectively. Females were also more likely to be on medication. A similar picture is seen when studying the results by nationality status. Qataris were more likely to have been diagnosed than non-Qataris and were also more likely to take medication.

As expected, the prevalence of arthritis increases with age, with only 3% of 18–29 year olds stating that they have arthritis, rising to 27% in the 60–69 age group. The percentage of those on medication also increases markedly in the older age groups. Finally, there is some evidence of differential prevalence by wealth quintile. The poorest group had a low proportion of people diagnosed, and a low proportion currently under medication. The richest had the highest proportion of formal diagnoses and almost 50% had had treatment for the illness in the two weeks leading up to the survey.

The analysis of the symptoms for arthritis, including experiencing pain, aching or stiffness around joints, indicates that a lower percentage of people were suffering from the symptoms than had a formal diagnosis. Overall, 4% of respondents had symptoms commensurate with arthritis. Differentials between groups were similar as before, with more females than males and more Qatari than non-Qatari nationals reporting the symptoms of the disease. Also, the prevalence of arthritis increased with age, with 1.7% of 18–29 years olds being verbally diagnosed, rising to 19.2% in the oldest age group of over 70.

Table 5.1 Prevalence of self-reported and verbally diagnosed arthritis and percentage taking medication

	Formally diagnosed				Verbally diagnosed			
	**% self report**	***N***	**% on medication**	***N***	**% self report**	***N***	**% on medication**	**N**
**Sex**								
Male	4.8	*2414*	36.2	*117*	2.2	*2414*	13.6	*52*
Female	10.8	*2359*	48.5	*256*	5.9	*2359*	32.4	*138*
**Nationality status**								
Qatari	11.8	*1610*	47.9	*190*	6.4	*1610*	25.0	*104*
Non-Qatari	5.8	*3163*	41.2	*183*	2.7	*3163*	30.0	*86*
**Region**								
Doha	7.3	*2596*	45.3	*189*	3.7	*2596*	31.8	*96*
Al Rayyan	8.6	*1470*	43.5	*127*	4.7	*1470*	22.3	*69*
Other regions	8.1	*707*	44.8	*57*	3.5	*707*	23.7	*25*
**Age group**								
18 to 29	3.1	*1100*	32.7	*34*	1.7	*1100*	0.0	*18*
30 to 44	7.1	*2387*	38.0	*169*	3.8	*2387*	21.2	*92*
45 to 59	10.7	*1125*	45.4	*121*	5.3	*1125*	35.7	*60*
60 to 69	26.6	*126*	70.5	*34*	11.2	*126*	42.3	*14*
70 or over	15.6	*35*	79.5	*16*	19.2	*35*	77.4	*7*
**Wealth quintile**								
Poorest	6.6	*1225*	39.4	*81*	3.9	*1225*	22.1	*48*
< average	9.3	*1018*	49.5	*95*	3.6	*1018*	42.7	*37*
Average	7.9	*1022*	45.4	*81*	4.1	*1022*	19.3	*42*
> average	6.1	*837*	39.0	*51*	4.7	*837*	25.5	*39*
Richest	9.6	*671*	47.3	*65*	3.7	*671*	30.3	*25*
**Total**	**7.8**	***4773***	**44.6**	***373***	4.0	***4773***	27.3	***190***

The percentage of those with arthritic symptoms who were then on medication was just over a quarter (27.3%). This percentage is less than that seen of those who have been formally diagnosed who are on medication. Males fared worse on this indicator, with only 13.6% of those with the symptoms taking medication for it in the previous two weeks. For females, the percentage taking medicine was 32.4%. Those in Doha classified as having arthritis from the verbal diagnosis were most likely to be taking medication, and the percentage also increased markedly by age. No one in the youngest age group who was thought to have arthritis was taking medicine, while 77.4% of the over 70s were on medication.

5.2.2 Angina

The prevalence and treatment for angina is displayed in Table 5.2. The percentage of respondents who stated that they had been formally diagnosed is only 0.8%, equating to only 38 people. The main burden of this condition fell on men, Qataris and the elderly. Indeed, in 60–69 age group the prevalence was seen to be 4.3%, rising to 12% in the over 70 age group. For those who had been diagnosed, more males than females were then taking medication. Interestingly, the percentage of those diagnosed and on medication was higher for non-Qataris compared to Qataris, with about three-quarters of non-Qatari nationals taking medication, compared to under half of Qatari nationals (47.3%). This is likely to be due to the different age profiles of the angina sufferers between Qataris and non-Qataris, with non-Qataris being older and more likely to be on medication. Regionally, those in the other regions are more likely to be taking medicine compared to Doha and Al Rayyan.

Table 5.2 Prevalence of self-reported and verbally diagnosed angina and percentage taking medication

		Formally diagnosed				Verbally diagnosed			
		% self report	*N*	% on medication	*N*	% self report	*N*	% on medicat-ion	*N*
**Sex**									
	Male	1.1	*2414*	66.8	*26*	0.3	*2414*	37.1	*8*
	Female	0.5	*2359*	51.3	*11*	0.9	*2359*	0.0	*20*
**Nationality status**									
	Qatari	1.1	*1610*	47.3	*18*	1.3	*1610*	3.4	*21*
	Non-Qatari	0.6	*3163*	75.7	*20*	0.2	*3163*	31.1	*7*
**Region**									
	Doha	0.8	*2596*	60.2	*21*	0.8	*2596*	5.2	*20*
	Al Rayyan	0.6	*1470*	57.1	*9*	0.1	*1470*	100.0	*2*
	Other regions	1.1	*707*	72.8	*8*	0.9	*707*	0.0	*7*
**Age group**									
	18 to 29	0.3	*1100*	0.0	*4*	0.5	*1100*	0.0	*6*
	30 to 44	0.5	*2387*	71.0	*11*	0.4	*2387*	0.0	*10*
	45 to 59	1.2	*1125*	68.6	*13*	0.9	*1125*	17.2	*10*
	60 to 69	4.3	*126*	61.2	*5*	1.6	*126*	65.4	*2*
	70 or over	12.0	*35*	72.5	*4*	0.0	*35*	-	*0*
**Wealth quintile**									
	Poorest	0.6	*1225*	49.7	*8*	0.5	*1225*	0.0	*6*
	< average	1.0	*1018*	70.3	*10*	0.7	*1018*	32.1	*7*
	Average	0.7	*1022*	60.3	*7*	0.7	*1022*	0.0	*7*
	> average	0.7	*837*	64.8	*6*	0.5	*837*	15.3	*5*
	Richest	1.0	*671*	63.2	*6*	0.5	*671*	0.0	*3*
**Total**		**0.8**	***4773***	**62.2**	***38***	**0.6**	***4773***	**10.7**	***28***

Verbal diagnoses were again made for angina, using information about pain or discomfort in the chest when in a hurry or when walking at an ordinary pace. Using the responses from these and other questions the prevalence for angina was only 0.6%. However, in contrast to the formal diagnosis, a higher percentage of females than males were thought to have the disease, with 0.9% of females compared with 0.3% of males. No females with this informal diagnosis were taking medication. The differences between Qataris and non-Qataris were heightened, with 1.3% of Qatari nationals compared to 0.2% of non-Qatari nationals being verbally diagnosed with the disease. The small number of non-Qataris with angina was much more likely to be on medication. The older adults, except for the oldest age group, were verbally diagnosed with angina more frequently than the younger adults, and were likely to be taking medication. Nobody was thought to have angina in the youngest two age groups or was on medication.

5.2.3 Diabetes

Table 5.3 shows the prevalence of diabetes among the survey respondents. The percentage of people who had been diagnosed with diabetes (termed also as high blood sugar in the questionnaire) was 8.3%. This can be compared with the worldwide diabetes prevalence of 5.1% (http://www.eatlas.idf.org/Prevalence/) and is closer to the global prevalence of impaired glucose tolerance (IGT), estimated to be 8.2%. Over 87% of those in Qatar who have been diagnosed with diabetes were then taking medication.

Table 5.3 Prevalence of self-reported diabetes and percentage receiving treatment

		Formally diagnosed			
		**% self report**	***N***	**% getting treatment**	***N***
**Sex**					
	Male	8.1	*2414*	84.8	*196*
	Female	8.5	*2359*	89.5	*200*
**Nationality status**					
	Qatari	11.6	*1610*	89.4	*187*
	Non-Qatari	6.6	*3163*	85.2	*209*
**Region**					
	Doha	7.7	*2596*	89.5	*200*
	Al Rayyan	8.7	*1470*	84.2	*127*
	Other regions	9.7	*707*	86.0	*69*
**Age group**					
	18 to 29	1.4	*1100*	76.5	*16*
	30 to 44	6.1	*2387*	83.6	*146*
	45 to 59	16.2	*1125*	89.1	*182*
	60 to 69	30.8	*126*	92.8	*39*
	70 or over	36.6	*35*	95.8	*13*
**Wealth quintile**					
	Poorest	8.8	*1225*	83.2	*107*
	< average	7.3	*1018*	89.1	*74*
	Average	8.7	*1022*	89.2	*89*
	> average	9.5	*837*	84.0	*80*
	Richest	6.7	*671*	95.0	*45*
**Total**		**8.3**	***4773***	**87.2**	***396***

Table 5.4 Prevalence of self-reported and verbally diagnosed asthma and percentage taking medication

	Formally diagnosed				Verbally diagnosed			
	**% self report**	***N***	**% on medication**	***N***	**% self report**	***N***	**% on medication**	**N**
**Sex**								
Male	2.3	*2414*	30.9	*55*	2.2	*2414*	28.9	*54*
Female	4.2	*2359*	39.8	*99*	3.5	*2359*	35.0	*84*
**Nationality status**								
Qatari	6.2	*1610*	37.1	*101*	5.8	*1610*	33.9	*93*
Non-Qatari	1.7	*3163*	35.8	*54*	1.4	*3163*	29.9	*44*
**Region**								
Doha	2.7	*2596*	34.2	*69*	2.1	*2596*	31.4	*54*
Al Rayyan	3.8	*1470*	33.2	*56*	3.2	*1470*	32.9	*47*
Other regions	4.0	*707*	49.3	*28*	5.2	*707*	34.1	*37*
**Age group**								
18 to 29	3.4	*1100*	19.2	*37*	2.9	*1100*	20.5	*32*
30 to 44	2.6	*2387*	40.8	*62*	2.6	*2387*	32.7	*62*
45 to 59	3.8	*1125*	39.6	*43*	2.7	*1125*	36.4	*21*
60 to 69	8.3	*126*	57.0	*10*	9.2	*126*	51.1	*12*
70 or over	5.0	*35*	60.7	*2*	4.8	*35*	63.5	*2*
**Wealth quintile**								
Poorest	3.4	*1225*	38.6	*42*	3.5	*1225*	31.6	*43*
< average	2.8	*1018*	60.6	*28*	2.8	*1018*	45.9	*29*
Average	3.0	*1022*	24.7	*31*	2.1	*1022*	25.9	*21*
> average	4.0	*837*	26.6	*33*	3.2	*837*	27.7	*26*
Richest	2.9	*671*	33.6	*19*	2.7	*671*	29.2	*18*
**Total**	3.2	***4773***	36.6	***154***	2.9	***4773***	32.7	***138***

There was a large variation of prevalence by age, with over one-third of over 70s stating a diagnosis of diabetes, compared to 1.4% of 18–29 year olds. The percentage of those having treatment for the condition also increased with age, although the differences between the age groups are not as large as for diagnosis. Almost 12% of Qatari nationals had been diagnosed with diabetes, compared to less than 7% of non-Qatari nationals. A larger proportion of nationals were also currently on medication if they had been diagnosed by a doctor with having diabetes.

Overall, 3.2% of the respondents stated that they had been diagnosed with asthma, and 36.6% of these had been taking medication in the two weeks prior to the interview. Females had a higher prevalence than males, and there was a large disparity between nationalities. Nationals of Qatar reported a prevalence of 6.2%, compared to only 1.7% for non-Qataris. Regarding treatment, more females were on medication than males, but there was only a small difference between the percentages of individuals on medication by nationality status. Even though more Qataris were diagnosed, there was a similar proportion of non-Qataris taking medication. Differences between the age groups were not large with respect to formal diagnoses, except for the elderly. However, the percentage being treated rose for each age group, possibly indicating that severity of asthma intensifies as age increases.

The percentage of respondents who are verbally diagnosed with asthma was similar to the percentages that have been formally diagnosed. Indeed, the differences between subgroups of the population are very similar to those seen for formal diagnoses, indicating that the questions capture well those who suffer from asthma. One interesting difference was by region however, with areas outside Doha and Al Rayyan having a higher prevalence (5.2%) or asthma sufferers than these two regions, which had an asthma prevalence of 2.1% and 3.2% respectively.

5.2.4 Depression

The respondents were asked to state whether they had ever been diagnosed with depression, with 1.9% reporting that this was the case (see Table 5.5). This figure obviously excludes those who had never had a formal diagnosis, and further questions asked elucidated those who were depressed yet had not been given a formal diagnosis. Depression was reported by a higher percentage of women, with 2.9% reporting the condition compared with only 0.9% of men. Depression was higher in Qataris too, with 2.7% stating that they suffered, in comparison with 1.4% of non-Qataris. Finally, 60–69 year olds had a prevalence of depression of 3.5%. Younger age groups reported a prevalence of under 2%.

Table 5.5 Prevalence of self-reported and verbally diagnosed depression and percentage taken medication

	Formally diagnosed				Verbal diagnosis			
	**% self report**	***N***	**% on medication**	***N***	**% self report**	***N***	**% on medication**	***N***
**Sex**								
Male	0.9	*2414*	20.4	*21*	0.9	*2279*	6.5	*21*
Female	2.9	*2359*	31.9	*67*	3.4	*2189*	13.2	*74*
**Nationality status**								
Qatari	2.7	*1610*	30.8	*44*	3.3	*1467*	12.5	*49*
Non-Qatari	1.4	*3163*	27.5	*45*	1.5	*3001*	11.0	*46*
**Region**								
Doha	1.6	*2596*	31.5	*42*	1.6	*2434*	14.2	*38*
Al Rayyan	2.3	*1470*	26.1	*34*	3.2	*1397*	11.7	*44*
Other regions	1.8	*707*	29.3	*13*	1.9	*637*	4.2	*12*
**Age group**								
18 to 29	1.8	*1100*	39.0	*20*	2.2	*1021*	11.5	*22*
30 to 44	1.9	*2387*	25.8	*45*	2.6	*2242*	10.1	*58*
45 to 59	1.8	*1125*	33.5	*20*	1.3	*1052*	20.4	*14*
60 to 69	3.5	*126*	0.0	*4*	0.6	*119*	0.0	*1*
70 or over	0.0	*35*	-	-	1.9	*33*	0.0	*1*
**Wealth quintile**								
Poorest	1.5	*1225*	29.5	19	2.0	*1150*	5.4	23
< average	2.5	*1018*	30.2	25	1.6	*944*	21.5	15
Average	1.8	*1022*	22.7	19	1.9	*957*	10.4	18
> average	1.8	*837*	24.6	15	2.3	*779*	6.1	18
Richest	1.6	*671*	43.5	11	3.2	*637*	17.8	20
**Total**	1.9	***4773***	29.1	***89***	2.1	***4468***	11.7	***95***

For those with a formal diagnosis of depression, 29.1% were taking medication or receiving treatment. Females were more likely to be receiving care than males, with treatment percentages of about 32% and 20% respectively. Even though prevalence is highest in the 60–69 age group, none of those diagnosed was receiving treatment. Conversely, those of younger ages who had depression were likely to be obtaining treatment. There is also slight evidence that those in the richest wealth quintile were more likely to report treatment than those in other quintiles.

A series of questions was asked about how the respondents had felt in the 12 months leading up to the survey, in order to gauge the prevalence of depression that had not been formally diagnosed. Using questions such as "have you had a period when you felt sad, empty or depressed?" and "have you felt that your energy levels have decreased?", a verbal diagnosis can be made. This information indicated that 2.1% of the respondents had depression, which is more than those who had been formally diagnosed. The same percentages of males were diagnosed verbally as diagnosed formally (0.9%). However, the percentage of females diagnosed increased from 2.9% for the formal diagnosis to 3.4% for the verbal diagnosis. More Qataris than non-Qataris were diagnosed, with 3.3% compared with 1.5%, and Al Rayyan had the highest prevalence in the country. The percentage of those verbally diagnosed with depression who were on medication was only 11.7%. A higher proportion of females than males took medication.

It is possible to directly compare the results from the verbal diagnosis with the response from another question on the questionnaire. This question asked "overall in the last 30 days, how much of a problem did you have with feeling sad, low or depressed?". This question will be further analysed in Chapter 7. However, the comparison between the verbal diagnosis and this question indicates that 2.1% of respondents were verbally diagnosed as being depressed, while 4.5% of respondents stated that they often or always had a problem with feeling sad, low or depressed. Thus the verbal diagnosis did not diagnose over half of those who felt sad or depressed in the month prior to the interview, assuming that the responses to this alternative question are reliable. Of the 4.5% of people who said that they have been sad or depressed, 36.3% were on medication, compared with 26.8% of those who said that they were sad or depressed either not at all, a little or sometimes.

5.2.5 Stroke

The respondents to the survey were asked whether they have ever been told by a health professional that they had had a stroke in their lifetime. Only 1 in 200 individuals responded that they had been told that they have suffered from a stroke (Table 5.6). Clearly this is related to age, with 2.2% of those aged 60–69 stating that they had had a stroke, and 1.8% of those over 70. However, there were only very few cases in each of these groups. A higher percentage of Qatari nationals also reported a stroke than non-Qatari nationals. The proportion of those who had had a stroke who were then on medication or other treatment varied widely between groups, possibly due to the small sample sizes. Overall, just under one-third of those who had had a stroke were currently taking medication.

5.2.6 Chronic lung disease

Chronic lung disease includes emphysema, bronchitis and chronic obstructive pulmonary disease. Table 5.6 shows that the percentage of those who had been diagnosed with lung disease in the sample is 0.9%, with about 36% of these people stating that they were then taking medication or receiving treatment for the condition. Once again, this was more common in Qataris than non-Qataris; Qataris were also more

likely to be taking medication; the trend increases with age. Regarding the prevalence by region, individuals in the Al Rayyan reported prevalence close to that of Doha, while the prevalence in Doha was almost half of that seen in other regions. Differences between subgroups in the taking of medication may be due to the small number of people stating that they had been diagnosed with one of these diseases.

5.2.7 Hypertension

A diagnosis of high blood pressure had been given to nearly 10% of the respondents (see Table 5.7). This contrasts with the figure obtained from the blood pressure measurements taken in the survey, where 14.4% of people were diagnosed with hypertension (see Table 4.5b). Males had a higher prevalence than females and were also slightly more likely to be taking medication for this condition. Qataris suffered from this condition more than non-Qatari nationals, although the percentage on medication for those diagnosed does not differ greatly. Age was also a major factor, with reports of hypertension increasing markedly between each successive age group. A higher proportion of those in the older age groups were also under medication, with almost 100% of those in the 60 to 69 age group taking medication.

5.2.8 Cancer

The survey asked all respondents about whether they had ever been diagnosed with malignant tumour. Out of the 4773 respondents to the survey, 25 stated that they had been diagnosed, resulting in a prevalence of 0.6%. A diagnosis was more likely in women, in nationals, in the region of Al Rayyan and in the richer wealth quintiles. Also, those in the older age groups were far more likely to have had cancer.

The main type of cancer reported was breast cancer. Over 50% of females reporting cancer stated that this was the form of cancer that they suffered from, while 22% reported cancer of the female reproductive organs. For men, cancers grouped into "other" cancers were the most common, although 30% stated that they had prostate cancer.

5.2.9 Summary

The prevalence of these major chronic illnesses/diseases by subgroup displays some interesting patterns. It is clear that Qataris were more likely to report a diagnosis than non-Qataris for all conditions analysed. Furthermore, females were more likely to report diagnoses than males for all conditions except for angina and hypertension. These systematic differences may be due to a number of reasons and may not be due to actual differences in prevalence. The higher prevalence of the conditions in these groups may be due to better health care seeking behaviour from Qataris and females and hence the higher likelihood of a diagnosis. The higher rates may also be due to a higher likelihood of reporting a condition to the interviewer, with females and Qataris feeling more comfortable in stating the various conditions in an interview setting. However, the differences may also be due to actual differences in the respondents and the fact that nationals and females do suffer from chronic diseases more than non-nationals and males.

Table 5.6 Prevalence of self-reported stroke and chronic lung disease and percentage taking medication

	Stroke				Chronic lung disease			
	**% self report**	***N***	**% on medication**	***N***	**% self report**	***N***	**% on medication**	***N***
**Sex**								
Male	0.4	*2414*	34.7	*10*	0.9	*2414*	34.1	*22*
Female	0.6	*2359*	30.3	*15*	1.0	*2359*	37.6	*22*
**Nationality status**								
Qatari	0.9	*1610*	13.8	*14*	1.5	*1610*	43.1	*25*
Non-Qatari	0.3	*3163*	56.5	*10*	0.6	*3163*	26.8	*20*
**Region**								
Doha	0.4	*2596*	55.3	*12*	0.8	*2596*	31.7	*21*
Al Rayyan	0.7	*1470*	13.3	*10*	0.9	*1470*	49.8	*13*
Other regions	0.4	*707*	0.0	*3*	1.5	*707*	26.4	*10*
**Age group**								
18 to 29	0.3	*1100*	31.6	*4*	0.8	*1100*	23.3	*8*
30 to 44	0.4	*2387*	27.2	*10*	0.7	*2387*	22.9	*16*
45 to 59	0.6	*1125*	18.3	*7*	1.3	*1125*	54.9	*15*
60 to 69	2.2	*126*	69.6	*3*	3.1	*126*	35.9	*4*
70 or over	1.8	*35*	100.0	*1*	1.5	*35*	100.0	*1*
**Wealth quintile**								
Poorest	0.3	*1225*	0.0	*4*	1.2	*1225*	31.9	14
< average	0.6	*1018*	19.0	*6*	0.8	*1018*	63.4	8
Average	0.6	*1022*	66.1	*6*	1.0	*1022*	21.2	10
> average	0.6	*837*	37.8	*5*	0.5	*837*	16.4	4
Richest	0.4	*671*	22.0	*3*	1.2	*671*	45.9	8
**Total**	0.5	***4773***	32.1	***24***	0.9	***4773***	35.9	***44***

Table 5.7 Prevalence of self-reported hypertension and cancer and percentage taken medication

	Hypertension						Cancer					
	% self report	*N*	% on medication	*N*	% diagnosed	*N*	Breast	Female reproductive organs	Prostate	Lung	Other	*N*
**Sex**												
Male	10.9	*2414*	69.1	*261*	0.3	*2414*	–	–	30.3	19.5	50.2	*7*
Female	8.9	*2359*	65.0	*211*	0.9	*2359*	56.7	22.5	–	0.0	20.8	*18*
**Nationality status**												
Qatari	12.5	*1610*	65.4	*200*	0.9	*1610*	21.3	25.1	0.0	10.1	43.6	*13*
Non-Qatari	8.6	*3163*	68.6	*272*	0.4	*3163*	63.4	6.6	17.6	0.0	12.4	*12*
**Region**												
Doha	10.2	*2596*	69.0	*265*	0.4	*2596*	21.5	24.2	18.5	0.0	35.8	*11*
Al Rayyan	9.2	*1470*	65.4	*135*	0.9	*1470*	65.2	11.7	0.0	6.2	17.0	*12*
Other regions	10.4	*707*	64.3	*72*	0.4	*707*	8.7	0.0	0.0	30.6	60.8	*2*
**Age group**												
18 to 29	2.3	*1100*	24.9	*25*	0.2	*1100*	–	–	–	–	–	*0*
30 to 44	6.4	*2387*	50.4	*153*	0.3	*2387*	29.5	10.4	0.0	0.0	60.1	*6*
45 to 59	20.1	*1125*	76.1	*226*	1.0	*1125*	46.9	32.2	4.1	0.0	16.9	*11*
60 to 69	39.6	*126*	94.4	*50*	6.2	*126*	45.3	0.0	21.4	9.5	23.9	*8*
70 or over	51.4	*35*	83.1	*18*	1.8	*35*	0.0	0.0	0.0	100.0	0.0	*1*
**Wealth quintile**												
Poorest	11.1	*1225*	65.5	136	0.3	*1225*	8.9	0.0	0.0	31.5	59.6	*2*
< average	8.1	*1018*	74.9	83	0.3	*1018*	58.4	0.0	17.1	0.0	24.5	*3*
Average	11.5	*1022*	70.8	117	0.9	*1022*	29.5	13.9	18.3	0.0	38.3	*9*
> average	8.3	*837*	64.7	69	0.9	*837*	50.6	20.2	0.0	10.4	18.8	*7*
Richest	10.1	*671*	58.1	68	0.7	*671*	53.3	31.5	0.0	0.0	15.2	*5*
**Total**	**9.9**	***4773***	**67.2**	***472***	**0.6**	***4773***	**41.1**	**16.4**	**8.3**	**5.4**	**28.9**	***25***

5.3 Coverage for vision care

Cataracts are diagnosed when the lens of an eye becomes cloudy and opaque, causing partial or total blindness. The lens can be removed and replaced with a clear, plastic substitute using laser surgery, which normally results in vision being fully restored. The Qatar World Health Survey asked respondents aged over 60 years whether they have been diagnosed with a cataract in one or both eyes, and also if they had had surgery to remove this cataract in the previous five years.

Table 5.8 presents the results for cataracts for those aged over 60 in the survey. Of them, 16.8% stated that they had had a cataract in the 5 years prior to the survey. Cataracts were least common in Doha, where only 11.8% of respondents reported this condition, compared with 20.1% in Al Rayyan and 27.3% in the rest of Qatar. However, in Doha there were 6% of the respondents who did not know whether they had suffered from cataracts. Qatari nationals are more likely to have stated that they had had a cataract, with over a quarter of this subgroup having been diagnosed with one, compared to 7.3% of non-Qataris.

For those who had been diagnosed with cataracts, nearly three-quarters had had eye surgery to remove the cataract. There was variation between subgroups, although the sample sizes involved were small. However, over 80% of males with a cataract had had an operation compared with 63% of females. Qataris were also far more likely to have had surgery. Finally, almost all sufferers in regions outside Doha and Al Rayyan had had surgery (92%), a greater percentage than in Doha (71%) and Al Rayyan (67%).

5.4 Coverage for oral health

Oral health problems can occur at any age. Risk of tooth decay is increased by the consumption of foods which are rich in sugar and starch. This kind of diet is becoming more common due to changing food habits and lifestyles. Table 5.9 presents the results for oral health in Qatar, focusing on the percentage of respondents who were missing at least one tooth or had reported problems with teeth in the previous year and the receipt of any dental care for these problems.

Table 5.8 Percentage of over 60 year olds with cataracts and percentage of sufferers having surgery

	Cateracts				
	% with cateracts	% don't know	*N*	% cateract surgery*	*N*
**Sex**					
Male	17.4	3.8	*98*	81.1	*17*
Female	15.9	4.3	*63*	63.3	*10*
**Nationality status**					
Qatari	25.9	4.7	*83*	79.7	*21*
Non-Qatari	7.3	3.3	*78*	54.7	*6*
**Region**					
Doha	11.8	6.1	*85*	70.8	*10*
Al Rayyan	20.1	1.2	*52*	66.8	*10*
Other regions	27.3	2.8	*24*	92.2	*7*
**Wealth quintile**					
Poorest	18.4	2.9	*48*	74.9	*9*
< average	17.7	0.0	*32*	73.7	*6*
Average	16.2	10.8	*36*	71.3	*6*
> average	17.6	0.0	*25*	100.0	*4*
Richest	12.0	5.7	*21*	37.2	*2*
**Total**	**16.8**	**4.0**	***161***	74.5	*27*

* Percentage of those with cataracts in the last 5 years who had surgery

Table 5.9 Percentage of respondents with dental problems and coverage for oral health

	% with dental problems	*N*	% treatment	*N*	% missing teeth	*N*
**Sex**						
Male	18.9	*2414*	73.8	*457*	31.9	*2412*
Female	27.6	*2359*	76.7	*651*	35.4	*2353*
**Nationality status**						
Qatari	31.0	*1610*	79.7	*500*	39.1	*1603*
Non-Qatari	19.2	*3163*	72.1	*607*	30.9	*3161*
**Region**						
Doha	20.3	*2596*	75.4	*527*	32.2	*2594*
Al Rayyan	26.7	*1470*	73.7	*393*	34.2	*1467*
Other regions	26.5	*707*	79.7	*187*	37.9	*703*
**Age group**						
18 to 29	21.5	*1100*	74.2	*237*	21.5	*1096*
30 to 44	24.0	*2387*	76.7	*573*	32.1	*2383*
45 to 59	21.8	*1125*	75.7	*249*	43.9	*1124*
60 to 69	33.1	*126*	72.3	*42*	63.1	*126*
70 or over	28.3	*35*	46.0	*10*	85.4	*35*
**Wealth quintile**						
Poorest	22.4	*1225*	75.5	*275*	33.4	*1224*
< average	22.7	*1018*	72.7	*231*	33.2	*1016*
Average	23.1	*1022*	75.7	*236*	32.9	*1018*
> average	23.0	*837*	75.3	*193*	33.9	*836*
Richest	25.7	*671*	79.1	*173*	35.7	*669*
**Total**	**23.2**	***4773***	**75.5**	***1107***	**33.7**	***4773***

The percentage of people who had a dental problem in the last 12 months was 23.2%. Females were far more likely to have had problems than males, and Qataris more than non-Qataris. This followed the same pattern as reported for previous conditions. There was a small increase in dental problems as age increased, and a small rise in reported need for care from poor to rich. The coverage for dental problems was over 75%, meaning that three out of four people with a reported problem obtained treatment. There were no large variations between groups, although there is some evidence that coverage was lower in Doha and Al Rayyan than in the other regions of Qatar and also that Qataris received more care when needed than non-Qataris.

Over one-third of the survey respondents reported missing at least one tooth. Females were slightly more likely to be missing a tooth than males, with 35.4% of females compared with 31.9% of males stating this. Qatari nationals were also more likely to have lost a tooth than respondents who were not classified as Qatari. The percentage of people with missing teeth also rose as age increased, as is expected. Over one-fifth of those in the youngest age group had a missing tooth, compared to almost two-thirds in the 60–69 year old age group.

5.5 Injuries and treatment of injuries

In 2020, road traffic accidents are projected to account for over 5% of the adult disease burden in the world (Peden et al., 2004). This disproportionately affects young adults, and especially young adult males. Injuries at home are also important to document, if they result in bodily injury. Questions on both types of injury were asked in the Qatar survey, along with the receipt of medical care for the injuries received in these accidents. It was observed that all those who had suffered bodily injury due to a road traffic accident or an accident at home obtained medical care or treatment, and thus the

results are not presented below. The percentages of people who had had a road traffic accident or a home accident are shown in Table 5.10.

The distribution of respondents who had had a road traffic accident differed between groups of the population. Of males, 2.5% stated that they had been injured in this way, in comparison with only 1.6% of females. Age was also associated with road traffic accidents, with the younger ages being affected more than older ones. This is shown in more detail in Figure 5.1, which plots the percentage involved in a road traffic accident by age and sex. It is clear that it is the younger males who were involved in road accidents, with the percentage falling in each subsequent age group. For women the picture was more complex, with the highest percentage in the 50 to 59 age group.

Table 5.10 Percentage of respondents injured in a road traffic accidents and at home

	Road traffic accidents	Injuries at home	*N*
**Sex**			
Male	2.5	1.4	*2414*
Female	1.6	1.0	*2359*
**Nationality status**			
Qatari	2.3	1.3	*1610*
Non-Qatari	1.9	1.1	*3163*
**Region**			
Doha	1.8	1.1	*2596*
Al Rayyan	2.2	1.2	*1470*
Other regions	2.4	1.2	*707*
**Age group**			
18 to 29	2.3	1.0	*1100*
30 to 44	2.1	1.4	*2387*
45 to 59	1.9	1.1	*1125*
60 to 69	0.9	0.0	*126*
70 or over	0.0	0.0	*35*
**Wealth quintile**			
Poorest	2.4	1.2	*1225*
< average	2.6	2.0	*1018*
Average	1.4	1.0	*1022*
> average	1.8	0.8	*837*
Richest	1.6	0.8	*671*
**Total**	2.0	1.2	***4773***

The percentage of Qataris involved in a road traffic accident was 2.3%, compared with 1.9% of non-Qataris. Finally, there is some evidence that there was a higher percentage of accidents in regions outside of Doha, and also that the poorer households had a higher percentage of accidents than the richer households.

For serious accidents in the home, 1.2% of respondents stated that this had occurred to them. This again affected males more than females and the young more than the old. The poorer group also had a higher rate percentage than the richer groups. However, the differences between all different subgroups of the population were small.

Figure 5.1 Percentage of respondents involved in a road traffic accident, by age and sex

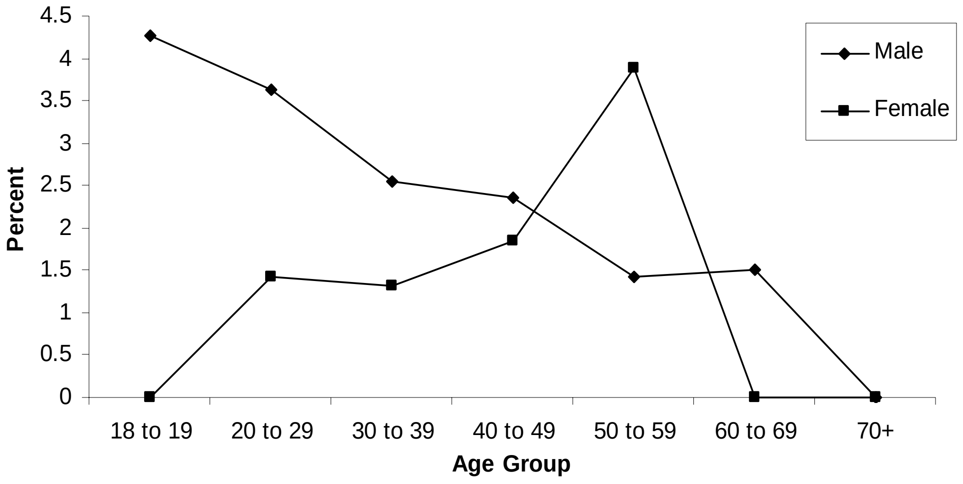

5.6 Female health care and screening

5.6.1 Cervical cancer

Cancer of the cervix is the second-most common cancer in women worldwide, with about 500 000 new cases and 250 000 deaths each year (WHO, 2006). In Qatar in 2005, this cancer is estimated to be the fourth-most common cause of cancer death in women (WHO, 2008), with an age-standardized incidence of 15 per 100 000 women. The primary approach to controlling cervical cancer is through prevention rather than treatment of the actual disease (WHO, 2004).

Cervical cancer takes many years to develop. Changes can be detected in the cervix for some time before the appearance of the cancer. Therefore screening women for these changes can detect the early development of the disease and, coupled with treatment, the development of the cancer can be halted. Screening programmes are therefore an important part of the fight against this cancer. The Qatar World Health Survey asked women aged over 18 questions regarding the last time that they had had a pelvic examination and whether a PAP smear test occurred at the same time. Only women who were selected to answer the individual questionnaire were asked these questions. Table 5.11 shows the percent of women who had had a pelvic examination, and when this happened. It also shows the percentage of women who had had an examination who also had a Pap smear test at the same time.

Table 5.11 Percentage of women screened for cervical and breast cancer

	Pelvic examination					Pap smear test			Mammography*				
	**Within last 3 years**	**4–5 years ago**	**More than 5 years ago**	**Never**	***N***	**% with test**	**don't know**	***N***	**Within last 3 years**	**4–5 years ago**	**More than 5 years ago**	**Never**	***N***
**Nationality status**													
Qatari	34.4	6.8	8.7	50.2	*844*	69.1	17.4	*421*	12.4	4.1	5.1	78.4	*321*
Non–Qatari	39.7	6.1	12.1	42.2	*1275*	68.7	20.9	*737*	21.6	3.5	5.3	69.5	*350*
**Region**													
Doha	36.0	6.7	12.7	44.7	*1121*	65.0	23.0	*620*	15.5	3.3	7.4	73.8	*363*
Al Rayyan	38.3	5.4	8.9	47.5	*694*	72.9	16.9	*365*	20.0	4.6	2.0	73.4	*208*
Other regions	42.0	7.2	7.6	43.2	*305*	73.9	13.4	*173*	17.7	4.1	3.8	74.4	*100*
**Age group**													
18 to 19	0.0	0.0	0.0	100.0	*43*	–	–	–	–	–	-	-	-
20 to 29	35.7	3.6	2.7	58.0	*501*	85.0	6.3	*210*	–	–	-	-	-
30 to 39	46.1	7.4	7.9	38.6	*906*	75.1	12.9	*557*	–	–	-	-	-
40 to 49	33.9	7.3	19.5	39.3	*459*	55.8	32.2	*279*	16.3	4.0	4.5	75.2	*459*
50 to 59	24.2	8.5	23.0	44.3	*158*	43.5	41.3	*88*	19.2	2.9	7.7	70.3	*158*
60 to 69	14.4	5.4	36.8	43.5	*43*	25.4	65.0	*25*	22.2	4.4	5.8	67.6	*43*
70 and over	0.0	0.0	0.0	100.0	*10*	–	–	–	6.4	5.2	0.0	88.4	*10*
**Wealth quintile**													
Poorest	37.8	6.9	10.3	45.0	*526*	68.7	18.8	*289*	10.9	2.9	5.0	81.2	*141*
< average	41.5	6.6	9.6	42.4	*462*	72.0	16.6	*266*	17.0	2.7	5.3	75.0	*144*
Average	37.8	5.9	13.6	42.7	*434*	66.0	23.7	*249*	16.7	5.0	9.0	69.4	*164*
> average	33.4	6.7	10.1	49.9	*379*	66.6	20.1	*190*	22.2	4.9	4.0	68.9	*121*
Richest	36.2	5.3	9.9	48.6	*319*	70.4	19.2	*164*	21.1	3.5	0.8	74.6	*100*
**Total**	**37.6**	**6.3**	**10.7**	**45.4**	***2120***	**68.8**	**19.6**	***1158***	**17.2**	**3.8**	**5.2**	**73.8**	***670***

* For women aged over 40 only

Over 45% of women stated that they had never had a pelvic examination, and only 37.6% of women had had an examination in the previous three years. More non-Qataris had had a pelvic examination than Qataris, with 35.8% having an examination in the previous three years. The percentage of women who had had an examination also differed by age. No women aged 18–19 had ever had an examination, while 58% of those aged 20–29 had also never been examined. Women aged between 30 and 39 were the most likely to have had an examination in the previous three years, but after this age band the percentage of women who had been examined falls again. About 10% of women did not answer this question, and this percentage varied widely between groups. This may have influenced the results obtained from the study.

For those who had had a pelvic examination, almost 69% had had a PAP smear test as well, although about 20% did not know whether this had been conducted. Women in the older age groups were more likely not to know if the smear test had been conducted at the same time as the pelvic examination. This leads to the percentage of women having had a test falling as age increased, although if those who reported that they did not know whether a test was conducted are assumed to have had a test, the percentage remained constant. However, over 10% of the women reported that a Pap smear test was not conducted during the pelvic examination.

5.6.2 Breast cancer

Breast cancer is the most common cancer among women globally, with about 500 000 deaths each year (www.who.int). In Qatar the estimated incidence of women in 2002 with breast cancer is 24 per 100 000 (age-standardized; WHO, 2008). A simple way to reduce mortality from breast cancer is to screen for the disease. The screening is usually done using a mammography, with or without a physical examination. This should be done once every two or three years and is seen to be worthwhile only in women aged over 40.

Women were asked when the last time that they had had mammography, or an x-ray of their breasts, to detect breast cancer. The results are also shown in Table 5.11. Almost three-quarters of the respondents said that they had never had a mammography, while only 17.2% said that they had had one in the previous three years. Over 20% of non-Qatari women had been screened in the last three years, compared to 12.4% of Qatari nationals. There is some evidence that the percentage screened in the previous three years increased as age rose. Wealth was also related to the percentage of women who had had mammography. The poorest quintile were much less likely to have been for screening in the previous three years, with only 10.9% reporting this, compared to over 20% for the top two wealth quintiles.

5.7 Care during pregnancy and childbirth

5.7.1 Antenatal care

Care during pregnancy and at the time of birth is vital for the health of the mother and the child. Antenatal care enables the detection of complications with respect to pregnancy, including anaemia, hypertensive disorders and bleeding, and also identifies higher-risk multiple pregnancies. Information and counselling can be given to the expectant mothers which enables the mother to make informed decisions about the pregnancy. It is recommended by WHO that a mother have at least three visits to a doctor during the term of the pregnancy. In Qatar the policy is for the expectant mother to have an antenatal check every month, with the checks increasing to every 10 days in

the last month. The Qatar NHA therefore recommends that mothers have at least 12 antenatal visits.

Women who had had a birth in the previous five years were asked about the care that they received during the pregnancy. The results from these questions are shown in Table 5.12. Overall, out of the 2123 women aged between 18–49, nearly 50% had given birth in the previous five years. These women were assessed for the number of antenatal visits and the checks that were done during the visits.

Overall, almost all women attended three or more antenatal visits. Only 3% of women saw a health professional this number of times, while 99.8% of the women had at least one visit to a health care provider. The target of 12 or more antenatal visits was achieved by 37.6% of women, with those living in the areas outside Doha and Al Rayyan being most likely to have had 12 or more visits. One aspect of the table to note is that 11.2% of the women with a birth in the previous five years did not report whether they were given antenatal care. It is unknown whether these mothers had antenatal care or not.

While at an antenatal visit a mother should have certain checks and tests carried out to ensure that the pregnancy is going to plan. These include blood pressure measurements, weighing, blood and urine samples taken and ultrasound. Also, the expectant mother should be told about the signs of pregnancy complications and what to do if these complications occur. In the survey, mothers were all asked about these different checks and tests. For all mothers, over 99% had had these checks apart from being told about pregnancy complications. For this component of antenatal care, only 87% of pregnant mothers stated that they were given this information. These results exclude the mothers who did not give a response to the antenatal care questions.

The high percentage of mothers who received the various antenatal checks means that there were few differences between women with different background characteristics except for being told about signs of complication. For this facet of antenatal care a higher percentage of non-Qataris received this information than Qatari nationals, while the percentage also increased with wealth. Finally, as age increased, the percentage of women having had the antenatal checks generally decreased, with those aged 40–49 being least likely to be told about the signs of complication.

Adequate care can be defined as having three or more antenatal visits, with blood pressure and a blood sample taken and who were told about pregnancy complications. If these dimensions of antenatal care are analysed together, only 83.4% of mothers are defined as receiving adequate care during pregnancy. The percentage of mothers with adequate care again fell with age, with only 73.4% of the oldest mothers obtaining adequate care, while 100% of those aged 18 or 19 received the requisite level. Wealth also was related to care, with a higher proportion of the wealthy than the poor attaining good care. Finally, more non-Qatari nationals had adequate antenatal care. Almost 80% of Qatari mothers obtained adequate care, compared to 85.4% of non-Qataris.

Table 5.12 Percentage of mothers receiving antenatal care

	Women 18–49	% with a birth last 5 years	Sufficient care*	% at least 1 visit	% at least 2 visits	% at least 3 visits	% at least 12 visits	% miss antenatal	Blood pressure checked	Weight measured	Blood sample given	Urine sample given	Ultra-sound	Told about complication signs	*N*
**Nationality status**															
Qatari	*823*	47.5	79.8	99.5	97.7	96.6	37.2	12.4	99.5	99.7	99.3	99.7	98.9	84.7	*375*
Non-Qatari	*1300*	51.2	85.4	100.0	98.0	97.2	37.8	10.5	99.6	99.9	99.3	99.4	99.9	88.5	*628*
**Region**															
Doha	*1107*	48.0	83.3	100.0	98.0	97.0	36.1	10.9	99.2	99.7	99.3	99.7	99.4	86.9	*499*
Al Rayyan	*713*	52.4	82.7	99.5	97.6	96.6	36.0	11.8	99.8	100.0	99.4	99.2	99.8	86.7	*357*
Other regions	*303*	50.0	85.5	100.0	98.5	97.9	46.7	10.8	100.0	100.0	99.2	99.7	99.5	88.2	*148*
**Age group**															
18 to 19	*65*	10.0	100.0	100.0	100.0	100.0	34.9	19.1	100.0	100.0	100.0	100.0	100.0	100.0	*7*
20 to 29	*580*	56.6	82.4	99.7	97.9	96.0	34.6	11.1	99.6	94.6	98.6	98.8	100.0	86.4	*310*
30 to 39	*968*	65.0	85.1	99.9	98.1	97.5	39.8	10.3	99.7	95.4	99.6	99.8	99.3	88.2	*602*
40 to 49	*511*	18.3	73.4	100.0	96.7	96.7	32.5	17.0	98.1	90.5	100.0	100.0	99.2	80.5	*84*
**Wealth quintile**															
Poorest	*589*	52.8	82.7	99.7	98.1	96.3	41.8	13.6	99.8	99.8	99.4	99.2	99.5	86.2	*267*
< average	*497*	50.7	79.1	100.0	97.9	97.9	33.1	9.4	100.0	99.8	99.8	99.8	99.8	82.4	*227*
Average	*420*	46.8	83.5	100.0	98.2	97.8	32.0	11.5	99.3	100.0	99.6	100.0	100.0	85.6	*191*
> average	*356*	48.4	87.3	99.5	97.2	95.0	39.3	11.4	99.1	99.6	99.1	100.0	98.4	93.5	*172*
Richest	*260*	49.1	86.4	100.0	98.0	98.0	42.2	8.8	99.2	100.0	98.1	98.5	100.0	90.1	*146*
**Total**	***2123***	**49.8**	**83.4**	**99.8**	**97.9**	**97.0**	**37.6**	**11.2**	**99.5**	**99.8**	**99.3**	**99.5**	**99.5**	**87.0**	***1003***

Percentage of women with a birth in the last five years who saw a health professional 3 or more times, had blood pressure taken, had a blood sample taken and was told about pregnancy complications

5.7.2 Attendance at delivery

Care during labour and delivery is a further way to improve the health of both mother and child, as complications can be identified and averted if a skilled professional is available during this time. Table 5.13 gives the percentage of births in the five years before the survey by the type of attendant. Also given is the place of delivery and the type of health facility, if appropriate.

All women in Qatar had skilled attendance at delivery, although 5% of women with births did not answer this question. The majority (82.6%) of women had a doctor attending (the category of doctor includes specialists such as gynaecologists, obstetricians and surgeons), while 15.7% had a nurse or a midwife. Very few (1.8%) had an auxiliary nurse or midwife in attendance. Non-Qataris were more likely to have had a doctor at the birth than Qataris, while mothers in Doha were the least likely.

Nearly all mothers (99.5%) who reported their location of birth gave birth in a hospital. Only 0.5% of the mothers who answered this question stated that they gave birth in another health facility. The type of hospital or health facility, either government or private, was also noted. Most mothers used a government facility, with 86.3% of mothers giving birth in these facilities. Only 13.7% of births occurred in private facilities. Private facilities were used mainly by non-Qataris, where over 20% of births took place, compared to only 2.3% for mothers who were Qatari nationals. Government hospitals were used the least in Doha, with only 79.3%, while in the other regions 95% of births occurred in these types of facility. Private facilities were also used more by those aged 20–39 than those aged 40–49. The seven mothers who gave birth and were aged 18 or 19 all gave birth in a government hospital.

5.8 Child health care (preventative and curative)

The care of children in the first few years of their lives can be enhanced through vaccinations and adequate care when the child does fall ill. The Qatar survey asked questions about care, both preventative and curative, relating to the youngest child in each household, if that child was under 5 years old.

It is important to note that there was a slight mistake in the questionnaire regarding this section. The intention was to obtain information for the youngest child under 5 in each household. However, due to an incorrect filter number on the questionnaire many households were not actually asked this group of questions and as a result this section was only asked to women with a birth in the previous five years. Males interviewees who live in a household with a young child were not asked about this information. Therefore the information obtained may not be representative of the general population and the results need to be interpreted with this in mind.

Table 5.13 Percentage of mothers receiving assistance at birth and place of delivery

	Assistance at birth				Place of delivery		Type of institution		
	Doctor	Nurse	Auxiliary nurse/ midwife	*N*	Hospital	Other health facility	Government	Private	*N*
**Nationality status**									
Qatari	78.4	18.1	3.5	*376*	99.2	0.8	97.7	2.3	*376*
Non-Qatari	85.1	14.2	0.7	*627*	99.7	0.3	79.5	20.5	*628*
**Region**									
Doha	82.0	17.0	1.0	*497*	99.4	0.6	79.3	20.7	*499*
Al Rayyan	82.6	15.5	1.9	*358*	99.6	0.4	92.5	7.5	*358*
Other regions	84.5	11.3	4.2	*148*	99.6	0.4	95.0	5.0	*148*
**Age group**									
18 to 19	100.0	0.0	0.0	*7*	100.0	0.0	100.0	0.0	*7*
20 to 29	83.3	15.2	1.4	*311*	99.1	0.9	83.6	16.4	*311*
30 to 39	81.3	16.8	1.9	*602*	99.7	0.3	86.2	13.8	*603*
40 to 49	87.6	10.3	2.1	*84*	100.0	0.0	95.7	4.3	*84*
**Wealth quintile**									
Poorest	83.4	14.8	1.8	*267*	99.7	0.3	82.7	17.3	*268*
< average	78.3	19.2	2.5	*227*	98.9	1.1	90.9	9.1	*227*
Average	84.5	14.8	0.8	*190*	99.5	0.5	84.8	15.3	*191*
> average	82.0	16.4	1.7	*173*	99.6	0.4	89.1	10.9	*173*
Richest	82.0	16.4	1.7	*146*	100.0	0.0	84.6	15.4	*146*
**Total**	**82.6**	**15.7**	**1.8**	***1003***	**99.5**	**0.5**	**86.3**	**13.7**	***1005***

5.8.1 Child vaccinations

For the youngest child under 5 years old in the household the respondent was asked about the vaccinations that had been received. In this section only those children aged over 1 year old were analysed, as it is recommended that a number of vaccines be given in the first year of birth. The survey asks about three different vaccines:

- DPT: immunization to protect against diphtheria, whooping cough and tetanus. It is recommended that children should have five shots, at 2, 4, 6 and 18 months of age, with the final one at ages 4 to 6
- HCV B: hepatitis B immunization. Three injections over a 6 to 12 month period are needed for full protection
- MMR: a single jab to protect against measles, mumps and rubella.

The vaccinations should be recorded on a card so that an official record is kept. In the survey there were three ways of obtaining the vaccination information. First, the information was transcribed from the vaccination card. Secondly, if the card was not available but the respondent stated that the card existed, detailed information was obtained from memory. The final method was for those children without a vaccination card. Less detailed information was recorded about the vaccinations for these children.

For infants with a vaccination card, 94.9% of children over 1 year of age have had three injections of DPT, while 87% have had the full course of three injections of HCVB (see Table 5.14a). The MMR jab had been given to 89.2% of infants. Interestingly, girls were less likely to have had the requisite jabs than boys. The difference was especially noticeable for the MMR vaccination, where 93.6% and 85.6% of boys and girls respectively had had the jab. Al Rayyan had the lowest percentage of infants adequately vaccinated for DPT and HCV B, while Doha had the lowest percentage for MMR. The other regions consistently had the highest percentage of children vaccinated.

A number of children were reported to have a vaccination card although it was not available during the interview for the vaccinations to be transcribed from. The results for these children are shown in Table 5.14b. The percentage of children in this group with vaccinations was far lower than the percentage for those with an observed card. The percentage of children reported to have had at least one DPT jab was only 32.9%, and out of these infants only 35.5% were reported to have had the requisite three injections. The percentage reported to have had an MMR jab was 20%.

The mothers of the final group of infants, those without a vaccination card, were asked if their child had ever had a vaccination; 46.4% stated that their child had been vaccinated. The rate was higher for boys than for girls, for nationals compared with non-nationals and for the richer households. Further questions were asked to identify the exact jabs that had been received, but these were not well answered and were not analysed.

Table 5.14a Percentage of infants with various vaccinations if vaccination card is seen for infants 1–5 years old

	Number of children	DPT1	DPT2	DPT3	HCVB1	HCVB2	HCVB3	MMR
**Sex of child**								
Male	*165*	97.8	95.2	95.0	96.6	95.0	88.7	93.6
Female	*203*	100.0	96.9	94.7	97.6	93.5	85.6	85.6
**Nationality status**								
Qatari	*146*	99.5	94.8	95.6	95.9	92.5	86.3	88.3
Non-Qatari	*223*	98.7	97.0	94.4	97.9	95.3	87.5	89.8
**Region**								
Doha	*193*	99.1	96.1	94.8	98.6	96.1	87.4	87.9
Al Rayyan	*121*	98.4	94.6	92.6	94.2	91.6	84.7	90.1
Other regions	*55*	100.0	100.0	100.0	98.6	93.4	90.8	92.1
**Wealth quintile**								
Poorest	*97*	99.3	97.0	94.9	96.0	88.4	82.2	88.1
< average	*86*	96.6	93.3	93.9	98.7	98.1	87.3	85.4
Average	*79*	100.0	94.4	94.9	98.2	97.8	89.1	94.2
> average	*64*	100.0	98.5	95.9	96.4	93.7	92.4	87.1
Richest	*43*	100.0	100.0	95.1	95.8	93.6	85.5	93.4
**Total**	***369***	**99.0**	**96.2**	**94.9**	**97.2**	**94.2**	**87.0**	**89.2**

Table 5.14b Percentage of infants with dpt and mmr vaccinations for infants with an unseen card and for infants without a card

	Infants with an unseen card						No card	
	**Number of children**	**% with DPT jab**	**% missing**	**% with at least 3 jabs***	**% with MMR jab**	**% missing**	**Number of children**	**% ever had vaccination**
**Sex of child**								
Male	*203*	34.1	5.9	46.5	18.1	10.6	*32*	53.6
Female	*136*	31.6	8.6	18.5	23.1	14.0	*15*	32.3
**Nationality status**								
Qatari	*134*	38.8	8.7	35.8	23.1	12.9	*7*	92.0
Non-Qatari	*206*	29.2	5.8	35.3	18.0	11.2	*43*	39.4
**Region**								
Doha	*168*	31.4	9.0	42.6	23.3	15.2	*28*	49.2
Al Rayyan	*123*	36.5	4.6	32.4	13.6	7.5	*18*	40.4
Other regions	*50*	29.4	5.4	19.8	24.6	11.4	*3*	55.0
**Wealth quintile**								
Poorest	*89*	33.0	11.3	38.3	17.8	15.8	*11*	21.1
< average	*72*	24.9	4.5	58.3	16.3	6.8	*13*	31.2
Average	*59*	33.2	7.2	22.8	24.8	9.6	*13*	46.9
> average	*61*	41.5	1.7	23.3	11.7	7.8	*6*	100.0
Richest	*59*	33.4	8.5	38.5	31.8	18.7	*6*	70.4
**Total**	***340***	**32.9**	**6.9**	**35.5**	**20.0**	**11.9**	*340*	**46.4**

* Percentage of those reported to have had a jab who are reported to have had 3 or more jabs

5.8.2 Childhood illness

The care of children when they are ill is obviously of vital importance, with certain strategies of giving an ill child more fluids and food being seen to be helpful for aiding recovery. It is also important to study the types of illness that are experienced by the children in the survey. To assess this, respondents were asked about the most recent time that their child had been ill, and if the child had ever been ill, what symptoms they had. The respondents were then asked about the care given when the child was ill.

Table 5.15 shows the percentage of children who had been ill in the month before the survey and who had ever been ill. If the child had ever been ill, then the symptoms for this illness were collected. It is seen that about 40% of the children had been ill in the previous month, while just over three-quarters of the children had ever been ill. Illnesses were most common in the previous month among Qataris, with 45.8% ill, compared to non-Qatari infants, with 36.3% ill. Children outside the capital were more likely to have been ill than those in Doha, while the percentage of illness fell as wealth increased.

A list of eight symptoms was given to the respondent to select as many as appropriate regarding the previous illness episode. These include fever, diarrhoea and coughing, alongside serious symptoms such as convulsions, difficult or fast breathing and blood in the stool. The most common symptom was fever, with 80.8% of infants displaying this, followed by coughing, experienced by just under half of children who had ever been ill. The other symptoms were experienced by far fewer children, with difficult or fast breathing, diarrhoea, vomiting and the inability to eat or drink being listed by between 15% and 17% of respondents.

A difference was seen between infants in Qatari and non-Qatari households. The percentage of Qatari infants with symptoms of difficult or fast breathing, diarrhoea and being unable to drink or eat is far higher than the corresponding percentages for non-Qataris. The percentages of the other symptoms are very similar.

The treatment of common symptoms such as fever, cough and diarrhoea was assessed. The giving to children of more liquid or more or the same amount of food is promoted as a method to aid recovery. The results for the treatment of these three symptoms are shown in Table 5.16. In all three cases, only a minority of children were given more to drink, with between 12.6% and 16.3% of mothers stating that their child was treated in this way. The percentage of infants given more or the same amount to eat as usual was also low, with between 26.9% and 29.3% of children cared for in this way.

Table 5.15 Percentage of children under 5 years who have been ill and symptoms of the illness

	Number of children	% ill in last month	% ever ill	% of children who have been ill with symptoms								
				Fever	Cough	Difficult/ fast breathing	Diarrhoea	Blood in stool	Vomiting	Unable to eat/drink	Convulsions	Other
**Sex of child**												
Male	*530*	40.2	78.5	80.1	49.0	17.7	20.5	1.4	16.2	17.2	3.3	6.5
Female	*503*	40.3	74.8	81.5	50.7	14.0	14.0	1.9	15.0	16.4	1.9	8.1
**Nationality status**												
Qatari	*399*	45.8	78.9	81.7	50.5	25.5	21.6	2.3	16.1	19.6	2.8	7.4
Non-Qatari	*643*	36.3	75.1	80.2	49.3	9.5	14.5	1.2	15.2	14.8	2.6	7.2
**Region**												
Doha	*520*	35.6	73.8	79.3	49.9	11.4	19.0	0.5	14.8	17.1	2.0	6.2
Al Rayyan	*371*	43.5	79.0	81.3	47.9	18.1	16.0	2.8	16.0	15.2	3.3	8.6
Other regions	*150*	46.0	80.2	84.4	53.9	24.4	15.1	2.2	16.8	19.0	2.9	7.6
**Wealth quintile**												
Poorest	*278*	43.0	78.3	82.5	53.1	14.9	16.1	0.3	16.7	17.4	2.9	5.1
< average	*230*	42.4	73.7	77.9	51.1	12.6	24.3	1.8	16.2	12.1	1.0	7.3
Average	*201*	40.6	76.7	83.5	46.4	17.1	15.2	1.9	20.2	21.3	0.7	6.4
> average	*185*	38.4	76.4	83.4	51.6	17.4	14.5	2.3	6.9	14.7	2.8	9.7
Richest	*148*	31.2	77.7	75.0	44.0	18.5	15.8	2.7	16.7	18.4	7.0	9.7
**Total**	***1042***	**39.9**	**76.5**	**80.8**	**49.8**	**15.8**	**17.3**	**1.6**	**15.5**	**16.7**	**2.6**	**7.3**

Table 5.16 Percentage of children treated for common illnesses through increasing intake of food and water

	Fever			Cough			Diarrhoea		
	Number of children	% given more liquid	% given same or more food	Number of children	% given more liquid	% given same or more food	Number of children	% given more liquid	% given same or more food
**Sex of child**									
Male	*332*	15.6	30.4	*205*	14.8	24.4	*85*	14.2	29.9
Female	*306*	16.8	28.4	*191*	14.6	33.6	*53*	10.1	22.2
**Nationality status**									
Qatari	*256*	10.6	29.1	*159*	9.4	28.6	*68*	8.4	27.5
Non-Qatari	*386*	20.0	29.5	*239*	18.7	29.0	*70*	16.8	26.4
**Region**									
Doha	*304*	22.8	31.4	*193*	21.7	31.8	*73*	16.0	30.6
Al Rayyan	*237*	6.1	25.9	*141*	7.0	29.0	*47*	5.6	20.9
Other regions	*102*	20.5	31.0	*65*	12.3	19.9	*18*	17.5	27.8
**Wealth quintile**									
Poorest	*178*	15.3	30.7	*116*	11.4	22.3	*35*	6.2	31.7
< average	*131*	16.5	27.1	*88*	17.9	35.5	*41*	21.6	34.6
Average	*129*	17.9	34.6	*71*	14.1	23.9	*23*	4.2	12.0
> average	*118*	14.8	29.1	*73*	17.4	29.0	*21*	7.1	13.2
Richest	*86*	17.4	22.4	*51*	15.7	39.1	*18*	22.4	35.5
**Total**	***643***	**16.3**	**29.3**	***398***	**15.0**	**28.9**	*138*	12.6	26.9

If the results are studied by subgroup, there was again a difference between Qatari and non-Qatari infants. About the same percentage of infants were given the same or more to eat in the two groups. However, non-Qatari infants were far more likely to have been given more to drink during their illness than infants in Qatari families. Roughly double the percentage of non-Qatari infants was given liquids compared with Qatari infants. There was also a difference by region, with those in Al Rayyan being far less likely to give their sick child more to drink compared to the other regions.

For more serious illnesses it is important that professional treatment be sought. Table 5.17 shows whether treatment was sought for three more dangerous symptoms: fast or difficult breathing, constant vomiting and if the child is not drinking or eating. If care was sought, then the place where the child first received care was recorded.

With all three symptoms, care was sought by almost all respondents. Over 99% of infants who had fast or difficult breathing went for treatment, compared with nearly 97% for constant vomiting and over 97% if the child was not eating or drinking. The most popular place to go for treatment was a hospital, closely followed by an outpatient facility such as a health centre, health post or clinic. For each of the illnesses over 80% of the children were taken to one of these two facilities for treatment. The majority of the other places where treatment was obtained were from a private physician. Private physicians were most likely to be used by Qatari households. There is also some evidence that as wealth increases treatment was obtained more from hospitals and less from health facilities.

5.9 Reproductive and sexual health care

Condom use at most recent sexual intercourse was investigated. Condoms are an effective way to stop the spread of sexually transmitted infections and to prevent unwanted pregnancy. Table 5.18 shows the percentage of those who used a condom at most recent sexual intercourse for both married and unmarried respondents.

Nearly 20% of those who are married or are living with their partner used a condom at most recent sexual intercourse. This was higher for non-nationals than nationals and also higher in Doha than other regions of Qatar. The percentage of those using a condom if they were not married was even lower, at about 10% of respondents, although the sample size for this group of people was small. All of those who were not married or cohabiting and used a condom were non-Qatari nationals, and were in the younger age groups.

Table 5.17 Percentage of children where treatment was sought for dangerous illnesses

	Fast or difficult breathing						Constant vomiting					Not drinking/eating						
	Number of children	% treatment sought	% hospital	% outpatient	% pharmacy	% private physician	Number of children	% treatment sought	% hospital	% outpatient	% private physician	Number of children	% treatment sought	% hospital	% outpatient	% pharmacy	% private physician
**Sex of child**																	
Male	*73*	99.2	45.9	35.3	0.8	18.1	*67*	100.0	47.3	42.1	10.6	*72*	96.3	44.8	33.8	0.8	19.3
Female	*53*	100.0	43.7	42.3	1.3	12.7	*56*	92.7	51.8	35.3	12.9	*62*	98.5	41.4	48.3	0.0	10.4
**Nationality status**																	
Qatari	*81*	99.3	43.9	33.9	1.5	20.6	*51*	98.0	50.8	28.7	20.5	*62*	97.5	43.0	34.7	0.9	21.4
Non-Qatari	*46*	100.0	46.8	45.9	0.0	7.3	*73*	95.7	48.2	46.5	5.3	*72*	97.2	43.3	45.7	0.0	9.6
**Region**																	
Doha	*43*	100.0	45.4	36.1	0.0	18.5	*56*	96.1	49.4	40.7	9.9	*66*	96.0	41.6	40.8	0.0	16.1
Al Rayyan	*54*	99.0	45.4	41.2	1.3	12.1	*47*	98.0	46.5	45.0	8.5	*45*	97.9	41.3	45.3	0.0	13.5
Other regions	*29*	100.0	43.4	36.1	1.9	18.7	*20*	95.0	55.6	20.7	23.7	*23*	100.0	51.3	31.0	2.4	15.3
**Wealth quintile**																	
Poorest	*33*	100.0	28.5	57.7	0.0	13.8	*36*	97.1	39.3	37.7	23.0	*32*	95.2	33.5	46.7	0.0	19.8
< average	*21*	97.3	58.5	13.8	6.1	21.7	*27*	92.1	59.1	38.5	2.4	*30*	100.0	53.5	28.0	2.6	11.3
Average	*26*	100.0	56.6	32.2	0.0	11.2	*31*	100.0	53.2	38.5	8.3	*35*	100.0	31.7	58.9	0.0	9.4
> average	*25*	100.0	48.8	42.6	0.0	8.6	*10*	100.0	32.1	56.2	11.7	*23*	96.1	53.1	32.3	0.0	14.6
Richest	*21*	100.0	38.7	34.0	0.0	27.3	*19*	95.1	57.6	34.7	7.7	*14*	95.6	58.8	21.2	0.0	20.1
**Total**	***127***	**99.6**	**44.9**	**38.3**	**1.0**	**15.8**	*123*	96.6	49.3	39.1	11.6	*135*	97.3	43.2	40.6	0.4	15.1

Table 5.18 Percentage of respondents using condom at last sexual intercour

	Currently married/ living with partner		Not married/living with partner	
	N	**% used condom last sex**	***N***	**% used condom last sex**
**Nationality status**				
Qatari	*297*	17.9	*21*	0.0
Non–Qatari	*445*	21.0	*18*	21.8
**Region**				
Doha	*350*	23.5	*20*	10.7
Al Rayyan	*277*	16.5	*16*	11.6
Other regions	*116*	16.2	*4*	0.0
**Age group**				
18 to 29	*227*	21.7	*20*	13.7
30 to 44	*495*	19.0	*17*	7.4
45 to 59	*19*	18.1	*2*	0.0
60 to 69	–	–	*1*	0.0
70 or over	*1*	0.0	–	–
**Wealth quintile**				
Poorest	*201*	15.1	*12*	3.3
< average	*166*	24.0	*5*	0.0
Average	*136*	20.2	*10*	35.8
> average	*132*	17.6	*7*	0.0
Richest	*108*	24.0	*5*	0.0
**Total**	***742***	**19.7**	*40*	10.1

6. Health state valuation

6.1 Introduction

The constitution of the World Health Organization states that "health is a state of complete physical, mental and social well-being and not merely the absence of disease or infirmity" (WHO, 1947). This principle focuses on the state of health rather than categories of disease or mortality and highlights the need to consider physical, mental and social structure in the conceptualization of health. However, this principle needs to be operationalized in order to develop indicators on these three aspects of health.

The working definition of health is expressed as the degree of conformity to an accepted standard of health for different demographic or social groups, such as those established for different ages, sexes, communities and regions, with normal limits of variation. These standards may vary between individuals, households and groups within the population in the way that some individuals may have higher expectations of health than others. However, in general, health refers to the psychological and physical functions that are essentially the attributes of individuals

The Qatar World Health Survey collected information on nine domains of health, while overall general health ratings were also investigated, encompassing all domains. The domains of health that were investigated in the survey were:

- mobility
- self-care
- pain and discomfort
- cognition
- interpersonal activities
- breathing
- sleep and energy
- affect
- vision and hearing.

The majority of these domains were investigated through two questions on the questionnaire, although some, such as self-care, pain and discomfort, interpersonal activities and vision had more questions than this. For each question on the domain the respondent was questioned about the amount of difficulty that they had with various aspects of health and were asked to rate their difficulties on a five-point scale, from none (no difficulty), mild, moderate, severe and extreme.

In order to ensure comparability of valuations across different subsections of the population (who may have different expectations of health) standardized questions regarding eight of the domains were asked. The domain of breathing was not further investigated in the questionnaire. These standardized questions took the form of vignettes. For each domain five vignettes were read to the respondent who then had to rate the health of the person in the vignette using the same scale as before, from no difficulty to extreme difficulty. For each vignette two questions were asked, mirroring the questions asked about the respondent's own health. The five vignettes covered the whole spectrum of health for that domain, ranging from someone who did not have any health problems, to someone who had extreme difficulties. The aim was to use the responses to these vignettes for different groups of the population to adjust the self-reports in order to provide a more accurate view of the health state of the population.

The responses to these vignettes did not follow the expected trends, with many respondents stating that the health of the people mentioned in all vignettes was good, even when this was obviously not correct. For example, one of the vignettes used for mobility was the following.

> Abdullah is paralysed from the neck down. He is unable to move his arms and legs or to shift body position. He is confined to bed (Q2115, Set A).

The respondents were asked "how much of a problem did Abdullah have with moving around?" The percentage of people who stated that Abdullah had no problem with moving around was 57%, while only 17% stated that he had extreme difficulty.

The vignette responses may not have followed the expected trends because the respondents misunderstood what the vignettes were about, or the interviewers did not explain the vignettes clearly enough to elicit accurate answers. The issues with the data for the vignettes means that the results are not reliable and therefore they were not used to adjust the self-rated health state descriptions as planned. The results for the vignettes are therefore not presented in this report.

6.2 General health rating

6.2.1 Overall health

Respondents were asked to rate their health on the day of the interview, from very good to very bad. Table 6.1 shows the percentages of individuals who rated themselves in the difference categories, broken down by certain demographic characteristics.

Table 6.1 Percentage distribution of general health ratings

	Very good	Good	Moderate	Bad	Very bad	*N*
**Sex**						
Male	49.3	42.0	7.7	0.8	0.2	*2414*
Female	39.2	44.6	13.6	2.3	0.3	*2359*
**Nationality status**						
Qatari	42.8	39.9	14.0	2.9	0.4	*1610*
Non-Qatari	45.1	45.0	8.9	0.8	0.2	*3163*
**Region**						
Doha	43.1	45.8	9.5	1.3	0.3	*2597*
Al Rayyan	44.5	41.9	11.4	1.8	0.4	*1470*
Other regions	48.2	37.1	12.9	1.8	0.1	*707*
**Age group**						
18 to 29	53.4	39.0	6.3	1.1	0.2	*1100*
30 to 44	44.8	43.8	10.1	1.2	0.1	*2387*
45 to 59	37.5	46.9	13.3	1.9	0.5	*1125*
60 to 69	27.8	40.5	24.3	5.6	1.9	*126*
70 or over	7.3	39.8	41.9	11.1	0.0	*35*
**Wealth quintile**						
Poorest	44.1	44.5	9.8	1.6	0.1	*1225*
< average	44.2	43.9	9.7	1.9	0.3	*1019*
Average	44.7	41.9	11.9	1.2	0.3	*1022*
> average	43.7	43.7	10.9	1.4	0.2	*838*
Richest	45.0	41.9	11.2	1.5	0.5	*670*
**Total**	**44.3**	**43.3**	**10.6**	**1.5**	**0.3**	***4773***

The majority of respondents rated their health as either very good or good, with 44.3% and 43.3% in these categories respectively. Over 10% responded that their health was moderate, while only 1.8% stated that their health was either bad or very bad. Males were much more likely to rate themselves as healthy than females, with 91.3% in the top two categories, compared with 83.8% of females. The proportion of females who stated that their health was moderate or bad was higher than the proportion of males in the same categories. Non-Qatari nationals were also more likely to rate their health as good or very good than Qatari nationals. Overall, 82.7% of Qataris fell into these categories, while 90.1% of non-Qataris stated that they had good health.

The relationship between age and self-rated health was as expected, with the highest percentage of those who said their health was very good in the youngest age group, and the lowest in the older age groups. The percentage of respondents in the moderate

health category increased from 6.3% for 18–29 year olds to 24.3% in the 60–69 age group, while the percentage of people who stated that their health was bad or very bad rose as age increased. There were minimal differences between health ratings by wealth, and there are slight differences by region. The percentage of respondents with very good self-rated health in Doha was 43.1% and in Al Rayyan 44.5%. However, in the other regions of Qatar a higher percentage of people stated that their health was very good, with 48.2% stating that this is the case.

6.2.2 Difficulty with work or household activities

A further aspect investigated with respect to the general health of the respondent was regarding difficulties with work or household activities. The respondents were asked to rate their difficulties on a scale from no difficulty to extreme difficulty or cannot do these activities. The results are shown in Table 6.2.

Almost three-quarters (72.9%) of the respondents reported that they had no difficulties with work or household activities, while 16.4% had only mild difficulties. Therefore there are 10.7% of people who report that they had either moderate, severe or extreme difficulties with these aspects of life.

Table 6.2 Percentage distribution of difficulty ratings with 'work or household activities'

	No difficulty	Mild difficulty	Moderate difficulty	Severe difficulty	Extreme/ cannot do	*N*
**Sex**						
Male	81.4	11.6	5.2	1.4	0.3	*2414*
Female	64.3	21.3	10.2	3.9	0.3	*2359*
**Nationality status**						
Qatari	66.3	19.6	9.3	4.5	0.3	*1610*
Non-Qatari	76.3	14.8	6.9	1.7	0.3	*3163*
**Region**						
Doha	73.9	15.9	7.5	2.5	0.4	*2597*
Al Rayyan	71.9	16.9	7.9	3.2	0.2	*1470*
Other regions	71.9	17.4	8.1	2.3	0.3	*707*
**Age group**						
18 to 29	78.6	13.8	5.6	1.8	0.2	*1100*
30 to 44	71.8	18.5	7.2	2.3	0.2	*2387*
45 to 59	72.7	15.1	9.1	2.7	0.3	*1125*
60 to 69	57.7	12.3	15.7	13.8	0.5	*126*
70 or over	34.4	13.4	29.5	17.7	5.1	*35*
**Wealth quintile**						
Poorest	72.1	17.6	8.0	2.0	0.3	*1225*
< average	72.6	15.9	7.1	4.0	0.4	*1019*
Average	72.5	16.5	8.6	2.2	0.3	*1022*
> average	74.6	16.4	6.5	2.4	0.1	*838*
Richest	73.7	15.0	8.1	2.8	0.4	*670*
**Total**	**72.9**	**16.4**	**7.7**	**2.7**	**0.3**	***4773***

Once again, men were more likely to report that they had no difficulty with these tasks compared with women. Over 81% of males stated that they have no difficulty, while the percentage was only 64.3% for females. There were a larger percentage of females in the mild category, with 21.3%, and over 10% state that they had moderate difficulty with work or household activities. Over three-quarters of non-Qataris had no difficulties, while about two-thirds of Qataris said the same. At each of the categories of mild, moderate, severe and extreme difficulty there was a higher percentage of Qatari than non-Qatari individuals. The relationship between age and difficulty with work or household activities was similar to that seen for overall health, with older people stating more difficulties than younger people. Those aged 18–29 had least difficulty, while those aged in the two age groups between 30–59 had a similar profile of difficulties. There are a far higher percentage of those aged over 60 with difficulties.

6.3 Mobility

Two questions were asked regarding the mobility of the individual. These related to the 30 days prior to the interview. The questions were:

- How much difficulty do you have with moving around?
- How much difficulty do you have in performing vigorous activities (such as cycling or working on the farm/*shamba*')

The first question was asked to assess whether respondents generally faced any difficulty in moving in and around their home, including increased effort, discomfort or pain, slowness or that they had had to adapt in the way they did a particular activity. This may include the use of assistive services or personal help in movement. The results for both questions on mobility are shown in Table 6.3.

6.3.1 Difficulty in moving around

The vast majority (88.5%) of respondents stated that they did not have any difficulties with moving around. The percentage of people who stated that they had mild difficulty was 6.7%, while the percentage with moderate, severe or extreme difficulty was only 4.8%. The differences between population subgroups was as for the general health rating, with males, non-Qatari nationals and the younger aged having the highest percentage of individuals reporting that they had no difficulty with moving around.

6.3.2 Difficulty with vigorous activities

Vigorous activities refer to either occupational or recreational activities, including sports such as cycling, running or aerobics, and heavy lifting, carrying or working in the fields. Fewer people stated that they had no difficulties with vigorous activities than for generally moving around, although almost three-quarters of respondents (73.7%) still stated that they no difficulty with vigorous activities. The percentage of individuals who said that they had severe difficulties was 7.1%, while 4.2% stated that they have extreme difficulty or that they could not do vigorous activities.

Table 6.3 Percentage distribution of difficulty with mobility

	Moving around						Vigorous activities					
	No difficulty	Mild Difficulty	Moderate difficulty	Severe difficulty	Extreme/ cannot do	*N*	No difficulty	Mild Difficulty	Moderate difficulty	Severe difficulty	Extreme/ cannot do	*N*
**Sex**												
Male	93.3	3.8	2.0	0.6	0.2	*2414*	82.2	7.5	4.8	3.4	2.1	*2406*
Female	83.6	9.7	4.4	1.9	0.4	*2359*	65.0	10.9	7.0	10.8	6.4	*2359*
**Nationality status**												
Qatari	82.7	9.8	4.5	2.7	0.3	*1610*	63.4	11.9	7.3	10.3	7.2	*1609*
Non-Qatari	91.5	5.1	2.5	0.6	0.3	*3163*	79.0	7.8	5.2	5.4	2.7	*3156*
**Region**												
Doha	89.6	5.8	3.3	0.9	0.4	*2597*	75.1	9.1	5.4	6.5	3.9	*2596*
Al Rayyan	87.2	7.6	3.1	1.9	0.2	*1470*	71.4	8.7	6.4	8.6	4.9	*1468*
Other regions	87.3	8.1	3.0	1.3	0.2	*707*	73.4	10.4	6.4	5.8	4.0	*701*
**Age group**												
18 to 29	92.1	5.0	2.3	0.6	0.0	*1100*	80.8	6.7	4.5	5.0	3.0	*1100*
30 to 44	89.3	6.6	2.9	1.1	0.2	*2387*	74.2	9.4	5.9	6.5	4.0	*2383*
45 to 59	87.8	6.9	3.6	1.4	0.3	*1125*	70.4	10.4	6.7	8.9	3.7	*1120*
60 to 69	62.4	18.4	10.0	4.7	4.5	*126*	48.5	15.0	6.0	15.1	15.4	*126*
70 or over	41.8	23.9	15.7	18.7	0.0	*35*	14.0	11.2	19.7	20.8	34.3	*35*
**Wealth quintile**												
Poorest	89.5	6.5	2.9	0.9	0.2	*1225*	75.0	8.3	7.0	6.3	3.4	*1224*
< average	86.5	7.6	3.4	2.0	0.4	*1019*	72.1	9.7	6.2	7.5	4.5	*1016*
Average	89.4	6.4	3.1	0.8	0.3	*1022*	75.0	9.3	4.5	7.5	3.7	*1020*
> average	88.7	7.3	2.7	1.2	0.2	*838*	71.9	10.1	5.4	8.2	4.3	*835*
Richest	88.2	5.7	4.3	1.6	0.3	*670*	74.1	8.7	5.8	5.8	5.7	*670*
**Total**	**88.5**	**6.7**	**3.2**	**1.3**	0.3	***4773***	73.7	9.2	5.9	7.1	4.2	***4765***

Almost a quarter of women (24.2%) stated that they had moderate, severe or extreme difficulties with vigorous activities. This can be compared with only 10.3% of males. There was a large difference in the percentage of individuals who said that they had no difficulty by sex, with 82.2% of males and 65% of females in this category. There was also a large difference between Qatari and non-Qatari nationals, albeit a smaller one than seen between males and females. Over 7% of Qataris reported that they had extreme difficulty with vigorous activities, while only 2.7% of non-Qataris said the same thing. Age and vigorous activities, as would be expected, also indicated a relationship. For each increasing age group the proportion of respondents with no difficulty fell, while the proportion in all other categories of difficulty rose. In the oldest age group of 70 years or more, only 14% of respondents had no difficulties, while 34.3% have extreme difficulties.

6.4 Self-care

Three questions were asked about the ability of individuals to care for themselves in the 30 days prior to the interview. These are:

- How much difficulty did you have with self-care, such as washing or dressing yourself?
- How much difficulty did you have in taking care of and maintaining your general appearance (e.g. grooming, looking neat and tidy)?
- How much difficulty did you have in staying by yourself for a few days (3 to 7 days)?

These questions were designed to obtain information about a wide range of activities such as washing and dressing which include a large amount of dexterity and upper and lower body movement. The results for the first two questions, on self-care and maintaining appearance, are shown in Table 6.4.

6.4.1 Self-care (washing and dressing)

Nineteen out of 20 respondents (95.2%) to the survey said that they had no difficulty at all regarding washing or dressing. In comparison, 1.7% stated that they had extreme difficulties or that they could not wash or dress themselves. This high percentage of respondents reporting that they had no problems may indicate an issue with the data quality or simply reflect that very few people in Qatar have problems with these tasks.

There were sex differences relating to this aspect of self-care, with 96.8% of males reporting no difficulties with washing or dressing, while 93.6% of females stated the same. The main difference between males and females was in those that had mild or moderate difficulties, with a higher proportion of females in these groups. The proportion of people in the severe or extreme difficulty categories was similar irrespective of sex. Differences between Qatari and non-Qatari nationals indicated that non-Qataris reported less difficulty than Qataris. Age was related to the ability to wash or dress, with the proportion of people with severe or extreme difficulties increasing as age rose. Finally, there was some evidence that the percentage of individuals who have extreme difficulty fell as wealth increased.

Table 6.4. Percentage distribution of difficulty with self-care

	Washing or dressing						Care and Maintenance					
	**No difficulty**	**Mild difficulty**	**Moderate difficulty**	**Severe difficulty**	**Extreme/ cannot do**	***N***	**No difficulty**	**Mild difficulty**	**Moderate difficulty**	**Severe difficulty**	**Extreme/ cannot do**	***N***
**Sex**												
Male	96.8	1.1	0.2	0.2	1.7	*2414*	96.3	1.4	0.4	0.2	1.7	*2414*
Female	93.6	3.0	1.5	0.3	1.7	*2359*	92.4	3.9	1.7	0.5	1.5	*2357*
**Nationality status**												
Qatari	91.6	3.2	1.6	0.5	3.1	*1610*	90.3	4.2	1.9	0.5	3.1	*1610*
Non-Qatari	97.0	1.4	0.4	0.1	1.0	*3163*	96.5	1.9	0.6	0.3	0.8	*3161*
**Region**												
Doha	96.5	1.5	0.6	0.2	1.2	*2597*	95.7	2.2	0.7	0.4	1.0	*2597*
Al Rayyan	93.8	3.1	1.3	0.4	1.5	*1470*	93.0	3.6	1.6	0.4	1.4	*1470*
Other regions	93.2	1.7	0.8	0.2	4.0	*707*	92.3	2.2	0.9	0.3	4.3	*705*
**Age group**												
18 to 29	95.6	1.3	1.2	0.1	1.9	*1100*	94.9	2.1	0.8	0.3	1.9	*1100*
30 to 44	95.9	2.0	0.4	0.2	1.6	*2387*	95.1	2.3	0.6	0.3	1.6	*2385*
45 to 59	95.2	2.1	0.9	0.3	1.6	*1125*	94.7	2.5	1.1	0.4	1.3	*1125*
60 to 69	84.6	7.1	4.7	1.4	2.3	*126*	79.9	10.2	6.7	1.0	2.3	*126*
70 or over	75.7	10.5	7.9	3.4	2.4	*35*	67.2	17.2	8.1	5.0	2.4	*35*
**Wealth quintile**												
Poorest	95.1	2.1	0.6	0.2	2.0	*1225*	94.2	2.3	1.1	0.6	1.8	*1225*
< average	94.1	2.0	1.6	0.1	2.2	*1019*	93.4	2.7	1.2	0.4	2.2	*1017*
Average	95.9	1.9	0.3	0.2	1.7	*1022*	95.3	2.2	0.5	0.3	1.6	*1022*
> average	95.5	2.4	0.8	0.3	1.0	*838*	93.9	3.9	1.2	0.1	0.9	*838*
Richest	95.7	1.6	1.0	0.4	1.3	*670*	95.3	2.1	1.0	0.2	1.3	*670*
**Total**	**95.2**	**2.0**	**0.9**	**0.2**	**1.7**	***4773***	94.4	2.6	1.0	0.4	1.6	*4771*

6.4.2 Taking care of and maintaining general appearance

The results for difficulties with taking care of and maintaining general appearance are very similar to the results obtained for washing and dressing. Nearly 95% of respondents stated that they had no problems with this aspect of care, while 1.6% said that they had extreme difficulties or could not do it themselves. Females encountered more difficulties than males, in general, while non-Qataris again stated fewer difficulties than Qatar nationals. There was no great difference between the percentages in each of the categories for the youngest three age groups (18–59), indicating that ability to maintain general appearance did not fall in general until over the age of 60.

6.5 Pain and discomfort

For this domain three questions were asked again to obtain information regarding the amount of pain and discomfort that individuals had suffered from in the 30 days prior to the interview. These were:

- How often do you have bodily aches or pains?
- How often do you have bodily discomfort?
- How much difficulty have you had in your daily life because of your pain?

These questions are designed to assess the difficulties that people experience that may affect their usual activities for either a short or long period of time. The first two questions, on bodily aches and pains and discomfort, were analysed, and the results shown in Table 6.5.

6.5.1 Bodily aches and pains

Two-thirds of the respondents reported that they had no aches and pains in the 30 days before the survey, while 18.6% reported that they suffered from pain rarely. The percentage of those who sometimes suffered from bodily aches and pains was 10.1%. Fewer than 5% of respondents stated that they often or always had aches and pains. Males were more likely than females to state that they never had any aches or pains, with over three-quarters of males saying this compared to only 55.8% of females. There was a large disparity between Qatari and non-Qatari nationals. 54% of Qataris stated that they never had any pain, compared with 72.8% of non-Qataris. The percentage of Qataris who often or always felt pain was 8.4%, nearly three times the percentage for non-Qataris in the same categories, which totalled 3%. Age showed the relationship seen in previous questions, with older adults reporting pain more often than younger adults, although there was not a large difference between the three age groups 18–29, 30–44 and 45–59. There was also a difference by wealth quintile, with the percentage of those who felt bodily aches and pains often or all the time increasing as wealth increased. The difference between the poorest and richest quintiles was not large, but a trend was evident as wealth increased.

Table 6.5 Percentage distribution of difficulty with pain and discomfort

	Aches and pains						Bodily discomfort					
	**None**	**Rarely**	**Sometimes**	**Often**	**Always**	***N***	**None**	**Rarely**	**Sometimes**	**Often**	**Always**	***N***
**Sex**												
Male	76.9	14.1	6.6	1.9	0.4	*2413*	76.8	14.4	6.3	2.2	0.3	*2414*
Female	55.8	23.1	13.7	6.6	0.9	*2359*	56.4	23.5	13.8	5.8	0.5	*2358*
**Nationality status**												
Qatari	54.0	24.3	13.3	7.2	1.2	*1610*	54.5	24.9	13.4	6.6	0.6	*1610*
Non-Qatari	72.8	15.6	8.5	2.7	0.3	*3163*	72.9	15.9	8.3	2.7	0.3	*3161*
**Region**												
Doha	67.6	17.8	9.9	4.1	0.7	*2596*	66.7	19.1	10.1	3.7	0.5	*2597*
Al Rayyan	66.4	18.3	10.1	4.3	0.7	*1470*	67.4	18.1	9.8	4.4	0.3	*1468*
Other regions	62.5	21.8	11.1	4.4	0.2	*707*	65.4	20.1	10.3	4.1	0.1	*707*
**Age group**												
18 to 29	73.8	14.4	7.9	3.4	0.5	*1100*	73.5	15.5	7.4	3.2	0.4	*1100*
30 to 44	64.6	20.5	10.3	4.1	0.6	*2386*	64.9	20.3	10.4	4.1	0.4	*2385*
45 to 59	67.1	17.4	10.3	4.5	0.7	*1125*	67.5	18.4	10.0	3.9	0.3	*1125*
60 to 69	43.0	28.4	18.6	8.9	1.2	*126*	45.3	27.6	19.6	6.9	0.7	*126*
70 or over	27.7	20.1	34.2	14.5	3.4	*35*	29.9	19.4	33.7	17.0	0.0	*35*
**Wealth quintile**												
Poorest	68.6	17.2	9.7	4.0	0.4	*1225*	69.6	16.9	10.3	3.1	0.1	*1224*
< average	64.4	20.6	10.3	4.1	0.6	*1018*	64.3	20.1	9.8	5.4	0.4	*1019*
Average	68.1	16.4	10.8	4.2	0.6	*1022*	67.7	18.4	10.1	3.6	0.3	*1022*
> average	66.4	19.6	9.1	4.2	0.7	*838*	67.7	19.0	9.3	3.5	0.5	*838*
Richest	63.3	20.1	10.8	4.9	1.0	*670*	62.4	21.5	10.5	4.8	0.9	*670*
**Total**	**66.5**	**18.6**	**10.1**	**4.2**	**0.6**	***4773***	66.7	18.9	10.0	4.0	0.4	*4772*

6.5.2 Bodily discomfort

The results for the frequency of experiencing bodily discomfort, shown in Table 6.5, are extremely similar to the results obtained for the frequency of experiencing bodily aches or pains. The differences between groups are of a similar magnitude. To summarize, about two-thirds of the respondents stated that they had no bodily discomfort, while 18.9% experienced discomfort rarely. The percentage of people experiencing bodily discomfort often or always was 4.4%. Women, Qatari nationals and older adults had smaller percentages stating that they never experienced discomfort compared to men, non-Qatari nationals and younger adults. However, there was no clear relationship between wealth quintile and bodily discomfort.

6.6 Cognition

Cognition relates to the abilities of the respondents in concentrating or remembering things associated with tasks, such as reading, writing, drawing or listening to others. It also refers to how well a person can learn something that is new to them. Two questions were asked about this on the questionnaire regarding the 30 days prior to the survey date. These were:

- How much difficulty did you have with concentrating or remembering things?
- How much difficulty did you have in learning a new task (for example learning how to get to a new place, learning a new game or learning a new recipe)?

The results for the responses to these two cognition questions are displayed in Table 6.6.

6.6.1 Concentrating or remembering

Over 80% of those surveyed stated that they had no difficulty at all with concentrating or remembering things, with just over 12% saying that they had mild difficulties doing these actions. Therefore there were only 7.6% of respondents who stated that they had moderate, severe or extreme difficulties in concentration or remembering. The same differences between groups were seen as in other domains. As age increased the percentage of people who said that they have any sort of difficulties also increases. For the 18–29 age group, 16% stated that they had some sort of problem with remembering or concentrating (ranging from mild to severe problems) compared with 37% of 60–69 year olds, ranging from mild to extreme difficulties. Interestingly, the percentage of people with no difficulties on these tasks was higher in Doha (82.8%) compared with Al Rayyan (77.4%) and the other areas of Qatar (76.8%). A higher percentage of males stated that they had no difficulties compared to females (85.3% compared with 75.1% respectively) while non-Qataris again gave better assessments of their own cognition than Qataris.

Table 6.6 Percentage distribution with difficulty with cognition

	Concentrating or remembering						Learning a new task					
	No difficulty	Mild difficulty	Moderate difficulty	Severe difficulty	Extreme/ cannot do	*N*	No difficulty	Mild difficulty	Moderate difficulty	Severe difficulty	Extreme/ cannot do	*N*
**Sex**												
Male	85.3	10.4	3.8	0.6	0.0	*2414*	89.9	6.5	2.6	0.8	0.1	*2414*
Female	75.1	13.9	8.1	2.7	0.1	*2359*	83.1	9.7	4.6	2.3	0.2	*2359*
**Nationality status**												
Qatari	68.6	17.8	9.8	3.6	0.2	*1610*	77.8	12.3	6.3	3.2	0.4	*1610*
Non-Qatari	86.2	9.2	4.0	0.6	0.0	*3163*	91.0	6.0	2.2	0.7	0.0	*3163*
**Region**												
Doha	82.8	11.1	5.0	0.9	0.1	*2597*	89.3	6.9	2.3	1.4	0.1	*2597*
Al Rayyan	77.4	13.1	6.9	2.6	0.0	*1470*	84.4	8.8	4.5	2.2	0.1	*1470*
Other regions	76.8	13.8	7.2	2.2	0.1	*707*	81.1	11.4	6.3	0.9	0.4	*707*
**Age group**												
18 to 29	84.0	11.2	3.8	1.0	0.0	*1100*	89.4	7.1	2.5	1.0	0.1	*1100*
30 to 44	80.6	11.5	6.1	1.7	0.1	*2387*	88.0	7.5	3.1	1.3	0.1	*2387*
45 to 59	78.8	12.9	6.7	1.5	0.1	*1125*	84.8	9.2	4.4	1.3	0.2	*1125*
60 to 69	63.0	22.5	10.8	3.7	0.0	*126*	64.5	16.6	10.1	7.9	0.9	*126*
70 or over	49.5	19.3	19.7	9.9	1.6	*35*	36.3	17.3	19.6	21.9	4.9	*35*
**Wealth quintile**												
Poorest	80.5	11.9	5.8	1.8	0.1	*1225*	86.1	7.9	3.8	2.0	0.2	*1225*
< average	79.2	13.5	5.2	2.1	0.1	*1019*	85.8	9.8	3.0	1.4	0.0	*1019*
Average	80.4	11.4	6.7	1.5	0.0	*1022*	87.1	7.0	4.1	1.6	0.1	*1022*
> average	81.9	11.7	5.1	1.3	0.1	*838*	87.3	8.3	3.3	1.0	0.1	*838*
Richest	79.2	12.3	7.2	1.2	0.2	*670*	86.9	7.4	3.7	1.5	0.5	*670*
**Total**	**80.3**	**12.1**	**5.9**	**1.6**	**0.1**	***4773***	86.6	8.1	3.6	1.6	0.2	*4773*

6.6.2 Learning a new task

Compared to the results for concentrating and remembering, a higher percentage of respondents stated that they had no difficulties in learning a new task. Over all respondents, 86.6% replied that there were no problems. Less than 10% stated that they had mild difficulties, while 3.6% had moderate difficulties and less than 2% replied that they had severe or extreme difficulties.

There is not as large a difference between sexes for this domain, as seen in previous domains, although a higher percentage of males are in the no difficulties category. Just under 90% of males had no difficulties, compared with 83.1% of females. However, the difference between Qatari nationals and non-Qatari nationals was of the same magnitude as seen in other domains, with 91% of non-Qataris having no difficulties, compared with 77.8% of Qataris. A higher percentage of Qataris had mild, moderate, severe and extreme difficulties compared to non-Qataris. The self-reported ability to learn new tasks fell markedly for the over 60 year olds. Finally, there were again regional differences, with Doha having the highest percentage of people reporting no difficulties (89.3%), compared to Al Rayyan (84.4%) and the other areas of Qatar (81.1%).

6.7 Interpersonal activities

An individual's ability to cope with interpersonal relationships is a further domain of health that was assessed in the Qatar World Health Survey. This includes ascertaining how much of an active role the respondents play in maintaining personal relationships and also in community activities. A further aspect is how well the individuals are able to deal with conflicts and tensions in personal relationships, including partners, relatives and friends. Meeting new people and making new friends, as well as dealing with strangers, are further dimensions of interpersonal activities that can be investigated.

Four questions were asked about interpersonal relationships in the 30 days before the survey. They were:

- How much difficulty did you have with personal relationships or participation in the community?
- How much difficulty did you have in dealing with conflicts and tensions with others?
- How much difficulty did you have with making new friendships or maintaining current friendships?
- How much difficulty did you have with dealing with strangers?

Responses to the first two questions were analysed, and the results are shown in Table 6.7.

6.7.1 Personal relationships and participation in the community

There was a high percentage of respondents who state that they have no difficulty with personal relationship or community participation. Over 90% stated that they had no problems, with almost 6% reporting only mild difficulties. Very few people answered that they had moderate, severe or extreme difficulties: only 3.7% of respondents stated one of these responses. Differences between population subgroups were small, albeit in the same direction as expected. A lower percentage of females and Qataris had no problems with personal relationships or participation in the community than males and non-Qataris, while difficulties increase with age.

Table 6.7 Percentage distribution of difficulty with interpersonal activities

	Personal relationships and community participation						Dealing with conflicts and tensions					
	No difficulty	Mild difficulty	Moderate difficulty	Severe difficulty	Extreme/ cannot do	*N*	No difficulty	Mild difficulty	Moderate difficulty	Severe difficulty	Extreme/ cannot do	*N*
**Sex**												
Male	92.9	4.9	1.7	0.5	0.0	*2414*	81.9	11.7	5.0	1.4	0.1	*2414*
Female	87.8	6.9	3.6	1.5	0.2	*2359*	72.0	15.9	8.4	3.5	0.2	*2359*
**Nationality status**												
Qatari	87.4	6.8	3.9	1.9	0.1	*1610*	68.1	17.0	10.0	4.6	0.3	*1610*
Non-Qatari	91.9	5.4	2.0	0.6	0.1	*3163*	81.6	12.1	5.0	1.3	0.1	*3163*
**Region**												
Doha	90.8	5.7	2.5	0.8	0.2	*2597*	78.6	13.0	6.5	1.9	0.1	*2597*
Al Rayyan	89.5	5.9	3.1	1.4	0.0	*1470*	74.8	14.3	7.1	3.7	0.2	*1470*
Other regions	90.4	6.4	2.0	1.2	0.0	*707*	75.9	15.6	6.6	1.8	0.1	*707*
**Age group**												
18 to 29	90.0	6.1	2.7	1.2	0.0	*1100*	75.1	15.2	6.6	3.1	0.0	*1100*
30 to 44	91.2	5.6	2.4	0.7	0.2	*2387*	77.2	13.7	6.8	2.1	0.2	*2387*
45 to 59	91.1	5.1	2.5	1.3	0.1	*1125*	79.8	12.3	5.8	2.1	0.1	*1125*
60 to 69	80.3	12.7	5.9	1.1	0.0	*126*	72.3	13.6	10.4	3.7	0.0	*126*
70 or over	61.7	17.7	14.1	6.5	0.0	*35*	55.7	20.0	17.7	6.7	0.0	*35*
**Wealth quintile**												
Poorest	91.0	5.4	2.3	1.2	0.2	*1225*	76.6	13.9	7.0	2.4	0.1	*1225*
< average	89.8	6.0	3.0	1.1	0.2	*1019*	77.2	13.5	6.3	2.9	0.2	*1019*
Average	90.5	5.7	3.0	0.8	0.1	*1022*	78.3	12.4	6.6	2.6	0.1	*1022*
> average	91.5	5.8	1.9	0.7	0.1	*838*	77.0	13.4	8.0	1.6	0.1	*838*
Richest	88.3	7.1	3.2	1.4	0.0	*670*	75.6	16.5	5.2	2.4	0.3	*670*
**Total**	90.4	5.9	2.6	1.0	0.1	***4773***	77.0	13.8	6.7	2.4	0.1	*4773*

6.7.2 Conflicts and tensions

Across all respondents to the survey, 77% replied that they did not have any difficulties with dealing with conflicts and tensions, while 13.8% stated that they had mild difficulties with this aspect of interpersonal activities. A small percentage (0.1%) reported extreme difficulties, and 6.7% and 2.4% stated moderate and severe problems respectively. The percentages of people in the different difficulty categories over the different age groups did not vary as much as seen in other domains of health. There was a small decrease in the percentage of people with no difficulties in the 60–69 age group compared to the 45–59 age group, although there was a large rise in the proportion of people with moderate difficulties. Once again, males were less likely to have difficulties than females, with 6.5% having moderate difficulties or worse, while 12.1% of females were in the same three categories. The differences between the two nationality status groups remained the same as previous results have indicated, with Qataris reporting more difficulties than non-Qataris. For Qataris, nearly 32% of respondents stated that they had at least a mild difficulty with dealing with conflicts and tensions, compared with 18.4% of non-Qataris.

6.8 Vision

An important facet of health is related to vision and hearing. The next two sections in this chapter relate to these areas. For vision, respondents were asked to state whether they used glasses or contact lenses to be able to see either short or long distances. After this, the respondents were asked about difficulties they may have seeing objects at distances and also with difficulties seeing objects at arm's length. Both of these questions were asked with the assumption that the answer would be given as if the respondent was wearing glasses or contact lenses, if needed, when looking at these objects. The two specific questions asked were:

- How much difficulty did you have in seeing and recognizing a person or object you know across the road (from a distance of about 20 metres)?
- How much difficulty did you have in seeing and recognizing an object at arm's length (for example reading)?

Table 6.8 presents the results for both questions and whether the individual wears glasses or contact lenses.

6.8.1 Need for glasses or contact lenses

More respondents needed glasses or contact lenses for distances than for seeing things close up, with 28% needing glasses for distance compared with 22.8% needing them for close up objects. More males than females needed either type of glasses or contact lenses, while non-Qatari nationals were more likely to wear glasses or contact lenses. For long distances, 28.9% of non-Qataris needed glasses compared to 26.2% of Qatari nationals, while for short distances 26% of non-Qataris needed glasses, while only 19.6% of Qataris did. Use is highest in Doha, with the other regions of Qatar and Al Rayyan having very similar percentages. As would be expected, use of vision aids increased with age, with the biggest increase being in the need for glasses for short distances.

Table 6.8 Percentage distribution of difficulty with vision

	Need glasses?		Difficulty in seeing and recognizing a person across road						Difficulty in seeing and recognizing an object at arms length					
	**For far away?**	**For up close?**	**No difficulty**	**Mild difficulty**	**Moderate difficulty**	**Severe difficulty**	**Extreme/ cannot do**	***N***	**No difficulty**	**Mild difficulty**	**Moderate difficulty**	**Severe difficulty**	**Extreme/ cannot do**	***N***
**Sex**														
Male	29.9	26.0	82.3	11.7	4.0	1.8	0.1	*2414*	83.5	10.5	4.4	1.4	0.2	*2410*
Female	25.9	19.6	80.3	11.0	4.8	3.7	0.2	*2359*	81.5	11.1	4.3	2.9	0.2	*2359*
**Nationality status**														
Qatari	26.2	19.6	78.2	11.4	5.4	4.5	0.4	*1610*	79.7	11.8	4.6	3.5	0.4	*1610*
Non-Qatari	28.9	24.5	82.9	11.3	3.9	1.8	0.0	*3163*	84.0	10.3	4.2	1.4	0.1	*3159*
**Region**														
Doha	30.0	25.7	81.5	11.5	4.1	2.7	0.2	*2597*	82.4	10.5	4.6	2.2	0.3	*2593*
Al Rayyan	25.6	20.4	82.0	10.8	4.2	2.8	0.2	*1470*	82.4	11.1	4.1	2.2	0.2	*1470*
Other regions	25.2	20.4	79.3	12.3	5.7	2.7	0.0	*707*	83.3	11.0	4.0	1.8	0.0	*707*
**Age group**														
18 to 29	23.9	11.4	82.5	10.1	4.0	3.2	0.1	*1100*	89.9	7.4	1.4	1.2	0.1	*1100*
30 to 44	23.5	16.9	85.1	9.0	3.7	2.1	0.1	*2387*	87.1	8.5	2.9	1.5	0.0	*2387*
45 to 59	38.7	43.4	75.7	16.6	5.1	2.2	0.4	*1125*	70.3	16.9	9.0	3.4	0.6	*1121*
60 to 69	46.2	45.7	57.6	19.4	13.2	9.8	0.0	*126*	49.9	26.6	14.7	8.2	0.6	*126*
70 or over	47.1	45.3	50.0	19.0	9.7	21.3	0.0	*35*	50.5	20.6	11.1	14.4	3.4	*35*
**Wealth quintile**														
Poorest	27.6	21.7	81.2	12.3	3.8	2.5	0.2	*1225*	83.5	11.7	3.4	1.2	0.2	*1223*
< average	26.1	20.8	83.5	9.4	4.4	2.4	0.2	*1019*	83.3	9.9	4.8	1.9	0.1	*1019*
Average	27.4	23.4	81.7	10.8	4.8	2.7	0.1	*1022*	81.7	10.4	5.4	2.4	0.2	*1022*
> average	30.1	24.5	80.0	11.8	4.5	3.5	0.2	*838*	84.1	8.9	4.1	2.5	0.3	*838*
Richest	29.7	25.1	79.3	13.1	4.7	2.8	0.2	*670*	78.8	13.5	4.2	3.3	0.2	*669*
**Total**	**28.0**	**22.8**	**81.3**	**11.4**	**4.4**	**2.8**	**0.2**	***4773***	82.5	10.8	4.4	2.1	0.2	***4769***

6.8.2 *Seeing and recognizing someone across a road*

The results for how difficult the respondents find seeing and recognizing a person or an object across the road are shown in Table 6.8. Over four in five people (81.3%) stated that they had no difficulty in seeing someone over the road, while 11.4% stated that they had mild difficulty in doing this. Very few people reported severe or extreme difficulties, even if they were wearingtheir glasses or contact lenses, with 2.8% and 0.2% falling in these two categories respectively. These results need to be interpreted with the knowledge that the question was answered with the assumption that the respondent was wearing glasses or contact lenses, if needed.

Differences in difficulties between population groups were smaller than those seen in previous health states. The main differences were seen between age categories, with the ability to see and object or recognize a person across a road decreasing as age increased. Over 80% of those in the 18–29 and 30–44 age groups had no difficulty in seeing long distances, while the corresponding value for 60–69 year olds is 57.6%. In this older age group 19.4% of respondents stated that they had mild difficulties while 13.2% had moderate difficulties. A higher percentage of males than females and non-Qataris than Qataris said that they did not have any difficulties, although as already stated, the differences between these groups was not large.

6.8.3 *Seeing and recognizing an object at arm's length*

-The percentages of people who had difficulties with seeing an object at arms length were very similar to the percentages who reported difficulties with seeing an object at a distance. The percentage of people who reported no difficulties was 82.5%, with 10.8% reporting mild difficulties. The percentage of people with severe or extreme difficulties totalled 2.3%. Once again, the highest percentages of those with some sort of difficulty were females and Qatari nationals, with 18.5% and 20.3% with difficulties respectively. This contrasts with males and non-Qatari nationals, with 16.5% and 16% respectively. Difficulties were more common as age increases, with only about 50% of those in the top two age groups saying that they did not have any difficulties at all.

6.9 Hearing

The health state description section of the questionnaire also asked the respondents to state whether they wore a hearing aid. The respondents were also asked certain questions about their hearing. Two questions were devoted to hearing. These were:

- How much difficulty did you have in hearing someone talking on the other side of the room in a normal voice (even with your hearing aid on, if you use one)?
- How much difficulty did you have in hearing what is said in a conversation with one other person in a quiet room (even with your hearing aid on, if you use one)?

The results for hearing are displayed in Table 6.9.

6.9.1 *Wearing hearing aids*

Across all respondents, 4.4% reported that they wear a hearing aid. There were 4.9% of the males in the survey who stated that they wore an aid, compared with 4% of females. There was also a difference between Qataris and non-Qataris, with 5.7% of the former wearing an aid compared with only 3.8% of the latter. Use did not vary greatly by age, with 5.5% wearing a hearing aid in the 18–29 age group, 3.7% in the 30–44 age group and 4.5% of the 45–59 age group. The percentage was highest in the 60–69 age group, where 8.1% wear an aid.

Table 6.9 Percentage distribution of difficulty with hearing

	Wear hearing aid?	Difficulty in hearing someone talking on other side of room						Difficulty in hearing conversation in a *Quiet Room*					
		No difficulty	Mild difficulty	Moderate difficulty	Severe difficulty	Extreme/ cannot do	*N*	No difficulty	Mild difficulty	Moderate difficulty	Severe difficulty	Extreme/ cannot do	*N*
**Sex**													
Male	4.9	93.9	3.7	2.0	0.4	0.1	*2414*	94.9	2.8	1.9	0.4	0.1	*2412*
Female	4.0	93.6	3.9	1.9	0.5	0.1	*2359*	94.0	3.6	1.7	0.6	0.1	*2359*
**Nationality status**													
Qatari	5.7	90.3	5.7	2.8	0.9	0.3	*1610*	91.2	5.2	2.2	1.3	0.2	*1610*
Non-Qatari	3.8	95.5	2.8	1.5	0.2	0.0	*3163*	96.1	2.2	1.5	0.1	0.1	*3160*
**Region**													
Doha	4.3	94.8	3.6	1.4	0.1	0.1	*2597*	95.4	2.9	1.4	0.2	0.1	*2594*
Al Rayyan	4.5	93.2	4.2	1.9	0.6	0.1	*1470*	93.2	4.0	2.1	0.7	0.1	*1470*
Other regions	4.8	91.0	3.7	4.0	1.3	0.1	*707*	93.6	2.5	2.7	1.1	0.1	*707*
**Age group**													
18 to 29	5.5	95.8	2.9	1.0	0.2	0.1	*1100*	95.7	2.5	1.3	0.4	0.1	*1100*
30 to 44	3.7	94.8	3.1	1.6	0.4	0.1	*2387*	95.6	2.6	1.3	0.4	0.1	*2387*
45 to 59	4.5	93.0	4.6	1.9	0.5	0.0	*1125*	93.6	4.0	1.9	0.5	0.0	*1123*
60 to 69	8.1	72.7	13.1	12.6	1.3	0.4	*126*	79.4	9.4	9.9	0.9	0.4	*126*
70 or over	5.7	57.5	15.1	14.8	9.1	3.4	*35*	55.4	17.6	14.9	8.7	3.4	*35*
**Wealth quintile**													
Poorest	4.8	93.0	4.1	2.5	0.3	0.1	*1225*	93.7	3.0	2.3	0.7	0.3	*1223*
< average	4.6	94.6	3.1	1.9	0.5	0.0	*1019*	94.8	2.9	1.7	0.7	0.0	*1019*
Average	3.1	93.5	3.9	2.1	0.5	0.1	*1022*	94.7	3.0	1.9	0.4	0.0	*1022*
> average	4.6	94.8	3.2	1.1	0.7	0.2	*838*	95.6	2.9	1.1	0.3	0.1	*838*
Richest	5.5	92.9	4.8	2.0	0.2	0.1	*670*	93.6	4.5	1.5	0.3	0.1	*670*
**Total**	**4.4**	**93.7**	**3.8**	**1.9**	**0.4**	**0.1**	***4773***	**94.5**	**3.2**	**1.8**	**0.5**	**0.1**	***4771***

6.9.2 Hearing someone on the other side of the room

Table 6.9 shows the distribution of results for hearing someone talking on the other side of a room. Most respondents stated that there was no difficulty with this, with 93.7% falling in this category. Only 3.8% said that they had mild difficulty while only 2.4% were in the three categories of moderate, severe or extreme difficulty. Differences between population groups were seen between Qataris and non-Qataris, with 95.5% of non-Qataris stating that they had no difficulties in hearing someone talking on the other side of a room, compared with 90.3% of Qataris. There are also differences between regions, with fewer difficulties with hearing reported in Doha and Al Rayyan than reported in the other regions of Qatar. In these other regions 4% of respondents stated that they had moderate problems with hearing, compared with 1.4% and 1.9% in Doha and Al Rayyan respectively. As anticipated, hearing ability decreased with age, with a higher percentage of people reporting hearing difficulties after the age of 60 than before.

6.9.3 Hearing a conversation in a quiet room

The percentage of people who reported difficulties in hearing what was said in a conversation with one other person is 5.5%. Most of these reported only mild difficulties, while 2.4% reported moderate, severe or extreme hearing difficulties. Once again a higher percentage of Qatari nationals reported difficulties than non-Qataris; 8.8% of Qataris had some sort of difficulty compared to 3.9% of non-Qataris. The relationship between age and hearing conversation was similar to that seen for hearing talking on the other side of a room, with few difficulties for younger adults and more difficulties for older adults.

6.10 Sleep and energy

Feeling rested and refreshed is important for good health. Lack of sleep can affect many areas of life and reduce functioning while awake. Reduced energy levels are one of the consequences of a lack of sleep, although it could also be a result of many other disorders. The survey asked two questions in relation to sleep and energy in the 30 days before the survey. These questions were:

- How much difficulty did you have with sleeping, such as falling asleep, waking up frequently during the night or waking up too early in the morning?
- How much difficulty did you have due to not feeling rested and refreshed during the day (e.g. feeling tired, not having energy)?

The distribution of the responses to these questions is shown in Table 6.10.

6.10.1 Sleeping

The results indicate that 78.9% of respondents did not have any difficulty associated with sleeping. Over 10% of the individuals interviewed reported mild difficulties, while 6.8% stated that they had moderate difficulties. Only 0.8% had extreme problems with sleeping, leaving 2.8% who reported severe problems.

The percentage of people with difficulty sleeping by background characteristics indicate the patterns observed in most of the health states analysed above. There were differences by sex, nationality status and age, and also varying proportions by region and wealth. Females were less likely to report that they had no difficulties with sleep than males, with 72.3% reporting no difficulties compared to 84.9% respectively. Over 13% of females reported mild difficulties, while 9.2% report moderate difficulties. This contrasts with 8.4% and 4.5% of males with mild and moderate problems respectively.

The percentage of non-Qatari individuals reporting difficulties with sleep was 15.2%. The corresponding percentage for Qatari respondents was 33.3%. The main categories where differences are seen between these two groups are in the mild, moderate and severe difficulties, where a far higher percentage of Qataris fell compared to non-Qataris. Difficulties with sleep increased with age, and it was also clear that residents of Doha reported far fewer problems with sleep that residents of Al Rayyan and other regions of Qatar.

6.10.2 Feeling rested and refreshed

The second aspect of sleep and energy was feeling rested and refreshed during the day. Table 6.10 presents the results for this, and it is seen that just over three-quarters (76.6%) of respondents said that they had no difficulties with feeling rested and refreshed. For those who did experience difficulties, 12.5% faced mild difficulties, 7.8% moderate difficulties and 2.7% severe difficulties. Only 0.3% of the respondents reported that the difficulties that they had with feeling rested and refreshed were extreme.

Once again, a higher percentage of males stated that they had no problems with this than females, with 82.8% of men and 70.4% of women reporting no difficulties. Women had higher percentages in each of the difficulty categories, from mild to extreme. The same pattern is seen by nationality status, with a higher percentage of non-Qatari nationals reporting no difficulties, while a higher percentage of Qatari nationals were seen in each of the remaining difficulty categories. The percentage of respondents in the mild to extreme problem groups increased markedly after the age of 60, although below this age there was minimal difference between the age groups.

6.11 Affect

Some people may be depressed in such a way that it interferes with their life and influences their health. The general name for such feelings is affect. This aspect of health was investigated through two questions relating to the last 30 days. The questions asked regarding affect are:

- How much of a problem did you have with feeling sad, low or depressed?
- How much of a problem did you have with worry or anxiety?

It is acknowledged that everybody worries to some extent, although it only becomes a problem when a person worries more than usual or worries excessively. Sadness can lead to someone feeling tired and losing interest in taking part in activities. Table 6.11 presents the results for the two questions relating to affect, and these results will be discussed further below.

Table 6.10 Percentage distribution of difficulty with sleep and energy

	Difficulty with sleeping						Not feeling rested					
	**No difficulty**	**Mild difficulty**	**Moderate difficulty**	**Severe difficulty**	**Extreme/ cannot do**	***N***	**No difficulty**	**Mild difficulty**	**Moderate difficulty**	**Severe difficulty**	**Extreme/ cannot do**	***N***
**Sex**												
Male	84.9	8.4	4.5	1.4	0.8	*2413*	82.8	10.3	5.4	1.3	0.2	*2413*
Female	72.3	13.4	9.2	4.2	0.9	*2359*	70.4	14.9	10.2	4.1	0.5	*2358*
**Nationality status**												
Qatari	66.7	16.2	11.3	4.6	1.1	*1609*	65.5	17.2	12.1	4.5	0.6	*1609*
Non-Qatari	84.8	8.1	4.5	1.9	0.7	*3163*	82.3	10.2	5.6	1.8	0.2	*3162*
**Region**												
Doha	81.4	9.2	5.7	2.7	1.0	*2596*	78.6	11.8	6.8	2.4	0.4	*2596*
Al Rayyan	76.4	11.3	8.1	3.5	0.8	*1470*	75.2	12.1	9.1	3.4	0.3	*1470*
Other regions	73.4	16.3	8.3	1.8	0.3	*707*	72.6	16.3	8.5	2.4	0.2	*706*
**Age group**												
18 to 29	79.0	10.9	6.7	2.9	0.6	*1099*	77.6	11.3	7.7	3.0	0.5	*1098*
30 to 44	78.8	10.7	7.0	2.7	0.8	*2387*	76.9	12.6	8.0	2.1	0.4	*2387*
45 to 59	80.3	10.5	5.5	2.6	1.1	*1125*	77.5	12.5	6.7	3.1	0.2	*1125*
60 to 69	65.4	17.8	13.3	2.5	1.1	*126*	62.1	21.6	12.9	3.4	0.0	*126*
70 or over	56.8	10.8	15.0	17.4	0.0	*35*	53.7	13.7	13.7	18.9	0.0	*35*
**Wealth quintile**												
Poorest	80.2	8.6	7.3	3.1	0.8	*1225*	79.4	9.9	7.4	2.9	0.3	*1225*
< average	76.5	12.2	7.7	2.4	1.2	*1019*	73.5	13.9	9.7	2.7	0.2	*1018*
Average	76.2	14.0	6.5	2.6	0.6	*1021*	74.9	15.1	7.0	2.7	0.3	*1021*
> average	82.3	8.3	5.8	3.3	0.3	*838*	79.2	11.9	6.3	2.4	0.2	*838*
Richest	78.4	11.3	6.5	2.5	1.4	*670*	75.8	12.2	8.7	2.6	0.8	*670*
**Total**	**78.7**	**10.9**	**6.8**	**2.8**	**0.8**	***4772***	76.6	12.5	7.8	2.7	0.3	***4771***

6.11.1 Feeling sad, low or depressed

Over a third of respondents (35.1%) stated that they had problems with feeling sad, low or depressed. The majority of these individuals with some sort of problem only felt this way “a little”, with 16.6% of all respondents falling into this category, while 13.5% of people stated that they “sometimes” felt sad or depressed. Respondents who felt sad or depressed consisted of 5% of those interviewed.

There were large differences between Qatari and non-Qatari nationals. Only about half of Qataris (51.5%) stated that they never felt sad or low, compared with 71.7% of non-Qataris. There are 17.7% of Qataris who felt this way sometimes, whereas only 11.4% of non-Qataris were in the same category. There was also a large difference between males and females, with females more likely to feel depressed than males. The percentage of females who felt sad or depressed at least a little was 42.2%, compared to 28.2% of males.

There was some evidence that the proportion of respondents who felt low or depressed at least some of the time was higher for older adults, although the differences between groups was not that large. However, between regions there was a difference. Those who live in Doha were less likely to admit to feeling sad or depressed than those living in Al Rayyan or other areas of Qatar. Those who did live in the other areas were more likely to say that they suffered from sadness a little, while residents of Al Rayyan were more likely than the other regions to state that they felt sad sometimes. The relationship between wealth and sadness was not strong, although the wealthiest quintile had the lowest percentage of respondents who stated that they never feel sad, low or depressed.

6.11.2 Worry or anxiety

Table 6.11 displays the percentage of respondents in different categories of the frequency of feeling worried or anxious. Overall, 63.2% of respondents stated that they never felt this way, while 17.9% said that they felt this way a little. Sometimes feeling worried or anxious was reported by 14.2% of people, and 3.8% stated that this happened often. The differences between groups with different background characteristics were very similar to those seen for feeling sad, low or depressed. A higher percentage of males than females had no problems with worry or anxiety, with 70.1% and 56.2% respectively, while there was still a large difference between Qatari and non-Qatari nationals on this measure. Fewer than 50% of Qataris never had these problems, while about 70% of non-Qataris felt the same way. Doha was again the region with fewest problems, with 33.9% stating a problem, compared to 40.4% in Al Rayyan and 40.3% in the other Qatari regions.

Table 6.11 Percentage distribution of difficulty with affect

	Problem with feeling sad, low or depressed						Problem with worry or anxiety					
	None	Little	Sometimes	Often	Always	*N*	None	Little	Sometimes	Often	Always	*N*
**Sex**												
Male	71.8	14.1	11.1	2.3	0.7	*2405*	70.1	15.3	11.5	2.5	0.6	*2405*
Female	57.8	19.2	16.0	5.8	1.2	*2355*	56.2	20.5	17.0	5.1	1.2	*2355*
**Nationality status**												
Qatari	51.5	22.6	17.7	6.7	1.6	*1608*	48.9	24.0	19.1	6.1	1.8	*1608*
Non-Qatari	71.7	13.6	11.4	2.7	0.6	*3152*	70.5	14.7	11.8	2.6	0.4	*3152*
**Region**												
Doha	67.2	16.1	12.5	3.4	0.9	*2592*	66.1	17.1	12.3	3.7	0.8	*2592*
Al Rayyan	62.8	15.4	16.0	4.8	1.0	*1466*	59.6	17.2	17.9	4.1	1.2	*1466*
Other regions	60.9	21.1	12.3	5.0	0.7	*702*	59.7	22.1	13.6	3.8	0.8	*702*
**Age group**												
18 to 29	66.4	15.4	12.0	5.4	0.9	*1096*	64.5	16.9	12.3	5.4	1.0	*1096*
30 to 44	63.5	17.7	14.2	3.6	1.1	*2383*	62.2	18.1	15.3	3.4	1.0	*2383*
45 to 59	66.9	16.0	12.8	3.7	0.7	*1120*	64.7	18.0	13.5	3.2	0.7	*1120*
60 to 69	63.2	13.0	19.8	2.7	1.4	*126*	61.0	16.4	18.6	3.4	0.5	*126*
70 or over	53.8	18.9	20.3	7.0	0.0	*35*	50.4	30.9	13.5	5.3	0.0	*35*
**Wealth quintile**												
Poorest	66.0	16.1	13.2	4.0	0.7	*1222*	64.3	17.5	13.6	4.0	0.6	*1222*
< average	66.3	15.5	12.7	4.2	1.3	*1014*	64.3	16.7	14.5	3.3	1.3	*1014*
Average	65.1	17.0	12.7	4.6	0.6	*1018*	63.4	18.4	12.3	5.3	0.7	*1018*
> average	64.8	15.6	15.0	3.8	0.9	*838*	63.0	16.6	16.3	3.1	1.0	*838*
Richest	60.4	20.1	14.8	3.4	1.4	*669*	59.5	21.1	15.5	2.9	1.0	*669*
**Total**	**64.9**	**16.6**	**13.5**	**4.1**	**0.9**	***4760***	63.2	17.9	14.2	3.8	0.9	***4760***

6.12 Breathing

The final aspect of health state that was asked in the Qatar World Health Survey was related to breathing. The two questions again were related to the experiences of the 30 days before the survey, and participants were asked:

- How much difficulty did you have with breathing, such as shortness of breath at rest?
- How much difficulty did you have with shortness of breath with mild exercise, such as climbing uphill for 20 metres or stairs (such as 12 steps)?

The results for this domain of health state are shown in Table 6.12.

6.12.1 Shortness of breath at rest

Only 10% of survey respondents stated that they had some problems with shortness of breath when they were resting, and out of these 60% had mild difficulty and 27% moderate difficulty with breathing. The percentage of people with difficulties in this area differed with age, with 90.8% of those aged between 18 and 44 reporting no problems, while 76.3% of 60–69 year olds stated that they could breathe easily when they were at rest. Males and non-Qataris, as usual, had lower percentages of difficulties compared to females and Qatari nationals. For Qataris, 10% reported mild difficulties with breathing at rest, while 5.1% had moderate difficulties, compared with only 3.9% and 1.5% of non-Qataris respectively. The respondents who lived in Doha were again more likely to report no difficulties with breathing than the other areas of Qatar, with only 7.8% reporting difficulties compared with 12.2% of respondents from Al Rayyan and 13.6% of those who live outside these two areas.

6.12.2 Shortness of breath after mild exercise

The percentage of people who had problems with shortness of breath after mild exercise was about double the percentage of those who had difficulties with shortness of breath at rest. Overall, 19.7% reported some difficulties, 12% reported mild difficulties and 4.5% say that they had moderate difficulties with breathing after doing a small amount of exercise. About 3% of respondents said that they have severe or extreme difficulties with this aspect of breathing.

Differentials between population subgroups are wider for this aspect of breathing than for breathing at rest, although the differences seen were in a similar direction to those observed for the other domains of health state. The percentage of males with no difficulties was 86.8%, while the percentage of females in the same category was only 73.7%, with a higher percentage of females in each of the difficulty categories. There was a 20 percentage point gap between Qataris and non-Qataris too, with 33% of Qataris reporting a problem in comparison to about 13% of non-Qataris. Age was related to shortness of breath in the expected fashion, while residents of Doha again reported fewer difficulties than those living elsewhere in Doha.

Table 6.12 Percentage distribution of difficulty with breathing

	Shortness of breath at rest						Shortness of Breath with Mild Exercise					
	**No difficulty**	**Mild difficulty**	**Moderate difficulty**	**Severe difficulty**	**Extreme/ cannot do**	***N***	**No difficulty**	**Mild difficulty**	**Moderate difficulty**	**Severe difficulty**	**Extreme/ cannot do**	***N***
**Sex**												
Male	93.5	4.0	1.9	0.4	0.2	*2413*	86.8	8.9	2.7	1.3	0.3	*2413*
Female	86.4	8.0	3.5	1.8	0.3	*2359*	73.7	15.3	6.4	3.7	1.0	*2359*
**Nationality status**												
Qatari	82.1	10.0	5.1	2.4	0.4	*1610*	67.0	19.4	7.2	5.0	1.4	*1610*
Non-Qatari	93.9	3.9	1.5	0.5	0.2	*3162*	87.1	8.3	3.2	1.2	0.2	*3162*
**Region**												
Doha	92.2	4.9	2.0	0.6	0.3	*2596*	83.3	10.2	4.0	2.0	0.5	*2596*
Al Rayyan	87.8	6.9	3.4	1.7	0.3	*1469*	76.4	14.2	5.3	3.2	0.9	*1469*
Other regions	86.4	8.0	3.8	1.7	0.1	*707*	77.3	14.5	4.9	2.6	0.7	*707*
**Age group**												
18 to 29	90.8	5.3	2.5	1.1	0.4	*1100*	83.0	11.2	3.6	1.8	0.4	*1100*
30 to 44	90.8	5.9	2.4	0.8	0.1	*2386*	81.2	11.9	4.3	2.4	0.2	*2386*
45 to 59	89.6	6.0	2.9	1.2	0.3	*1124*	79.3	12.2	5.1	2.5	0.8	*1124*
60 to 69	76.3	12.0	6.9	2.4	2.5	*126*	60.8	17.9	10.2	7.2	4.0	*126*
70 or over	68.6	6.0	9.6	15.8	0.0	*35*	34.4	20.9	17.0	10.7	17.1	*35*
**Wealth quintile**												
Poorest	91.1	4.7	2.9	0.9	0.4	*1224*	80.5	11.4	5.9	1.8	0.4	*1224*
< average	88.9	6.6	2.7	1.4	0.4	*1018*	79.5	12.0	4.9	3.3	0.4	*1018*
Average	89.5	6.1	3.0	1.1	0.3	*1022*	80.5	12.1	4.4	2.2	0.8	*1022*
> average	90.5	6.0	2.2	1.1	0.1	*838*	80.8	12.7	3.4	2.2	0.9	*838*
Richest	89.4	7.0	2.5	1.1	0.1	*670*	80.4	12.3	3.3	3.3	0.8	*670*
**Total**	**90.0**	**6.0**	**2.7**	**1.1**	**0.3**	***4772***	80.3	12.0	4.5	2.5	0.6	***4772***

6.13 Functioning assessment

The health state of the individual respondents was assessed using a tool for measuring functioning and disability. The WHO Psychiatric Disability Schedule (WHODAS) is available in a number of lengths. In the Qatar World Health Survey the short 12-item version of the tool was used.

Respondents were asked 12 questions regarding their difficulty in performing certain tasks, such as standing for long periods (such as 30 minutes), learning a new task, washing their whole body and getting dressed. Also probed were difficulties with their day-to-day work, maintaining a friendship and how much they had been emotionally affected by their health problems. The responses were recorded on a scale of 1 to 5, with 1 indicating no difficulty and 5 indicating extreme difficulty. These scores were combined using established methods to produce a WHODAS score, ranging from 0 to 100, with 0 indicating no impairment and 100 indicating complete impairment.

An important point to note is that non-response on any one of the 12 items results in the individual not having a score calculated. On analysis of the 12 questions for the WHODAS tool it was seen that one item, how much of a problem did the individual have in joining in community activities, had a large percentage of people who stated that this item was not applicable to them. In order to include these people in the final WHODAS score distribution, all those who were recorded as giving this response had their responses changed to be equal to the mean response for those who gave a valid response.

The mean WHODAS score is shown in Table 6.13. Also shown in the table is an assessment of the impact that these health difficulties investigated in the WHODAS on the individual's life. The responses to this question ranged from no impact to extreme impact.

The mean WHODAS score for all respondents was 5.6. This score is very close to 0, indicating that the average level of disability in these individuals was very low. This tool highlights the trends and relationships that were seen throughout this chapter with regard to health states. Females had over double the mean WHODAS score of males, with a figure of 7.6 for women compared to a score of 3.6 for males. Qataris also had a higher score than non-Qataris, again by about a factor of two. The younger age groups had a lower average WHODAS result than the older ages, with an average score of 22.8 for those in the 70 or over age group, compared with an average score of only 4.9 for the 18 to 29 year olds. Results by sex, nationality status and age group are shown in Figure 6.1.

The graph indicates that non-Qatari males had the lowest mean score on the WHODAS instrument, indicating the best health, and this rose with age. Qatari males and non-Qatari females were similar in their average scores, while Qatari females scored the highest, indicating the lowest level of functioning and the highest level of disability. If the mean scores are calculated for sex and nationality status together, it is observed that Qatari females had the highest mean WHODAS score, with 11.0, with Qatari males and non-Qatari females having a similar score of 5.3 and 5.4 respectively. Male non-Qataris had the lowest WHODAS score of 2.9. The mean scores for the 70 and over age group are unreliable due to very small sample sizes in this category.

Over three-quarters (77.1%) of respondents state that health difficulties, if experienced, did not interfere with their life at all. This percentage falls to 14.2% for those who said that their life has been mildly interfered with, and to 5.4% for those with moderate

interference. The percentage of those who had not experienced any interference was similar for the youngest three age groups, although there was a small increase in those who said that they had been affected by their health problems moderately or severely in these groups. However, the percentages of respondents whose life had been interfered with by these difficulties was far higher for those aged over 60, with 43% stating that their health difficulties had interfered with their life. The percentage of males who had no interference was higher that the percentage of females, while non-Qataris again had fewer people saying that their lives had been affected by health than Qataris (82.5% and 66.6% respectively).

Table 6.13 WHODAS score and percentage distribution of the effect of health difficulties on life

		Average WHODAS score	*N*	How much do health difficulties interfere with life?					
				Not at all	Mildly	Moderately	Severely	Extremely	*N*
**Sex**									
	Male	3.6	*2108*	84.9	9.7	3.1	1.7	0.6	*2268*
	Female	7.6	*2142*	69.2	18.7	7.6	3.4	1.0	*2260*
**Nationality status**									
	Qatari	8.7	*1418*	66.6	19.7	8.3	4.1	1.2	*1549*
	Non-Qatari	4.1	*2831*	82.5	11.3	3.8	1.7	0.6	*2979*
**Region**									
	Doha	5.0	*2301*	79.1	13.2	4.9	1.9	0.8	*2443*
	Al Rayyan	6.0	*1359*	76.5	14.3	5.1	3.4	0.7	*1429*
	Other regions	7.3	*591*	71.1	17.4	7.5	3.0	1.0	*656*
**Age group**									
	18 to 29	4.9	*983*	79.8	13.6	3.7	1.9	1.0	*1043*
	30 to 44	5.4	*2144*	77.8	14.0	5.3	2.1	0.8	*2259*
	45 to 59	5.6	*988*	76.7	14.4	5.2	3.1	0.6	*1069*
	60 to 69	13.6	*107*	57.0	16.8	17.0	8.3	0.8	*121*
	70 or over	22.8	*27*	38.5	26.4	22.9	10.6	1.6	*35*
**Wealth quintile**									
	Poorest	5.4	*1093*	77.7	14.5	5.1	2.3	0.5	*1159*
	< average	5.9	*899*	77.4	13.6	5.9	1.9	1.2	*969*
	Average	5.6	*914*	77.4	12.8	5.1	4.1	0.6	*969*
	> average	5.8	*740*	78.4	13.3	6.1	1.7	0.4	*790*
	Richest	5.4	*603*	73.6	17.6	4.5	2.6	1.6	*640*
**Total**		**5.6**	***4250***	77.1	14.2	5.4	2.5	0.8	***4528***

Figure 6.1 Graph showing average WHODAS score by age, sex and nationality

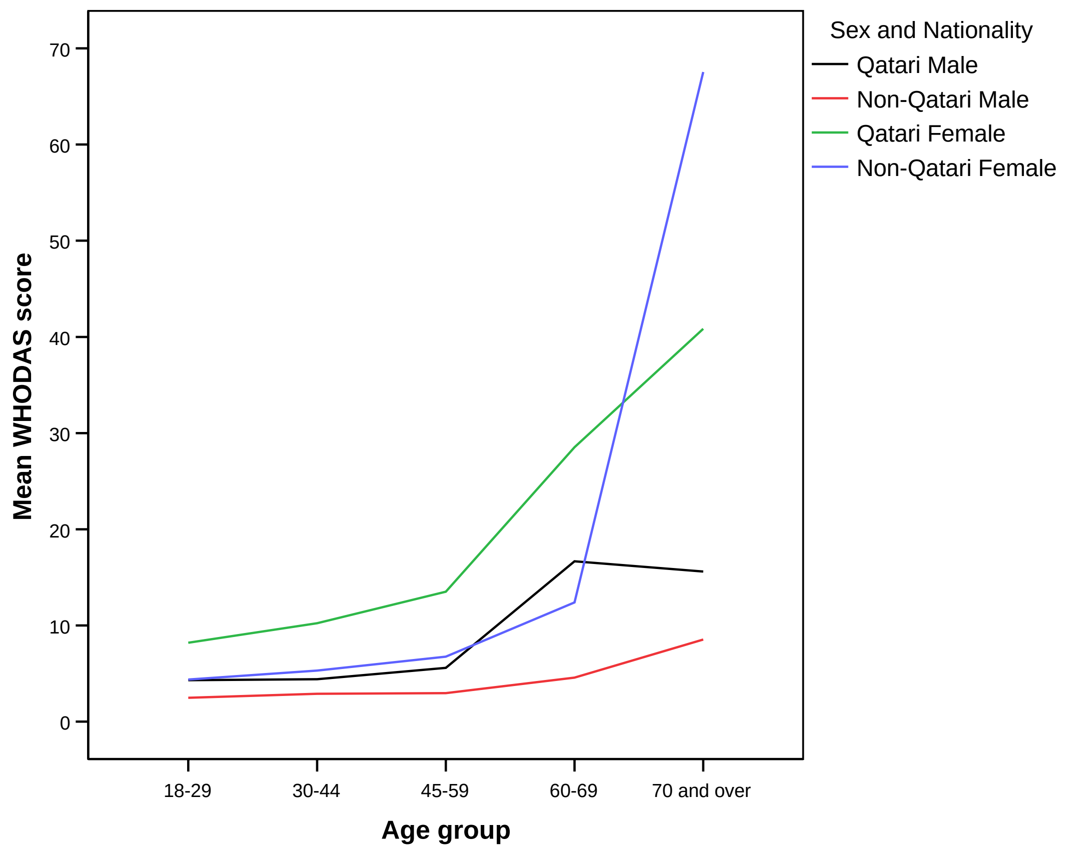

7. Health system responsiveness

7.1 Introduction

Health systems responsiveness, by definition, covers a wide range of outcomes and varies from context to context. For example, the quality of the health care systems and its response to patient needs may improve use patterns, thereby directly affecting health outcomes. On the other hand, showing responsiveness to citizens in the provision of timely health information can contribute to a raised level of public trust in the health care system and the willingness to pay for that system through the levy of taxes (Valentine, Ortiz & Tandon, 2003). The response of both public and private institutions towards the provision of health needs is defined as health system responsiveness. It is the individuals’ experience of their interaction with the health system on a number of different aspects, or domains, that are of interest, as the interaction between individual and health system influences well-being.

The Qatar World Health Survey investigated a number of different domains of health system responsiveness. These are obtained from self-reports about personal experiences. However, it is possible to adjust these self-reports for expectation and to ensure comparability between population groups. Vignettes can be used to measure population subgroups’ expectations of health care, with all individuals answering questions about how they rate a fictional person’s health system interaction. These responses can be used as a baseline to judge the individual experience against.

The health system in Qatar is undergoing radical changes, with the introduction of a health insurance model of healthcare delivery. The Qatar World Health Survey was conducted before the implementation of these changes occurred and therefore measures satisfaction with healthcare under the old system. Further surveys are required to assess the benefits, or otherwise, of the new health system organization to the user.

7.2 Self-assessed need for health care

7.2.1 Need for any type of health care

The responsiveness of the health system is measured by the ability of the health system to meet the health requirements of its population. Individuals were asked to report the most recent time that they needed health care, and if this was in the previous three years to the survey they were asked whether they obtained the health care that they needed. The type of health care needed was not specified in the question and therefore the responses include both inpatient and outpatient care, and care at home. The results for this are shown in Table 7.1.

Almost two-thirds of the individuals responding to the survey stated the most recent time that they needed health care was in the three years prior to the survey. Of these, 98.9% stated that they received care at this time. This means that only 1% of the individuals did not obtain health care the when they needed it.

There are some major differences between subgroups of the population. Females were more likely to need health care than males: only 58.7% of male respondents stated they needed care in the previous three years, compared with 73.9% of females. A higher proportion of females also received the care that they needed, although there was no large difference between the sexes. Qatari nationals were also more likely to have needed health care than non-Qatari nationals, although a higher percentage of non-Qataris received the required care.

Table 7.1 Percentage of people needing and receiving health care in the past three years

		Needing health care			Obtaining health care if needed	
		% needing	% not needing	Count	% yes	*N*
**Sex**						
	Male	58.7	41.3	*2405*	98.6	*1341*
	Female	73.9	26.1	*2347*	99.2	*1689*
**Nationality status**						
	Qatari	75.1	24.9	*1604*	98.3	*1181*
	Non-Qatari	61.7	38.3	*3147*	99.3	*1849*
**Region**						
	Doha	61.6	38.4	*2579*	99.0	*1537*
	Al Rayyan	73.2	26.9	*1467*	98.7	*1023*
	Other regions	68.8	31.2	*706*	99.1	*469*
**Age group**						
	18 to 29	63.3	36.7	*1095*	98.9	*666*
	30 to 44	69.2	30.8	*2374*	98.9	*1580*
	45 to 59	62.1	37.9	*1122*	99.0	*673*
	60 to 69	67.4	32.7	*126*	99.2	*83*
	70 or over	81.6	18.4	*35*	100.0	*29*
**Self-assessed health**						
	Very good	58.6	41.4	*2099*	98.6	*1161*
	Good	70.2	29.9	*2060*	99.5	*1404*
	Moderate	78.7	21.3	*505*	98.0	*390*
	Bad	86.8	13.2	*73*	99.0	*63*
	Very bad	88.0	12.1	*13*	93.7	*11*
**Wealth quintile**						
	Poorest	65.1	34.9	*1220*	98.6	*759*
	< average	66.6	33.5	*1013*	98.6	*642*
	Average	67.4	32.6	*1017*	99.0	*666*
	> average	64.5	35.5	*836*	99.5	*521*
	Richest	68.0	32.0	*666*	99.1	*441*
**Total**		**66.2**	**33.8**	***4751***	98.9	***3030***

Differences between regions are obvious. Doha had the lowest percentage of people who needed health care, with 61.6%, compared with other regions of Qatar with 68.8% and Al Rayyan with 73.2%. However, the percentage of those who needed it who received care was about the same, irrespective of region. The health care requirements of those with self-perceived bad health was also assessed. As self-perceived health became worse, a higher percentage of people stated that they needed health care.

7.3 Need for outpatient health care

The Qatar World Health Survey assessed the receipt of outpatient care among the respondents, and the percentage of people who obtained outpatient health care when they last needed it. Outpatient care is defined as care not involving an overnight stay in a hospital, and so includes care received at a hospital, health centre, clinic, private office or at home from a health care worker. The period of reference for the questions on the last outpatient visit was the 12 months prior to the date of interview. Only those who stated that they had used any type of health care in the previous three years were asked about outpatient care. The results are shown in Table 7.2.

Nearly four out of five of the respondents (79.6%) stated that they had received outpatient care in the previous 12 months. There were slightly higher proportions of females than males and Qatar nationals than non-Qatari nationals who used outpatient care. Those living in Doha used outpatient care more than those living in the other regions of Qatar, while there was some evidence of decreasing use as wealth increased. Need increased as self-assessed health deteriorated, except for those who placed themselves in the worst health category. Only 60.8% of respondents in this group stated that they had used outpatient care, although there were only 11 respondents in this group.

For those who did receive outpatient health care, most stated that they received the care when they needed it. Overall, 98.8% of respondents said that health care was forthcoming when they needed it. There are few differences between subgroups of the population, and receipt of care is ubiquitously high.

Table 7.2 Percentage of people needing and receiving outpatient health care in the past 12 months

		Outpatient care in the past 12 months		Outpatient care received when needed?	
		**% yes**	***N***	**% yes**	**N**
**Sex**					
	Male	79.3	*1341*	98.8	*1062*
	Female	79.9	*1689*	98.9	*1340*
**Nationality status**					
	Qatari	80.3	*1181*	98.3	*947*
	Non-Qatari	79.2	*1849*	99.2	*1455*
**Region**					
	Doha	80.7	*1537*	98.8	*1232*
	Al Rayyan	80.2	*1023*	98.8	*818*
	Other regions	75.0	*469*	99.4	*351*
**Age group**					
	18 to 29	79.8	*666*	98.8	*526*
	30 to 44	78.9	*1580*	98.6	*1240*
	45 to 59	80.9	*673*	99.2	*544*
	60 to 69	75.5	*83*	100.0	*62*
	70 or over	100.0	*29*	100.0	*29*
**Self-assessed health**					
	Very good	75.9	*1161*	98.5	*877*
	Good	81.4	*1404*	99.2	*1137*
	Moderate	85.1	*390*	98.5	*331*
	Bad	80.3	*63*	98.8	*51*
	Very bad	60.8	*11*	100.0	*7*
**Wealth quintile**					
	Poorest	83.0	*759*	98.7	*626*
	< average	80.7	*642*	98.8	*516*
	Average	77.9	*666*	99.1	*516*
	> average	78.8	*521*	99.2	*410*
	Richest	76.0	*441*	98.5	*333*
**Total**		**79.6**	***3030***	98.8	*2402*

7.4 Health systems responsiveness

The World Health Organization identifies seven major domains against which health system responsiveness in a country needs to be judged. These domains address the different aspects and concerns of the people who are seeking health care, and are described as follows.

1. Autonomy: the ability of individuals to obtain information about their disease and to be consulted about the treatment offered. This domain also relates to the involvement of the patient in the decision-making process.
2. Choice: the freedom for patients to choose their health care provider. This includes having the information available for an informed choice to be made.
3. Communication: the two-way interaction between health care provider and patient, encompassing clear explanations and the availability of time to interact with the health care provider.
4. Confidentiality: the conducting of consultations in a manner which protects the patients' privacy, safeguarding the confidentiality of the information provided by the patient.
5. Dignity: the right of people to be treated as persons in their own right and to be given respect by the health care workers. This also covers privacy during physical examinations.
6. Quality of basic amenities: the provision of the basic physical infrastructure and a conducive care environment, including a clean environment and healthy and edible food.
7. Prompt attention: the provision of timely and appropriate care. This domain contains three different aspects: rapid care in emergencies, care within a reasonable time for non-emergency care (i.e. short waiting lists) and a short waiting time for consultations and treatment.

The Qatar World Health Survey asked respondents to rate their mot recent inpatient and outpatient visit on these seven domains through asking a series of questions, with one question being asked on each domain. The respondents were given a scale of 1 to 5 to rate their experience, with 1 representing very good care while 5 represented very bad care. It is assumed that the difference between very bad and bad is the same as the difference between very good and good ratings on each domain.

Each respondent was also asked to respond to a series of vignettes regarding health system responsiveness. A small vignette was read to the respondent, who then rated the subject of the vignette's experience on the same scale as before. There was one vignette for each of the domains, and these vignettes were chosen to represent an average experience of care. The responses to these vignettes give an idea about the expectation of the individual. Their own responses on each of the domains can be judged against these vignettes to assess if their experience is better or worse than the average experience.

Therefore there are three different ways in which health responsiveness can be analysed.

1. Recording the percentage of people stating that they had a good or very good experience of health care in each of the domains.

2. Calculating a score out 100 for health system responsiveness, with 100 representing complete responsiveness and 0 a complete absence of responsiveness. This was calculated by obtaining the average score for the respondents and rescaling this score to be between 0 and 100.

3. Matching the personal satisfaction ratings against the vignette scores, and calculating the percentage of those with a higher score, a lower score or the same score. A higher personal score than vignette score indicates an experience which is worse than average, while a lower personal score represents an experience which is better than an average experience. The actual vignettes are contained in the questionnaire (see Appendix A).

Responsiveness can be analysed for both inpatient and outpatient visits. However, due to a mistake in the questionnaire the questions which were aimed to collect information about outpatient care, this question asked about *all* health care, including inpatient care. Questions about inpatient care had already been asked, so it is assumed that the responses to the question mainly represented outpatient care although this assumption cannot be tested.

7.4.1 Responsiveness for outpatient care

The responsiveness ratings for outpatient care, measured in all three methods noted above, are presented in Tables 7.3a, 7.3b and 7.3c. Table 7.3a gives the percentage of people rating outpatient care as very good or good, while Table 7.3b shows the responsiveness score obtained for each domain. The final table, Table 7.3c, displays the percentage of respondents who rated their own experience as better, the same or worse than the corresponding domain vignette.

Quality of basic amenities is the domain that had the highest percentage of ratings of good or very good. 89.5% of respondents rated the quality in this way, while other domains with a percentage of users with satisfied ratings over 80% include dignity, communication and confidentiality. The domains of autonomy and choice were rated as good by almost 80% of respondents. The domain that had the lowest rating was prompt attention, where only 69.5% of individuals stated that this was good. Generally, ratings on all domains were lowest in Doha and highest in the other regions of Qatar. However, on some of the domains these differences were negligible. With regard to self-assessed health, on the domains of prompt attention, dignity, communication and quality of amenities the percentage satisfied decreased as health got worse. On the other three domains the health status of the individual did not have much of an affect on satisfaction.

For the responsiveness ratings of outpatient care on a scale of 0 to 100 (Table 7.3b) similar patterns emerged. Quality of amenities had the highest rating, while prompt attention had the lowest, although there is only a difference of about 11 points between the two. The regions outside Al Rayyan and Doha had the highest ratings on most of the domains, while ratings fell on certain aspects of the care as self-rated health deteriorated. An overall rating of the outpatient health system indicated that the rating given was 76.5 out of 100. There were minimal differences in ratings between the sexes and nationality status. Doha and Al Rayyan had the same average rating of 76.2, which was lower than the average rating in the other areas of Qatar, where the raring was 79.2. There were some differences between age groups, although no clear trends could be discerned.

Table 7.3a Distribution of outpatient care satisfaction on seven domains of responsiveness

		% Satisfied with the services offered *						
	Prompt attention	Dignity	Communication	Autonomy	Confidentiality	Choice	Quality of amenities	*N*
**Sex**								
Male	72.9	85.0	82.0	79.1	87.7	77.5	90.8	*1079*
Female	66.9	85.1	82.6	79.4	86.9	77.5	88.5	*1360*
**Nationality status**								
Qatari	70.0	86.1	81.1	79.7	85.6	77.9	85.9	*961*
Non-Qatari	69.3	84.4	83.2	79.0	88.3	77.2	91.8	*1479*
**Region**								
Doha	68.1	84.0	81.1	77.4	87.1	77.5	89.1	*1251*
Al Rayyan	70.8	84.3	82.4	79.0	85.4	76.5	90.3	*829*
Other regions	71.7	90.4	86.6	86.2	92.0	79.8	89.2	*360*
**Age group**								
18 to 29	68.3	84.2	81.3	79.6	85.9	75.8	88.0	*538*
30 to 44	68.4	83.8	80.3	77.0	85.8	77.1	89.6	*1253*
45 to 59	75.0	88.5	87.5	84.1	90.9	80.1	90.8	*555*
60 to 69	58.5	86.6	81.1	72.9	90.9	73.0	88.7	*63*
70 or over	61.8	85.4	93.1	92.2	96.1	88.5	90.7	*29*
**Self-assessed health**								
Very good	73.9	87.3	84.5	80.3	88.3	76.9	91.0	*890*
Good	68.7	84.8	83.0	78.5	87.3	78.9	90.4	*1153*
Moderate	62.6	82.0	75.8	80.2	84.0	75.1	84.8	*338*
Bad	58.7	70.2	76.4	76.5	88.2	75.6	77.6	*51*
Very bad	69.7	100.0	64.9	50.8	100.0	42.4	60.9	*7*
**Wealth quintile**								
Poorest	73.1	86.2	84.0	81.4	88.5	78.5	89.5	*640*
< average	68.2	85.1	80.2	79.4	86.3	77.4	91.9	*517*
Average	67.9	84.2	83.1	77.0	86.4	76.2	87.1	*523*
> average	66.6	83.8	82.9	78.9	89.1	77.2	89.9	*415*
Richest	70.9	85.5	80.7	79.0	85.5	78.1	89.1	*345*
**Total**	**69.5**	**85.0**	**82.4**	**79.3**	**87.2**	**77.5**	**89.5**	***2439***

* Satisfied is a rating of 'very good' or 'good'

Table 7.3b Overall responsiveness ratings for seven domains of outpatient care

	Mean rating of outpatient care (0 = very bad; 100 = very good)*								
	**Prompt attention**	**Dignity**	**Communication**	**Autonomy**	**Confidentiality**	**Choice**	**Quality of amenities**	**Overall**	***N***
**Sex**									
Male	71.2	77.4	76.7	75.7	79.0	74.8	81.5	76.6	*1086*
Female	69.4	77.8	77.1	75.3	79.1	74.9	80.7	76.3	*1367*
**Nationality status**									
Qatari	70.3	79.0	77.3	76.5	79.5	75.9	79.8	76.9	*963*
Non-Qatari	70.1	79.0	76.6	74.8	78.8	74.2	81.9	76.2	*1491*
**Region**									
Doha	69.4	77.3	76.7	74.9	79.3	74.9	80.7	76.2	*1261*
Al Rayyan	69.9	76.6	75.6	74.4	77.6	73.9	81.1	76.2	*833*
Other regions	73.2	80.7	80.5	74.4	81.5	76.7	82.3	79.2	*360*
**Age group**									
18 to 29	70.3	77.4	76.5	75.2	78.6	73.5	80.3	76.0	*543*
30 to 44	69.4	77.1	76.2	74.6	78.6	74.7	80.9	75.9	*1261*
45 to 59	72.7	78.7	78.9	77.7	80.4	76.8	81.9	78.2	*557*
60 to 69	65.8	79.8	76.4	75.1	79.3	71.9	82.2	75.8	*63*
70 or over	63.1	78.9	81.0	80.4	82.0	71.9	82.6	78.0	*29*
**Self-assessed health**									
Very good	73.8	80.5	80.2	78.1	78.5	77.3	83.9	79.4	*897*
Good	68.8	76.1	75.4	74.0	77.6	73.8	79.7	75.1	*1160*
Moderate	65.8	75.3	73.5	73.8	76.7	72.6	78.8	75.1	*338*
Bad	61.6	72.5	71.3	71.0	75.8	70.4	75.3	71.1	*52*
Very bad	66.1	77.8	71.1	65.3	76.0	58.2	71.0	69.4	*7*
**Wealth quintile**									
Poorest	71.7	77.9	77.8	76.2	79.5	75.8	81.4	77.2	*641*
< average	70.3	77.1	76.3	75.5	78.7	74.8	81.2	76.3	*526*
Average	69.3	77.6	76.7	75.5	78.6	73.8	79.9	75.8	*524*
> average	68.7	77.9	77.4	75.6	79.8	74.7	81.9	76.6	*418*
Richest	70.4	77.4	76.1	75.3	78.5	75.0	81.1	76.2	*345*
**Overall mean**	70.2	77.6	76.9	75.5	79.0	74.9	81.1	76.5	***2453***

* Responsiveness rating calculated by taking the average of the satisfaction scores for each domain and scaling this mean to range from 0 to 100

Table 7.3c Distribution of the comparison of outpatient care ratings against vignette ratings

	Prompt attention			Dignity			Communication			Autonomy			Confidentiality			Choice			Quality of basic amenities			
	**Better**	**Same**	**Worse**	**Better**	**Same**	**Worse**	**Better**	**Same**	**Worse**	**Better**	**Same**	**Worse**	**Better**	**Same**	**Worse**	**Better**	**Same**	**Worse**	**Better**	**Same**	**Worse**	***N***
**Sex**																						
Male	88.6	9.4	2.0	67.2	22.9	9.9	86.6	11.0	2.4	63.3	25.6	11.1	85.9	10.1	4.0	77.8	17.3	5.0	95.4	4.0	0.6	*1385*
Female	87.0	10.9	2.1	66.8	22.1	11.1	87.5	10.3	2.2	64.1	24.9	11.1	86.4	10.2	3.4	77.9	18.0	4.1	95.1	4.1	0.8	*1721*
**Nationality status**																						
Qatari	86.0	11.6	2.4	66.5	23.6	9.8	85.7	12.2	2.0	65.1	25.4	9.5	86.8	10.3	2.9	78.0	17.8	4.2	93.6	5.4	1.0	*1208*
Non-Qatari	88.8	9.3	1.8	67.3	21.7	11.1	88.0	9.6	2.4	62.9	25.1	12.1	85.9	10.1	4.1	77.7	17.6	4.7	96.3	3.1	0.6	*1897*
**Region**																						
Doha	86.0	11.7	2.3	66.3	23.1	10.6	86.2	11.0	2.8	64.1	24.2	11.7	85.2	11.9	2.9	78.4	17.3	4.3	94.4	4.9	0.7	*1580*
Al Rayyan	88.4	9.6	2.0	65.8	22.7	11.6	86.9	11.0	2.2	61.3	27.3	11.4	84.9	9.6	5.5	75.8	19.7	4.5	95.9	3.3	0.8	*1045*
Other regions	91.9	6.9	1.3	71.8	19.8	8.5	90.7	8.6	0.7	68.0	23.9	8.1	92.3	5.6	2.1	80.6	14.5	4.9	96.4	2.8	0.9	*481*
**Age group**																						
18 to 29	87.7	10.1	2.2	68.3	22.1	9.7	89.8	8.6	1.7	61.6	26.2	12.2	87.4	9.2	3.4	75.1	18.9	6.0	95.3	4.0	0.8	*694*
30 to 44	87.8	10.2	2.0	66.4	21.5	12.2	85.3	11.6	3.1	62.4	26.3	11.4	84.8	11.6	3.6	77.9	18.2	3.9	95.2	4.1	0.7	*1604*
45 to 59	88.1	9.8	2.1	67.6	23.9	8.5	89.4	9.6	1.0	69.5	21.4	9.1	88.5	7.7	3.8	80.8	15.0	4.2	95.2	3.8	1.0	*694*
60 to 69	84.0	15.2	0.8	65.5	30.0	4.5	79.6	17.9	2.5	58.5	29.2	12.3	82.4	12.4	5.2	72.9	22.4	4.8	93.8	6.2	0.0	*83*
70 or over	80.2	14.8	5.0	62.1	27.9	10.0	91.4	8.6	0.0	67.6	23.2	9.3	90.7	5.6	3.8	83.5	12.7	3.8	98.4	1.6	0.0	*30*
**Self-assessed health**																						
Very good	89.8	8.9	1.4	68.4	21.9	9.7	88.0	9.9	2.1	64.7	24.9	10.4	86.5	9.8	3.6	78.2	17.4	4.4	95.3	4.2	0.6	*1196*
Good	87.0	10.4	2.6	67.4	22.5	10.2	87.5	10.0	2.5	63.1	25.7	11.2	86.0	10.4	3.6	78.1	17.5	4.4	95.6	3.6	0.8	*1437*
Moderate	85.5	12.3	2.2	61.7	23.9	14.4	84.8	13.4	1.7	64.5	23.6	11.9	85.7	10.3	4.1	77.7	18.0	4.3	95.1	4.1	0.8	*398*
Bad	83.0	14.9	2.1	68.8	18.6	12.6	80.9	17.2	1.9	61.5	26.6	11.9	89.1	8.4	2.4	70.5	21.8	7.7	91.5	6.6	1.9	*64*
Very bad	64.0	36.0	0.0	42.2	45.4	12.4	59.9	34.0	6.1	37.0	40.4	22.6	77.4	16.6	6.1	47.2	40.4	12.5	76.2	23.8	0.0	*11*
**Wealth quintile**																						
Poorest	90.4	7.4	2.3	67.7	22.1	10.2	88.7	8.9	2.4	63.1	25.6	11.3	87.1	8.6	4.3	79.5	16.8	3.7	96.0	3.3	0.6	*784*
< average	87.1	10.8	2.1	65.8	23.8	10.4	85.8	11.8	2.4	66.7	23.8	9.5	85.2	11.1	3.7	76.5	17.1	6.4	96.0	3.4	0.6	*654*
Average	86.9	11.3	1.8	69.5	20.4	10.1	87.7	10.0	2.3	63.6	25.1	11.3	85.7	11.0	3.2	77.8	18.1	4.1	94.4	4.8	0.8	*679*
> average	86.1	11.4	2.5	66.5	22.6	11.0	87.8	9.8	2.4	62.2	25.9	12.0	88.1	8.9	3.0	77.5	19.2	3.4	95.5	3.6	1.0	*532*
Richest	87.0	11.4	1.6	64.3	24.0	11.8	84.3	14.0	1.6	62.6	25.9	11.5	84.6	11.6	3.9	77.3	17.7	4.9	93.7	5.5	0.8	*456*
**Total**	**87.7**	**10.2**	**2.1**	**67.0**	**22.4**	**10.6**	**87.1**	**10.6**	**2.3**	**63.7**	**25.2**	**11.1**	**86.2**	**10.2**	**3.7**	**77.8**	**17.7**	**4.5**	**95.2**	**4.0**	**0.7**	***3106***

* Satisfied is a rating of ‘very good’ or ‘good’

The final table relating to the responsiveness of outpatient care, Table 7.3c, again indicates that quality of basic amenities was the highest rated domain. 95.2% of respondents rated their own experience better than experience described in the vignette, while only 0.7% rated their experience as worse. Other domains which were also rated well were prompt attention (87.7% better and 2.1% worse than vignette), communication (87.1% and 2.3% respectively) and confidentiality (86.2% and 3.7% respectively).

The percentage of respondents who rated their experience of choice as better than the situation stated in the vignette was only 77.8%, while 17.7% rated their experience at the same level as the vignette. However, the domains of dignity and autonomy had the lowest percentage of individuals with own experience ratings better than the vignette, with a high proportion of people who rated their experience lower than the vignette. For dignity, about a third of respondents rated their experiences as the same or worse than the vignette, while for autonomy the percentage rating their experience as the same or worse was 36.3%.

7.4.2 Responsiveness for inpatient care

The three tables for responsiveness for inpatient care are displayed in Tables 7.4a, 7.4b and 7.4c. These again list the percentage of respondents who were satisfied, the overall responsiveness rating of the different domains and the comparison with the domain vignettes respectively.

The percentage of respondents with a hospital stay who were satisfied on each domain of responsiveness was very similar to the satisfaction seen for outpatient responsiveness, except for prompt attention, where the satisfaction was higher for inpatient care. Four domains again had a satisfaction score of over 80%. These domains were dignity, communication, confidentiality and the quality of the amenities. Prompt attention had the lowest percentage of people who were satisfied, with only 75.1% of respondents stating that this aspect was very good or good. The other two domains all had satisfaction ratings of nearly 80%. It must be noted that there was more than 8% of individuals who stayed in a hospital over the previous 12 months who did not respond to the satisfaction questions. These were excluded from the analysis, but it is unknown how these respondents rated the responsiveness of the care that they received.

Table 7.4a Distribution of inpatient care satisfaction on seven domains of responsiveness

	% satisfied with the services offered *								
	Prompt attention	Dignity	Communication	Autonomy	Confidentiality	Choice	Quality of amenities	*N*	% missing
**Sex**									
Male	76.8	87.4	84.2	79.8	85.9	80.1	93.4	*123*	7.4
Female	74.6	85.7	81.9	79.0	86.4	77.8	87.2	*364*	9.0
**Nationality status**									
Qatari	72.5	84.8	79.4	81.3	84.9	76.4	82.9	*218*	8.9
Non-Qatari	77.3	87.1	85.0	77.5	87.4	80.0	93.4	*269*	8.4
**Region**									
Doha	72.2	84.6	75.9	73.7	81.1	75.3	87.0	*212*	12.4
Al Rayyan	80.3	88.4	89.0	83.1	89.9	79.8	91.4	*185*	4.8
Other regions	71.4	84.9	84.8	84.1	90.9	82.9	87.4	*90*	7.0
**Age group**									
18 to 29	80.0	83.9	79.2	76.1	83.0	76.5	87.8	*137*	8.0
30 to 44	73.8	87.3	83.4	79.5	86.7	78.4	88.3	*271*	8.5
45 to 59	73.0	84.9	81.5	78.8	90.2	82.4	90.6	*59*	10.4
60 to 69	65.2	93.6	97.0	100.0	97.0	91.8	97.0	*16*	8.1
70 or over	71.9	71.9	85.3	85.3	71.8	30.3	85.3	*4*	10.8
**Self-assessed health**									
Very good	78.9	87.6	86.2	78.2	86.5	80.1	89.8	*165*	9.9
Good	75.4	87.6	81.0	80.4	87.3	78.5	88.3	*231*	7.7
Moderate	75.2	84.6	83.9	78.8	87.7	79.7	89.2	*69*	6.5
Bad	48.5	61.1	62.2	73.1	71.5	64.2	84.0	*19*	16.7
Very bad	37.4	84.5	84.5	84.5	66.0	49.6	81.6	*4*	0.0
**Wealth quintile**									
Poorest	75.0	86.8	83.8	77.6	85.0	79.5	86.1	*116*	11.2
< average	75.4	84.0	80.6	76.0	85.4	82.4	90.9	*121*	9.3
Average	75.9	85.8	80.3	80.3	85.5	76.8	89.3	*112*	7.3
> average	70.6	88.4	84.2	76.9	86.6	74.1	87.3	*76*	8.0
Richest	78.9	86.5	85.7	89.1	91.4	76.7	90.1	*62*	6.1
**Type of hospital**									
Public	75.0	85.8	82.1	78.8	86.2	77.8	88.9	*445*	8.8
Private	78.9	90.4	88.2	84.5	89.7	86.1	89.2	*40*	6.2
Other	33.7	60.3	60.3	60.3	33.7	60.3	33.7	*2*	7.8
**Total**	**75.1**	**86.1**	**82.5**	**79.2**	**86.3**	**78.4**	**88.7**	***487***	**8.6**

* Satisfied is a rating of ‘very good’ or ‘good’

Table 7.4b Overall responsiveness rating for seven domains of inpatient care

	Mean rating of inpatient care (0 = very bad; 100 = very good)*								
	**Prompt attention**	**Dignity**	**Communication**	**Autonomy**	**Confidentiality**	**Choice**	**Quality of amenities**	**Overall**	***N***
**Sex**									
Male	70.8	77.7	77.3	75.2	77.7	73.0	83.1	76.4	*133*
Female	70.4	77.3	75.4	74.4	78.1	74.3	80.8	75.8	*401*
**Nationality status**									
Qatari	70.2	78.0	76.3	76.8	78.8	74.6	80.1	76.4	*240*
Non-Qatari	70.8	76.8	75.5	72.8	77.4	73.5	82.5	75.7	*294*
**Region**									
Doha	68.7	76.2	73.0	72.2	75.9	72.3	79.7	74.0	*242*
Al Rayyan	72.7	78.5	78.3	75.8	79.4	74.3	83.2	77.6	*195*
Other regions	70.4	77.7	77.7	78.1	79.4	77.4	81.5	77.5	*96*
**Age group**									
18 to 29	71.4	76.1	74.7	73.3	76.9	73.5	80.9	75.3	*149*
30 to 44	70.2	77.4	75.3	74.5	78.0	73.4	80.9	75.7	*297*
45 to 59	70.2	77.6	78.9	76.4	78.0	73.4	82.7	77.6	*66*
60 to 69	70.2	88.1	83.9	82.7	82.9	79.6	88.9	82.3	*18*
70 or over	69.1	74.8	77.4	69.1	68.9	61.0	79.9	71.5	*5*
**Self-assessed health**									
Very good	73.2	79.9	80.4	78.2	81.0	77.2	83.0	79.0	*183*
Good	70.1	76.8	73.8	73.3	77.2	73.3	80.8	75.1	*250*
Moderate	70.4	75.6	73.8	71.4	77.2	71.6	79.6	73.9	*73*
Bad	60.9	70.9	71.4	74.8	75.2	69.3	80.6	72.2	*23*
Very bad	41.3	72.3	74.6	68.8	73.3	57.2	81.6	67.0	*4*
**Wealth quintile**									
Poorest	70.3	77.5	75.4	74.9	77.9	76.5	82.0	76.4	*130*
< average	70.3	76.5	75.5	73.4	78.5	75.8	82.0	76.0	*133*
Average	70.4	76.5	75.2	74.9	77.4	73.1	79.2	75.1	*121*
> average	69.7	78.3	78.8	74.0	77.8	70.3	81.2	75.9	*83*
Richest	72.4	79.1	74.9	76.4	79.0	72.6	83.0	76.7	*67*
**Type of hospital**									
Public	70.3	76.8	75.8	74.5	78.0	73.6	81.2	75.7	*489*
Private	73.3	83.6	76.9	76.0	79.2	78.9	83.8	78.8	*43*
Other	66.8	73.5	73.5	65.1	66.8	73.5	66.8	69.4	*2*
**Total**	**70.5**	**77.4**	**75.9**	**74.6**	**78.0**	**74.0**	**81.4**	**76.0**	***534***

* Responsiveness rating calculated by taking the average of the satisfaction scores for each domain and scaling this mean to range from 0 to 100

Table 7.4c Distribution of the comparison of inpatient care ratings against vignette ratings

		Prompt attention			Dignity			Communication			Autonomy			Confidentiality			Choice			Quality of basic amenities			
		Better	Same	Worse	Better	Same	Worse	Better	Same	Worse	Better	Same	Worse	Better	Same	Worse	Better	Same	Worse	Better	Same	Worse	*N*
**Sex**																							
	Male	88.7	8.7	2.6	68.0	21.0	11.0	88.4	6.0	5.6	65.0	20.9	14.1	84.6	8.4	7.0	72.4	21.8	5.8	97.0	2.3	0.7	*135*
	Female	90.0	8.8	1.2	70.3	17.7	12.0	86.8	11.0	2.2	64.2	21.7	14.1	89.6	7.2	3.2	79.4	16.4	4.2	96.2	2.7	1.1	*425*
**Nationality status**																							
	Qatari	86.9	11.4	1.7	71.3	18.4	10.4	84.7	12.4	2.9	62.8	25.7	11.6	88.6	8.0	3.4	75.2	19.5	5.3	94.2	4.2	1.6	*257*
	Non-Qatari	92.1	6.5	1.4	68.4	18.7	12.9	89.3	7.6	3.1	65.7	18.0	16.3	88.3	7.0	4.7	79.9	16.2	4.0	98.3	1.3	0.4	*303*
**Region**																							
	Doha	87.2	11.8	1.0	68.9	17.7	13.4	85.5	9.9	4.6	65.7	18.5	15.7	85.1	10.3	4.6	77.1	19.1	3.8	96.0	3.5	0.6	*244*
	Al Rayyan	92.0	5.2	2.9	70.3	17.3	12.4	89.1	8.8	2.1	60.5	25.9	13.6	90.9	4.9	4.2	76.6	17.0	6.3	96.4	2.2	1.4	*213*
	Other regions	91.1	8.9	0.0	70.5	23.1	6.4	87.0	11.9	1.2	69.0	19.5	11.5	91.1	6.2	2.7	81.5	15.7	2.9	97.4	1.5	1.1	*103*
**Age group**																							
	18 to 29	89.7	8.6	1.6	71.8	18.8	9.4	88.7	10.5	0.8	64.6	22.3	13.1	91.7	6.1	2.2	78.5	17.6	3.9	97.1	2.0	0.9	*153*
	30 to 44	89.3	9.5	1.1	67.8	18.4	13.8	84.7	11.1	4.3	64.2	20.7	15.1	86.8	8.5	4.8	76.3	19.0	4.7	95.4	3.3	1.3	*318*
	45 to 59	91.9	4.3	3.8	72.7	19.4	7.9	93.5	3.3	3.2	66.4	24.1	9.5	91.2	5.9	2.9	83.9	9.9	6.2	100.0	0.0	0.0	*68*
	60 to 69	88.2	11.8	0.0	72.8	18.5	8.7	93.6	6.4	0.0	53.7	18.6	27.7	77.7	10.9	11.5	83.6	13.6	2.9	93.4	6.6	0.0	*17*
	70 or over	87.8	12.2	0.0	78.3	10.0	11.8	88.2	11.8	0.0	78.4	21.7	0.0	88.2	0.0	11.8	45.1	54.9	0.0	100.0	0.0	0.0	*5*
**Self-assessed health**																							
	Very good	90.1	8.5	1.4	66.7	21.0	12.2	92.2	6.3	1.6	73.2	18.0	8.8	90.5	6.8	2.7	82.9	13.8	3.3	98.0	1.7	0.3	*182*
	Good	90.0	8.3	1.8	71.9	16.1	12.0	85.3	10.1	4.6	59.7	23.4	16.9	86.5	8.3	5.2	74.9	19.4	5.7	96.1	2.8	1.1	*266*
	Moderate	91.6	6.9	1.5	71.4	20.5	8.1	87.6	10.9	1.5	63.0	20.8	16.2	90.6	7.2	2.2	81.2	16.6	2.2	94.2	3.7	2.1	*84*
	Bad	88.2	11.8	0.0	67.0	21.5	11.6	67.0	33.0	0.0	53.8	35.1	11.2	89.2	2.9	7.8	57.2	40.4	2.4	94.7	5.3	0.0	*20*
	Very bad	41.3	58.7	0.0	50.7	14.2	35.1	73.9	14.2	11.9	55.5	9.4	35.1	73.9	14.2	11.9	61.3	0.0	38.7	100.0	0.0	0.0	*6*
**Wealth quintile**																							
	Poorest	89.4	9.2	1.4	69.8	14.6	15.6	89.0	6.4	4.6	63.1	23.5	13.4	88.4	6.2	5.3	85.3	10.6	4.1	96.2	1.9	1.9	*132*
	< average	87.0	12.0	0.9	63.5	24.7	11.8	87.3	8.7	3.9	68.4	17.4	14.1	84.2	11.6	4.2	74.6	21.6	3.8	96.8	3.2	0.0	*131*
	Average	91.4	5.9	2.7	69.3	19.1	11.6	88.4	9.7	2.0	63.2	22.5	14.3	90.6	7.1	2.3	79.8	15.8	4.5	96.3	2.6	1.1	*126*
	> average	89.2	8.4	2.3	72.4	15.8	11.8	85.0	13.5	1.5	61.7	19.6	18.7	90.0	6.2	3.8	70.7	21.2	8.0	95.1	3.2	1.6	*94*
	Richest	92.6	7.4	0.0	77.7	17.1	5.2	84.2	13.4	2.4	64.8	25.7	9.5	90.1	4.9	5.0	75.4	22.0	2.6	97.7	2.3	0.0	*77*
**Type of hospital**																							
	Public	89.4	9.0	1.6	70.0	18.3	11.7	87.2	10.3	2.5	64.0	21.7	14.3	88.6	6.9	4.5	76.9	18.1	5.0	96.9	2.1	1.1	*511*
	Private	93.5	5.1	1.4	68.0	19.3	12.8	86.1	5.0	8.9	70.8	19.6	9.6	86.0	14.1	0.0	86.5	13.5	0.0	91.3	8.7	0	*46*
	Other	73.4	26.6	0.0	39.7	60.3	0.0	100.0	0.0	0.0	0.0	26.6	73.4	100.0	0.0	0.0	100.0	0.0	0.0	100.0	0.0	0	*1*
**Total**		**89.7**	**8.7**	**1.5**	**69.8**	**18.5**	**11.7**	**87.2**	**9.8**	**3.0**	**64.4**	**21.5**	**14.1**	**88.4**	**7.5**	**4.1**	**77.7**	**17.7**	**4.6**	**96.4**	**2.6**	**1.0**	***559***

There were larger differences between subgroups for inpatient compared to outpatient responsiveness. Satisfaction for males was higher than satisfaction for females on each of the domains of responsiveness except for confidentiality, where the difference was negligible. The largest difference in satisfaction between the sexes was for quality of amenities—93.4% of males compared with 87.2% of females said that this aspect was satisfactory. Non-Qatari nationals also had a higher percentage of satisfaction on each domain than Qatari nationals, with the exception of autonomy, where Qataris were more satisfied. The largest difference was again in the quality of amenities aspect of responsiveness, where there was nearly a 10% difference between the two groups.

Al Rayyan had the highest ratings of satisfaction on all domains bar choice and confidentiality, while Doha had the lowest percentage satisfied on every domain except prompt attention. Only 75.9% of those in Doha were satisfied with the communication they experience, compared with 84.8% in the other regions of Qatar and 89% in Al Rayyan. There were only small differences between the satisfaction ratings of people who rated their own health as very good, good or moderate. A lower percentage of respondents who rated their health as bad or very bad were satisfied with the health care compared to those with better health, although there were only small numbers of respondents in these groups. Finally, the percentage of people who were satisfied with inpatient care on each domain was higher for those who stayed in a private hospital compared to a public hospital. The largest difference between public and private was in the domain of choice, while the smallest was in the domain of quality of amenities.

The domain responsiveness scores presented in Table 7.4b show a similar pattern to that seen for the percentage of people who were satisfied with inpatient care, with quality of amenities obtaining the highest score and prompt attention scoring the worst. One noticeable aspect is that the scores for each of the domains were similar to those for outpatient care. The overall average responsiveness score for inpatient care was calculated to be 76 out of 100, slightly below the score for outpatient care, which was 76.5.

Differences between subgroups of the population were much reduced when considering responsiveness score rather than satisfaction percentage. The differences between males and females and Qataris and non-Qataris were minimal, except for autonomy, where nationals gave a higher rating than non-nationals. Region did not have as great an effect either, although Doha still had the lowest ratings on each domain compared to the other two regions. Age and self-rated health were related to responsiveness rating. Ignoring the over 70s group, due to small sample sizes, rating increased with age for all domains except for prompt attention and, to a lesser degree, choice. Private hospitals again received a higher rating than public hospitals, with the largest discrepancy on the domain of dignity.

The final method of studying responsiveness indicated that very few individuals rated their own experience as worse than the vignette on the domains of prompt attention, communication, confidentiality, choice and quality of basic amenities. The ratings for the remaining domains, dignity and autonomy, indicate that 11.7% and 14.1% of respondents respectively gave a response for their own experience that was worse than their response for the vignette. For autonomy, under a third stated that their experience was better than that described in the vignette.

There were only small differences in the percentages of personal experience that were better, the same or worse than the rating given for the vignette by subgroup. A domain with a large difference between groups was the confidentiality domain for region, with Doha having a lower percentage of people who gave a higher score than the vignette and a much higher percentage of respondents who gave the same score. A similar difference between regions was seen for the prompt attention domain.

The differences in satisfaction by self-rated health status were reduced compared with the other methods of measuring responsiveness for most domains. This indicates that the experience of the respondents who were not healthy may not actually be worse than for healthy respondents, but simply they perceived it to be worse. Two domains that did not conform to this are communication and autonomy, where the percentage of people with a better rating than the vignette fell as health deteriorated. Large differences were seen on the autonomy and choice domains between public and private hospitals. Responsiveness on these two domains was much higher in private hospitals compared with public hospitals.

7.4.3 Responsiveness for inpatient and outpatient care

The previous sections analysed the responsiveness for both inpatient and outpatient care separately. This section discusses the two together to observe if there are similarities between the ratings for the two different types of care. These will also be compared against the ratings for the responsiveness vignettes, which relate to both inpatient and outpatient care.

Figure 7.1 shows stacked bar charts for each of the seven domains of responsiveness. For each domain the responses for inpatient and outpatient care are shown, next to the responses for the vignettes.

On all the domains except for cleanliness there was a higher percentage of people who rated the outpatient care as very good than in the same category for inpatient care. However, for the prompt attention and dignity domains there were actually a higher percentage of people who rated their inpatient care as better than moderate than for outpatient care. There were only very small differences in ratings between the two types of care aside from these differences.

The responses to the vignette questions were far more widely distributed than those given for inpatient and outpatient care. The most probable reason is that the vignette described a bad experience of care. Comparing the answers given to the care obtained the most recent time the respondent went to a health provider and the vignettes it is clear that the respondents, in general, obtained a much better quality of care than those described in the vignettes.

Figure 7.1 Responsiveness of inpatient and outpatient care and responses to vignettes

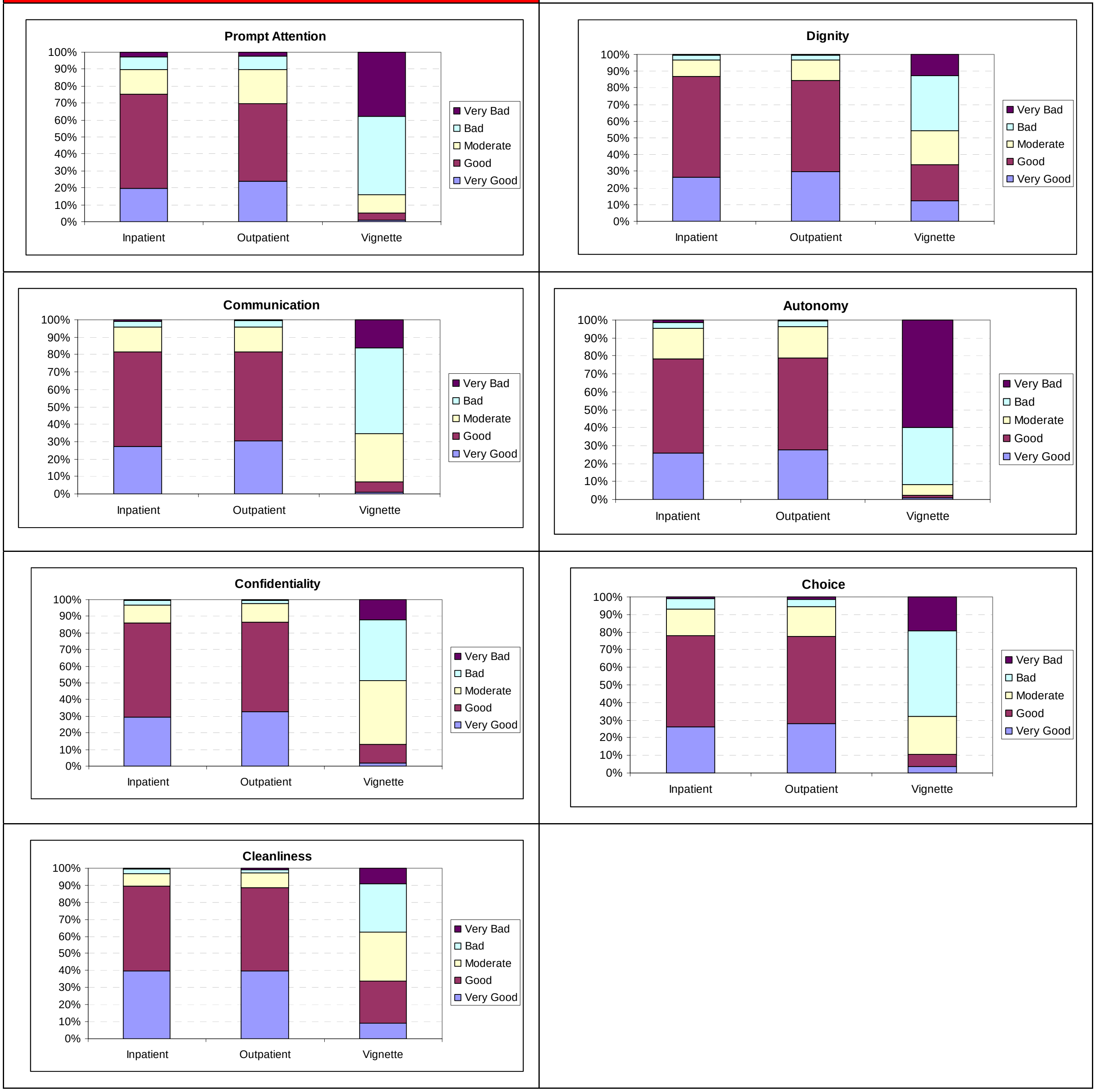

7.5 Health provider information

7.5.1 Most recent visit to a health provider

The respondents to the survey who had needed health care in the three years prior to the survey were asked about the most recent health provider that they visited. This was done separately for inpatient and outpatient visits. The respondents were also asked to report the type of the health provider

Table 7.5 indicates that the majority of inpatients, 91.6%, visited a public facility the most recent time that they went to hospital, while 8% visited a private facility. Males and Qataris were more likely to use a private hospital than females and non-Qataris respectively. There were differences between regions as well, with 11.4% of residents of Doha choosing a private hospital compared to 6.2% in Al Rayyan and 3.4% of those in the other regions of Qatar.

The most recent visits of outpatients are also shown in Table 7.5. The two most visited types of facility were public clinics and public hospitals, with 35% and 34.3% respectively. Private doctors, clinics and hospitals were visited by 14.6%, 8.4% and 6.6% of respondents respectively. For Qataris a higher percentage visited a public hospital than a public clinic, while for non-Qataris the converse was true. Nationals were also far more likely to have seen a private doctor than non-nationals. Individuals in the regions outside of Al Rayyan and Doha were least likely to use a private facility for an outpatients visit, and more likely to use a public clinic.

The type of health care provider visited the most recent time health care was obtained is shown in Table 7.6. Over 90% of the most recent health care providers were described as doctors. The only other type of healthcare professional described by a number of people was the dentist, whom 6% of respondents had visited.

Respondents who had visited a health facility in the last 12 months were asked for the reason why they used health care; whether it was for a chronic condition, a new condition or a new chronic condition. Table 7.7 presents the results for this for different subgroups of the population. Over two-thirds of the respondents stated that the reason for visiting a health provider was for a new condition, while 27.8% stated that it was for an ongoing chronic condition. There are differences in the reasons by nationality status, with 32.8% of Qataris stating that they visited for a chronic condition, compared with only 24.5% of non-Qataris. There was also a difference by region of Qatar. Respondents from Doha were more likely to state that their visit was due to a new visit than those in Al Rayyan, with 69.9% and 68.4% in this category. The percentage fell further in the other areas of Qatar, with only 63.7% of those in these areas visiting the health facility for a new condition.

As age rises the percentage of visits for a chronic condition increases, while the percentage of visits due to a new condition falls. Indeed, over three-quarters of 18–29 year olds visit the health facility for a new condition, compared with just over one-quarter of those aged 60–69. The percentage of chronic conditions increased as self-assessed health deteriorated. The percentage of those who visited the health provider for a new chronic condition also increased as the respondent stated that their health gets worse, except for those who stated that their health was very bad (although there were only a very few respondents in this category).

Table 7.5 Percentage distribution of the type of facility visited for last inpatient and outpatient visit

	Inpatient				Outpatient						
	Public	Private	Other	*N*	Private doctor	Private clinic	Private hospital	Public clinic	Public hospital	Other	*N*
**Sex**											
Male	89.3	10.2	0.5	*133*	13.9	9.2	6.0	35.9	33.5	1.5	*1083*
Female	92.3	7.3	0.4	*401*	15.2	7.8	7.1	34.4	35.2	0.4	*1363*
**Nationality status**											
Qatari	88.8	10.3	0.9	*240*	18.4	6.9	6.3	30.0	37.8	0.6	*961*
Non-Qatari	93.8	6.2	0.0	*294*	12.2	9.4	6.8	38.3	32.2	1.1	*1486*
**Region**											
Doha	88.6	11.4	0.0	*242*	14.2	9.2	7.4	35.6	32.8	0.8	*1254*
Al Rayyan	93.0	6.2	0.8	*195*	16.4	8.1	6.7	32.4	36.0	0.4	*833*
Other regions	95.9	3.4	0.8	*96*	11.8	6.5	3.6	39.7	36.1	2.4	*360*
**Age group**											
18 to 29	91.8	7.3	0.9	*149*	15.3	10.2	7.5	32.9	33.4	0.7	*539*
30 to 44	92.0	8.0	0.0	*297*	14.0	8.0	6.7	35.6	34.8	1.0	*1260*
45 to 59	90.1	10.0	0.0	*66*	16.2	8.0	5.8	36.4	32.6	1.0	*556*
60 to 69	88.6	6.4	5.0	*18*	8.3	6.0	7.5	36.1	40.9	1.1	*62*
70 or over	86.4	13.6	0.0	*5*	10.2	6.9	0.0	25.6	57.3	0.0	*29*
**Self-assessed health**											
Very good	92.5	7.6	0.0	*183*	15.9	8.7	5.9	38.0	30.6	0.9	*894*
Good	91.6	7.9	0.6	*250*	13.1	8.4	7.4	33.7	36.6	0.9	*1155*
Moderate	89.3	9.5	1.2	*73*	16.1	8.7	4.7	32.9	36.4	1.2	*338*
Bad	89.5	10.5	0.0	*23*	16.0	3.3	13.0	30.0	37.7	0.0	*52*
Very bad	100.0	0.0	0.0	*4*	16.9	0.0	0.0	38.1	45.1	0.0	*7*
**Wealth quintile**											
Poorest	89.2	9.6	1.2	*130*	14.6	8.8	8.9	35.6	30.6	1.6	*641*
< average	94.9	5.1	0.0	*133*	14.8	8.4	5.5	33.5	37.2	0.6	*521*
Average	88.9	11.1	0.0	*121*	14.6	9.4	4.7	36.1	34.4	0.9	*521*
> average	92.3	6.9	0.8	*83*	13.7	6.3	4.8	34.5	40.3	0.5	*417*
Richest	93.3	6.7	0.0	*67*	15.5	8.9	9.1	35.8	30.1	0.6	*345*
**Total**	**91.6**	**8.0**	**0.4**	***534***	**14.6**	**8.4**	**6.6**	**35.0**	**34.3**	**0.9**	*2453*

Table 7.6 Distribution of the last health care provider visited by the respondent

	Medical doctor	Nurse/ midwife	Dentist	Physiotherapist/ chiropractor	Traditional medicine	Pharmacist	Don't know	*N*
**Sex**								
Male	90.4	1.5	6.2	0.9	0.0	0.8	0.2	*1083*
Female	90.9	1.5	5.8	1.2	0.1	0.3	0.3	*1364*
**Nationality status**								
Qatari	88.0	1.7	8.4	0.9	0.1	0.6	0.3	*962*
Non-Qatari	92.4	1.4	4.4	1.2	0.0	0.4	0.2	*1486*
**Region**								
Doha	91.4	1.4	4.9	1.2	0.0		0.3	*1254*
Al Rayyan	90.1	1.2	7.4	1.1	0.1	0.0	0.2	*833*
Other regions	89.6	2.9	6.5	0.5	0.2	0.2	0.2	*360*
**Age group**								
18 to 29	88.0	1.9	8.7	0.5	0.0	0.6	0.4	*540*
30 to 44	91.9	1.1	4.9	1.2	0.1	0.5	0.3	*1260*
45 to 59	89.3	2.5	6.7	1.2	0.0	0.2	0.0	*556*
60 to 69	99.0	0.0	0.0	0.0	0.0	1.0	0.0	*62*
70 or over	98.1	0.0	0.0	1.9	0.0	0.0	0.0	*29*
**Self-assessed health**								
Very good	90.7	1.4	5.9	1.1	0.0	0.7	0.2	*894*
Good	89.8	1.9	6.7	0.9	0.0	0.4	0.3	*1156*
Moderate	93.1	0.9	4.1	1.4	0.2	0.2	0.2	*338*
Bad	94.9	0.0	3.8	1.4	0.0	0.0	0.0	*52*
Very bad	100.0	0.0	0.0	0.0	0.0	0.0	0.0	*7*
**Wealth quintile**								
Poorest	92.5	1.0	4.9	0.5	0.1	0.5	0.5	*641*
< average	90.5	1.0	6.3	1.6	0.0	0.6	0.0	*521*
Average	90.6	1.3	6.2	0.5	0.1	0.7	0.6	*522*
> average	89.8	1.1	7.0	1.7	0.0	0.4	0.0	*418*
Richest	88.8	4.3	5.8	1.2	0.0	0.0	0.0	*345*
**Total**	**90.5**	**1.5**	**6.0**	**1.0**	**0.0**	**0.5**	**0.3**	***2453***

7.5.2 Number of times visiting health providers

The Qatar World Health Survey asked those respondents who stated that they had used health care in the three years before the survey about the number of times that they had used the health system in the previous year only. Questions were asked separately about inpatient and outpatient care, and the results are shown in Table 7.7.

The mean number of inpatient visits, for those who had been admitted to hospital in the last year, is 1.3. The number of visits was higher for Qataris than non-Qataris, with 1.5 and 1.2 visits respectively. As expected, the number of inpatient stays increased as age rose, while the same occurred as self-rated health decreased. Over all respondents the percentage of people who had had at least one overnight stay in the previous 12 months in a hospital or a long-term care facility was 11.2%.

For outpatient visits, the mean number of visits in the year before the survey for those who have used the health system was 3.4. Females used the health system more than males, with 3.7 visits compared with 2.9 respectively, while Qataris again reported more visits than non-Qataris. Similar patterns were shown to the inpatient visits, with visits increasing as age rose and self-rated health deteriorated.

7.5.3 Reasons for health care

The respondents were also asked for the reasons why they used both inpatient and outpatient health care. They were asked to state which of 17 categories most fitted the reason why they were admitted to hospital or went for their most recent outpatient visit. The results are shown in Table 7.8.

For inpatient care, the main reason for staying in hospital overnight was for maternal and perinatal conditions, with just under half of the visits due to these reasons. Obviously, there were just females in this category, and there was a slightly higher percentage of non-Qataris than Qataris attending a hospital due to these reasons. There were a large percentage of respondents who stated that the reason for their inpatient stay was for a reason not listed on the questionnaire, with a larger percentage of males and Qataris than females and non-Qataris in this category. The third most common reason for an inpatient visit was for surgery, with 13.5% stating that they visited for this reason. A higher percentage of males than females stayed overnight in a hospital for surgery. There were only small percentages (under 7%) of respondents in the other categories.

Table 7.7 Distribution of the type of condition for most recent visit to a health care provider and mean number of times visiting health care in previous 12 months

		Type of condition for last visit				Mean number of times visiting health care			
		**Chronic**	**New condition**	**Both**	***N***	**Inpatient**	**Count**	**Outpatient**	***N***
**Sex**									
	Male	29.8	67.3	2.9	*1049*	1.4	*133*	2.9	*1069*
	Female	26.2	69.4	4.4	*1326*	1.3	*401*	3.7	*1343*
**Nationality status**									
	Qatari	32.8	62.6	4.6	*931*	1.5	*240*	3.7	*942*
	Non-Qatari	24.5	72.3	3.2	*1445*	1.2	*294*	3.1	*1470*
**Region**									
	Doha	25.8	69.9	4.3	*1229*	1.3	*242*	3.2	*1240*
	Al Rayyan	28.7	68.4	2.9	*804*	1.4	*195*	3.2	*816*
	Other regions	32.8	63.7	3.5	*342*	1.3	*96*	4.0	*357*
**Age group**									
	18 to 29	19.8	77.0	3.2	*530*	1.3	*149*	3.1	*532*
	30 to 44	24.1	72.5	3.4	*1223*	1.3	*297*	3.3	*1241*
	45 to 59	38.1	58.0	3.9	*533*	1.4	*66*	3.5	*551*
	60 to 69	63.4	25.9	10.8	*61*	1.7	*18*	5.5	*59*
	70 or over	65.6	26.0	8.4	*28*	2.0	*5*	4.2	*29*
**Self-assessed health**									
	Very good	20.8	77.3	1.9	*872*	1.3	*183*	2.7	*882*
	Good	26.9	68.9	4.2	*1122*	1.3	*250*	3.3	*1138*
	Moderate	44.7	49.2	6.0	*327*	1.5	*73*	5.1	*335*
	Bad	55.4	34.8	9.8	*48*	1.9	*23*	4.8	*50*
	Very bad	61.6	38.4	0.0	*7*	1.4	*4*	6.0	*7*
**Wealth quintile**									
	Poorest	26.9	69.7	3.4	*620*	1.3	*130*	3.2	*629*
	< average	27.0	70.0	3.0	*507*	1.3	*133*	3.3	*515*
	Average	27.6	67.9	4.5	*509*	1.4	*121*	3.7	*515*
	> average	27.9	68.4	3.7	*406*	1.3	*83*	3.2	*411*
	Richest	30.7	65.1	4.2	*333*	1.6	*67*	3.4	*342*
**Total**		**27.8**	**68.5**	**3.7**	***2375***	1.3	***534***	3.4	*2412*

Table 7.8 Distribution of the reason for visiting health care provider by sex and nationality

	Inpatient						Outpatient					
	Sex		Nationality		Overall		Sex		Nationality		Overall	
	Male	Female	Qatari	Non-Qatari		*N*	Male	Female	Qatari	Non–Qatari		*N*
Communicable disease	1.2	0.7	1.4	0.3	0.8	*5*	0.6	0.8	0.9	0.6	0.7	*17*
Maternal and perinatal conditions	0.0	63.7	45.2	51.5	48.6	*299*	0.2	15.9	8.2	9.5	9.0	*214*
Nutritional deficiencies	0.0	0.2	0.3	0.0	0.1	*1*	0.2	0.4	0.3	0.3	0.3	*7*
Acute conditions	10.7	4.9	2.6	9.6	6.3	*39*	37.2	29.3	29.1	35.2	32.8	*783*
Injury	8.9	1.1	2.9	3.0	2.9	*18*	3.3	1.4	1.3	2.9	2.2	*54*
Surgery	25.1	9.9	13.7	13.3	13.5	*83*	1.2	1.0	1.3	1.0	1.1	*26*
Sleep problems	0.0	0.1	0.2	0.0	0.1	*1*	0.0	0.1	0.1	0.0	0.0	*1*
Occupation/work related	–	–	–	–	–	*0*	0.0	0.1	0.0	0.1	0.1	*2*
Pain in joints	5.0	1.7	2.0	3.0	2.5	*15*	4.8	6.6	7.0	5.1	5.8	*139*
Heart problems	5.4	1.3	2.1	2.5	2.3	*14*	1.7	1.4	1.8	1.3	1.5	*36*
Breathing problems	6.7	2.6	5.4	1.9	3.6	*22*	2.2	3.5	4.5	1.9	2.9	*70*
High blood pressure	3.0	0.5	0.7	1.5	1.1	*7*	6.4	4.6	6.2	4.8	5.4	*129*
Stroke	0.4	0.6	0.6	0.6	0.6	*4*	0.0	0.1	0.1	0.0	0.0	*1*
Generalized pain	2.3	1.6	2.8	0.8	1.7	*11*	5.9	5.0	5.7	5.2	5.4	*130*
Depression or anxiety	–	–	–	–	–	*0*	0.0	0.6	0.6	0.2	0.3	*8*
Cancer	4.1	0.7	1.0	1.9	1.5	*9*	0.6	0.2	0.4	0.4	0.4	*10*
Other	27.1	10.4	19.2	10.1	14.4	*88*	35.6	29.0	32.5	31.6	31.9	*762*
**Total**	**100.0**	**100.0**	**100.0**	**100.0**	**100**	***616***	**100.0**	**100.0**	**100.0**	**100.0**	**100.0**	***2388***

There were large percentages of respondents who visited a health provider on an outpatient visit who went for an acute condition. Nearly one-third of respondents stated this, with a higher percentage of males than females stating that this was the reason, while 35.2% of non-Qataris went to the health provider for acute conditions, compared with 29.1% of Qataris. A large percentage (31.9%) of those who have visited a health provider in the last 12 months went for a condition not listed in the questionnaire. Maternal and perinatal conditions were the reason for 9% of the visits, again all females. The other conditions were not that common, with under 6% of respondents stating any of these other conditions.

7.6 Overall satisfaction with the health care system

A number of questions were asked regarding the individual's satisfaction with their interaction with the health system. These questions included their satisfaction with their most recent hospital stay and satisfaction with how health care services were run in the country. Also requested was a rating of how the health care system involved the individual in decisions and a rating of how health care is run in the country. The results are shown in Tables 7.9 to 7.12. All these questions were asked using a five point scale, ranging from very satisfied to very unsatisfied.

Table 7.9, regarding the most recent hospital stay, shows that 87.9% of the respondents were either satisfied or very satisfied with the care that they received, while only 7.2% were dissatisfied or very dissatisfied. A greater percentage of males were very dissatisfied compared with women, and the same can be observed for Qatari nationals compared to non-Qatari nationals. If satisfaction is studied for both sex and nationality, Qatari males were the most satisfied, with 40% stating that they were very satisfied, compared with less than 35% for Qatari females and non-Qataris. Interestingly, Qatari males were also the least satisfied, with over 15% stating that they were either dissatisfied or very dissatisfied, compared with 9.5% of Qatari females, 5.8% of non-Qatari males and 3.6% of non-Qatari females. A larger percentage of those who live in the Al Rayyan region stated that they were satisfied or very satisfied with the most recent inpatient treatment compared with the other two reasons. The largest difference between groups is between those who used private and public hospitals. Over 98% of individuals using a private hospital were in the top two categories of satisfaction, compared with only 87% of those using a public hospital.

Table 7.9 Percentage distribution of satisfaction with last hospital stay

	Very satisfied	Satisfied	Neither satisfied nor unsatisfied	Dissatisfied	Very dissatisfied	*N*
**Sex**						
Male	36.2	47.8	6.6	5.9	3.5	*133*
Female	34.2	55.0	4.4	5.6	0.9	*401*
**Nationality status**						
Qatari	35.7	49.0	4.6	8.3	2.4	*240*
Non-Qatari	33.9	56.6	5.2	3.5	0.8	*294*
**Gender and nationality**						
Qatari male	40.0	40.8	3.7	10.6	4.9	*49*
Qatari female	34.5	51.1	4.8	7.7	1.8	*191*
Non-Qatari male	34.0	51.9	8.3	3.2	2.6	*84*
Non-Qatari female	33.9	58.5	3.9	3.6	0.0	*210*
**Region**						
Doha	32.6	52.8	7.1	5.8	1.6	*242*
Al Rayyan	36.6	54.5	3.1	4.0	1.8	*195*
Other regions	36.2	51.6	3.0	8.6	0.6	*96*
**Age group**						
18 to 29	25.6	60.7	4.3	8.1	1.4	*149*
30 to 44	38.3	50.3	5.7	4.7	1.1	*297*
45 to 59	32.4	58.5	3.3	4.1	1.8	*66*
60 to 69	55.7	26.6	4.8	3.1	9.8	*18*
70 or over	50.6	25.5	0.0	24.0	0.0	*5*
**Self-assessed health**						
Very good	38.3	53.0	2.5	4.0	2.2	*183*
Good	32.7	53.8	7.6	5.6	0.3	*250*
Moderate	32.2	56.5	2.1	6.5	2.8	*73*
Bad	35.0	42.5	4.9	15.5	2.1	*23*
Very bad	43.6	27.7	0.0	12.2	16.4	*4*
**Wealth quintile**						
Poorest	32.5	56.2	6.2	4.0	1.1	*130*
< average	30.7	53.2	8.2	7.4	0.5	*133*
Average	33.3	54.3	3.2	8.2	1.1	*121*
> average	38.0	51.8	2.3	4.3	3.5	*83*
Richest	45.7	47.0	2.2	2.6	2.6	*67*
**Type of hospital**						
Public	34.3	52.7	5.1	6.2	1.7	*489*
Private	41.2	57.3	1.5	0.0	0.0	*43*
Other	0.0	75.5	24.5	0.0	0.0	*2*
**Total**	**34.7**	**53.2**	**4.9**	**5.7**	**1.5**	***534***

The questions on health care service satisfaction and ratings were asked only to those who had received outpatient health care in the 12 months before the survey and thereby excluded those who did not have need to interact with the health system. Therefore there is a large amount of missing data, shown in Tables 7.10 to 7.12. Only those who responded to the questions were included in the following analyses. The large amount of missing data may bias the results, as even though those who did not answer these questions had not used the health system for a number of years, if at all, they still would have an opinion on how the system was run. The absence of the non-users therefore means that the responses are representative of only those who have used the health system recently, and not the full population of Qatar.

Satisfaction with the way in which the health care services were run is shown in Table 7.10. The majority of respondents stated that they were satisfied with the system, with 61.8% in this category. The percentage stating that they were very satisfied is 18.9%. There were fewer than 10% of people who said that they were dissatisfied, and 2.1% who were very dissatisfied. In general, males were more satisfied than females, while there was a large difference between Qataris and non-Qataris. Qatari nationals were less pleased with the way that health care was run, with 17% in the dissatisfied categories, compared to only 7.4% of non-Qatari nationals. Non-Qatari males were the most satisfied, with Qatari females the least. As age increased the satisfaction with the health system also increased, with a higher percentage of people who are very satisfied and a lower proportion who are dissatisfied.

A further question asked was “how would you rate the way health care in your country involves you in deciding what services it provides and where it provides them?” The results from this question are displayed in Table 7.11. The general ratings provided regarding involvement were slightly worse than the satisfaction with the way services were run (shown in Table 7.10). The majority of people say that the involvement was good, with 55.9% in this category. The percentages of those who said that the involvement was very good or moderate were similar, with 16.5% and 15.8% respectively. Almost 12% of respondents thought that the involvement was bad or very bad.

As has occurred in the previous tables, Qatari nationals rated this aspect of the health service as worse than non-Qatari nationals. Over 16% of nationals stated that the involvement was bad or very bad, compared with about 9% of non-nationals. The residents of regions outside of Doha and Al Rayyan rated this aspect much higher than those who live in these areas.

Finally, there was a question about how health care was run in the country overall and how satisfied the individual was about health care. Table 7.12 shows the results obtained from this question. The most common category for the response was the satisfied category, with just under two-thirds of respondents answering this. There were 17.1% of individuals who stated that they are very satisfied, while 11.4% say that they were either dissatisfied or very dissatisfied. Non-Qataris were more likely to be satisfied with the health care provided, while Qataris were more likely to be dissatisfied. The percentage of people in the bottom two groups for Qataris was 17.8%, in comparison with 7.2% for non-Qataris. Satisfaction increased with age, and dissatisfaction increased as self-rated health decreased.

Table 7.10 Percentage distribution of the satisfaction with health care services

	Very satisfied	Satisfied	Neither satisfied nor unsatisfied	Dissatisfied	Very dissatisfied	*N*	Missing
**Sex**							
Male	20.4	59.8	8.5	9.5	1.8	*1376*	42.8
Female	17.7	63.3	8.0	8.7	2.4	*1713*	27.0
**Nationality status**							
Qatari	16.7	56.9	9.4	13.6	3.4	*1208*	24.7
Non-Qatari	20.3	64.9	7.4	6.1	1.3	*1881*	40.2
**Gender and nationality**							
Qatari male	18.2	53.4	9.7	15.7	2.9	*464*	30.2
Qatari female	15.8	59.0	9.2	12.3	3.7	*744*	20.8
Non-Qatari male	21.5	63.1	7.8	6.4	1.2	*912*	47.6
Non-Qatari female	19.1	66.7	7.0	5.9	1.4	*969*	31.1
**Region**							
Doha	17.7	62.7	9.0	8.8	1.8	*1566*	39.3
Al Rayyan	18.1	61.6	7.5	9.8	3.0	*1045*	28.8
Other regions	24.5	59.0	7.0	8.4	1.1	*478*	32.3
**Age group**							
18 to 29	17.7	60.7	9.5	10.3	1.8	*690*	37.0
30 to 44	17.5	62.6	7.6	9.9	2.5	*1600*	32.6
45 to 59	22.3	61.0	8.6	6.5	1.6	*685*	39.0
60 to 69	24.0	62.2	6.2	6.7	0.9	*84*	33.5
70 or over	27.6	59.7	8.5	2.2	2.0	*30*	13.8
**Self-assessed health**							
Very good	24.5	58.9	6.5	8.1	2.0	*1194*	43.1
Good	15.5	64.1	9.0	9.6	1.9	*1428*	30.7
Moderate	13.6	64.7	9.4	9.8	2.4	*393*	22.2
Bad	21.0	47.9	13.4	12.5	5.3	*64*	12.8
Very bad	20.7	56.3	10.2	0.0	12.8	*11*	12.1
**Wealth quintile**							
Poorest	18.9	61.2	8.2	9.8	2.0	*781*	36.0
< average	20.0	61.4	7.8	9.5	1.4	*651*	35.7
Average	17.8	62.3	8.8	8.3	2.8	*677*	33.4
> average	17.9	63.6	9.1	7.7	1.8	*533*	36.3
Richest	20.0	60.4	6.8	10.0	2.7	*446*	32.9
**Total**	**18.9**	**61.8**	**8.2**	**9.1**	**2.1**	***3089***	**35.0**

Table 7.11 Percentage distribution of the ability to decide which services are provided and where services are provided rating

		Very good	Good	Moderate	Bad	Very bad	*N*	Missing
**Sex**								
	Male	17.4	54.2	16.6	8.9	3.0	*1376*	42.8
	Female	15.8	57.3	15.2	9.2	2.6	*1713*	27.0
**Nationality status**								
	Qatari	16.2	51.1	16.3	12.6	3.8	*1208*	24.7
	Non-Qatari	16.7	59.0	15.4	6.8	2.1	*1881*	40.2
**Gender and nationality**								
	Qatari male	17.2	48.3	18.0	12.6	3.9	*464*	30.2
	Qatari female	15.5	52.9	15.3	12.7	3.7	*912*	20.8
	Non-Qatari male	17.5	57.1	15.9	7.0	2.5	*744*	47.6
	Non-Qatari female	16.0	60.7	15.0	6.5	1.8	*969*	31.1
**Region**								
	Doha	14.7	55.5	17.6	9.6	2.7	*1566*	39.3
	Al Rayyan	16.5	55.6	14.6	10.0	3.3	*1045*	28.8
	Other regions	22.4	58.0	12.5	5.1	2.0	*478*	32.3
**Age group**								
	18 to 29	16.4	56.4	16.3	9.5	1.3	*690*	37.0
	30 to 44	14.7	55.8	16.0	9.9	3.5	*1600*	32.6
	45 to 59	19.8	55.3	15.6	6.7	2.6	*685*	39.0
	60 to 69	19.0	62.5	9.7	7.2	1.7	*84*	33.5
	70 or over	30.5	44.8	13.7	8.9	2.0	*30*	13.8
**Self-assessed health**								
	Very good	22.0	52.9	13.6	9.0	2.5	*1194*	43.1
	Good	13.1	58.6	16.5	9.2	2.7	*1428*	30.7
	Moderate	11.8	58.4	18.3	8.3	3.3	*393*	22.2
	Bad	20.4	38.0	25.2	11.2	5.3	*64*	12.8
	Very bad	10.9	49.4	16.7	16.8	6.3	*11*	12.1
**Wealth quintile**								
	Poorest	17.8	54.3	16.5	8.7	2.7	*781*	36.0
	< average	15.6	59.2	13.7	9.4	2.0	*651*	35.7
	Average	17.5	54.7	17.1	7.5	3.3	*677*	33.4
	> average	14.6	57.6	15.6	9.6	2.6	*533*	36.3
	Richest	16.2	53.9	15.7	10.9	3.3	*446*	32.9
**Total**		**16.5**	**55.9**	**15.8**	**9.1**	**2.8**	***3089***	**35.0**

Table 7.12 Percentage distribution of the satisfaction with the way health care is run in the country

	Very satisfied	Satisfied	Neither satisfied nor unsatisfied	Dissatisfied	Very dissatisfied	*N*	Missing
**Sex**							
Male	18.0	61.9	8.5	9.5	2.2	*1376*	42.8
Female	16.5	63.6	8.9	9.5	1.6	*1713*	27.0
**Nationality status**							
Qatari	15.8	56.6	9.9	14.6	3.2	*1208*	24.7
Non-Qatari	18.0	66.9	7.9	6.2	1.0	*1881*	40.2
**Gender and nationality**							
Qatari male	15.5	55.4	9.7	15.3	4.1	*464*	30.2
Qatari female	16.1	57.3	9.9	14.1	2.6	*744*	20.8
Non-Qatari male	19.3	65.2	7.8	6.5	1.2	*911*	47.6
Non-Qatari female	16.8	68.4	8.1	5.9	0.9	*968*	31.2
**Region**							
Doha	15.5	63.7	10.1	9.3	1.6	*1566*	39.3
Al Rayyan	17.2	61.8	7.6	10.7	2.6	*1045*	28.8
Other regions	22.5	62.4	6.5	7.4	1.3	*478*	32.3
**Age group**							
18 to 29	15.3	64.5	8.6	9.8	1.8	*690*	37.0
30 to 44	16.3	62.3	8.6	10.5	2.3	*1600*	32.6
45 to 59	19.7	62.9	8.6	7.5	1.2	*685*	39.0
60 to 69	23.1	61.1	10.0	4.9	0.9	*84*	33.5
70 or over	27.6	57.4	10.7	2.3	2.0	*30*	13.8
**Self-assessed health**							
Very good	23.1	59.7	6.8	8.7	1.7	*1194*	43.1
Good	13.3	66.2	9.4	9.3	1.8	*1428*	30.7
Moderate	12.6	62.1	10.9	12.2	2.2	*393*	22.2
Bad	20.3	51.1	15.3	10.7	2.6	*64*	12.8
Very bad	10.9	56.6	10.2	9.6	12.8	*11*	12.1
**Wealth quintile**							
Poorest	17.6	62.1	9.1	9.7	1.6	*855*	36.0
< average	16.4	64.9	8.1	9.2	1.4	*739*	35.9
Average	18.0	61.9	8.7	9.0	2.4	*603*	33.5
> average	16.9	63.8	8.9	8.5	1.9	*504*	36.3
Richest	16.5	61.3	8.6	11.4	2.2	*388*	32.7
**Total**	**17.1**	**62.8**	**8.7**	**9.5**	**1.9**	***3089***	**35.0**

N.B. % Given as if there was no missing data - Missing is the percentage of full sample not answering this question

8. Health expenditure, family support networks and transfers

8.1 Introduction

Many countries around the world are reforming their health systems in an effort to improve the efficiency and management of health services as well as the distribution of these services, particularly among the poor. With health systems growing in scope and complexity, policy-makers need tools to better manage their health care resources (WHO regional policy briefs). Knowledge about household expenditure in general coupled with knowledge of expenditure on health is important to contribute to better-informed policy choices in the area of health financing.

This chapter presents the results regarding expenditure on health from Qatar. Financial resources to pay for healthcare may be increased through the receipt of monetary and in-kind transfers to the households. Alternatively, the household may provide financial aid to people not living in the household or provide personal or health care to others, thereby reducing the need to formal health care use. These aspects of healthcare will also be explored in this chapter. The responses to the questions analysed in this chapter were all asked to a key respondent in the household, and the unit of analysis for most of this chapter is the household, and not the individual. All amounts shown in this chapter have been standardized to account for different household sizes. Therefore the financial amounts quoted are on a per capita basis.

8.2 Health expenditure

Health expenditure comprises both government and private sector spending, alongside household expenditure. In 2004 it was estimated that in Qatar 77.7% of the total expenditure on health was by the government (WHOSIS, 2008), while private expenditure represented the rest of health expenditure. As a percentage of private expenditure, out-of-pocket costs represented 86.4%. This is the percentage of the costs that are direct outlays by households and includes direct payments to public and private health care providers (WHO, 2006).

Health insurance is usually an important part of a health system, and in the survey questions regarding health insurance were asked. However, only 14 respondents in five different households stated that they were covered by health insurance. Therefore, insurance cover was not analysed further in this chapter.

Concern about the links between ill-health and impoverishment has placed health at the centre of development agencies' poverty reduction targets and strategies (DFID, 1999; World Bank, 2000). Health systems can deliver health services, preventive and curative, that can make a difference to peoples' health. However, accessing these services can lead to individuals having to pay catastrophic proportions of their available income and push many households into poverty (Xu et al, 2003). The Qatar World Health Survey allows the investigation of health expenditure. A number of questions were asked about spending on health care, including a breakdown of the costs into the component parts, such as inpatient and outpatient costs, dentists, drugs, health products and tests.

Regarding expenditure on health, there are a number of terms that will be used throughout this chapter. These terms are explained in Box 8.1.

BOX 8.1. Definitions of key expenditure concepts

Subsistence spending is the minimum requirement to maintain a basic life in a society. A poverty line is used in the analysis as subsistence spending.

Household capacity to pay is defined as a household's non-subsistence spending.

Household consumption expenditure (EXP) comprises both monetary and in-kind payment on all goods and services and the money value of the consumption of home-made products.

Food expenditure is the amount spent on all foodstuffs by the household plus the value of the family's own food production consumed within the household. It excludes expenditures on alcoholic beverages, tobacco and food consumption outside the home (e.g. hotels and restaurants).

Poor a household is considered poor when its total household expenditure is smaller than its subsistence spending

Out-of-pocket health payments (OOP) are payments made by households at the point they receive health services. It includes doctor's consultation fees, purchases of medication and hospital bills. Spending on alternative and/or traditional medicine is included. Expenditure on health related transportation and special nutrition is excluded. Insurance reimbursements are also excluded.

Impoverished a non-poor household is impoverished by health payments when it becomes poor after paying for health services

Catastrophic health expenditure occurs when a household's total out-of pocket health payments equals or exceeds 10% of the household's capacity to pay or non-subsistence spending

8.2.1 Out-of-pocket expenditure on health

Table 8.1 shows a summary of the distribution of health expenditure in the month before the survey over different groups of the Qatari population. It shows that the mean household expenditure (on everything, not only healthcare) was QR8912 (US$2450). Expenditure was over double for Qataris than non-Qataris, and there was great inequity between the bottom expenditure decile and the top decile. The top decile spent about 32 times the amount that the bottom decile spent.

A Poor household is defined when subsistence spending is higher than its total expenditure. These households spent on average QR1414 (US$389), compared to the non-poor households, who spent QR8713 (US$2395). Overall, 13.6% of households were estimated to be poor; 14.4% of non-Qatari households were poor, compared to only 11.9% of Qatari households. There were a larger percentage of poor households outside Doha.

Capacity to pay is the amount of money that a household has spare after spending on subsistence is subtracted from total spending. Table 8.1 shows the out-of-pocket expenditure as a percentage of capacity to pay (OOP % non-subsistence). The average percentage is 6.7%. Figure 8.1 shows this in graphical format. It indicates that in about 81% of households the out-of-pocket expenditure was less than 10% of capacity to pay. However, in 5% of households the percentage of out-of-pocket expenditure was above 40%. By definition, households in this category have faced a catastrophic payment. There were 8% of households spending between 10% and 20% of non-subsistence spending on healthcare, while 6% spend between 20% and 40%. Qatari nationals paid a larger percentage of their non-subsistence spending towards healthcare payments than non-nationals, spending 9.5% and 5.3% respectively.

Table 8.1 Distribution of health expenditure across different population groups

Groups	Expenditure Qatari riyals	% poor	% impoverished	% catastrophic	OOP % EXP	OOP % non-subsistence spending	Mean out-of-pocket health payments (OOP) in local currency
**Nationality status**							
Qatari	13675	11.9	1.6	7.1	7.2	9.5	1364
Non-Qatari	6482	14.4	1.5	3.7	3.8	5.3	273
**Region**							
Doha	8574	11.2	1.6	4.5	4.7	6.3	505
Al Rayyan	9513	16.5	1.3	5.7	5.5	7.6	957
Other regions	8903	16.2	1.7	4.6	4.7	6.6	482
**Catastrophic payment made?**							
No	8783	13.4	0.7		2.9	3.9	326
Yes	11426	16.5	17.3		45.5	61.8	6819
**Poor household?**							
No	10089		1.8	4.7	5.0	6.7	733
Yes	1414		0.0	5.9	4.7	6.8	63
**Expenditure decile**							
1st	1118	100.0	0.0	5.4	4.7	6.6	44
2nd	2541	35.5	10.6	7.3	4.4	7.7	117
3rd	3594	0.0	2.5	3.1	3.5	5.5	123
4th	4477	0.0	1.3	5.1	4.2	6.7	191
5th	5457	0.0	0.1	4.0	3.9	5.6	233
6th	6507	0.0	0.0	4.8	5.0	7.0	345
7th	7673	0.0	0.4	4.3	5.0	6.4	430
8th	9411	0.0	0.2	4.2	5.2	6.5	515
9th	12883	0.0	0.2	3.7	5.7	6.8	771
10th	35476	0.0	0.0	6.7	7.8	8.5	3650
**Total**	**8912**	**13.6**	**1.5**	**4.9**	**4.9**	**6.7**	**642**

Figure 8.1. Percentage of out-of-pocket expenditure as a share of capacity to pay

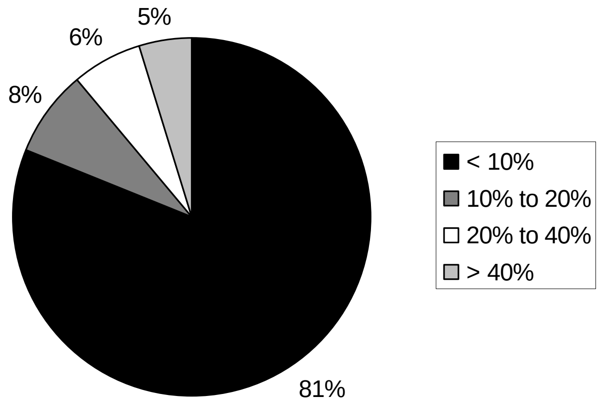

8.2.2 Catastrophic spending on health

As noted earlier, catastrophic spending on health occurs when a household must reduce its basic expenses over a period of time in order to cope with the medical payments of one or more of its members. Poorer households in Qatar tended to spend a larger proportion of their income on health care as there is little insurance coverage in the country. Over all households, almost 5% were identified as having made a catastrophic health payment. Households that did make these payments spent, on average, QR6819 (US$1874) on health care, compared with QR326 (US$90) for households that did not make a catastrophic payment. The major differences were seen in inpatient costs and on drug expenditure, where spending was just under 50 times as much in catastrophic payment households compared to non-catastrophic ones (see Table 8.2).

8.2.3 Impoverishment

It is estimated that 1.5% of households were impoverished due to healthcare payments. This is when non-poor households become poor due to the scale of the health payments required. Figure 8.2 displays the percentage of households with catastrophic payments and the percentage of households impoverished by the health treatment costs, by expenditure deciles.

Catastrophic expenditure occurred in each of the expenditure deciles, with no large differences between the deciles. However, households in the second lowest expenditure decile were more likely to be impoverished than in other deciles. There was no impoverishment in the lowest decile as all households in this decile are already poor and therefore cannot be impoverished further; 35.5% of the households in the second decile were also poor prior to the consideration of healthcare payments.

Table 8.2 Structure of out-of-pocket health payments

Group		Total	Inpatient	Outpatient	Traditional	Drugs	Other	N of HH
**Nationality status**								
	Qatari	1364	138	180	21	458	566	*1536*
	Non-Qatari	273	31	42	2	62	136	*3013*
**Region**								
	Doha	505	81	88	5	92	238	*2499*
	Al Rayyan	957	39	100	11	443	365	*1412*
	Other regions	482	77	67	13	58	266	*639*
**Catastrophic**								
	No	326	21	68	5	61	171	*4329*
	Yes	6819	982	489	69	2828	2452	*221*
**Poor**								
	No	733	77	101	9	224	321	*3933*
	Yes	63	4	14	1	16	28	*617*
**Expenditure decile**								
	1st	44	2	11	0	11	21	*456*
	2nd	117	8	19	2	31	56	*454*
	3rd	123	15	19	3	26	60	*457*
	4th	191	8	32	2	45	105	*454*
	5th	233	23	53	2	55	99	*455*
	6th	345	44	79	1	69	152	*455*
	7th	430	45	83	10	76	217	*455*
	8th	515	37	101	27	109	240	*455*
	9th	771	61	150	15	132	413	*455*
	10th	3650	433	342	18	1406	1452	*455*
**Total**		**642**	**67**	**89**	**8**	**196**	**281**	***4550***

Figure 8.2. Percent distribution of households with catastrophic expenditure and impoverishment

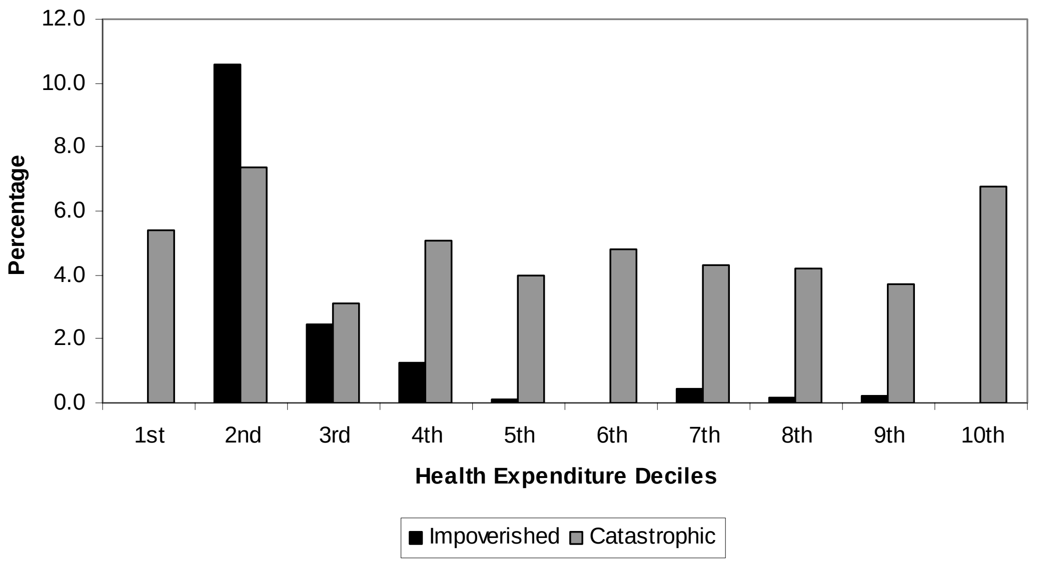

8.2.4 Structure of out-of-pocket health payments

The total expenditure on health treatment incurred by the households in the survey is shown in Table 8.2. On average, households spent QR642 (US$176)on treatment. The breakdown of these payments is also shown in the table. The main component of expenditure on health treatment is classified into the "other" category, totalling QR281 (US$77). This category includes expenditure on dentists, health care products such as glasses and hearing aids, and diagnostic and laboratory tests. Studying the breakdown of this category further indicates that the largest component is dental costs, representing over 50% of expenditure. The average spent on drugs in the 30 days prior to the survey was QR196 (US$54), while inpatient and outpatient costs were lower at QR67 (US$18) and QR89 (US$24) respectively. Only QR8 (US$2) was spent on average on traditional or alternative healers. The percentage of out-of-pocket expenditure for each category is shown in Figure 8.3.

Figure 8.3 Structure of out-of-pocket expenditure on health payments

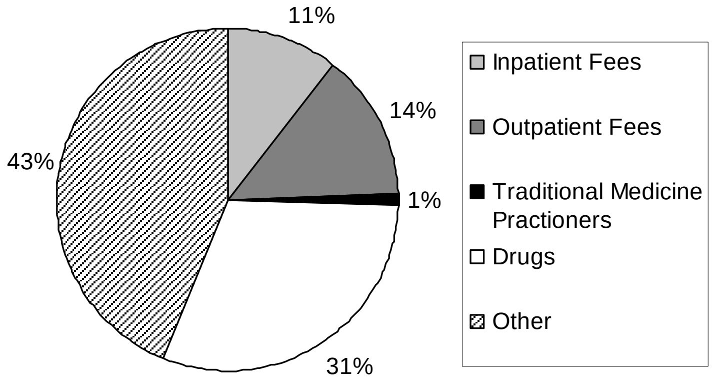

The average health treatment expenditure for Qatari nationals far exceeded the payments made by non-Qataris. On average, nationals paid QR1364 (US$375) on health care, compared to only QR273 (US$75) for the non-Qatari nationals. Qataris paid more for every type of health-care, including 12 times as much, on average, on drugs. The mean payments differed by region too, with Al Rayyan spending QR957 (US$263), compared to Doha and the other regions of Qatar where payments were around QR500 (US$137). In Al Rayyan the households spent less on inpatient care, but far more on drugs than the other regions.

The different types of health care expenditure were also calculated by whether the household was poor, suffered catastrophic health care payments and by expenditure decile. Households which experienced catastrophic health care expenditure spent, on average, QR6819 (US$1875) in the previous month, compared to QR326 (US$90) for households which did not experience such an event. Poor households spent far less than non-poor households on health care treatments. The average figure spent by poor households was QR63 (US$17), as opposed to QR733 (US$202) in non-poor households. As expected, as general expenditure rose, spending on health care also rose. This was a fairly steady rise until the final decile, where health treatment expenditure increased markedly, from QR771 (US$212) for the ninth decile to QR3650 (US$1003) in the top decile. This large increase was mainly driven by increases in drug and inpatient spending.

8.2.5 Sources of health financing

The sources of funding for the health care payments were collected in the survey, and this was categorized by nationality status, region and other groupings. Households could give multiple sources for where the money for the health care costs was obtained, but the responses indicate that a number of households did not give any sources of financing at all. Hence in Table 8.3, which lists the sources of financing, the percentages do not sum to 100.

Overall, 75.8% of the financing for healthcare came from current income. Only 3.5% came from household savings, and 1.5% from borrowing from relatives and friends.

Less than 1% of the financing was attributed to having to sell items, borrowing from someone who was not a friend or family or from insurance. Qatari nationals were more likely to fund expenditure from savings than non-Qatari nationals, and a higher percentage also funded the health treatment through selling items. The differences by regions indicated that households the other regions of Qatar were more likely to pay for healthcare from savings, selling items, borrowing from friends and relatives and from insurance than households in Doha and Al Rayyan.

The sources of healthcare financing were also broken down by the percentage of out-of-pocket costs as a percentage of non-subsistence spending (see Box 8.1), whether a hospitalization had occurred in the household and by healthcare expenditure decile. As the proportion of out-of-pocket costs as a percentage of non-subsistence spending increased, households were more likely to pay for the healthcare from their savings, by selling household items and by borrowing money (both from friends and relatives and from other sources). Also, the same is seen if a household had had a family member in hospital, with more use of sources which were not current income. Households with a hospitalization also relied on insurance payments more than households without. Studying the patterns of health service expenditure sources by expenditure decile highlights few strong trends. There is some evidence that as expenditure increased the percentage of households using savings increased, while the proportion of those borrowing money, from any source, decreased.

8.3 Household and family support networks and transfers

An aspect of a household's ability to cope with unexpected health payments is in the receiving and giving of financial and other in-kind assistance. Households can send a significant proportion of their income to relatives in different households and countries. Alternatively, some households will obtain transfers of assistance from elsewhere. This could be from family and kin, the local community or from the government. All these transfers in and out of the household will influence the amount of money available for healthcare payments. However, support networks are not only related to financial help or donation. Care can be given to people with a physical or mental illness or disability, either in the household or in a different household, and this care can take various forms. This section will discuss these different forms of support. For all these sections the individual answering the questions on behalf of the household may not know whether transfers are given or received. These responses have been analysed as "don't knows" and are included where applicable in subsequent tables.

8.3.1 Transfers out of the household

Financial aid and in-kind care given to family outside of each household, as well as aid to other kin, neighbours and the community, were included in the survey questionnaire. The amount given to each of these categories of recipient was also recorded. Table 8.4 presents the responses to these questions.

Table 8.3 Financial sources used by households to pay for health services

	Groups	Savings	Sold items	Borrow from relatives and friends	Borrow from others	Insurance	Current income	Others	*N*
**Nationality status**									
	Qatari	6.3	1.1	2.0	0.9	0.2	72.2	2.2	*1537*
	Non-Qatari	2.1	0.5	1.2	0.9	1.1	77.6	1.2	*3013*
**Region**									
	Doha	3.9	0.5	1.0	0.7	0.7	75.7	1.7	*2499*
	Al Rayyan	2.4	0.8	1.6	0.8	0.5	75.7	1.1	*1412*
	Other regions	4.4	1.1	2.9	1.7	1.8	76.6	2.0	*639*
**OOP % non-subsistence spending**									
	less than 10%	3.0	0.5	1.1	0.6	0.8	73.3	1.4	*3686*
	10-20%	6.8	1.3	2.4	1.0	1.1	87.1	2.6	*359*
	20-40%	5.4	2.1	3.9	2.2	0.8	85.3	1.1	*284*
	above 40%	5.4	1.6	3.5	3.2	0.0	86.8	2.1	*221*
**Hopitalization**									
	No	3.3	0.5	1.1	0.6	0.7	74.9	1.4	*3751*
	Yes	4.7	1.7	3.3	2.3	1.6	79.9	2.0	*799*
**Expenditure decile**									
	1st	1.8	0.0	2.2	1.2	0.1	70.1	1.0	*456*
	2nd	2.7	1.1	2.1	1.4	0.3	74.1	0.8	*454*
	3rd	2.9	0.5	2.4	1.6	0.2	74.8	1.7	*457*
	4th	2.6	1.4	0.7	1.2	0.5	77.2	0.9	*454*
	5th	3.1	0.7	1.2	0.8	1.8	72.2	1.2	*455*
	6th	2.3	0.3	1.5	0.2	1.1	78.8	1.0	*455*
	7th	4.1	0.2	0.4	0.1	1.6	75.4	2.3	*455*
	8th	4.2	1.1	1.0	0.7	0.0	77.4	2.0	*455*
	9th	4.1	1.1	1.8	0.2	0.9	80.7	1.4	*455*
	10th	7.5	0.5	1.4	1.3	1.8	77.4	3.2	*455*
**Total**		**3.5**	**0.7**	**1.5**	**0.9**	**0.8**	**75.8**	**1.5**	***4550***

Table 8.4 Financial transfers out of the household

	Transfers to close family					Transfers to friends, community and neighbours				
	**% Transfers**	**% Don't know**	***N***	**Average amount**	***N***	**% Transfers**	**% Don't know**	***N***	**Average amount**	***N***
**Nationality status**										
Qatari	29.2	2.1	*1615*	15515	*472*	48.7	2.2	*1615*	4891	*787*
Non-Qatari	37.8	0.5	*3164*	12689	*1195*	29.0	0.7	*3164*	4622	*917*
**Region**										
Doha	37.0	0.9	*2600*	13862	*962*	33.4	1.3	*2600*	4517	*869*
Al Rayyan	29.7	1.2	*1471*	11096	*437*	36.0	1.0	*1471*	4666	*530*
Other regions	38.0	1.1	*707*	16042	*269*	43.1	1.6	*707*	5538	*305*
**Wealth quintile**										
Poorest	33.6	0.8	*1214*	7280	*408*	25.2	1.1	*1214*	3035	*306*
< average	31.1	1.0	*1005*	10571	*313*	32.2	1.0	*1005*	3728	*324*
Average	36.8	1.2	*1014*	10566	*373*	35.9	1.6	*1014*	3474	*364*
> average	35.8	0.9	*875*	20550	*313*	42.3	1.0	*875*	4975	*371*
Richest	38.8	1.2	*670*	22433	*260*	50.9	1.5	*670*	8352	*341*
**Total**	**34.9**	**1.0**	***4779***	**13489**	*1625*	**35.7**	**1.2**	***4779***	**4746**	***1704***

Overall, nearly 35% of households stated that they provided financial aid to family and roughly the same number stated that they gave financial assistance to other recipients. Almost 38% of non-Qataris provided financial aid to family members, compared to 29% of Qataris. However, a higher percentage of Qataris gave aid to sources outside of family than the non-nationals. Al Rayyan was the region that the lowest percentage of households give aid to family members, but Doha was the region with the lowest proportion of household giving aid to other beneficiaries. The households in the poorest quintile are the least likely to give money to both family and other sources.

The average amount of money per year given to family was nearly QR13 500 (US$3711), while the average given to other sources was almost QR4800 (US$1320). Even though fewer Qatari nationals gave aid to family, those that did gave on average a higher amount than non-Qatari nationals. The average amount given to other recipients outside of the close family was very similar for each nationality status. By region, other areas of Qatar outside of Doha and Al Rayyan gave the most money, on average. These other regions gave about QR16 000 (US$4398) to family, compared to QR14 000 (US$3849) in Doha and QR11 000 (US$3024) in Al Rayyan. The amount given to both family and other sources rose as wealth quintile increased.

8.3.2 Transfers into the household

Table 8.5 shows that the percentage of households who say that they received support from any of the three sources of family and kin, the community or the government was much lower than those stating that they gave assistance to others. Only 4.1% of households received transfers from family and kin, 1.8% from the community and 2.4% from the government.

Qatari nationals were the main beneficiaries of assistance from all three potential sources of assistance. For transfers from close family, 7.6% of Qataris received assistance compared to 2.3% of non-nationals, and for government help the respective percentages were 6.5% and 0.4%. The percentage of households receiving assistance was higher in poorer wealth quintiles than in the richer quintile for all types of transfer. Doha was the region that fewest household receive any type of assistance.

By comparing Tables 8.4 and 8.5 it can be calculated that the average amount of assistance received from each of the different sources over the 12 months prior to the survey was far less than the average amount of assistance transferred out of the households. The average amount of financial assistance received by households in the survey from family members was about QR8500 (US$2337), while assistance from the community was on average less than QR5900 (US$1622). However, assistance from the government was on average about QR24 500 (US$6375) in the year before the survey. Qatari nationals received a far higher average amount than non-Qatari nationals, especially with regard to governmental assistance: Qataris on average received QR26 413 (US$7261) and non-Qataris received only QR6411 (US$1762). Those living in the other regions of Qatar also received a far higher average amount of government assistance. This is due to three households who reported that they obtain much assistance in this manner, receiving QR320 000 (US$87968), QR600 000 (US$164960) and QR1 410 000 (US$387609). These three households are all classified as Qatari nationals and living outside of Doha and Al Rayyan.

Table 8.5 Financial and other transfers into the household

	Transfers from close family					Transfers from community					Financial/in-kind assistance from government				
	% transfers	% missing	*N*	Average amount	N	% transfers	% missing	N	Average amount	N	% transfers	% missing	N	Average amount	*N*
**Nationality status**															
Qatari	7.6	0.6	*1615*	9748	*123*	2.4	0.4	*1615*	6226	*38*	6.5	0.7	*1615*	26413	*105*
Non-Qatari	2.3	0.2	*3164*	6399	*73*	1.5	0.1	*3164*	5538	*48*	0.4	0.3	*3164*	6411	*11*
**Region**															
Doha	3.7	0.4	*2600*	9451	*97*	1.0	0.3	*2600*	5872	*27*	1.8	0.3	*2600*	10187	*47*
Al Rayyan	3.8	0.3	*1471*	6032	*56*	2.9	0.2	*1471*	6760	*43*	2.7	0.6	*1471*	8192	*39*
Other regions	6.0	0.4	*707*	9613	*42*	2.3	0.2	*707*	3392	*16*	4.3	0.4	*707*	67148	*31*
**Wealth quintile**															
Poorest	4.8	0.5	*1214*	5587	*58*	2.9	0.4	*1214*	6542	*35*	2.8	0.9	*1214*	8644	*34*
< average	4.3	0.6	*1005*	6532	*43*	2.6	0.3	*1005*	6122	*27*	2.9	0.5	*1005*	35949	*29*
Average	4.1	0.2	*1014*	9307	*41*	1.1	0.2	*1014*	3336	*11*	2.4	0.2	*1014*	11789	*24*
> average	3.8	0.3	*875*	9050	*33*	0.9	0.0	*875*	7675	*8*	2.1	0.1	*875*	7835	*18*
Richest	3.0	0.1	*670*	18538	*20*	0.7	0.0	*670*	1835	*5*	1.7	0.2	*670*	96303	*11*
**Total**	**4.1**	**0.4**	***4779***	**8501**	***195***	**1.8**	**0.2**	***4779***	**5845**	***86***	**2.4**	**0.4**	***4779***	**24461**	***117***

8.3.3 Transfers in and out of households

The analysis of transfers in and out of households conducted in previous sections does not inform about households that receive and give no support to or from other households, or regarding households that both give and receive. Table 8.6 presents information about this, listing the percentages of households that have no transfers flowing in or out, those that have only transfers in, those that have only transfers out, and those that have both transfers in and out of the household.

Just over half of the households (50.9%) provided financial aid or in-kind to support to other households, but did not receive any help themselves, while 2.8% received support without any transfer flowing out of the household. Almost 5% of households said that they both received and gave assistance, while 41.9% did not have any transfers in or out. Households classified as Qatari were more likely to have both types of transfer occurring in the household, with 9.3% saying that this occurred, compared to only 2% of non-Qatari households. Non-Qatari households were more likely not to have any transfers associated with them, or to be the providers of assistance. The households in the richest quintiles were more likely to be giving assistance without receiving any, while those in the poorest quintiles were more likely to be beneficiaries of assistance than richer households.

8.3.4 Provision of personal or health care to others

The giving of personal or health care to household members or to people outside of the household is an important part of the health system. Informal arrangements such as this reduces the burden on the system and ensures that people who are ill can live at home rather than having to be in a hospital or care home. Table 8.7 displays the percentage of respondents to the household questionnaire that give help to people in their own home or to other households, and also analysis the type of care that is given.

Giving care to someone within the household was a task performed by 3.7% of respondents, while 7% gave care to someone who lived outside the household. A much greater percentage of Qatari nationals executed these tasks than non-Qatari nationals. Less assistance was given to household members in Doha than in the other regions, although giving help to outside people was roughly similar across all of Qatar. Wealth was also related to care-giving, with those in the richer quintiles giving more care to those within and outside the household than those in the poorer quintiles.

The type of care that was given ranged from personal care to medical care, conducting household activities, watching over an ill person and helping movement outside the home. About 50% of carers were involved in each of the listed tasks, except for the task of watching over someone due to their behaviour being upsetting or dangerous. Only 18.9% of carers stated that they did this. Qataris were more likely than non-Qataris to give care in the form of personal care, such as helping toilet activities and getting someone dressed, and also were more likely to watch over someone. Conversely, non-Qataris were more likely to help with household activities such as meal preparation.

Table 8.6 Transfers in to households, out of households and households that have both types of transfer

	Provided help to a relative or friend due to illness				
	No transfers	Transfers out only	Transfers in only	Both transfers in and out	N
**Nationality status**					
Qatari	38.7	47.1	4.8	9.3	*1615*
Non-Qatari	43.5	52.8	1.8	2.0	*3164*
**Region**					
Doha	40.8	53.1	2.3	3.8	*2600*
Al Rayyan	47.5	45.0	3.3	4.2	*1471*
Other regions	34.0	55.1	3.7	7.3	*707*
**Wealth quintile**					
Poorest	47.5	43.7	4.9	3.9	*1214*
< average	45.3	46.1	3.4	5.2	*1005*
Average	39.5	54.0	1.8	4.7	*1014*
> average	39.1	54.5	1.2	5.1	*875*
Richest	33.5	61.7	1.6	3.2	*670*
**Total**	**41.9**	**50.9**	**2.8**	**4.5**	***4779***

Table 8.7 Provision of personal or health care to others

	Provided help to a relative or friend due to illness				Type of care provided					
	% yes within household	% yes outside household	% no help	*N*	% giving personal care	% giving medical care	% helping household activities	% watching person	% helping get around	*N*
**Nationality status**										
Qatari	6.9	10.8	82.3	*1615*	49.9	49.2	48.2	23.7	51.9	*286*
Non-Qatari	2.0	5.1	92.9	*3164*	41.6	47.9	55.7	12.7	53.9	*225*
**Region**										
Doha	2.6	7.3	90.1	*2600*	42.4	46.7	44.2	16.5	49.3	*259*
Al Rayyan	5.0	6.6	88.4	*1471*	49.7	50.9	60.3	21.2	59.5	*171*
Other regions	4.9	6.7	88.4	*707*	51.0	49.9	56.3	21.4	49.7	*82*
**Wealth quintile**										
Poorest	3.1	4.3	92.6	*1214*	48.6	48.6	44.0	20.0	48.9	*90*
< average	3.5	6.6	89.9	*1005*	48.6	47.4	59.6	24.7	58.6	*101*
Average	3.3	6.4	90.3	*1014*	46.0	53.5	52.8	21.2	47.9	*98*
> average	4.1	9.1	86.8	*875*	39.8	43.2	48.2	14.9	58.8	*115*
Richest	5.0	10.9	84.0	*670*	49.1	51.0	52.5	14.4	48.6	*107*
**Total**	**3.7**	**7.0**	**89.3**	***4779***	**46.2**	**48.6**	**51.5**	**18.9**	**52.8**	***512***

9. Social capital and subjective well-being

9.1 Introduction

The understanding of social capital is an important aspect of comprehending connections within and between social networks. They are also increasingly thought to positively influence public health (Harpham, Grant & Thomas, 2002). Social capital is a concept that encompasses a wide range of facets, including sociability, networks, trust and participation in local organizations. Public health is not only shaped by the individual, but also influenced by the interaction of individuals with their social environment.

A further aspect of health is how individuals perceive their own health and general well-being. A person may be objectively healthy, but if the perception of their own health and quality of life is low then the benefits of good health are not experienced by that person. The relationship between health and happiness is complex, although it has been argued that good health is a necessary predeterminant for well-being (Kircaldy, Furnham & Veenhoven, 2005). Hence, the health system has an important role to play in the wellbeing and quality of life.

The Qatar World Health Survey asked a series of questions about both social capital and subjective well-being and quality of life. These questions were asked to all individual respondents in the survey.

9.2 Social capital

9.2.1 Opinions on life

Three questions were asked to respondents about “things in their life” over the 30 days prior to the survey. These were related to control, coping and satisfaction with life. Table 9.1 presents the results for the questions relating to feelings of being unable to control important things in their life and regarding the frequency of not being able to cope with all the things that they had to do. Table 9.2 shows the results for satisfaction with life in general.

The results for opinions on control and coping were similar, with the same trends and relationships shown between subgroups. For all respondents, fewer than 80% stated that they had never or almost never felt unable to control aspects of their life or cope with things. Nearly 20% stated that they had sometimes felt unable to control or cope, while about 2% stated that they felt this way fairly often or very often. Males were more likely to state that they never felt this way, with women more likely to say that they sometimes or almost never had these feelings. Other differences were observed between Qataris and non-Qataris. Qataris were more likely to state that they rarely felt unable to control or cope with aspects of their life, compared to non-Qataris who were more likely to answer that they were never unable to cope or control things. Ability to control important things and ability to cope decreased as self-rated health deteriorated.

For the responses to the question item on satisfaction with life (Table 9.2), the majority of respondents said that they were satisfied with life. Almost 60% of people placed themselves in this category, compared to 36% who said that they were very satisfied. Only small percentages of people said that they were neither satisfied or dissatisfied or worse. There was little difference in responses by sex or wealth, but there were differences between the regions, nationality status and age.

Table 9.1 Percentage distribution of opinions on aspects of life in the past 30 days

	How often felt unable to control important things					How often felt unable to cope					
	Never	Almost never	Sometimes	Fairly often	Very often	Never	Almost never	Sometimes	Fairly often	Very often	*N*
**Sex**											
Male	63.4	19.4	15.6	1.0	0.6	62.7	20.2	15.6	1.2	0.3	*2411*
Female	49.6	25.2	22.3	1.9	1.0	49.5	25.9	22.0	1.6	1.0	*2355*
**Nationality status**											
Qatari	52.9	26.2	17.9	1.9	1.2	51.6	26.9	18.7	1.9	0.9	*1606*
Non-Qatari	58.5	20.2	19.4	1.2	0.7	58.6	21.0	18.8	1.2	0.5	*3160*
**Region**											
Doha	57.1	21.5	18.7	1.7	0.9	57.4	22.4	18.4	1.4	0.5	*2594*
Al Rayyan	56.8	22.8	18.3	1.2	0.9	55.5	23.5	18.5	1.6	0.9	*1466*
Other regions	54.2	23.9	20.8	0.6	0.6	53.2	24.2	20.8	1.2	0.6	*705*
**Age group**											
18 to 29	52.9	22.1	22.8	1.0	1.2	52.6	23.0	21.9	1.7	0.8	*1097*
30 to 44	56.1	23.4	18.2	1.5	0.8	56.3	23.5	18.4	1.3	0.5	*2384*
45 to 59	60.9	20.1	17.2	1.7	0.2	59.7	22.2	16.2	1.4	0.6	*1123*
60 to 69	62.2	21.1	14.5	1.5	0.7	55.2	21.8	20.8	1.0	1.2	*126*
70 or over	50.7	17.5	19.9	1.9	10.0	52.6	17.4	23.4	0.0	6.6	*35*
**Self-Rated Health**											
Very good	62.7	19.6	16.4	0.9	0.4	62.5	21.0	15.2	1.0	0.4	*2109*
Good	56.0	22.6	19.4	1.2	0.8	54.7	23.8	19.7	1.4	0.4	*2065*
Moderate	37.6	30.7	25.9	3.1	2.7	40.0	27.6	28.0	2.6	1.8	*506*
Bad	25.7	32.0	29.6	10.2	2.5	31.5	29.2	30.8	4.4	4.1	*73*
Very bad	63.9	12.0	14.6	5.3	4.2	54.5	15.1	20.9	5.3	4.2	*13*
**Wealth quintile**											
Poorest	58.4	21.2	18.8	1.4	0.2	58.6	22.4	17.6	0.9	0.5	*1224*
< average	54.2	21.5	21.4	1.6	1.3	53.3	23.1	21.1	1.2	1.1	*1017*
Average	56.0	22.6	19.1	1.3	1.1	55.4	24.9	18.0	1.4	0.3	*1019*
> average	55.6	25.2	16.5	1.5	1.2	54.5	23.7	19.2	2.2	0.5	*836*
Richest	59.0	21.1	18.1	1.2	0.5	59.7	20.0	18.0	1.5	0.8	*670*
**Total**	**56.6**	**22.2**	**18.9**	**1.4**	**0.8**	**56.2**	**23.0**	**18.8**	**1.4**	**0.6**	***4765***

Table 9.2 Percentage distribution of ratings of satisfaction with life

	Very satisfied	Satisfied	Neither satisfied or dissatisfied	Dissatisfied	Very dissatisfied	*N*
**Sex**						
Male	36.1	59.8	2.8	1.2	0.1	*2413*
Female	35.9	59.6	2.9	1.5	0.1	*2355*
**Nationality status**						
Qatari	38.8	56.2	3.3	1.6	0.0	*1607*
Non-Qatari	34.6	61.4	2.6	1.2	0.1	*3161*
**Region**						
Doha	35.6	60.2	3.0	1.2	0.1	*2594*
Al Rayyan	35.1	60.8	2.3	1.7	0.0	*1466*
Other regions	39.5	55.6	3.3	1.3	0.4	*707*
**Age group**						
18 to 29	36.5	57.1	3.9	2.3	0.1	*1097*
30 to 44	36.0	60.1	2.5	1.1	0.2	*2384*
45 to 59	35.1	61.6	2.5	0.7	0.0	*1125*
60 to 69	38.9	57.2	2.5	1.4	0.0	*126*
70 or over	40.4	54.9	1.8	3.0	0.0	*35*
**Self-assessed health**						
Very good	46.4	51.1	1.9	0.7	0.1	*2111*
Good	29.7	65.9	3.0	1.4	0.1	*2065*
Moderate	20.6	70.6	5.6	3.2	0.0	*506*
Bad	26.5	60.9	6.8	4.3	1.5	*73*
Very bad	22.9	45.3	10.4	14.6	6.9	*13*
**Wealth quintile**						
Poorest	35.8	60.1	3.1	1.0	0.0	*1224*
< average	37.3	57.6	3.3	1.6	0.2	*1018*
Average	37.8	58.0	2.7	1.5	0.0	*1019*
> average	32.1	63.9	2.5	1.4	0.1	*837*
Richest	36.7	59.3	2.5	1.2	0.3	*670*
**Total**	**36.0**	**59.7**	**2.8**	**1.3**	**0.1**	***4768***

A higher percentage of people in the other regions of Qatar reported that they were very satisfied compared to the two regions of Doha and Al Rayyan. These two regions had a higher percentage of people who were only satisfied with life. Qatari nationals were more likely to state that they were very satisfied, with 38.8%, than non-Qatari nationals, with 34.6%. However, nationals were also more likely to state that they were neither satisfied or dissatisfied or worse. The differences between age groups indicated that as age increased the percentage of people who stated that they were very satisfied increased. It is also noticeable that the percentage of people saying that they were neither satisfied or dissatisfied fell as age rose, from 3.9% of 18–29 year olds to 1.8% of those over 70. Finally, satisfaction decreased with self-rated health. Out of those who

stated that their health was very good, 97.5% were very satisfied or satisfied with life. For those who said that their health was bad, the same figure was 68.2%.

9.2.2 Involvement in the community

The interaction between the respondents and their community was measured using a series of question regarding their activities in the 12 months before the survey. Respondents were asked whether they had:

- attended a public meeting where local or school affairs were discussed
- met with a community leader
- attended a club or organizational meeting
- had friends over to their home
- been in the house of someone from a different neighbourhood, or had people over to their home
- socialized with co-workers outside of work
- worked with other people in the neighbourhood to fix or improve something.

The percentage of people who had conducted each of these activities is shown in Table 9.3. The activity that had the highest percentage of respondents saying that they had done in the last 12 months was to have friends over to their house, with 77.4% of respondents saying that this had happened. Over three-quarters of respondents also stated that they had been to the house of someone from a different neighbourhood or had them over to their home. Socializing with co-workers was an activity conducted by 43.4% of respondents, while almost a third (32.1%) attended a public meeting. Meeting with a community leader, attending a club or a meeting and fixing or improving something in the neighbourhood was performed by a minority of people.

A higher percentage of females than males had attended a public meeting, although all the other activities were seen to be either predominantly conducted by males or there was minimal difference in participation between the sexes. Meeting with a community leader, attending a club or meeting, socializing with co-workers and working to fix or improve something in the neighbourhood had about double the percentage of males than females stating that this was an activity that they had done in the previous 12 months. Qataris were more active than non-Qataris in the activities listed, especially with regard to meeting a community leader and attending a public meeting.

The region of Qatar where the respondents resided had little effect on the activities performed, although a higher percentage of people attending a public meeting and working to improve the neighbourhood was observed in regions outside of Doha or Al Rayyan. In general, participation increased as age rose until the 45–59 age group, where the highest percentages for participation were seen. The percentages then fell in the oldest two age groups. Wealth does not seem to have an effect on participation, although there is some evidence that the poorest group of respondents have the lowest rates of participation.

Table 9.3 Percentage of respondents involved with different aspects of the community

	In the last 12 months did the respondent*:							
	**Attend public meeting**	**Meet with community leader**	**Attend club/ meeting**	**Have friends to house**	**Visit people in different neighbourhood**	**Socialize with co-workers**	**Work to fix/ improve something in neighbourhood**	***N***
**Sex**								
Male	27.8	19.9	21.2	76.6	74.7	57.6	16.1	*2413*
Female	36.6	8.2	12.4	78.2	76.3	28.9	7.8	*2355*
**Nationality status**								
Qatari	37.8	19.9	15.9	77.8	77.7	46.1	13.5	*1607*
Non-Qatari	29.2	11.2	17.3	77.2	74.4	42.1	11.3	*3161*
**Region**								
Doha	30.9	13.7	17.9	77.2	75.9	44.2	11.0	*2595*
Al Rayyan	33.1	14.8	14.8	78.7	74.2	39.9	11.3	*1466*
Other regions	34.6	14.2	17.0	75.6	76.6	48.0	17.1	*707*
**Age group**								
18 to 29	20.0	11.9	13.5	76.2	72.8	38.0	9.7	*1097*
30 to 44	39.3	13.9	16.5	79.1	76.9	45.5	12.4	*2385*
45 to 59	32.3	17.0	21.1	76.8	77.1	46.8	13.5	*1125*
60 to 69	6.9	12.2	13.4	66.4	62.0	30.8	13.1	*126*
70 or over	8.8	14.9	16.8	54.2	58.3	17.7	10.0	*35*
**Wealth quintile**								
Poorest	32.2	14.1	16.2	75.7	73.9	40.9	10.3	*1225*
< average	34.2	13.5	18.2	77.8	74.1	42.9	12.9	*1018*
Average	31.9	16.1	19.3	79.2	77.6	46.0	12.8	*1019*
> average	30.4	12.4	15.7	76.2	75.9	45.0	12.7	*837*
Richest	31.4	14.3	13.6	78.6	76.6	43.0	11.9	*670*
**Total**	**32.1**	**14.1**	**16.8**	**77.4**	**75.5**	**43.4**	**12.0**	***4768***

* Respondents could state that they were involved in more than one activity listed. Hence the percentages do not total 100%

9.2.3 Social trust

There are many studies that have shown that there is a link between trust in society and health, at both individual and community levels (Kawachi et al., 1997; Veenstra, 2002). Respondents were asked whether they could generally trust those in three different groups: their neighbourhood, co-workers and strangers. Responses were recoded into two categories—those who said that some or a lot of people could be trusted, and those who stated that they only had a little trust or no trust at all. Some people also stated that they did not know if they could trust. The results are shown in Table 9.4.

Just under 50% of respondents stated that they had trust in people in their neighbourhood. Trust was higher in males then females: 52.1% of males stated that they had trust in neighbours, while only 42.8% of females said the same thing. A larger proportion of non-nationals stated that they had trust than nationals, and trust was higher outside of Doha and Al Rayyan. In the 45–59 age group, 54.2% of people said that they had trust in people in the neighbourhood, while only 38.2% of 18–29 year olds said the same thing.

Trust at work was much higher for males than females, although there were a far higher percentage of women who stated that they did not know about the trust levels for those that they worked with. Again, the 45–59 age group had the highest level of trust, with 59% stating that they could trust their co-workers, with the 18–29 year olds displaying the least trust on this scale. Co-worker trust in Al Rayyan was much less that that seen in Doha and other regions, with 47.6% reporting trust compared to 56% respectively.

The level of trust in strangers was much lower than the trust seen in the neighbourhood and at work. Once more, males and non-Qataris had more trust on this aspect than females and Qatari nationals. There are differences between the regions, with respondents in Doha reporting more trust than in the other regions of Qatar. In Doha almost 28% of people said that they had some or a lot of trust in strangers, compared to 22.8% in Al Rayyan and 22.6% in other regions. Trust in strangers also grew as age increased, with only 20% of those in the youngest age group reporting trust, compared to 33.5% in the 60–69 age group.

9.2.4 Crime and fear of crime

A final aspect of social trust is related to crime. There are two different dimensions to crime: the fear of crime occurring and the actual experience of crime. Questions were asked about how safe the respondents felt when they were alone at home and when they were walking down their street alone after dark. A final question investigated whether the individual or anyone in the household had been a victim of violent crime. Table 9.5 presents the results for crime in Qatar. The percentage of people who were classified as feeling safe was the percentage of respondents who stated that they felt completely safe or very safe.

Nearly 90% of the people questioned in the survey felt safe alone at home, while over three-quarters felt safe walking alone down their street after dark. A higher percentage of males felt safe both at home and on the street than females. Only 62.9% of females felt safe walking down their street alone after dark, compared with 89% of males. Regarding safety on the street, this was higher outside in the regions outside of Doha and Al Rayyan. In these other regions 81.4% felt safe, as opposed to 77% in Al Rayyan and 74.2% in Doha. Feelings of safety also increase with age.

Table 9.4 Percentage distribution of trust in different groups of people

		Does respondent trust people:						
		In neighbourhood		At work		Strangers		
		**Yes***	**Don't know**	**Yes***	**Don't know**	**Yes***	**Don't know**	**N**
**Sex**								
	Male	52.1	5.9	61.7	3.3	28.9	3.0	*2413*
	Female	42.8	6.0	45.0	14.8	22.2	3.2	*2355*
**Nationality status**								
	Qatari	43.4	3.9	46.2	8.7	18.3	2.3	*1607*
	Non-Qatari	49.6	7.0	57.1	9.2	29.3	3.5	*3161*
**Region**								
	Doha	48.4	6.4	56.0	7.8	27.9	3.0	*2595*
	Al Rayyan	44.0	6.2	47.6	11.4	22.8	3.1	*1466*
	Other regions	51.5	3.8	56.2	8.4	22.6	3.5	*707*
**Age group**								
	18 to 29	38.2	5.6	43.8	10.5	20.0	3.0	*1097*
	30 to 44	48.8	6.7	55.6	9.1	25.2	3.3	*2385*
	45 to 59	54.2	4.8	59.0	6.5	31.1	2.8	*1125*
	60 to 69	45.3	4.3	48.4	14.7	33.5	4.6	*126*
	70 or over	43.8	7.7	45.2	12.9	22.4	0.0	*35*
**Wealth quintile**								
	Poorest	46.9	5.9	52.5	10.4	25.4	3.5	*1225*
	< average	44.8	5.9	54.0	8.5	25.4	2.8	*1018*
	Average	49.8	5.5	54.1	8.0	25.7	3.6	*1019*
	> average	48.4	7.5	51.7	11.0	26.3	3.0	*837*
	Richest	48.1	4.8	55.5	6.3	25.0	2.1	*670*
**Total**		**47.5**	**5.9**	**53.4**	**9.0**	**25.6**	**3.1**	***4768***

* Percentage of respondents reporting that they had 'some' or 'a lot' of trust

Table 9.5 Percentage distribution of fear of crime and percentage respondents victim of crime

	How safe from crime and violence does respondent feel:				Victim of violent crime in last 12 months		
	**At home***	***N***	**On street after dark***	***N***	**% Yes**	**% No**	***N***
**Sex**							
Male	92.8	*2408*	89.0	*2397*	3.5	96.5	*2392*
Female	86.5	*2352*	62.9	*2338*	3.1	96.9	*2342*
**Nationality status**							
Qatari	89.5	*1603*	74.7	*1597*	3.9	96.1	*1596*
Non-Qatari	89.8	*3157*	76.8	*3139*	3.0	97.0	*3138*
**Region**							
Doha	88.6	*2591*	74.2	*2576*	3.0	97.0	*2568*
Al Rayyan	91.5	*1466*	77.0	*1458*	4.2	95.8	*1463*
Other regions	89.9	*703*	81.4	*702*	2.7	97.3	*703*
**Age group**							
18 to 29	88.0	*1095*	71.9	*1086*	2.6	97.4	*1088*
30 to 44	88.7	*2380*	74.7	*2374*	3.4	96.6	*2366*
45 to 59	92.5	*1124*	82.2	*1115*	3.9	96.1	*1119*
60 to 69	94.9	*126*	79.8	*126*	2.1	97.9	*125*
70 or over	100.0	*35*	95.8	*35*	2.8	97.2	*35*
**Wealth quintile**							
Poorest	88.7	*1223*	75.7	*1216*	3.8	96.2	*1216*
< average	89.7	*1017*	76.8	*1009*	3.3	96.7	*1013*
Average	90.6	*1016*	77.1	*1016*	2.6	97.4	*1010*
> average	89.0	*834*	74.0	*830*	3.1	96.9	*832*
Richest	91.1	*670*	77.1	*665*	3.7	96.3	*664*
**Total**	**89.7**	***4760***	**76.1**	***4736***	**3.3**	**96.7**	***4734***

* Percentage of respondents reporting that they felt ‘completely safe’ or ‘very safe’

The question on actual experiences of crime was asked in the individual questionnaire, although it referred to the respondent or anyone else in the household. There were 3.3% of respondents who stated that they, or someone else in their household, had been a victim of violent crime in the three months prior to the survey. The percentage of households who had experienced this was higher for Qatari households and those located in Al Rayyan. Furthermore, respondents aged 30–59 reported more violent crime experienced by their households compared with respondents who were older or younger.

9.3 Subjective well-being and quality of life

9.3.1 Subjective well-being

Subjective well-being was measured in a series of questions relating to energy, money and satisfaction with various aspects of their life. Satisfaction was again coded into two categories of satisfied and dissatisfied, with satisfaction being assumed if the respondent had stated that they were very satisfied or satisfied with the different spheres of well-being that were asked. The results for these questions are shown in Table 9.6a.

Over 95% of the respondents stated that they had enough energy for everyday life, while only 81.3% stated that they had enough money to meet every day needs. Age had the strongest relationship to energy, with younger age groups having a higher percentage of respondents with enough energy compared to the older ages. A lower proportion of females than males stated they had enough energy (93.7% and 97.2% respectively), while more non-Qataris felt that they did have enough energy compared with Qatari nationals (96.5% and 93.5% respectively). Feeling that there was enough money did not vary greatly between gender or nationality status. The percentage of respondents in Al Rayyan who felt they did not have enough money was lower than in Doha, with figures of 77.3% and 81.7% respectively. There was no large variation in the responses to having enough money to meet needs by wealth quintile, indicating that this variable may not measure wealth that accurately.

Table 9.6a Percentage distribution of satisfaction with different aspects of life

		Enough energy for everyday life?	Enough money to meet needs?	Satisfaction with:								
		% Yes	% Don't know	% Yes	% Don't know	Health	Themselves	Ability to perform daily living activities	Personal relationships	Conditions of living place	Life as a whole	*N*
**Sex**												
	Male	97.2	0.4	81.5	1.3	97.1	97.4	96.7	97.0	88.3	96.9	*2405*
	Female	93.7	0.5	81.2	1.4	93.9	96.4	94.3	96.3	88.2	96.2	*2355*
**Nationality status**												
	Qatari	93.5	0.4	82.3	1.3	94.5	97.0	92.8	96.2	89.2	96.0	*1604*
	Non-Qatari	96.5	0.5	80.8	1.4	96.0	96.8	96.9	96.9	87.8	96.8	*3156*
**Region**												
	Doha	96.3	0.4	83.5	1.3	95.9	97.2	95.7	96.3	88.0	96.7	*2586*
	Al Rayyan	94.5	0.4	77.3	1.2	95.1	96.8	95.6	97.1	88.3	96.5	*1468*
	Other regions	94.6	0.7	81.7	1.6	94.8	95.8	94.7	96.8	88.9	96.2	*706*
**Age group**												
	18 to 29	97.0	0.3	80.7	0.9	95.4	95.7	94.5	96.1	86.3	94.9	*1091*
	30 to 44	95.6	0.5	81.2	1.4	95.4	96.7	95.5	96.2	87.0	97.0	*2385*
	45 to 59	95.1	0.2	81.4	1.8	96.5	98.4	97.4	97.8	92.0	97.1	*1125*
	60 to 69	89.6	1.7	89.5	0.9	91.3	100.0	92.5	98.2	94.3	99.1	*125*
	70 or over	73.3	0.0	81.5	0.0	82.3	87.9	77.4	98.5	93.9	94.2	*35*
**Wealth quintile**												
	Poorest	95.6	0.4	81.2	1.0	94.1	96.8	94.9	95.8	86.8	96.4	*1222*
	< average	94.8	0.9	80.5	1.4	95.5	95.9	94.6	96.9	87.6	96.0	*1017*
	Average	97.0	0.3	82.0	1.9	96.1	97.2	96.1	97.2	89.2	97.0	*1016*
	> average	94.3	0.3	81.0	1.2	96.5	97.3	96.4	96.6	89.2	96.6	*837*
	Richest	95.5	0.1	82.3	1.2	95.9	97.5	96.1	96.9	89.3	97.0	*668*
**Total**		**95.5**	**0.4**	**81.3**	**1.3**	**95.5**	**96.9**	**95.5**	**96.6**	**88.2**	**96.6**	***4760***

Satisfaction with various aspects of life was high, with over 95% of respondents saying that they were satisfied on each of the issues, except for the question regarding the conditions of their living place. For this issue, only 88.2% of the respondents stated that they were satisfied. 96.6% of individuals said that they were satisfied with their life as a whole. Variation by subgroup indicated that males were more satisfied than females, especially in the domains of health and their ability to perform daily living activities. A further trend to highlight is satisfaction with living conditions, which increased as age rose. Only 86.3% of the youngest age group were satisfied with this aspect of their life, compared with 94.3% of those in the 60–69 age group and 93.9% of those aged over 70.

9.3.2 WHO quality of life rating

The World Health Organization has developed an instrument to measure overall quality of life. This instrument is aimed to measure quality of life in such a way that it is cross-culturally valid and sensitive. The initial quality of life instrument has 100 questions, but this has been shortened to an eight-item scale (WHOQol-8). The questions analysed in the previous section are those included in the quality of life instrument.

To obtain the final WHOQol-8 score, the responses to the questions were combined and scaled to range from 0 to 100. A score of 0 would indicate an extremely poor quality of life, while a score of 100 is a very good quality of life. Table 9.6b shows the results for the quality of life, broken down by subgroup of population.

The results indicate that quality of life in Qatar was very high, with an average score of 91. This reflects the high percentages of people who were satisfied with the various aspects of the quality of life noted in Table 9.6a. There are some interesting differential between population subgroups with respect to the WHOQol-8 score. Minimal differences are seen by sex or nationality status. There are slight differences by region, with Al Rayyan having the lowest mean quality of life, with 90.1, and the regions outside of Al Rayyan and Doha having the highest, with 92.9. Quality of life differed by age, with the mean score increasing with age, except for the final age group of the over 70s, where the mean rating fell dramatically.

Possibly the most interesting relationship was between quality of life and wealth. The poorest wealth quintile had an average quality of life score of 88.5. As wealth quintile increased, so did the average score, with the wealthiest group having a mean score of 94.1. This relationship is as would be expected, with the wealthier group of people being able to enjoy a better quality of life due to the resources available to them.

9.3.3 Overall quality of life

The final questions asked to the respondents in the Qatar World Health Survey related to the individuals views on their overall quality of life. There were two questions relating to this, each with a five-point scale ranging from very good to very bad. The first question asked the individual to rate their overall quality of life, while the second simply asked the person to rate “how they are these days”. The results are shown in Table 9.7.

Table 9.6b Mean WHO quality of life (WHOQol-8) score by population subgroup

		WHOQol-8 Score	N
**Sex**			
	Male	91.2	*2405*
	Female	90.7	*2355*
**Nationality status**			
	Qatari	91.2	*1604*
	Non-Qatari	90.8	*3156*
**Region**			
	Doha	90.9	*2586*
	Al Rayyan	90.1	*1468*
	Other regions	92.9	*706*
**Age group**			
	18 to 29	89.7	*1091*
	30 to 44	91.1	*2385*
	45 to 59	92.0	*1125*
	60 to 69	92.2	*125*
	70 or over	84.1	*35*
**Wealth quintile**			
	Poorest	88.5	*1359*
	< average	91.0	*1222*
	Average	91.7	*919*
	> average	92.4	*723*
	Richest	94.1	*538*
**Total**		**91.0**	***4760***

Regarding overall quality of life, 36.7% of respondents stated that they had a very good quality of life, while 51.2% said that it was good. The percentage reporting a moderate quality was only 11.4%, with only very small percentages of people reporting a bad or very bad quality of life. A higher percentage of females reported a very good quality compared with males, and more males fell into the moderate category. Qatari nationals were also more likely to be very happy with their quality of life than individuals who were not Qatari nationals. The youngest age group were the most positive about their life, with 40% saying that their quality of life was very good, while the oldest were the least positive, with only 31.7% saying that their life was very good, and 14.7% stating only a moderate quality.

For the question relating to how the respondent was "these days", fewer people stated that they were very good. Only 28.3% of people placed themselves in this category, while 62.3% said that they were good. The percentage of respondents in the moderate category was 8%, while 1.3% was in the bad or very bad categories. More Qataris than non-Qataris said that they were in the best category, very good, but also a higher percentage placed themselves into the bad groups. Once again the percentages in each group varied by age group, with a higher percentage of those aged over 60 saying that they were very good than those in the younger age groups.

Table 9.7 Percentage distribution of overall ratings of quality of life

		Overall quality of life						How is the respondent these days?					
		Very good	Good	Moderate	Bad	Very bad	Don't know	Very good	Good	Moderate	Bad	Very bad	Don't know
**Sex**													
	Male	34.9	52.0	12.3	0.5	0.3	0.0	28.7	62.1	8.2	0.7	0.4	0.0
	Female	38.4	50.3	10.5	0.6	0.1	0.1	27.9	62.5	7.9	1.3	0.2	0.1
**Nationality status**													
	Qatari	43.7	47.6	8.2	0.4	0.1	0.0	30.9	61.3	6.0	1.6	0.2	0.0
	Non-Qatari	33.1	53.0	13.1	0.6	0.2	0.0	27.0	62.8	9.1	0.7	0.4	0.1
**Region**													
	Doha	36.5	52.1	10.5	0.5	0.2	0.1	28.3	61.8	8.6	0.9	0.3	0.1
	Al Rayyan	36.7	50.2	12.5	0.5	0.1	0.0	27.2	64.5	6.9	1.1	0.2	0.0
	Other regions	37.0	49.8	12.3	0.7	0.2	0.0	30.7	59.5	8.2	1.2	0.5	0.0
**Age group**													
	18 to 29	40.0	47.5	11.2	0.7	0.5	0.1	29.9	59.1	9.0	1.4	0.6	0.1
	30 to 44	35.6	53.0	10.6	0.7	0.1	0.0	26.8	64.4	7.3	1.0	0.3	0.1
	45 to 59	35.6	50.4	13.6	0.3	0.2	0.0	29.2	61.0	9.0	0.8	0.1	0.0
	60 to 69	38.2	53.4	8.4	0.0	0.0	0.0	32.8	64.3	2.9	0.0	0.0	0.0
	70 or over	31.7	53.6	14.7	0.0	0.0	0.0	33.5	52.6	12.1	1.7	0.0	0.0
**Wealth quintile**													
	Poorest	37.2	50.6	11.7	0.2	0.3	0.0	28.0	62.3	8.5	0.9	0.3	0.0
	< average	36.8	49.5	12.8	0.5	0.3	0.1	30.3	60.6	7.9	0.7	0.4	0.1
	Average	38.5	50.4	10.3	0.8	0.0	0.0	31.8	60.2	7.0	0.8	0.2	0.0
	> average	32.9	56.1	10.3	0.5	0.1	0.1	23.4	66.1	8.8	1.2	0.2	0.3
	Richest	37.3	49.7	11.8	1.0	0.2	0.0	26.5	63.4	8.0	1.7	0.4	0.0
**Total**		**36.7**	**51.2**	**11.4**	**0.6**	**0.2**	**0.0**	**28.3**	**62.3**	**8.0**	**1.0**	**0.3**	**0.1**

10. Major findings and policy recommendations

10.1 Introduction

The Qatari Health Survey has reinforced the growing body of evidence to show that the Qatari population has achieved a high status of health, and that standards are ever increasing, even by the standards of the industrialized nations. Life expectancy has risen sharply as healthcare provision has improved, reaching 77 years in 2005 (WHO Statistical Information System WHOSIS[*]), compared with 53 in 1960. A significant portion of this reduction has been achieved through the improved survival of children, as Qataris have seen a 50% drop in infant mortality from 17.0 per 1000 live births in 1981 to 8.2 per 1000 now, while under-5 mortality rates have also plummeted, from 140 in 1960, to 32 in 1980 and to the present rate of 12 in 2005 (WHOSIS). This is coupled with a much lower fertility rate—now fewer than three children per woman living throughout her childbearing years—much reduced compared with levels seen at independence[†].

These statistics are well known from previous sources, but the Qatari 2006 World Health Survey provides evidence a significant improvement in the availability of reliable health information as a foundational step from which the National Health Authority and Qatar Statistical Authority can move forward. Detailed health information of this kind is a crucial initial step for Qatar's strategic vision for the future: helping people achieve their full potential, thereby benefiting the individuals, their families, the community and the nation. Enhancing the wellness of the people of Qatar means understanding weaknesses in the health system and knowing which parts of the population have health problems. Tackling key health problems effectively will help to establish a vibrant, healthy and productive society for today and for the future.

The Qatari World Health Survey, a major undertaking in data collection and analysis, comprises the collective responses to a well designed questionnaire on all aspects of health from a sample of respondents that were scientifically selected to represent the nation. The resulting population sample was interviewed in a total of 4779 households, in which a key informant over the age of 18 within the household answered on behalf of all members of that household. Overall, the survey covered a representative population of 29 339 inhabitants. The results showed that 48.5% of the household population was male, and 39.5% were Qatari nationals. They also showed that just over a third of the sampled population were under 15 years of age, while only 2.9% were over 60 years old. There were higher percentages of Qatari nationals in these two age groups—children and older persons—compared with non-Qatari nationals, while in the middle ages—30–59 years—there was a higher percentage of non-Qataris. Non-Qatari women dominated the 25–44 age groups, to a lesser extent, so did non-Qatari men in the same age group. The age structure of this representative population indicates that fertility of Qataris has fallen in the past 10 to 15 years, although the fertility rate of non-Qataris is still relatively high.

The age and sex structure of the population is the most basic building block on which health policy must be built, but employment and educational status are also important given the links between work and health and the maintenance of a healthy and educated workforce. In the survey sample, nearly 45% had completed high school or above, while nearly 15% were without any formal schooling, with almost two-thirds of those

---

[*] http://www.who.int/whosis/en/

[†] WHO Regional Health Systems Observatory (2006), Qatar Health System Profile. EMRO, WHO Cairo.

individuals being able to read and write. Although some age structure effects may be distorting the patterns; in general non-Qatari nationals had higher educational achievement than Qataris, while more Qataris had low levels of formal education. In terms of employment the majority of respondents worked for the government, while 36.6% worked in the private sector. For females who were currently working, a higher percentage worked in the government sector than males, while a greater proportion of males had a private employer or were self-employed. Qatari nationals were mainly employed by the government, with only 7.6% working privately. Conversely, almost half of non-Qataris worked in the private sector, with 42.3% working for the government. These patterns have important implications for health and also for the health system as the receipt of benefits from work can form an important part of employment packages and can include pensions and medical services.

Based on a representative survey population with the above-mentioned characteristics, this chapter summarizes the key findings from the Qatari World Health Survey and discusses the key findings. These findings are divided into two sections: public health characteristics, which tell us about the health status of Qatari people, and health system characteristics which provide information on the functioning of health services and governance. These results represent a key component on which health policy formation can be developed in Qatar. They also serve as a baseline against which results from future surveys can be compared against to assess the success of health system changes and initiatives.

10.2 Public health in Qatar

This section provides a summary and discussion of results from the survey on the state of the population's health in Qatar, the health-related quality of life in Qatar, the risk factors that predispose Qataris to ill health and disease, and the illness patterns experienced among Qataris. These general results should always be interpreted in the light of

- their self-reported nature
- the influence of non-response
- the age, sex and nationality of the populations that make up the headline figures.

When considering the first of these, it is important to realize that no health workers were involved in the responses to individual questionnaires, so that all data are composed of the experiences of ordinary people in Qatar. This is entirely appropriate given that the survey aims to understand Qatari health from the very people who might suffer ill health. The results are therefore subject to possible biases resulting from differential experience of pain, discomfort and interaction with health services. An added dimension is that one respondent in a household is often asked to complete a questionnaire on behalf of others, and the result may bear the bias of the characteristics of the primary respondent (is this true? – has it been checked out?). Biomarkers such as cholesterol testing and blood-pressure measurement may be seen to be more objective, but still suffer from the influences from non-response. Those individuals that exercise their right to refuse either to participate, or to answer particular questions or undergo particular tests may share similar characteristics. However, the extent to which non-response affects results in this survey is minimal, especially as weights are applied to compensate for unit non-response (a household with no returned questionnaire; see chapter 2). Where respondents do not answer a particular question (item non-response), this is shown in the tables. The final consideration when interpreting findings is the

composition of the population. It is interesting to note that the female non-Qatari middle aged population (22–49 years) almost matched an equivalent pattern among male non-Qataris. Headline statistics highlighted by the Qatar 2006 survey become easier to interpret, especially when the separate figures are prepared based on disaggregated data by age, sex and nationality group.

10.2.1 State of the population's health

A good entry point for understanding the details of the state of Qatari health is to assess summaries of the population's responses to basic health questions on mobility, pain levels, sleeping problems, breathing, anxiety levels, vision and hearing, and concentration levels – all of which give an underlying view of levels of well-being. These can be supplemented by details on the personal relationships that Qataris build for themselves and the self care that they manage outside the formal health care system, both of which are important for general levels of health. As these aspects of health have never been measured before on a nationwide basis, the results represent both an interesting benchmark for policy-building as well as a baseline for future studies that might be undertaken after corrective policies have been put into place.

In terms of mobility the picture is encouraging, with nearly 89% of the population able to move around without difficulty, and nearly a further 7% able to move around with only mild difficulty. Abilities to perform basic self-care activities were also widespread with only very small minorities with difficulties. Problems were, however, more pronounced among women, and also among nationals—the latter probably because of the older age-profile of Qatari nationals as compared with non-Qataris. Health programmes that take reduced mobility and self-care capacity of these subgroups into consideration are clearly needed. The tendency for isolation among older people (particularly older women) was further underlined by the survey results on personal relationships which, although on the whole indicated a population well connected by personal relationships and community participation, also pointed at the problems that older people and women suffer in terms of their ability to deal with conflicts and tensions.

The results for pain indicate that two-thirds of people do not experience aches, pains or bodily discomfort and a further 20% experience discomfort only very rarely. Concentration and remembering things are also not problems for the great majority of the population. These are encouraging results, but again the picture is worse for Qataris, and also worse for women—in terms of lacking aches and pain the sex differential is over 20 percentage points. The same pattern is seen with breathing problems, with more than 10 percentage points between men with no breathing difficulties as compared with women, and even more of a gap between Qataris and non-Qataris. Again women and Qataris rate themselves worse than mean and non-Qataris. A general picture of sex and nationality disadvantage is emerging but differentials by wealth status do not emerge here, signifying an equitable health distribution with respect to household resources.

Equity in the availability and distribution of health services and access to healthcare is also reflected in sleep and energy results, as well as those for anxiety. Some respondents reported that they have difficulty with sleeping, and this proportion, although small, was highest among Qatari respondents (one-third said that they have difficulties sleeping). Difficulties with sleep increase with age, and residents of Doha report fewer problems than others. Anxiety and feeling low or depressed was reasonably common (just over one-third of respondents), but followed the now well

established pattern of higher prevalence among women and Qataris, this time with very sizeable differentials.

A less subjective set of results were produced for vision and hearing, with respondents asked whether they needed glasses, contact lenses or hearing aids. This set of results reversed the previous tendencies, with men and non-Qataris stating greater need, albeit by only a small margin. One in five people find it difficult to see and recognize someone across a road, one in 10 people find it hard to hear what is said in a conversation with one other person in a quiet room, and just over 4% of the population reported the use of hearing aids. These are relatively high proportions, especially for hearing aids, but there are no appreciable differentials between subgroups of the population such as sex, nationality or wealth groups. Problems do multiply with age, but this is to be expected. On the whole it is to be commended that a reasonably significant health problem is seemingly dealt with equitably in Qatar, but it should be noted that health information—particularly in written form, but also in spoken form as a component of consultation—should be delivered in a way that is sensitive to those with hearing and vision problems.

When reporting more subjective aspects of health it is important to acknowledge the possible biases inherent in self-reported health whereby subgroups of the population may have greater or lesser health expectations, thus rating their health status accordingly. Health vignettes administered as part of the survey attempted to identify the extent to which women and Qataris may report their health status differently from their male and non-Qatari counterparts as a results of different expectations. The results showed that these effects are not enough to distort the findings significantly.

10.2.2 Quality of life

A sum total of specific the specific health states reported above such as sleep, energy, depression, hearing, pain and mobility could be expressed as an overall satisfaction with life, or an assessment of quality of life. The Qatari Health Survey set out to evaluate exactly that summary of health by including a set of self-assessed general levels of health and satisfaction. This showed that the population of Qatar are indeed generally happy with the quality of their lives and their health with less than 2% rating their overall health as bad. Men are more likely to rate themselves as healthy than women, and non-Qataris more likely to rate themselves healthy compared with Qataris. Fear of crime is no real problem, but there is a significant group of households that have been affected by violent crime: 3.3%. The high levels of general satisfaction are also reflected in the responses of the population of Qatar to the internationally verified WHODAS (WHO Psychiatric Disability Schedule) questionnaire. This shows that the average level of disability is very low, and psychiatric functioning is high, despite familiar differences between men and women (with women reporting worse health states) and between Qataris and non-Qataris (with Qataris reporting worse health than non-Qataris). The results reinforce the main message from this part of the questionnaire: over three-quarters of respondents state that health difficulties do not interfere with their life at all and most of the remainder cite only mild interference.

10.2.3 Coping with risk factors

Some behaviour is known to predispose to poor health and is known to increase the likelihood of illness. Smoking, poor dietary habits and inadequate physical exercise are prime examples of this behaviour—risk factors for poor health. This behaviour was investigated as part of the Qatar Health Survey. Also included was an assessment of the

proportion of adults and children who are overweight or obese, and an analysis of blood pressure and chemistry for adults.

The link between smoking and lung problems, most notably cancer, is now irrefutable, and effectively planning health care for a population means having adequate information about smoking to estimate likely health system costs going forward. From the survey results smoking is currently mainly a problem for men, despite the declining prevalence of daily smoking and average consumption amongst young men, and indeed, young people generally. But Qatar needs to plan for a continuing, and possibly worsening “bulge” of male smoking-related health problems for its citizens in their middle to old age. Tobacco Free Youth campaigns could hasten the decline in smoking. Successful smoking cessation initiatives could significantly affect Qatar’s economy due to the potential positive health impacts related to reductions in illness related to lost time in the workplace. Smoking cessation and banning smoking in public places could cut down passive smoking too; these have been successful elsewhere. However the WHO World Health Survey smoking results are not consistent with findings from the 2007 WHO Global Youth Tobacco Survey (2007 GYTS) completed in Qatar. In a survey completed in 2000, Qatar reported the estimated smoking rate among adult males to be 42%. More research is needed to reconcile conflicting evidence and to understand the true magnitude of the health-related problems linked to smoking. Results from the 2007 Qatar Global Youth Tobacco Survey indicate that as many as 20% of youths use tobacco in Qatar. Smoking statistics from Qatar are representative of the GCC and Eastern Mediterranean Region; however, smoking among adults (estimated 38%) is considered to be twice as common as in Europe or North America where smoking among adults has been estimated to be 20%[*].

Physical activity and diet can be considered as more of a threat than smoking behaviour in Qatar because poor diets and obesity are on the increase, and low levels of activity are particularly a problem amongst women. More than 50% of the adult population (aged 18+ years) have levels of activity that are insufficient; making this a key policy area even for young people. Given these tendencies, it is not surprise that diet is uniformly poor, leading to almost a third of the population obese across the sex, age and wealth spectrum. Childhood obesity increases with age, and despite some questions over the survey data, all the indications are that children’s weight levels are unacceptably high. Hypertension is estimated at nearly 20% in men, high cholesterol is common and other blood chemistry results do not point to a good level of underlying health, with implications for multiple burdens on the health system as well as a compromised workforce. Health information campaigns to simultaneously improve consumption of fruit and vegetables, improve exercise levels and target women’s health, diet and activities are now urgently needed.

10.2.4 Patterns of ill-health

Survey results on the illness patterns in Qatar provide a very solid basis for health infrastructure and human resource planning. Prevalence figures for conditions such as cancers, asthma, depression, cataracts, diabetes, lung disease, tooth decay, and injuries are summarized in Table 10.1 below. Associated survey questions on levels of medication and screening reveal key issues for public health policy.

---

[*] WHO Global Youth Tobacco Survey, 2007: National Health Authority, Qatar, Noncommunicable Disease Section.

Table 10.1 The health of Qatar's population by condition, 2006

Condition	Overall prevalence measure (per 10 000 population)	Percentage of population with condition	
		Males	Females
Arthritis	7.8	4.8	10.8
Angina	0.8	1.1	0.5
Diabetes	8.3	8.1	8.5
Asthma	3.2	2.3	4.2
Depression	1.9	0.9	2.9
Stroke	0.5	0.4	0.6
Lung disease	0.9	0.9	1
Cancer	0.5	0.3	0.8
Cataracts	16.8	17.4	15.9
Tooth decay	23.2	18.9	27.6
Major injuries	3.2	3.8	2.5

In summary, arthritis is common in women (one in 10), more than one in three people with angina and stroke are not on medication, diabetes has reached 12% among Qatari nationals, asthma levels are at 2%–4% (more among nationals), and depression prevalence is up to 3%, mainly among women. Further investigation is needed into why many people in Qatar who suffer from a noncommunicable disease are not taking medication—the extent to which this represents unmet need for medicine is not clear. Reproductive cancers are the most common, but the numbers of sufferers are small. However, screening for women's cancer looks to be inadequate, especially breast cancer screening. Problems with teeth including tooth decay are very common, especially among women, but the level of treatment for cataracts is high. Finally injuries are worryingly prevalent, particularly road traffic accidents among young men (2%) and home injuries among women.

Maternal, reproductive and child health services are generally good, with wide coverage in the population, although there are gaps. Condom use is low, only 70% of women have the recommended PAP smears and 17% have insufficient levels of antenatal care given the 12 visits that the Qatar health system aspires to. However the overwhelming majority of women have at least three antenatal visits and are assisted by a skilled attendant at childbirth. For children there are high treatment rates for common conditions but there is more work to be done to ensure that child vaccination cards are effectively distributed to record vaccinations.

10.3 The Qatari health system

Turning from the health characteristics of the population in Qatar, to the way that the health system is set up to tackle health problem, this section provides information on health services in Qatar and how they are governed and funded. Since the publication of the WHO 2000 *World health report*, which emphasized the importance of the health system and the measurement of its performance, indicators and survey instruments have been developed to gauge key aspects of the public health structure and response. These

findings are as important as the findings on health, representing the nature and extent of investment in health.

The development of social services, including health care, accelerated after the accession in 1972 of Shaykh Khalifa ibn Hamad, who dramatically altered the allocation of oil revenues. This included transferring the ruler's 25% of oil revenues to the state budget. But the health budget subsequently suffered because of the downturns in oil revenues. In 1986, for example, there were cuts in clinic staff. However, the National Health Authority has tried with some success to ensure that health system provision keeps pace with an expanding population. As of 1985 there were three hospitals, with a total of 885 beds, and 20 health centres. In 1990 there were 2.9 hospital beds per 1000 inhabitants. The nurse to doctor ratio was 1.5 to 1 in 1992. As of 2001 there were 2.2 physicians, 4.9 nurses, 0.4 dentists, and 0.9 pharmacists per 1000 people. All the population have access to health care services.

Survey results supplement these previous findings in three important ways. First, the survey seeks to determine the coverage of health services in relation to need and, subsequently, the levels of satisfaction expressed by individuals in Qatar. Finally the level of vulnerability of people in Qatar to financial problems related to their own health problems is documented together with the health system supports and institutional mechanisms for the financial protection for health in Qatar.

10.3.1 Responding to health needs

A triumph of the Qatari health system is revealed by the survey finding that a full two-thirds of the population have needed some form of health services within the three years before the survey and almost all have received care. Indeed 11.2% of respondents needed to go into hospital in the previous year alone. Mainly this has been government care (particularly maternity care) at free public hospitals. Not surprisingly this is the most commonly accessed service, although private hospitals are not an insignificant proportion of total service use. Private care is used particularly by men, nationals and Doha residents. On a more negative note waiting times have apparently increased in the two years since the survey—and these were already lengthy.

10.3.2 Satisfaction levels

Satisfaction levels expressed by survey respondents, often referred to as the responsiveness of health services, is measured separately for a range of aspects of satisfaction, namely autonomy, choice, communication, confidentiality, dignity, amenities, and promptness. On the whole the news is positive, with high levels of satisfaction. However, there is room for improvement—for example women often complain of a lack of prompt attention. This may be an important area for improvement, given the worse experience of health for women compared with men. It should be noted that there have been some changes to the system since the survey was administered.

10.3.3 Financial protection for health

As government income has increased in the wake of the oil price boost, Qatar has been able to provide free health care to all nationals and expatriates. However, rising costs and increased pressure on the budget led the government in 1999 to require expatriates to purchase health cards. The costs are still low and do not come close to meeting the actual cost of health provision, but their introduction signalled a shift in government policy. The country is currently actively pursuing an alternative system of health care financing through health insurance and has established several new private hospitals.

From the survey it was surprising to find that health expenditure is high among the population; 19% of households spent over 10% of their non-subsistence spending on health care, and 5% of households spent over 40% on health care, a level that is often referred to as “catastrophic”. It should be noted, however, that for a rich household, even catastrophic levels of expenditure may be easily coped with, but more worryingly there were signs from the survey that some families had been pushed into poverty by health care costs. The largest component of these costs includes dentistry and glasses/hearing aids, and Qataris spend more in this respect than non-Qataris, probably partly as a result of their older age structure. Most of the funding for this comes out of people’s current income, compared with much smaller amounts from individual’s savings and borrowing. Furthermore, informal care for an unwell relative or friend either inside or outside of the household is an important part of health care—indeed around 10% of households provide some sort of care. These aspects of health financing should be investigated further.

11. References

FAO (Food and Agriculture Organization of the United Natons; 2003). *Projections of tobacco production, consumption and trade to the year 2010*.

Harpham T, Grant E, Thomas E (2002). Measuring social capital within health surveys: key issues. *Health policy and planning*, 17(1):106–11.

Jha P, Ranson MK, Nguyen S, Yach D (2002). Estimates of global and regional smoking prevalence in 1995, by age and sex. *American journal of public health*, 92(6):1002–6.

Kawachi I, Kennedy B, Lochner K, Prothrow-Stith D (1997). Social capital, income inequality, and mortality. *American journal of public health*, 87:1491–8.

Kirkcaldy B, Furnham A, Veenhoven R (2005). Health care and subjective well-being in nations. In Antoniou ASG, Cooper CL (eds). *Research companion to organizational, health psychology*. Cheltenham UK, Edward Elgar Publishers, 2005. At http://www2.eur.nl/fsw/research/veenhoven/Pub2000s/2005h-full.pdf.

Longmore M, Wilkinson I, Turmezei T, Cheung CK (2007). *Oxford handbook of clinical medicine*, 6th revised edition. Oxford, Oxford University Press.

Peden M, Scurfield R, Sleet D, Mohan D, Hyder A, Jarawan E, Mathers C, eds. (2004). *World report on road traffic injury prevention*. Geneva, World Health Organization.

Valentine NB, Ortiz JP, Tandon A et al. (2003). Patients' experiences with health services: population surveys from 16 OECD countries. In C.J. Murray CJ, Evans DB, eds. *Health system performance assessment: debates, methods and empiricism*. Geneva, World Health Organization.

Van't Veer P, Jansen MCJF, Klerk M, Kok FJ (1999). Fruit and vegetables in the prevention of cancer and cardiovascular disease. *Public health nutrition*, 3(1):103–7.

Veenstra G (2002). Social capital and health (plus wealth, income inequality and regional health governance). *Social science and medicine*, 54(6):849–68.

WHO (2003). *Diet, nutrition and the prevention of chronic diseases*. Report of a Joint WHO/FAO Expert Consultation. Geneva.

WHO (2006). Preparing for the introduction of HPV vaccines: policy and programme guidance for countries. Geneva.

WHO (2006). *Core health indicators for Qatar*. At http://www.who.int/whosis/database/core/core_select_process.cfm?country=qat&indicators=nha).

WHO (2008a). *Why does childhood overweight and obesity matter?* At www.who.int/dietphysicalactivity/childhood_consequences/en/index.html. Accessed 16/05/08.

WHO (2008b). WHO Anthro software and macros. At www.who.int/childgrowth/software/en/index.html. Accessed 16/05/08.

WHO (2008). *WHO global infobase: the impact of cancer in your country—Qatar report*. At http://www.who.int/infobase/report.aspx?rid=126, accessed 16/05/08.

World Bank (1999). *Curbing the epidemic: governments and the economics of tobacco control*. Washington DC.
